# Virtual Screening, Synthesis and In Vitro Characterization of Histamine H_4_ Receptor Ligands Based on Pyrimidine Scaffolds

**DOI:** 10.3390/ijms27156892

**Published:** 2026-08-01

**Authors:** Olga Michalak, Marcin Cybulski, Piotr Krzeczyński, Oliwia Zegrocka-Stendel, Małgorzata Dutkiewicz, Dorota Dymkowska, Agnieszka Olejarz-Maciej, Tadeusz Karcz, Mariam Dubiel, Pakhuri Mehta, Marek Kubiszewski, Marcin Lorkowski, Jakub Jakowiecki, Paweł Pasznik, Przemysław Miszta, Holger Stark, Katarzyna Koziak, Sławomir Filipek

**Affiliations:** 1 Pharmacy, Cosmetic Chemistry and Biotechnology Research Group, Łukasiewicz Research Network-Industrial Chemistry Institute, 8 Rydygiera Str., 01-793 Warsaw, Polandpiotr.krzeczynski@ichp.lukasiewicz.gov.pl (P.K.); 2 Department of Biochemistry and Nutrition, Centre for Preclinical Research and Technologies, Medical University of Warsaw, 1b Stefana Banacha Str., 02-097 Warsaw, Polandkatarzyna.koziak@wum.edu.pl (K.K.); 3 Laboratory of Cellular Metabolism, Nencki Institute of Experimental Biology, Polish Academy of Sciences, 3 Ludwika Pasteura Str., 02-093 Warsaw, Poland; 4 Department of Chemical Technology and Biotechnology of Drugs, Jagiellonian University Medical College, 9 Medyczna Str., 30-688 Kraków, Poland; 5 Institute for Pharmaceutical and Medicinal Chemistry, Heinrich Heine University Düsseldorf, Universitaetsstrasse 1, 40225 Duesseldorf, Germany; 6 Faculty of Chemistry, Biological and Chemical Research Centre, University of Warsaw, 1 Pasteura Str., 02-093 Warsaw, Poland; 7 Analytical Research Section, Pharmaceutical Analysis Laboratory, Łukasiewicz Research Network–Industrial Chemistry Institute, 8 Rydygiera Str., 01-793 Warsaw, Poland

**Keywords:** histamine, histamine H4 receptor, H_4_R, 4-methylhistamine, virtual screening, radioligand binding, cAMP accumulation, β-arrestin recruitment, agonist, antagonist, cytosolic calcium influx

## Abstract

Histamine is a biologically active monoamine acting through four *G* protein-coupled receptors (H_1_R–H_4_R), which represent attractive therapeutic targets for a range of diseases. Nowadays, H_4_R is recognized as a key player in inflammation and cancer. Here, we describe the design, syntheses and characterization of new H_4_R ligands containing pyrido[2,3-*d*]pyrimidine or pyrimidine scaffold. Candidate structures were scored in silico by docking to the structure of the human inactive H_4_R. Favorable structures were synthesized and, after confirmation of identity, their affinities were verified in in vitro screenings. Tested compounds did not exhibit any relevant cytotoxic (PrestoBlue) or antiproliferative (BrdU incorporation) effects at concentrations used in the functional assays. In a radioligand displacement assay (H_4_R affinity) the pyrimidine series showed lower binding, whereas several pyrido[2,3-*d*]pyrimidine derivatives and one pyrimidine analog produced >60% inhibition at 1 µM. Two similar compounds with the highest and moderate affinities were selected for further studies. In the *G*_i_–cAMP pathway, both compounds behaved as moderate H_4_R antagonists, but in contrast, their profiles diverged in the β-arrestin pathway. These findings suggest ligand-dependent biased differences in signaling across *G*-protein and β-arrestin pathways at H_4_R. Overall, **YAN-153** emerges as a promising lead structure for further optimization and for more detailed in vitro (e.g., metabolic stability, selectivity) and in vivo studies.

## 1. Introduction

Histamine is an important biogenic amine that acts as a neurotransmitter and local mediator in numerous physiological and pathological processes. Its effects are mediated through four *G* protein-coupled receptors (H_1_R–H_4_R), which differ in tissue distribution, signaling properties and pharmacological profiles [1,2]. These receptors belong to the rhodopsin-like GPCR family and have emerged as attractive targets for the treatment of allergic, inflammatory, neurological and neoplastic diseases.

The human H_4_ receptor (H_4_R) was identified in 2000–2001 by several groups, based on its high sequence similarity to the histamine H_3_ receptor (H_3_R) [3,4,5,6,7]. Despite this homology, the expression pattern of H_4_R is clearly distinct, which indicates different physiological roles [8]. H_4_R is mainly expressed in cells and tissues associated with immune and inflammatory responses, including eosinophils, mast cells, monocytes, lymphocytes and macrophages [9,10,11,12]. In line with this distribution, H_4_R has been linked to the regulation of chemotaxis, cytokine release and other functions of immune cells.

Since its discovery, H_4_R has been associated with the proper functioning of the immune system and with a variety of inflammatory and allergic conditions. H_4_R expression has been demonstrated in synovial cells from patients with rheumatoid arthritis (RA) [13] and in inflammatory epidermal dendritic cells (IDECs) in the skin [14].

In mouse models, H_4_R has been shown to play a role in allergic lung inflammation by modulating T-cell responses [15]. Collectively, these findings support the involvement of H_4_R in diseases such as atopic dermatitis, asthma and chronic arthritis [13,14,15,16].

More recently, H_4_R has also been implicated in cancer. Immunohistochemical studies and gene expression analyses indicate that H_4_R is differentially expressed in primary tumors compared with corresponding normal tissues, suggesting a role in carcinogenesis [17,18,19]. H_4_R has been detected in various cancer cell lines and biopsy specimens, including breast cancer, leukemia, lymphoma, kidney cancer and lung cancer [19]. In a mouse model of triple-negative breast cancer, H_4_R deficiency reduced tumor growth and lung metastases, further underscoring its potential as a target in oncology [20].

**Figure 1:**
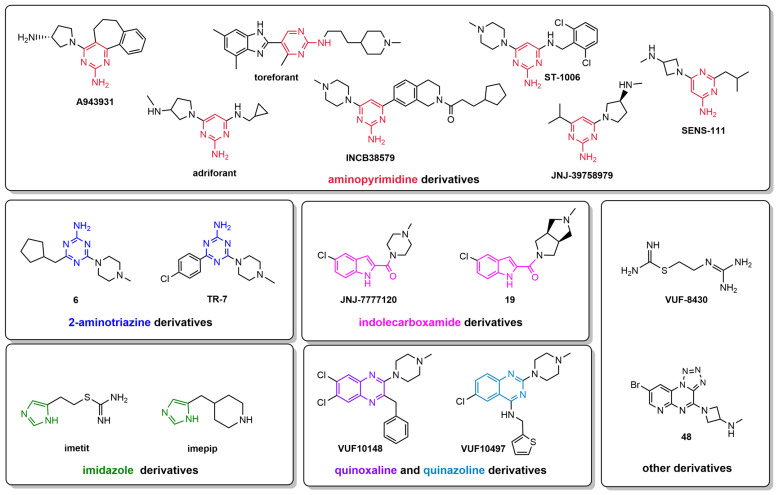
Known H_3_R and H_4_R ligands containing: aminopyrimidine, 2-aminotriazine, indolecarboxamide, imidazole, quinazoline and quinoxaline scaffolds capable of interacting with H_4_R. The numbering of compounds, if names are not present, are cited from references they are presented in.

The recognition of H_4_R as a key player in inflammation and cancer has stimulated intensive research on H_4_R ligands for potential use in inflammatory, allergic and autoimmune diseases, as well as for their analgesic effects. Since the early 2000s, numerous H_4_R ligands have been described, including agonists, antagonists and inverse agonists. However, the high similarity in amino acid sequence between H_3_R and H_4_R led to the hypothesis, later confirmed, that many known H_3_R ligands are also capable of interacting with H_4_R [21]. As a result, H_4_R selectivity remains a major challenge, and there is a need for new chemotypes that provide favorable pharmacological profiles at H_4_R with reduced H_3_R liability. Promising results have been obtained for compounds containing imidazole, indolecarboxamide, aminopyrimidine, quinazoline and quinoxaline scaffolds (Figure 1) [21]. Examples include imidazole derivatives such as imepip and imetit, which are structurally related to histamine and act as potent agonists with high affinity for H_3_R and H_4_R [22,23,24,25]. Other noteworthy ligands are ST-1006, a 2-aminopyridine derivative acting as an agonist at both receptors [26], and VUF-8430, an aliphatic H_4_R agonist with high affinity [27].

In parallel, there has been increasing interest in H_4_R antagonists and inverse agonists. The first reference compound, **JNJ-7777120**, showed efficacy in preclinical models of asthma, allergic rhinitis and pruritus [28]. Subsequent H_4_R antagonists displayed anti-inflammatory effects in various in vivo models, including dermatitis (INCB38579 [29]), peritonitis (TR-7 [30], A-943931 [31]) and atopic dermatitis (adriforant and related compounds [32,33]). Compound **6** is another H_4_R antagonist with demonstrated analgesic effects [34]. Several H_4_R ligands, including SENS-111 (seliforant) for unilateral vestibulopathy [35], **JNJ-39758979** and adriforant for atopic dermatitis [32,36], and toreforant for rheumatoid arthritis, have advanced to clinical trials. However, many of these studies were terminated due to limited therapeutic benefit or adverse effects.

Modern molecular docking has become a widely used tool in early phase of drug discovery. By placing three-dimensional ligand structures into the receptor binding site and estimating binding energies, docking provides insight into receptor-ligand interactions and supports hit identification and optimization [37,38]. Although the ultimate success of docking-based approaches in bringing new drugs to the market is sometimes questioned, recent improvements in computational methods, increased computing power and broad access to structural databases have greatly enhanced their utility in virtual screening campaigns [39,40].

Despite promising preclinical and early clinical data for H_4_R ligands in indications such as rheumatoid arthritis, asthma, dermatitis and psoriasis, no H_4_R-targeted drug has yet been approved for clinical use [12]. Nevertheless, H_4_R continues to be considered a promising target in the search for new anti-inflammatory and immunomodulatory agents [41,42]. There is still a need for structurally novel ligands that can provide favorable pharmacological profiles, improved safety and, ideally, better selectivity against H_3_R.

In this context, we aimed to design, synthesize and characterize new H_4_R ligands containing pyrido[2,3-*d*]pyrimidine or pyrimidine scaffold. Candidate structures were first scored in silico by docking to constructed 3D structure of the human inactive H4 receptor. Compounds with favorable predicted binding scores were selected for synthesis and in vitro verification. The synthesized compounds were structurally confirmed by NMR and HRMS and then subjected to radioligand binding and functional assays to assess their affinity and intrinsic activity at H_4_R.

## 2. Results and Discussion

### 2.1. Design and Virtual Screening

Quinazoline and quinoxaline scaffolds have been widely explored as chemotypes for the development of histamine H_4_ receptor ligands [21]. Smits et al. demonstrated that a quinoxaline fragment could be successfully incorporated into potent H_4_R ligands [43]. In parallel, fragment-based screening revealed that quinazoline derivatives bind to H_4_R with micromolar affinity, adopting binding modes consistent with the characteristic three-pocket architecture of the receptor [44]. These studies also showed that quinazoline and quinoxaline share low-energy conformations suitable for stabilizing ligand–receptor interactions. Subsequent optimization guided by pharmacophore modeling and in silico calculations yielded quinazoline derivatives acting as inverse agonists at rat H_4_R and displaying anti-inflammatory activity in vivo. Further QSAR-driven optimization led to the identification of quinazoline sulfonamides as H_4_R inverse agonists with enhanced anti-inflammatory properties [45].

Additional evidence supporting the relevance of bicyclic nitrogen-containing heterocycles for H_3_/H_4_ receptor modulation comes from studies on quinoline-based structures. Depending on substitution patterns, these compounds were characterized as H_3_R inverse agonists [46] or antagonists [47]. Collectively, these reports indicated that heteroaromatic systems with electron-rich bicyclic cores are well-suited for binding within the H_4_R orthosteric pocket.

Guided by these observations, we designed two novel structural series. The first series comprises pyrido[2,3-*d*]pyrimidine derivatives, representing an unexplored bicyclic scaffold with structural analogy to quinazoline and quinoxaline, yet bearing distinct physicochemical properties. The second series includes pyrimidine-substituted pyridines, selected for their more linear, flexible topology, which was hypothesized to facilitate accommodation within the H_4_R binding site and adjacent sub-pockets [48,49].

Both series were subjected to comprehensive computational evaluation, including docking and molecular dynamics simulations. Ligands were prepared in LigPrep (Schrödinger suite of programs) generating relevant protonation states and stereochemical variants. Docking to the human H_4_ receptor was performed using Glide XP, (Schrödinger suite of programs) followed by IFD-MD (Schrödinger suite of programs) refinement, enabling simultaneous optimization of ligand poses and receptor side-chain conformations, while accounting for water-mediated interactions and desolvation effects. Top-ranked poses were subsequently evaluated in 20 ns molecular dynamics simulations in an explicit POPC/cholesterol membrane environment to assess binding stability and interaction persistence.

**Figure 2:**
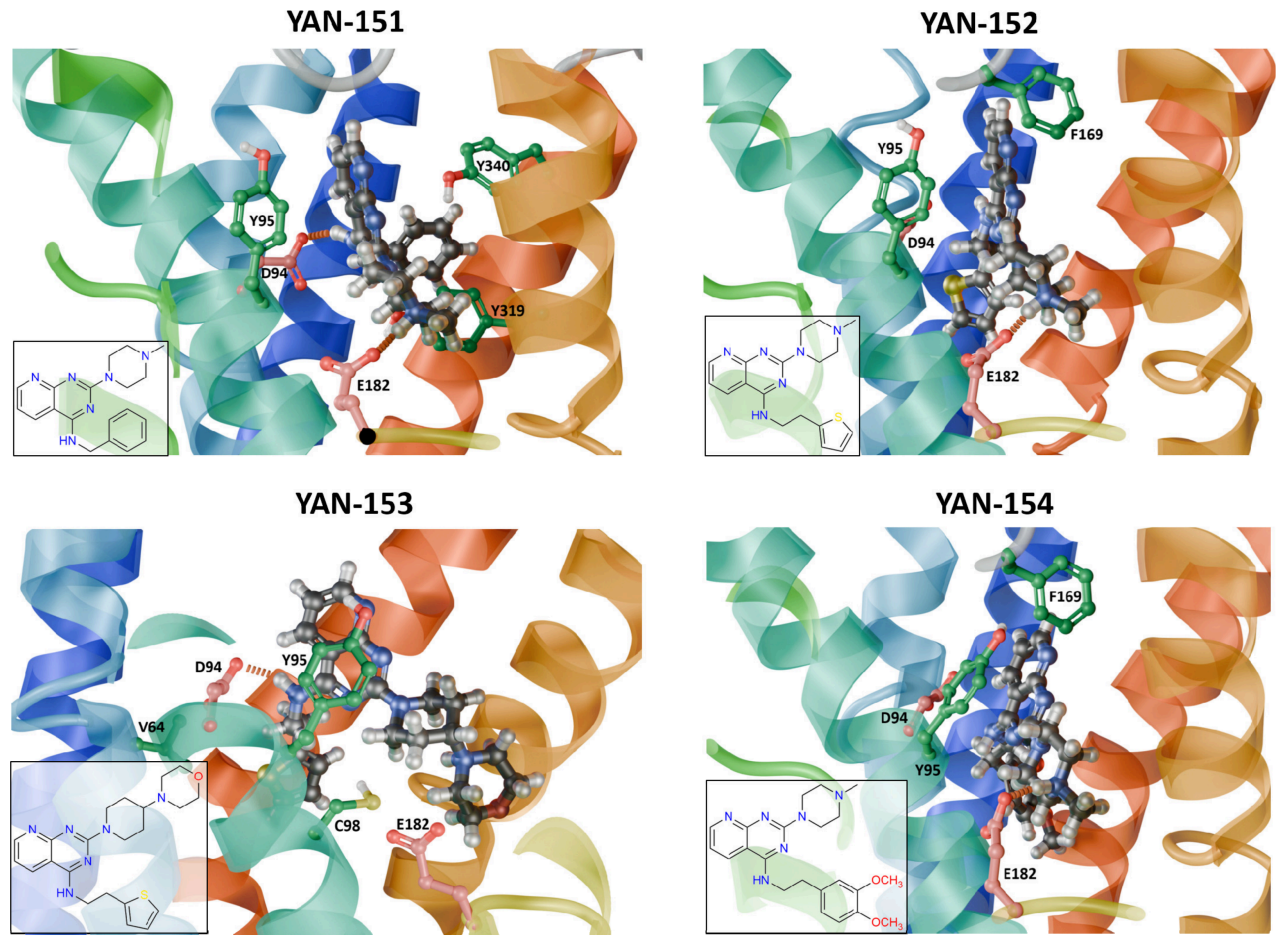
The final structures of **YAN-151** to **YAN-154** docked to human H_4_ receptor. The ligands are colored orange; the residues D94 and E182 are colored purple. Coloring scheme of transmembrane helices: TM1 in blue, TM2 in light blue, TM3 in cyan, TM4 in green, TM5 in yellow, TM6 in orange and TM7 in red.

The final structures of compounds **YAN-151** to **YAN-154** from docking and then 20 ns MD simulations are shown in Figure 2.

The final poses of **YAN-151** to **YAN-154** are similar, with the protonated and positively charged amine group bound to E182^5.46^ via ionic interactions, but **YAN-153** without a hydrogen bond to E182^5.46^. **YAN-151** and **YAN-153** additionally form a hydrogen bond with D94^3.32^. There are also many hydrophobic and π-π interactions especially with Y95^3.33^ and F169^ELC2^. **YAN-152** does not form a hydrogen bond with D94^3.32^ but the hydrophobic and π-π interactions are similar to **YAN-151** since they both residue on the same binding site and their aromatic ring system is nearly in the same place. **YAN-153** forms especially strong π-π interaction of its quinazoline structure with Y95^3.33^, and additionally its thiophen ring interacts with several residues including V64^2.53^ and C98^3.36^. **YAN-154** does not form a hydrogen bond with D94^3.32^ but the π-π interaction of its quinazoline structure with Y95^3.33^ is also strong as for **YAN-153**.

### 2.2. Docking and MD Results for ***YAN-155*** to ***YAN-159*** and ***JNJ-7777120***

**Figure 3:**
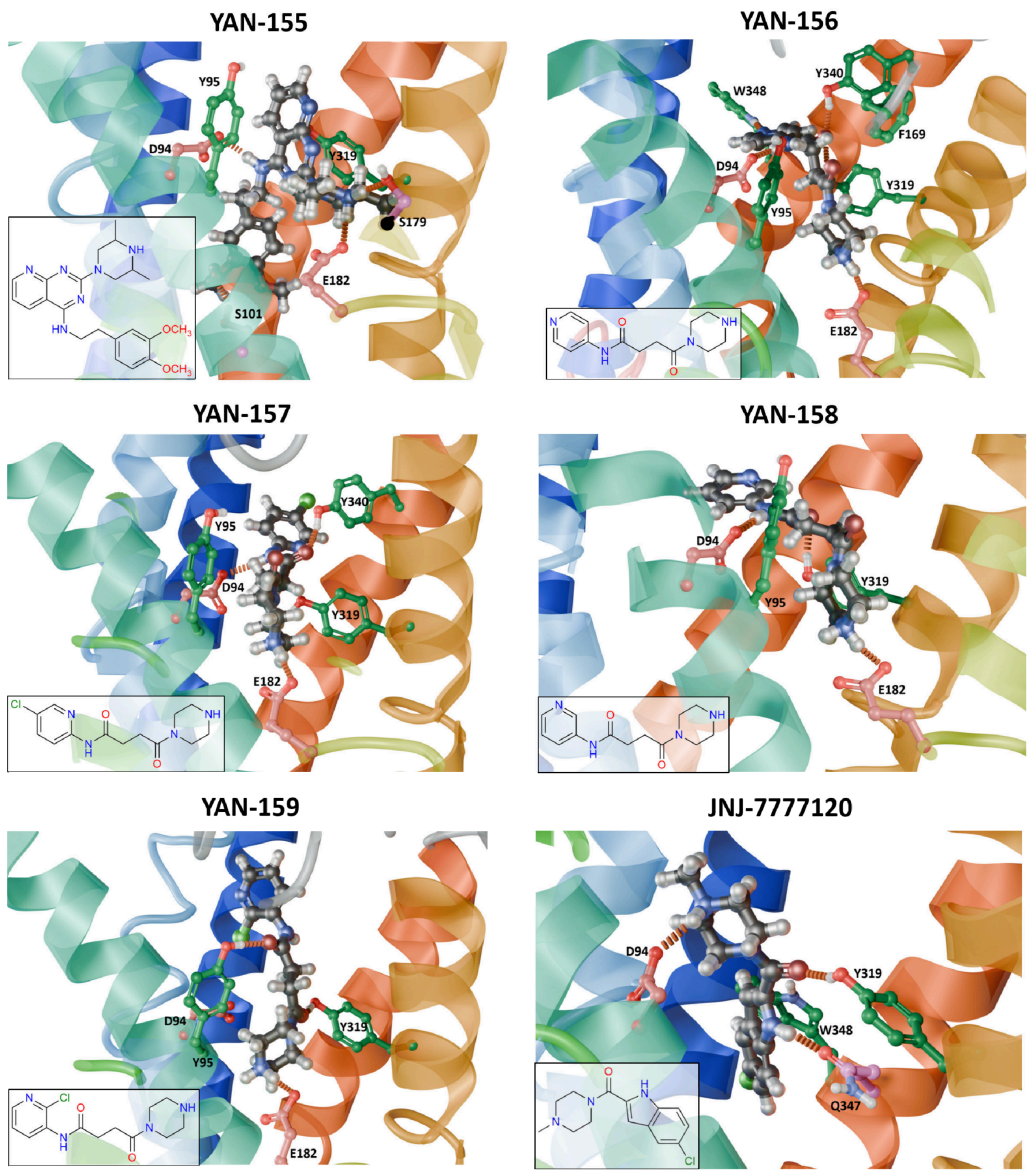
The final structures of **YAN-155** to **YAN-159** and **JNJ-7777120** docked to human H_4_ receptor. The ligands are colored orange; the residues D94 and E182 are colored purple. Coloring scheme of transmembrane helices: TM1 in blue, TM2 in light blue, TM3 in cyan, TM4 in green, TM5 in yellow, TM6 in orange and TM7 in red.

The docking poses of **YAN-155** to **YAN-159** and the reference antagonist **JNJ-7777120** are shown in Figure 3.

All **YAN** compounds maintain an ionic interaction between their protonated amine and E182^5.46^, consistent with the binding mode of the previously described ligands in this series. **YAN-155** forms three hydrogen bonds (to D94^3.32^, S101^3.39^ and S179^5.43^), in addition to hydrophobic and aromatic contacts. **YAN-156** forms hydrogen bonds with D94^3.32^, Y319^6.51^, Y340^7.35^ and W348^7.43^ but displays minimal π–π stacking. **YAN-157** exhibits a similar pattern but engages fewer residues in hydrogen bonds (D94^3.32^ and Y340^7.35^). **YAN-158** interacts through hydrogen bonding with D94^3.32^ and Y319^6.51^, whereas **YAN-159** forms hydrogen bonds with Y95^3.33^ and Y319^6.51^, suggesting a partially shifted binding mode. **JNJ-7777120** displayed several stable binding poses during docking; in the most frequently observed conformation, it forms an ionic interaction with D94^3.32^ and not with E182^5.46^ (contrary to **YAN** compounds). The hydrogen bonds are created with Y319^6.51^ and Q347^7.42^, accompanied by notable π–π stacking with W348^7.43^.

### 2.3. Chemistry

#### 2.3.1. Synthesis of Ligands with a Pyrido[2,3-*d*]pyrimidine

**Scheme 1:**
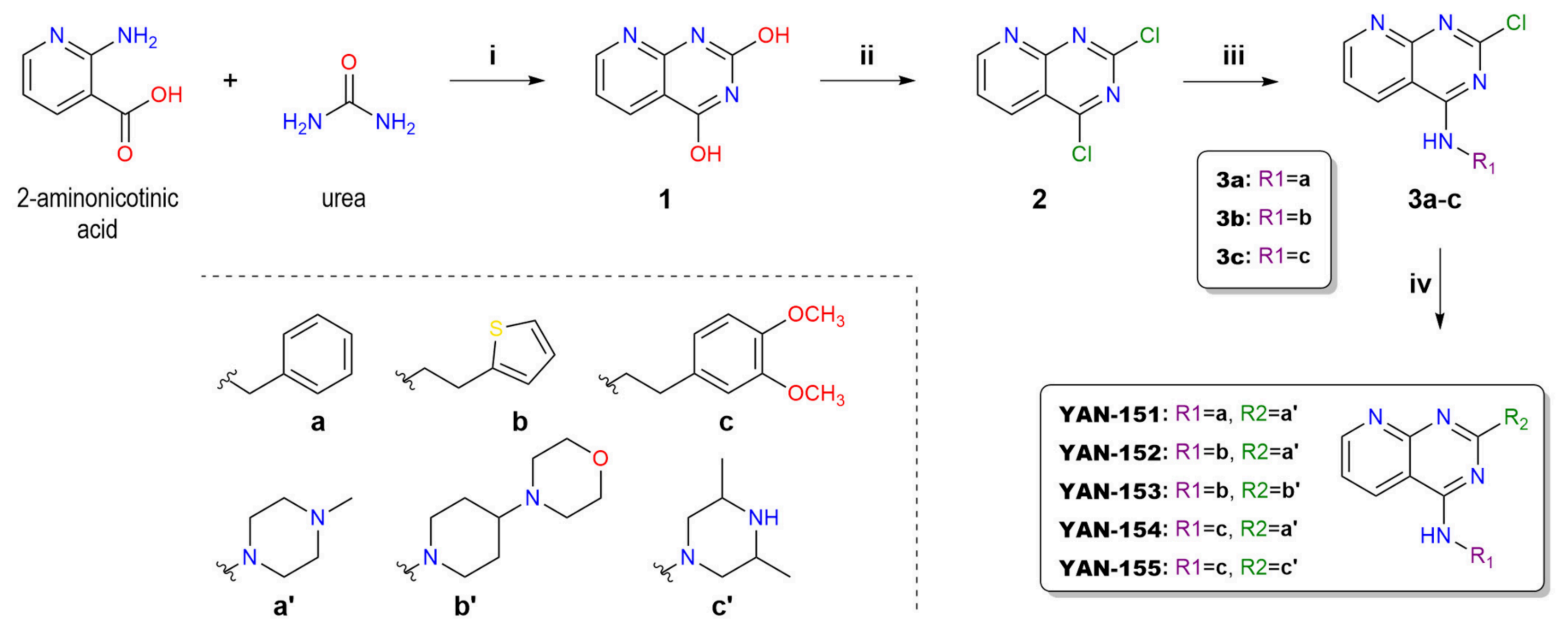
Synthesis of new pyrido[2,3-*d*]pyrimidine derivatives. Reagents and conditions: (i) 180–200 °C; (ii) POCl_3_, DMF, reflux, 3 h; (iii) **a**-NH_2_, **b**-NH_2_ or **c**-NH_2_, TEA, THF, rt.; (iv) **a**′-NH_2_, **b**′-NH_2_ or **c**’-NH_2_, EtOH, reflux.

Compounds **YAN-151** to **YAN-155** were synthesized in four steps according to Scheme 1.

In the first step, 2-aminonicotinic acid was condensed with urea affording pyrido[2,3-*d*]pyrimidine-2,4-diol **1** [50], followed by hydroxyl groups substitution by chloride atoms after treatment with phosphorus oxychloride (POCl_3_) and catalytic amount of *N*,*N*-dimethylformamide (DMF) [51] to yield compound **2**. In the next step, the amine substituents were introduced at the C-4 position of the aromatic ring of **2**. The observed regioselectivity was a result of the different activation energies of the chlorides at C-2 and C-4 in 2,4-dichloropyrido[2,3-*d*]pyrimidine expected for the nucleophilic amine substitution. The regioselective introduction of primary amines (benzylamine **a**-NH_2_, 2-thiophenethanamine **b**-NH_2_ or 3,4-dimethoxybenzeneethanamine **c**-NH_2_) at the C-4 position of **2** was carried out at room temperature in the presence of TEA. In the next step, the amine derivatives from second group, embracing *N*-methylpiperazine **a′**-NH_2_, 4-(piperidin-4-yl)morpholine **b′**-NH_2_ or 2,6-dimethylpiperazine **c′**-NH_2_, were subsequently introduced at C-2 position of aromatic ring. These conversions were carried out using excess amounts of amine substrates in EtOH at elevated temperature. The expected products **YAN-151**–**YAN-155** were obtained in high yields 81–98%.

#### 2.3.2. Synthesis of Ligands with a Pyrimidine Scaffold

**Scheme 2:**
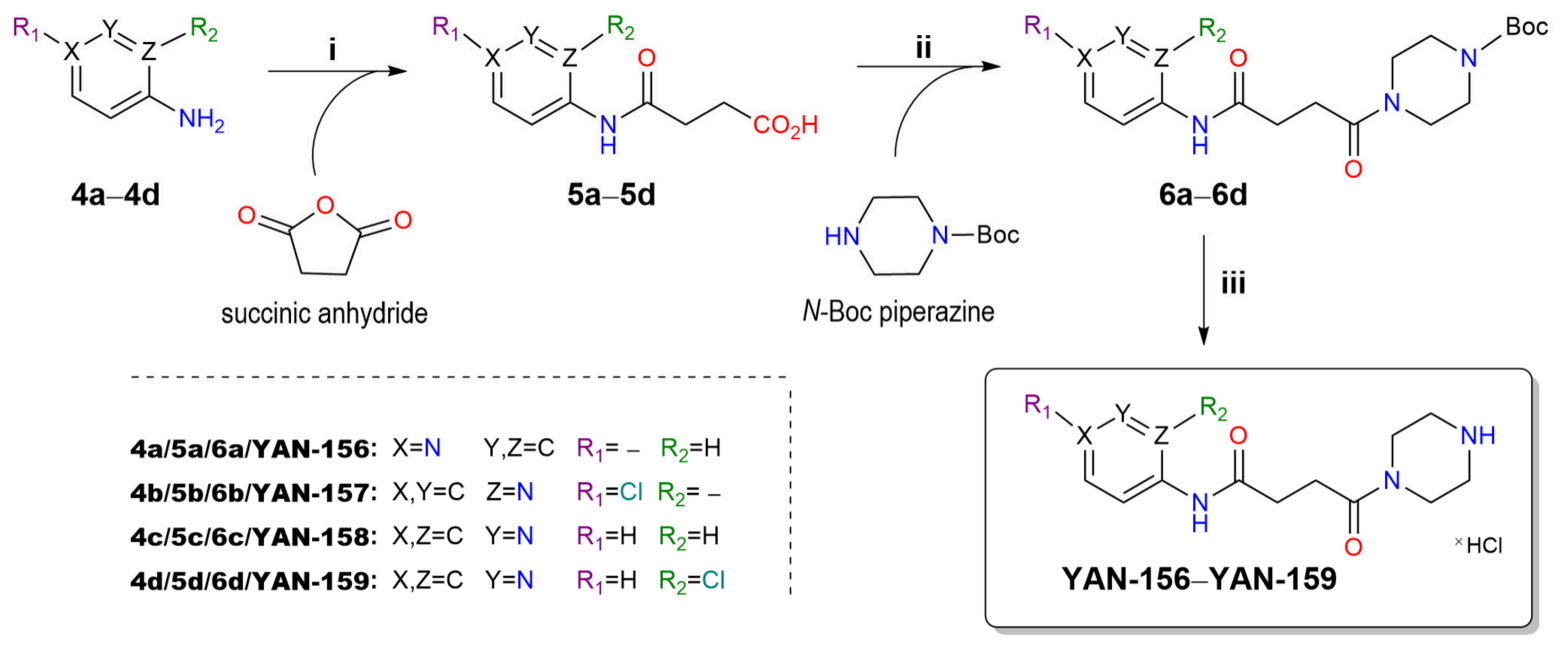
Synthesis of new pyridine derivatives. Reagents and conditions: (i) THF, rt for **5a** and **5c**, reflux for **5b** and **5d**; (ii) EDCI × HCl, DIPEA, DMF, rt, overnight; (iii) 6M HCl in EtOH:AcOEt (1:1 *v*/*v*), rt, 5 min.

Compounds **YAN-156** to **YAN-159** were synthesized in three steps as shown in Scheme 2.

In the first step, a series of aminopyridine derivatives (4-aminopyridine **4a**, 2-amino-5-chloropyridine **4b**, 3-aminopyridine **4c** and 3-amino-2-chloropyridine **4d**) were condensed with succinic anhydride to obtain four 4-oxo-4-(pyridylamino)butanoic acid derivatives (**5a**–**d**). The synthesis of compounds **5a** and **5c** was quick (within 1 h) at room temperature, while the synthesis of derivatives **5b** and **5d** required an increased temperature and a longer reaction time. Products **5a**–**d** were precipitated from the reaction solution. After filtering and drying under vacuum, the intermediates were used for the next step—condensation with *N*-Boc-piperazine. This reaction was carried out in DMF in the presence of DIPEA and EDCI as a coupling reagent. Then, reaction mixtures were extracted using a water/DCM solvent system. The crude products were isolated from the organic layers and then purified by flash chromatography (chloroform/methanol 98:2 → 95:5). The intermediates **6a**–**d** were obtained in yields ranging from 24 to 72%. In the final step, the Boc groups were removed by treating with a 6 M solution of gaseous HCl in EtOH:AcOEt (1:1 *v*/*v*). This deprotection resulted in the formation of the appropriate hydrochlorides **YAN-156** to **YAN-159** as final products.

### 2.4. In Vitro Pharmacological Studies

#### 2.4.1. Radioligand Binding Assay

The binding affinity of compounds **YAN-151**–**YAN-159** at hH_4_R was evaluated in a radioligand binding assay, as previously described [48]. In brief, [^3^H]-histamine (10 nM) was used as the radioligand, and the hH_4_R receptor was expressed in Sf9 cells together with the Gα_i2_ and Gβ_1_γ_2_ subunits of the G protein. Although single-concentration screening at 1 µM does not provide quantitative *K_i_* values, such an approach is widely used for rapid hit identification in early-stage drug discovery [52,53,54]. In our study, it served to prioritize compounds for further functional characterization. The results are expressed as percentage inhibition of specific [^3^H]histamine binding at 1 µM and are summarized in Table 1. The reference antagonist **JNJ-7777120** was not screened under these conditions; it was applied at a saturating concentration (100 µM) to define non-specific binding, which was subtracted from all data. The value of 100% inhibition listed for **JNJ-7777120** in Table 1 therefore represents an operational reference point rather than a potency value measured at the screening concentration and is not directly comparable with the test compounds evaluated at 1 µM. A quantitative comparison with the reference antagonist is instead provided by the functional assays, in which **JNJ-7777120** was tested as a full concentration–response curve (cAMP accumulation, Section 2.4.3) or at the same concentration as the test compounds (Ca^2+^ mobilization, Section 2.4.5).

At the screening concentration of 1 µM, the compounds showed inhibition values ranging from 39% to 70%. This variability was dependent on both the core scaffold and the substitution pattern. In general, the pyrido[2,3-*d*]pyrimidine series exhibited higher inhibition, with **YAN-151**, **YAN-153**, and **YAN-155** exceeding 60%. Within this group, **YAN-153** reached the highest inhibition (69.9%). In contrast, **YAN-152** and **YAN-154**, which share the same aromatic core, displayed clearly lower inhibition, indicating that modifications at R_1_/R_2_ positions strongly influence H_4_R binding.

**Figure 4:**
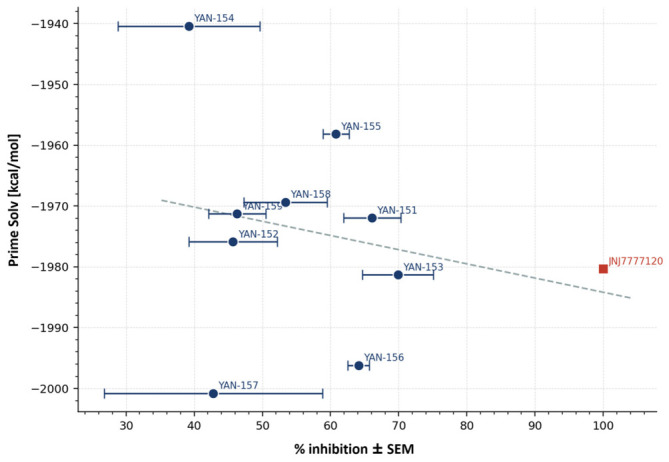
The correlation of experimental % inhibition and the prediction of free energy binding of ligands by the MM-GBSA Solvation method for all **YAN** ligands and **JNJ-7777120**. The Pearson correlation coefficient r = −0.241 and the Spearman rank correlation coefficient ρ = −0.297. The dashed line corresponds to the linear regression fit.

A plot showing the correlation of experimental % inhibition and the free energy prediction of ligand binding is shown in Figure 4.

The free energy values were calculated using the MM-GBSA Solvation method for all **YAN** compounds and the reference ligand **JNJ-7777120,** for which the inhibition was set to 100% and its concentration was 100 times larger than for **YAN**s. Since the probability distribution was not normal, we calculated the Spearman rank correlation coefficient apart from the Pearson correlation coefficient. These coefficients were rather low (r = −0.241 for Pearson correlation, and ρ = −0.297 for Spearman rank correlation) but correctly predicted a direction of changes in ligand binding free energy. There are two outliers, **YAN-154** and **YAN-157**. They have the smallest % inhibition, around 40%, and the largest standard deviation of this value (10.4 and 16.0 respectively). However, the free energy predictions are surprising: **YAN-154** was predicted as the weakest ligand and **YAN-157** as the strongest ligand. Such outliers indicate that the calculated binding energy is too sensitive for small changes in ligand structure and location in the receptor binding site—similar compounds **YAN-157** and **YAN-158** have very different predicted energies but similar % inhibition. Usage of *K*_i_ values instead of % inhibition could improve accuracy of experimental binding data but too sensitive predictions remain. The statistics would be better only with a large number of compounds.

The pyrimidine series (**YAN-156**–**YAN-159**) tended to show somewhat lower inhibition. An exception was **YAN-156** (64.1% inhibition), whose binding at 1 µM was comparable to the most active pyrido[2,3-*d*]pyrimidine derivatives. This suggests that the particular substitution pattern in **YAN-156**, including the position of the nitrogen atom in the pyridine moiety (para position relative to the long-chain substituent), may favor additional interactions within the receptor binding pocket.

Compounds **YAN-152** and **YAN-153** share the same pyrido[2,3-*d*]pyrimidine core and the same R1 substituent (2-(thiophen-2-yl)ethylamino), differing only in the R2 group (*N*-methylpiperazine in **YAN-152** versus 4-(piperidin-4-yl)morpholine in **YAN-153**). This single modification at R2 markedly improved H_4_R affinity in **YAN-153**, highlighting the contribution of the R2 substituent to receptor binding. Therefore, two compounds were selected as a matched molecular pair for detailed functional characterization, allowing the functional consequences of a single, defined structural change to be assessed while contrasting the highest-affinity (**YAN-153**) and a moderate-affinity (**YAN-152**) member of the series. The 60% inhibition value was used only as an initial triage criterion; the pair was deliberately chosen to span it, so that **YAN-152** (45.7%) provides the moderate-affinity reference required for this comparison rather than being excluded. It should be noted that radioligand displacement reports binding affinity but not intrinsic activity, since agonists and antagonists alike compete for the orthosteric site; the functional profile of each ligand was therefore established separately in the assays described below.

#### 2.4.2. Cell Viability and Cell Proliferation Effects

To exclude non-specific effects related to cell death or altered cell growth, all synthesized compounds were first evaluated in cell viability and cell proliferation (BrdU incorporation) assays in CHO-H_4_R cells. At the concentration used in the functional assays (10 µM), none of the compounds affected cell viability or proliferation, and no effects were observed at 100 µM either (Figure S88). At the highest concentration tested (1 mM), a marked loss of viability, accompanied by reduced BrdU incorporation, was observed for compounds requiring 5% DMSO to reach this concentration (**YAN-151**–**YAN-155** and **YAN-157**); however, the vehicle alone at 5% DMSO reduced viability to a comparable extent (DMSO control, Figure S76), whereas **YAN-156**, **YAN-158** and **YAN-159**, applied in 2% DMSO, remained unaffected. The effects seen at 1 mM therefore reflect the vehicle rather than the compounds.

#### 2.4.3. Functional Characterization in cAMP Accumulation Assay

**Figure 5:**
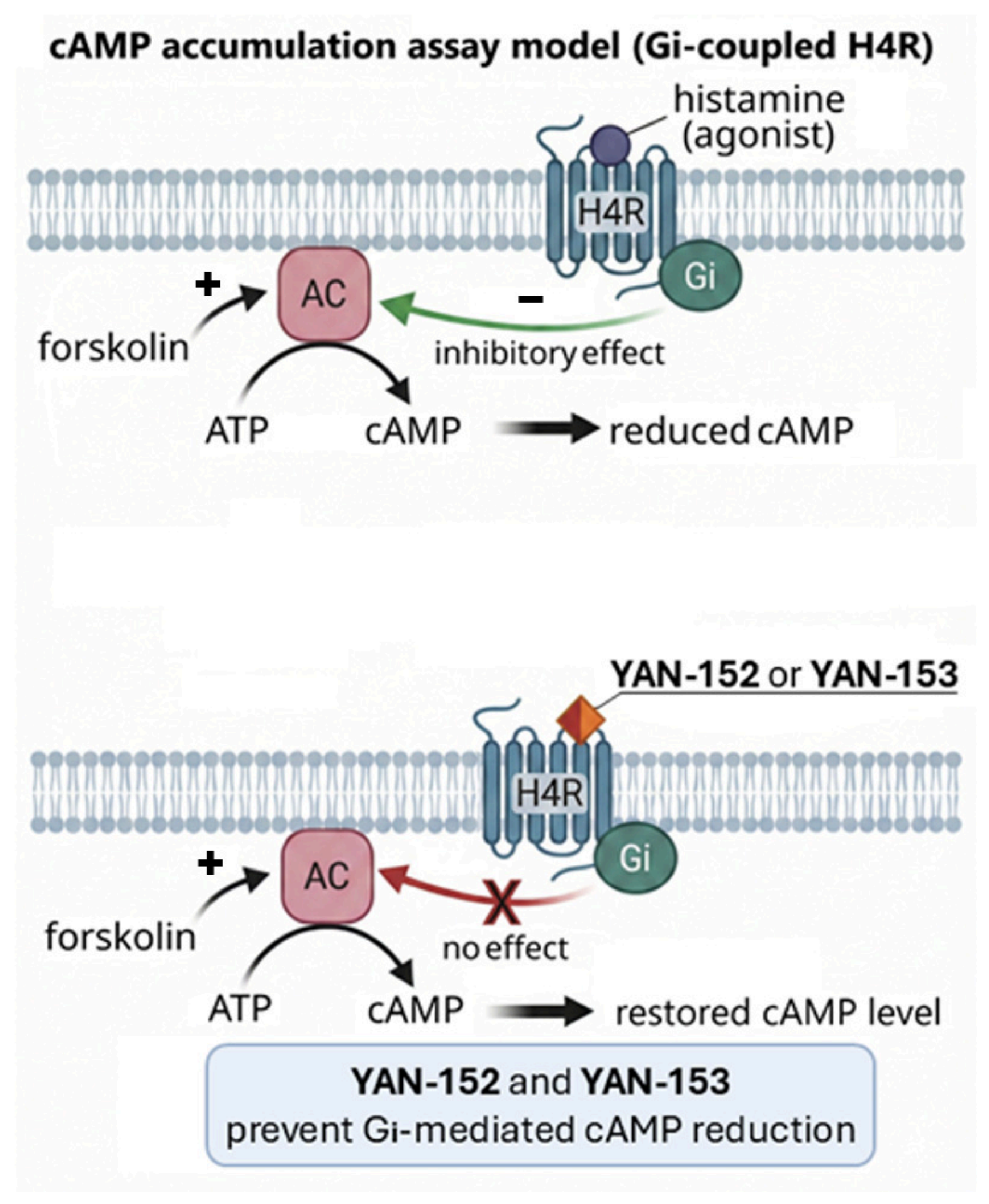
Schematic representation of the cAMP accumulation assay model. Histamine activation of *G*i-coupled H_4_R reduces cAMP levels, whereas **YAN-152** or **YAN-153** block this effect and restore cAMP accumulation in histamine and forskolin co-stimulated CHO-H_4_R cells.

The functional activity of candidate H_4_ receptor ligands was assessed in a cAMP accumulation assay to evaluate their ability to block *G*_i_-coupled H_4_R signaling using an antagonist-mode protocol. Upon H_4_R activation by histamine, *G*_i_ coupling leads to reduced intracellular cAMP; antagonists are therefore expected to prevent this decrease and restore cAMP levels in histamine and forskolin co-stimulated cells, as illustrated in Figure 5.

**Figure 6:**
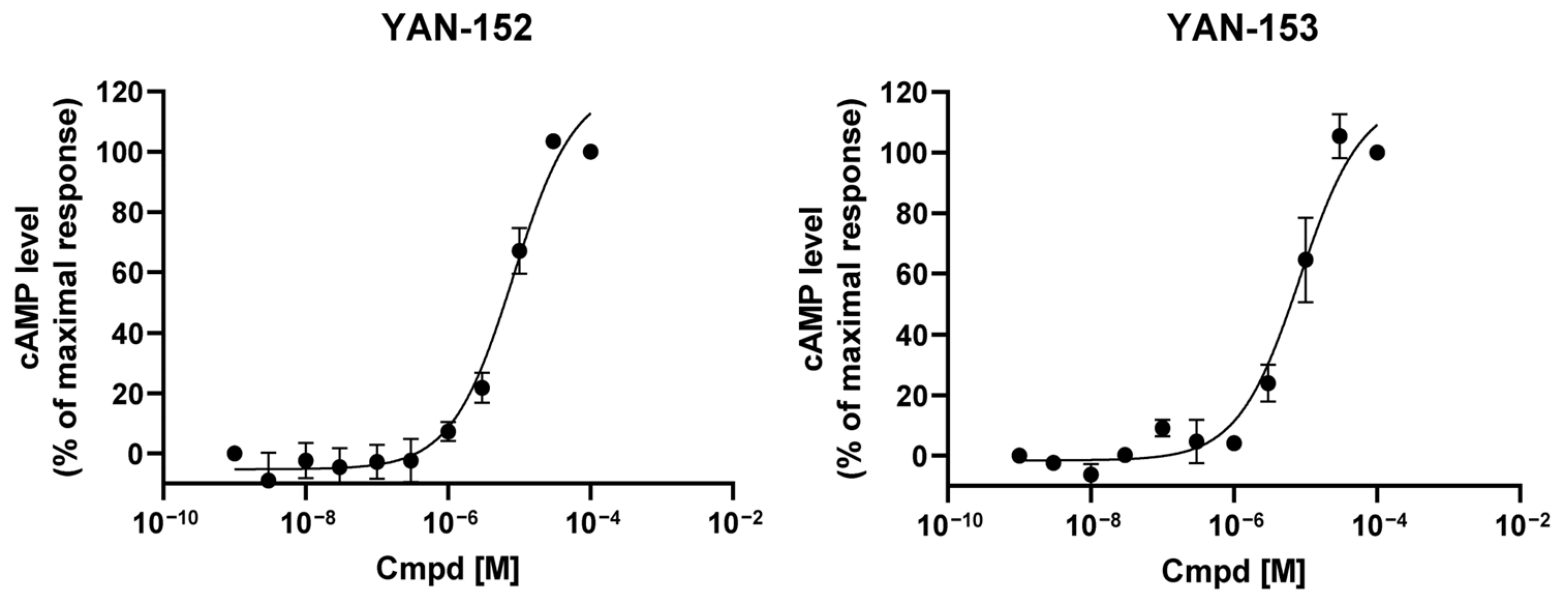
Concentration–response curves illustrating antagonistic effects of **YAN-152** and **YAN-153** on cAMP accumulation in CHO-H_4_R cells. Data are mean ± SD from three independent experiments. Curves were fitted using a four-parameter logistic model.

The functional activity of the tested compounds in the *G*i-coupled H_4_ receptor pathway was evaluated using full concentration-response cAMP accumulation assay in CHO cells stably expressing human H_4_R. **YAN-152** and **YAN-153** were selected as representative examples with the lower and higher binding to hH_4_R, respectively. Both compound concentrations dependently reversed histamine-induced inhibition of cAMP accumulation in forskolin-stimulated cells. The IC_50_ values in antagonist mode were 9.91 ± 3.83 µM for **YAN-152** and 8.95 ± 3.03 µM for **YAN-153**, compared with 0.0833 ± 0.030 µM for the reference antagonist **JNJ-7777120** (Figure 6, Table 2), reflecting the differences observed in the radioligand binding assay.

These results indicate that both compounds act as moderate antagonists of H_4_R in the *G*i-cAMP pathway. While the assay clearly demonstrates antagonistic activity, it does not provide direct information on whether the antagonism is surmountable or non-surmountable; such mechanistic characterization would require additional experiments.

#### 2.4.4. Functional Characterization in β-Arrestin Recruitment Assay

**Figure 7:**
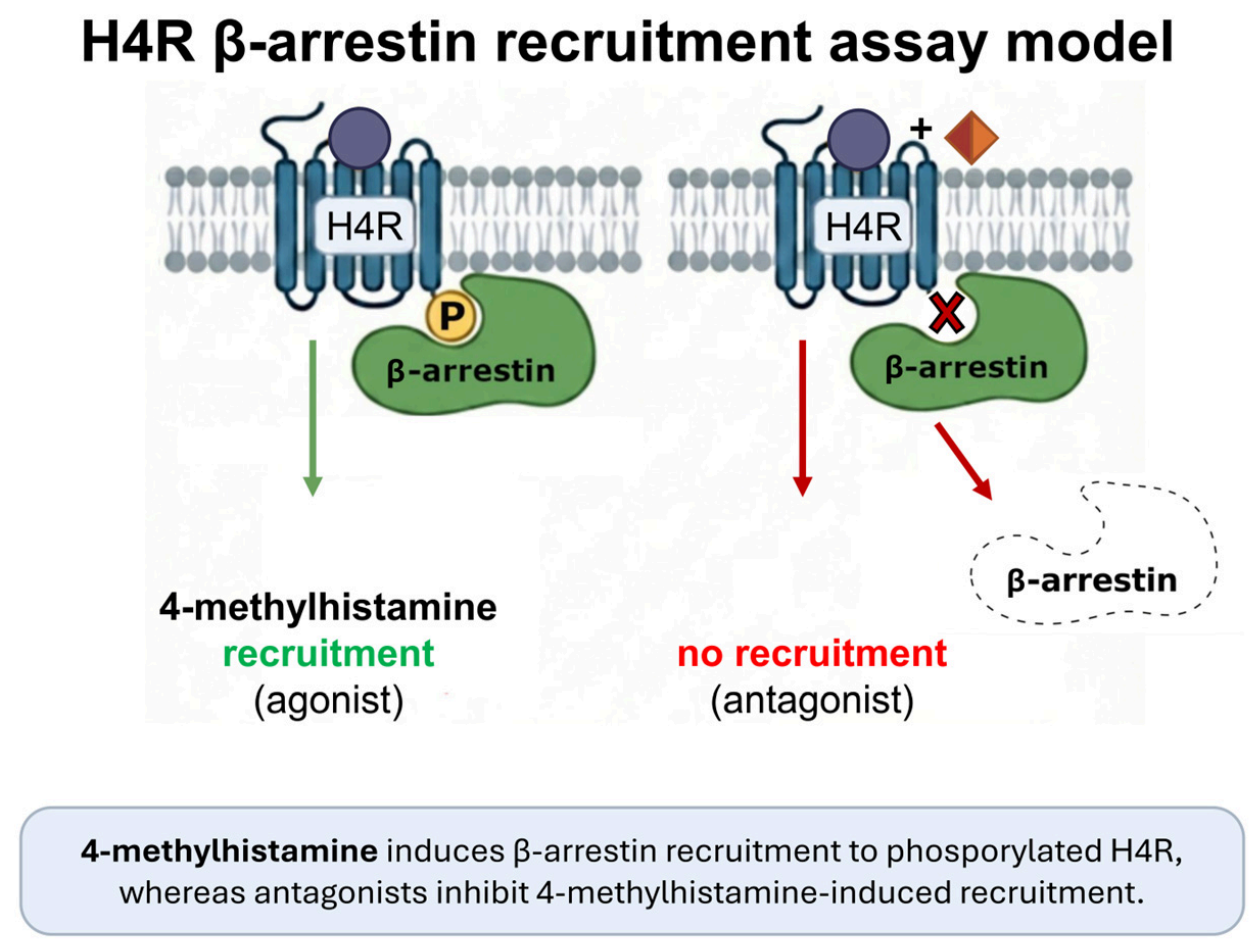
Schematic illustration of β-arrestin recruitment assay model at the H_4_ receptor. Agonists promote β-arrestin recruitment to phosphorylated H_4_R, whereas inverse agonists reverse the agonist-induced effects.

The activity of H_4_R ligands in the β-arrestin pathway was assessed using a cell-based LiveBLAzer assay monitoring β-arrestin recruitment to human H_4_R in Tango H4-bla U2OS cells. In this system, recruitment of β-arrestin to activated human H_4_R triggers the release of a β-lactamase transcription factor, which activates a β-lactamase reporter. Enzymatic activity is detected with a FRET-based substrate, in which cleavage of the β-lactam ring changes the fluorescent signal, providing an indirect measure of β-arrestin recruitment (Figure 7).

**Figure 8:**
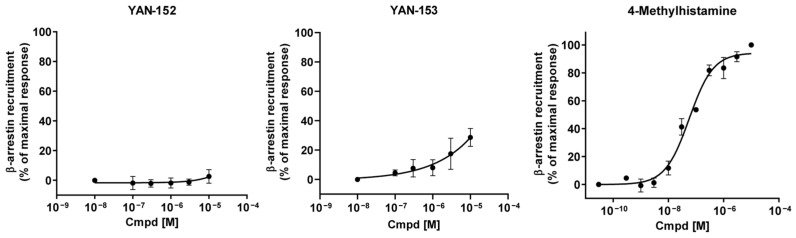
Concentration–response curves for β-arrestin recruitment at human H_4_R in agonist mode for **YAN-152**, **YAN-153** and the reference agonist **4-MH**. Data are mean ± SD from three independent experiments (each performed in triplicate); curves were fitted using a four-parameter logistic model.

Compounds were tested in both agonist and antagonist modes. In agonist mode, **YAN-153** elicited a weak, partial agonist response, reaching 29 ± 6% of the maximal 4-methylhistamine (**4-MH**) effect (E_max_) at the highest concentration tested (10 µM), whereas **YAN-152** produced only minimal activation (3 ± 5%; Table 2, Figure 8). In antagonist mode, **YAN-152** inhibited **4-MH**-induced β-arrestin recruitment with an IC_50_ of 2.37 ± 0.56 µM, while **YAN-153** showed only limited inhibition (IC_50_ > 10 µM).

These findings indicate that **YAN-152** behaves as a predominantly antagonist ligand in the β-arrestin pathway, whereas **YAN-153** displays weak partial agonist properties with poor antagonist efficacy. Considered together with the cAMP data, these results reveal ligand-dependent variation in signaling across *G*-protein and β-arrestin pathways at H_4_R, consistent with the well-established concept of functional selectivity in GPCR signaling [38].

#### 2.4.5. Cytosolic Calcium Influx

**Figure 9:**
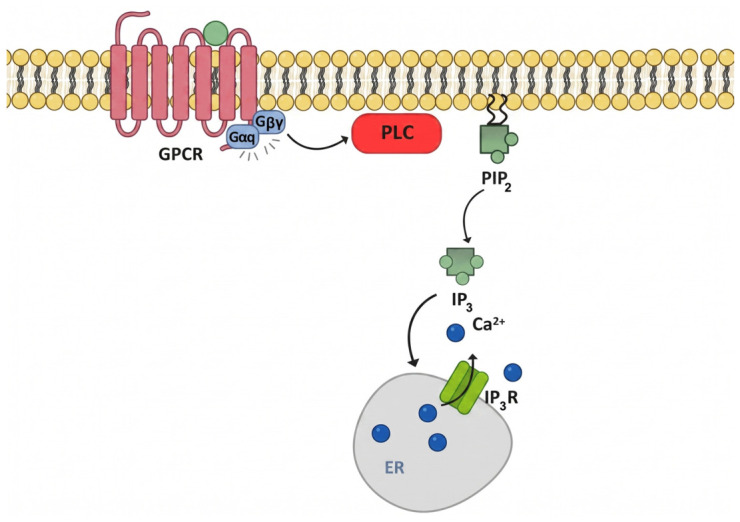
Schematic representation of a GPCR-mediated calcium signaling pathway involving phospholipase C (PLC) activation and IP3-dependent Ca^2+^ release from the endoplasmic reticulum (ER).

H_4_R is primarily coupled with *G*i/o proteins. Calcium (Ca^2+^) mobilization occurs downstream of *G*_βγ_ subunit activation, which stimulates phospholipase C (PLC). PLC hydrolyses phosphatidylinositol-4,5-bisphosphate (PIP_2_) to generate inositol 1,4,5-trisphosphate (IP_3_). IP_3_ binds to IP_3_ receptors on the endoplasmic reticulum (ER), inducing Ca^2+^ release from intracellular stores; in some systems, this is followed by an additional influx of Ca^2+^ from the extracellular space (Figure 9).

The resulting Ca^2+^ response is rapid, transient, and dependent on the concentration of the H_4_R agonist **4-MH** [7,55,56,57].

In CHO cells stably expressing human H_4_R (CHO-H_4_R), stimulation with **4-MH** (10 µM) produced a marked increase in cytosolic Ca^2+^ concentration. Previous studies have shown that this H_4_R-dependent signal is abolished by the selective H_4_R antagonist **JNJ-7777120** [58,59], confirming that Ca^2+^ mobilization in this model is specifically mediated by H_4_R activation. Therefore, Ca^2+^ mobilization provides a useful functional readout for studying histamine H_4_R ligands.

**Figure 10:**
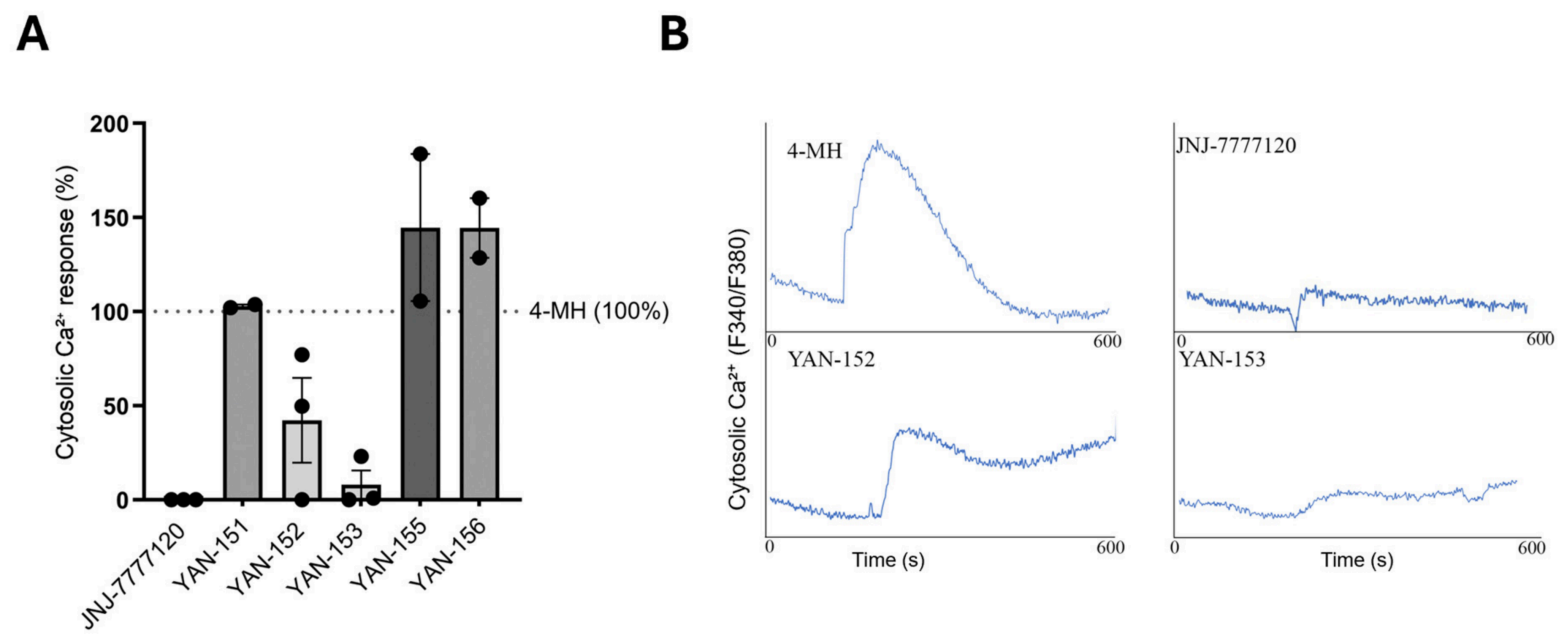
H_4_R-mediated Ca^2+^ mobilization in CHO-H_4_R cells. (**A**) Quantification of peak Ca^2+^ responses expressed as percentage of the paired **4-MH**-evoked response measured in the same experiment (**4-MH** response set to 100%, indicated by the dashed reference line). Bars represent mean ± SEM of individual experiments (*n* = 2–3), with individual data points overlaid; **JNJ-7777120** served as the H_4_R antagonist control. (**B**) Representative traces of cytosolic Ca^2+^ responses induced by 10 µM **4-MH** in the absence or presence of 10 µM **JNJ-7777120**, **YAN-152** or **YAN-153**, shown as Fura-2 fluorescence ratio over time.

As shown in Figure 10A, stimulation of CHO-H_4_R cells with 10 µM **4-MH** induced a rapid, transient increase in cytosolic calcium concentration, characteristic of H_4_R-mediated mobilization from intracellular stores. Pre-incubation with the selective H_4_R antagonist **JNJ-7777120** (10 µM) almost completely abolished the Ca^2+^ signal, confirming that the response is H_4_R-dependent. When applied alone (without **4-MH**), none of the tested compounds evoked a detectable Ca^2+^ response, indicating that none behaved as an H_4_R agonist in this assay; their effects were therefore assessed as modulation of the **4-MH**-evoked response, each normalized to its paired **4-MH** control (set to 100%; Figure 10A).

Quantification of peak responses (Figure 10A) revealed that **YAN-153** markedly lowered the signal, to approximately 8% of the paired **4-MH** control, whereas **YAN-152** produced a weaker and more variable inhibition (to approximately 42% on average). Thus, both new compounds inhibited H_4_R-mediated Ca^2+^ mobilization, with **YAN-153** showing the stronger and more consistent effect in this pathway. **YAN-151** did not appreciably alter the response, behaving as functionally silent. In contrast, **YAN-155** and **YAN-156** enhanced the **4-MH**-evoked Ca^2+^ response, an unexpected effect within the same chemotype that warrants further mechanistic investigation.

## 3. Materials and Methods

Reagents, solvents, and other materials were of commercial origin and used without additional operations. Reactions were monitored on silica gel TLC plates 60 F_254_ (Merck, Darmstadt, Germany). Visualizations were performed with UV light (254 and/or 365 nm) and then with CeMo stain and subsequent charring. The melting points were determined using the MP70 Melting Point System (Mettler-Toledo, Greifensee, Switzerland). Solvents were evaporated under reduced pressure at 40 °C on the Büchi Rotavapor (BÜCHI Labortechnik AG, Flawil, Switzerland). Flash column chromatography was performed on silica gel (200–300 mesh). The chromatographic analysis (HPLC) was performed using a Waters HPLC system (Waters Assoc., Milford, MA, USA) consisting of two Waters pump 515, Waters autosampler 717-plus, Waters column oven and Photodiode Array Detector 2996. Chromatographic parameters: stationary phase—Kromasil C-8 (250 × 4.6 mm, 5 µm), mobile phase–A (0.1% TFA in acetonitrile) and B (0.1% TFA in water) 50/50, elution-isocratic—1 mL/min, duration—15 min., column temp.—25 °C, injection volume—3 µL, UV detection wavelength—ʎ = 278 nm. The ^1^H and ^13^C NMR spectra were acquired in CDCl_3_, DMSO-*d*6, CD_3_OD and D_2_O solutions on Bruker AVANCE III HD 500 MHz spectrometer (Bruker Corporation, Billerica, MA, USA) at the temperature of 298 K. To identify the structures of all isolated products, analysis of the results of 1D and 2D NMR experiments was performed. The ^1^H and ^13^C NMR chemical shifts are given relative to the TMS signal at δ = 0.0 ppm. Mass spectra were recorded on the MaldiSYNAPT G2-S HDMS (Waters Corporation, Milford, MA, USA) spectrometer via electrospray ionization (ESI–MS). High-resolution mass spectrometry (HRMS) measurements were performed using the Synapt G2-Si mass spectrometer (Waters Corporation, Milford, MA, USA) equipped with an ESI source and a quadrupole-time-of-flight mass analyzer. The results of the measurements were processed using the MassLynx 4.1 software (Waters Corporation, Milford, MA, USA).

### 3.1. Virtual Screening

#### 3.1.1. Preparing Ligand and Receptor Structures

The structure of reference compound **JNJ-7777120** was taken from PubChem [60] while the 3D structures of test compounds were constructed in LigPrep module of Schrödinger suite of programs (v. 2024-2, Schrödinger LLC, Portland, OR, USA). LigPrep works by expanding tautomeric and ionization states, ring conformations, and stereoisomers. The representative structure for each test compound was set assuming neutral pH. The full structure of inactive human H_4_ receptor (UniProt Q9H3N8) was obtained from AlphaFold Protein Structure Database [61] since there is no structure of inactive H_4_R in PDB. The structure is nearly identical (Calpha RMSD of 1.354 Å over 264 aligned residues with 50.76% sequence identity) to crystal structure of human H_3_R in complex with antagonist PF03654746 [62]. The full structure of human H_4_R has 390 residues; however, for the purpose of molecular ligand docking some cytoplasmic parts of the receptor located far from the ligand binding site were removed: the long cytoplasmic loop between helices TM5 and TM6 (residues 218–286) and the C-terminus beyond helix H8 (residues 381–390). The removed parts had also a low and very low confidence values (pLDDT—the predicted local distance difference test) in AlphaFold database. The N-terminus (residues 1–10) was kept as it would be important for ligand binding.

#### 3.1.2. Molecular Docking and Molecular Dynamics

The ligand docking was performed in Glide module (Schrödinger suite of programs) in XP (extra precision) mode and then IFD-MD (Induced Fit Docking—Molecular Dynamics) (Schrödinger suite of programs) was used. IFD-MD performs local search of the best ligand pose using the MD procedure and additionally (i) incorporates the effects that water molecules have on binding as an important component of the IFD-MD scoring function, and (ii) detects and penalizes desolvation of polar groups caused by non-native ligand poses. To check whether the ligands were stably docked to the receptor, the 20 ns classical MD simulations were performed in the explicit membrane-water environment. The receptor with appropriate ligands was immersed in a hydrated POPC bilayer (enriched by 20% (n/n) cholesterol), and then MD simulations were performed in AMBER24 using CHARMM36m force field [63]. All systems were generated in CHARMM-GUI service v. 3.8 (https://www.charmm-gui.org) [64] and the receptor orientation in the membrane was set according to the OPM database [65]. The standard system contained ~70,000 atoms, ~127 lipid molecules (cholesterol + POPC), and concentration of ions Na^+^ and Cl^−^ was 0.15 M. The average dimensions of a periodic cell were 74 Å × 74 Å × 140 Å. TIP3P water model parametrized to use with CHARMM force fields was employed. Before each MD simulation the system was submitted to a restrained energy minimization (5000 cycles). The first 2500 minimization cycles were performed with a steepest-descent method and after that the conjugate gradient was switched on. Next, a six-step equilibration was performed (375 ps total) at a constant pressure and temperature (NPT ensemble; 310 K, 1 bar). During equilibration the restraints were released gradually, until the last step (last 100 ps of equilibration), in which no restraints were used. In the production simulations (as well as in the last three equilibration steps) all bond lengths to hydrogen atoms were constrained using SHAKE algorithm (version in AMBER24 program) [66], which allowed us to use a longer time step (2 fs instead of 1 fs). Van der Waals and short-range electrostatic interactions cutoff = 12 Å, with 10–12 Å switching function, were used. Long-range electrostatic interactions were computed using the Particle Mesh Ewald [67] summation scheme.

#### 3.1.3. Predictions of Free Energy of Ligand Binding

The calculations of free energy ligand binding of the poses obtained from IFD procedure were performed in Prime module of Schrödinger package using MM-GBSA (Molecular Mechanics/Generalized Born Surface Area) method. The following standard Prime energy components were used: Coulomb energy, Covalent binding energy, Van der Waals energy, Lipophilic energy, Generalized Born electrostatic solvation energy, Hydrogen-bonding correction, π–π packing correction, and Self-contact correction. The newer VSGB 2.1 (Variable Solvent Generalized Born) solvation model was used, which is a refit of VSGB 2.0 [68], and calculated in OPLS3 force field [69] (for VSGB 2.0 the calculations were done in OPLS_2005 force field).

#### 3.1.4. The Ballesteros–Weinstein Numbering Scheme

For direct comparison of ligand–protein interactions between different GPCRs we use the Ballesteros–Weinstein numbering scheme of residues. In this numbering scheme each GPCR residue is marked with two numbers separated by a dot. The first number indicates the transmembrane helix while the second number the position of the residue relative to the most conserved residue (assigned the number 50) on the same helix in a sequential order [70].

### 3.2. Chemical Synthesis

#### 3.2.1. Pyrido[2,3-*d*]pyrimidine-2,4(1H,3H)-dione **1**

Compound **1** was obtained by melting a mixture of 2-aminonicotinic acid and urea and then heating 2 h at 200 °C [50]. The product was used in the next step without further purification. Yield 97%.

#### 3.2.2. 2,4-Dichloropyrido[2,3-*d*]pyrimidine **2**

Compound **2** was synthesized by heating **1** with POCl_3_ in DMF according to a well-established literature procedure [51]. Yield 89%.

#### 3.2.3. Synthesis of **YAN-151**–**YAN-155**—General Procedure

2-Chloro-4-(phenylmethylamino)pyrido[2,3-*d*]pyrimidine **3a**

A total of 50 mg (0.25 mmol, 1 eq.) of **2** was dissolved in 5 mL of THF. To this solution 70 µL (0.50 mmol, 2 eq) of TEA and then 27 µL (0.25 mmol, 1 eq) of benzylamine **a**-NH_2_ were added. After a while a white solid precipitated. This suspension was stirred at room temperature overnight. Reaction completion was confirmed by TLC (DCM/MeOH 95:5 *v*/*v*). Post-reaction mixture was diluted with 15 mL of water, the product was extracted with DCM (2 × 15 mL), the organic layer was dried over magnesium sulfate and concentrated under vacuum. The crude mixture was purified on silica gel (DCM/MeOH 98:2 *v*/*v*) giving 61 mg (0.23 mmol) of the pure product. Yield: 90%; white solid, **MP**: 178 °C (dec); **^1^H NMR** (25 °C, MeOH-*d*_1_): 8.92 (bs, 1H, H7_Pyridine_), 8.61 (dd, 1H, H5_Pyridine_), 7.52 (dd, 1H, H6_Pyridine_), 7.41 (m, 2H, H2_Ph_ and H6_Ph_), 7.33 (m, 2H, H3_Ph_ and H5_Ph_), 7.26 (m, 1H, H4_Ph_), 4.88 (s, 2H, NHCH_2_Ph); **^13^C NMR** (25 °C, MeOH-*d*_1_): 163.50 (C2_Pyrimidine_), 160.53 (C8a_Pyridine_), 160.25 (C4_Pyrimidine_) 157.18 (C7_Pyridine_), 139.21 (C1_Ph_), 134.27 (C5_Pyridine_), 129.62 (C3_Ph_/C5_Ph_), 129.07 (C2_Ph_/C6_Ph_), 128.53 (C4_Ph_), 123.03 (C6_Pyridine_), 46.13 (CH_2_); **HRMS**: calcd for C_14_H_12_ClN_4_: 271.0750 found: 271.0753 [M + H]^+^.

The following compounds were obtained according to the same synthetic procedure.

2-Chloro-4-(2-thiopheneethanamino)pyrido[2,3-*d*]pyrimidine **3b**

The compound **3b** was synthesized using 200 mg (1.00 mmol, 1 eq.) of (**2**), 114 µL (1.00 mmol, 1 eq) of 2-thiopheneethanamine **b**-NH_2_ and 278 µL (2.00 mmol, 2 eq.) of TEA. After purification on silica gel 256 mg (0.88 mmol) of the pure product was obtained. Yield: 88%; white solid, **MP**: 155 °C (dec); **^1^H NMR** (25 °C, CDCl_3_): 8.91 (bs, 1H, H7_Pyridine_), 8.40 (d, 1H, H5_Pyridine_), 7.39 (bs, 1H, H6_Pyridine_), 7.14 (dd, 1H, H5_Thiophene_), 6.92 (dd, 1H, H4_Thiophene_), 6.86 (d, 1H, H3_Thiophene_), 3.91 (t, 2H, NHCH_2_CH_2_), 3.23 (t, 2H, NHCH_2_CH_2_); **^13^C NMR** (25 °C, CDCl_3_): 161.46 (C4_Pyrimidine_), 161.29 (C2_Pyrimidine_), 158.15 (C8a_Pyridine_), 155.36 (C7_Pyridine_), 140.86 (C2_Thiophene_), 132.92 (C5_Pyridine_), 127.06 (C4_Thiophene_), 125.55 (C3_Thiophene_), 124.04 (C5_Thiophene_), 121.49 (C6_Pyridine_), 108.68 (C4a_Pyridine_), 43.05 (NHCH_2_CH_2_), 28.80 (NHCH_2_CH_2_); **HRMS**: calcd for C_13_H_12_ClN_4_S: 291.0471 found: 291.0475 [M + H]^+^.

2-Chloro-4-(3,4-dimethoxybenzeneethanamino)pyrido[2,3-*d*]pyrimidine **3c**

This compound was synthesized using 100 mg (0.50 mmol, 1 eq.) of **2**, 84 µL (0.50 mmol, 1 eq) of 3,4-dimethoxybenzeneethanamine **c**-NH_2_ and 139 µL (1.00 mmol, 2 eq.) of TEA. After purification on silica gel 67 mg (0.20 mmol) of the pure product was obtained. Yield: 40%; white solid, **MP**: 170 °C (dec); **^1^H NMR** (25 °C, CDCl_3_): 8.77 (dd, 1H, H7_Pyridine_), 8.26 (dd, 1H, H5_Pyridine_), 7.26 (dd, 1H, H6_Pyridine_), 6.70 (m, 1H, H2_Ph_), 6.69 (m, 1H, H5_Ph_), 6.68 (m, 1H, H6_Ph_), 3.75 (m, 2H, NHCH_2_CH_2_), 3.73 (s, 6H, both CH_3_), 2.85 (t, 2H, NHCH_2_CH_2_); **^13^C NMR** (25 °C, CDCl_3_): 161.77 (C4_Pyrimidine_), 161.26 (C2_Pyrimidine_), 158.80 (C8a_Pyridine_), 155.68 (C7_Pyridine_), 148.77 (C3_Ph_), 147.47 (C4_Ph_), 132.32 (C5_Pyridine_), 131.36 (C1_Ph_), 121.33 (C6_Pyridine_), 120.74 (C6_Ph_), 112.00 (C2_Ph_), 111.25 (C5_Ph_), 108.71 (C4a_Pyridine_), 55.76 (both CH_3_), 43.08 (NHCH_2_CH_2_), 34.21 (NHCH_2_CH_2_); **HRMS**: calcd for C_17_H_18_ClN_4_O_2_: 345.1118 found: 345.1121 [M + H]^+^.

2-(4-Methyl-1-piperazinyl)-4-(phenylmethylamino)pyrido[2,3-*d*]pyrimidine **YAN-151**

A total of 50 mg (0.19 mmol, 1 eq.) of **3a** was suspended in 5 mL of EtOH and 41 µL (0.37 mmol, 2 eq) of *N*-methylpiperazine **a′**-NH_2_ was added. The mixture was heated for 4 h under reflux. The reaction progress was monitored by TLC (AcOEt/MeOH/TEA 90:5:5 *v*/*v*/*v*) until traces of starting materials were not observed. Post-reaction mixture was condensed under vacuum resulting in the crude product which was purified on silica gel (DCM/MeOH 95:5 → 90:10 *v*/*v*) obtaining 50 mg (0.15 mmol) of the pure product. Yield: 79%; **Purity**: 99.67% (HPLC); **MP**: 180 °C (dec); **^1^H NMR** (25 °C, DMSO-*d*_6_): 8.66 (d, 1H, H7_Pyridine_), 8.77 (d, 1H, H5_Pyridine_), 7.35 (m, 2H, H2_Ph_ and H6_Ph_), 7.26 (m, 1H, H4_Ph_), 7.21 (m, 2H, H3_Ph_ and H5_Ph_), 6.93 (dd, 1H, H6_Pyridine_), 6.75 (bs, 1H, NH), 4.76 (d, 2H, NHCH_2_), 4.00 (bs, 4H, CH_2Piperazine_ at C2/C6), 2.50 (m, 4H, CH_2Piperazine_ at C3/C5), 2.35 (s, 3H, CH_3_); **^13^C NMR** (25 °C, DMSO-*d*_6_): 160.87 (C8a_Pyridine_), 160.39 (C2_Pyrimidine_), 160.36 (C4_Pyrimidine_), 155.07 (C7_Pyridine_), 138.41 (C1_Ph_), 131.06 (C5_Pyridine_), 128.60 (C3_Ph_ and C5_Ph_), 127.75 (C2_Ph_ and C6_Ph_), 127.45 (C4_Ph_), 116.37 (C6_Pyridine_), 105.13 (C4_Pyridine_), 54.56 (C3_Piperazine_ and C5_Piperazine_), 45.56 (CH_3_), 45.32 (NHCH_2_), 43.16 (C2_Piperazine_ and C6_Piperazine_); **HRMS**: calcd for C_19_H_23_N_6_: 335.1984 found: 335.1985 [M + H]^+^.

The following compounds were obtained according to the same synthetic procedure.

2-(4-Methyl-1-piperazinyl)-4-(2-thiopheneethanamino)pyrido[2,3-*d*]pyrimidine **YAN-152**

The compound **YAN-152** was synthesized using 80 mg (0.28 mmol, 1 eq.) of **3b** and 61 µL (0,55 mmol, 2 eq.) of *N*-methylpiperazine **a′**-NH_2_. After purification on silica gel 90 mg (0.25 mmol) of the pure product was obtained. Yield: 89%; **Purity**: 98.35% (HPLC); **MP**: decomposition at 180 °C; **^1^H NMR** (25 °C, DMSO-*d*_6_): 8.62 (dd, 1H, H7_Pyridine_), 8.27 (dd, 1H, H5_Pyridine_), 7.22 (dd, 1H, H5_Thiophene_), 7.09 (dd, 1H, H6_Pyridine_), 6.94 (dd, 1H, H4_Thiophene_), 6.88 (d, 1H, H3_Thiophene_), 4.01 (bs, 4H, CH_2Piperazine_ at C2/C6), 3.82 (t, 2H, NHCH_2_CH_2_), 3.24 (t, 2H, NHCH_2_CH_2_), 2.55 (t, 4H, CH_2Piperazine_ at C3/C5), 2.37 (s, 3H, CH_3_); **^13^C NMR** (25 °C, DMSO-*d*_6_): 162.17 (C2_Pyridine_ + C4_Pyridine_), 161.95 (C8a_Pyridine_), 155.47 (C7_Pyridine_), 142.83 (C2_Thiophene_), 133.64 (C5_Pyridine_), 127.87 (C4_Thiophene_), 126.26 (C3_Thiophene_), 124.72 (C5_Thiophene_), 117.79 (C6_Pyridine_), 107.12 (C4a_Pyridine_), 55.91 (CH_2Piperazine_ at C3/C5), 46.14 (CH_3_), 44.47 (CH_2Piperazine_ at C2/C6), 44.20 (NHCH_2_CH_2_), 29.96 (NHCH_2_CH_2_); **HRMS**: calcd for C_18_H_23_N_6_S: 355.1705 found: 355.1708 [M + H]^+^.

2-(4-(piperidin-4-yl)morpholine)-4-(2-thiopheneethanamino)pyrido[2,3-*d*]pyrimidine **YAN-153**

The compound **YAN-153** was synthesized using 80 mg (0.28 mmol, 1 eq.) of **3b** and 94 mg (0,55 mmol, 2 eq.) of 4-(piperidin-4-yl)morpholine **b′**-NH_2_. After purification on silica gel 115 mg (0.27 mmol) of the pure product was obtained. Yield: 98%; **Purity**: 97.87% (HPLC); **MP**: decomposition at 98.9 °C; **^1^H NMR** (25 °C, DMSO-*d*_6_): 8.68 (dd, 1H, H7_Pyridine_), 7.85 (dd, 1H, H5_Pyridine_), 7.18 (dd, 1H, H5_Thiophene_), 6.96 (dd, 1H, H4_Thiophene_), 6.92 (dd, 1H, H6_Pyridine_), 6.85 (d, 1H, H3_Thiophene_), 6.18 (t, 1H, NHCH_2_), 5.07 (d, 2H, two of CH_2Piperidine_ at C2/C6), 3.85 (q, 2H, NHCH_2_), 3.73 (t, 4H, CH_2Morpholine_ at C3/C5), 3.22 (t, 2H, NHCH_2_CH_2_), 2.91 (t, 2H, two of CH_2Piperidine_ at C2/C6), 2.59 (t, 4H, CH_2Morpholine_ at C2/C6), 2.50 (m, 1H, CH_Piperidine_ at C4), 1.93 (d, 2H, two of CH_2Piperidine_ at C3/C5), 1.50 (d, 2H, two of CH_2Piperidine_ at C3/C5); **^13^C NMR** (25 °C, DMSO-*d*_6_): 161.03 (C8a_Pyridine_), 160.33 (C2_Pyrimidine_), 160.22 (C4_Pyrimidine_), 155.14 (C7_Pyridine_), 141.31 (C2_Thiophene_), 130.46 (C5_Pyridine_), 127.11 (C4_Thiophene_), 125.38 (C3_Thiophene_), 124.04 (C5_Thiophene_), 116.10 (C6_Pyridine_), 104.84 (C4a_Pyridine_), 67.10 (CH_2Morpholine_ at C3/C5), 62.64 (CH_Piperidine_ at C4), 49.68 (CH_2Morpholine_ at C2/C6), 43.29 (CH_2Piperidine_ at C2/C6), 42.78 (NHCH_2_), 29.19 (NHCH_2_CH_2_), 28.15 (CH_2Piperidine_ at C3/C5); **HRMS**: calcd for C_22_H_29_N_6_OS: 425.2124 found: 425.2119 [M + H]^+^.

2-(4-Methyl-1-piperazinyl)-4-(3,4-dimethoxybenzeneethanamino)pyrido[2,3-*d*]pyrimidine **YAN-154**

The compound **YAN-154** was synthesized using 80 mg (0.23 mmol, 1 eq.) of **3c** and 52 µL (0.464 mmol, 2 eq.) of *N*-methylpiperazine **a′**-NH_2_. After purification on silica gel 93 mg (0.22 mmol) of the pure product was obtained. Yield: 96%; **Purity**: 99.22% (HPLC); **MP**: slow decomposition from 80 °C; **^1^H NMR** (25 °C, DMSO-*d*_6_): 8.69 (dd, 1H, H7_Pyridine_), 7.73 (dd, 1H, H5_Pyridine_), 6.82 (dd, 1H, H6_Pyridine_), 6.82 (d, 1H, H5_Ph_), 6.76 (dd, 1H, H6_Ph_), 6.71 (d, 1H, H2_Ph_), 5.83 (t, 1H, NHCH_2_CH_2_), 4.06 (bs, 4H, CH_2Piperazine_ at C2 and C6), 3.87 (s, 3H, OCH_3_ at C4_Ph_), 3.82 (m, 2H, NHCH_2_CH_2_), 3.81 (s, 3H, OCH_3_ at C3_Ph_), 2.94 (t, 2H, NHCH_2_CH_2_), 2.53 (t, 4H, CH_2Piperazine_ at C3 and C5), 2.37 (s, 3H, CH_3_); **^13^C NMR** (25 °C, DMSO-*d*_6_): 161.04 (C8a_Pyridine_), 160.57 (C2_Pyrimidine_), 160.26 (C4_Pyrimidine_), 155.34 (C7_Pyridine_), 149.04 (C3_Ph_), 147.74 (C4_Ph_), 131.30 (C1_Ph_), 130.08 (C5_Pyridine_), 120.63 (C6_Ph_), 116.23 (C6_Pyridine_), 111.87 (C2_Ph_), 111.36 (C5_Ph_), 104.84 (C4a_Pyrimidine_), 55.89 (OCH_3_ at C4_Ph_), 55.78 (OCH_3_ at C3_Ph_), 54.97 (C3_Piperazine_ and C5_Piperazine_), 46.00 (CH_3_), 43.51 (C2_Piperazine_ and C6_Piperazine_), 42.57 (NHCH_2_CH_2_), 34.59 (NHCH_2_CH_2_); **HRMS**: calcd for C_22_H_29_N_6_O_2_: 409.2352 found: 409.2354 [M + H]^+^.

2-(2,6-Dimethylpiperazinyl)-4-(3,4-dimethoxybenzeneethanamino)pyrido[2,3-*d*]pyrimidine **YAN-155**

This compound was synthesized using 80 mg (0.23 mmol, 1 eq.) of **3c** and 53 mg (0.46 mmol, 2 eq.) of 2,6-dimethylpiperazine **c′**-NH_2_. After purification on silica gel 93 mg (0.22 mmol) of the pure product was obtained. Yield: 96%; **Purity**: 99.74% (HPLC); **MP**: 165 °C (dec); **^1^H NMR** (25 °C, DMSO-*d*_6_): 8.70 (dd, 1H, H7_Pyridine_), 7.68 (dd, 1H, H5_Pyridine_), 6.91 (dd, 1H, H6_Pyridine_), 6.83 (d, 1H, H5_Ph_), 6.77 (dd, 1H, H6_Ph_), 6.71 (d, 1H, H2_Ph_), 4.97 (bs, 2H, two of CH_2Piperazine_ at C2 and C6), 3.87 (s, 3H, OCH_3_ at C4_Ph_), 3.83 (m, 2H, NHCH_2_CH_2_), 3.81 (s, 3H, OCH_3_ at C3_Ph_), 2.94 (m, 2H, CH_2Piperazine_ at C3 and C5), 2.94 (m, 2H, NHCH_2_CH_2_), 2.56 (t, 2H, two of CH_2Piperazine_ at C3 and C5), 1.18 (s, 6H, CH_3_); **^13^C NMR** (25 °C, DMSO-*d*_6_): 161.14 (C8a_Pyridine_), 160.38 (C2_Pyrimidine_), 160.23 (C4_Pyrimidine_), 155.42 (C7_Pyridine_), 149.06 (C3_Ph_), 147.77 (C4_Ph_), 131.31 (C1_Ph_), 129.93 (C5_Pyridine_), 120.65 (C6_Ph_), 116.11 (C6_Pyridine_), 111.89 (C2_Ph_), 111.37 (C5_Ph_), 104.75 (C4a_Pyrimidine_), 55.90 (OCH_3_ at C4_Ph_), 55.80 (OCH_3_ at C3_Ph_), 50.96 (C3_Piperazine_ and C5_Piperazine_), 50.13 (both CH_2Piperazine_), 42.56 (NHCH_2_CH_2_), 34.58 (NHCH_2_CH_2_), 19.27 (CH_3_); HRMS: calcd for C_23_H_31_N_6_O_2_: 423.2508 found: 423.2501 [M + H]^+^.

4-Oxo-4-(4-pyridinylamino)butanoic Acid **5a**

4-Aminopyridine **4a** (1.88 g, 20 mmol, 1.0 eq.) was dissolved in 40 mL of THF, forming a clear, colorless solution (A). Succinic anhydride (2.20 g, 22 mmol, 1.1 eq.) was dissolved in 20 mL of THF separately, forming a clear, colorless solution (B). The solution B was added to the solution A, while vigorously stirring at room temperature. The mixture immediately got cloudy and the white solid started to precipitate within 5 min. The resulting suspension was concentrated to approx. ⅓ of its initial volume. Precipitated solid was filtered, washed with a small volume of cold THF and dried in vacuo at 40 °C. A total of 1.78 g (9.2 mmol) of a pure product was obtained. Yield: 46%; **MP**: 191 °C; **^1^H NMR** (25 °C, DMSO-d_6_): 9.66 (bs, 1H, CO_2_H), 8.00 (d, 2H, H2_Pyridine_ and H6_Pyridine_), 6.75 (s, 1H, NH), 6.57 (d, 2H, H3_Pyridine_ and H5_Pyridine_), 2.34 (s, 4H, both CH_2_); **^13^C NMR** (25 °C, DMSO-d_6_): 174.51 (CO_2_H), 174.45 (NHC(=O)), 156.46 (C4_Pyridine_), 145.62 (C2_Pyridine_ and C6_Pyridine_), 108.80 (C3_Pyridine_ and C5_Pyridine_), 30.41 (both CH_2_); **HRMS**: calcd for C_9_H_11_N_2_O_3_: 195.0770 found: 195.0772 [M + H]^+^, calcd for C_9_H_9_N_2_O_3_: 193.0613 found: 193.0612 [M + H]^–^.

The following compounds were obtained according to the same synthetic protocol (reaction temperature and time are given separately in each case).

4-[(5-Chloro-2-pyridinyl)amino]-4-oxobutanoic Acid **5b**

The compound **5b** was synthesized using 2-amino-5-chloropyridine **4b** (2.57 g, 20 mmol, 1.0 eq.) in 20 mL of THF and 2.60 g (26 mmol, 1.3 eq.) of succinic anhydride in 25 mL of THF under reflux overnight. After workup and purification as described for **5a** 3.79 g (17 mmol) of the pure product was obtained. Yield: 85%. **MP**: 197 °C; **^1^H NMR** (25 °C, DMSO-*d*_6_): 12.11 (s, 1H, CO_2_H), 10.67 (s, 1H, NH), 8.33 (d, 1H, H6_Pyridine_), 8.08 (d, 1H, H3_Pyridine_), 7.86 (dd, 1H H4_Pyridine_), 2.62 (m, 2H, CH_2_CH_2_CO_2_H), 2.50 (m, 2H, CH_2_CH_2_CO_2_H); **^13^C NMR** (25 °C, DMSO-*d*_6_): 173.73 (CO_2_H), 171.20 (NHC(=O)), 150.71 (C2_Pyridine_), 146.24 (C6_Pyridine_), 137.81 (C4_Pyridine_), 124.74 (C5_Pyridine_), 114.39 (C3_Pyridine_), 30.85 (CH_2_CH_2_CO_2_H), 28.47 (CH_2_CH_2_CO_2_H); **HRMS**: calcd for C_9_H_10_ClN_2_O_3_: 229.0380 found: 229.0384 [M + H]^+^.

4-Oxo-4-(3-pyridinylamino)butanoic Acid **5c**

This compound was synthesized using 3-aminopyridine **4c** (1.88 g, 20 mmol, 1.0 eq.) in 10 mL of THF and 2.20 g (22 mmol, 1.1 eq.) of succinic anhydride in 20 mL of THF at room temperature for 1 h. After workup and purification as described for **5a** 3.61 g (19 mmol) of the pure product was obtained. Yield: 93%. **MP**: 187 °C; **^1^H NMR** (25 °C, DMSO-*d*_6_): 12.16 (s, 1H, CO_2_H), 10.16 (s, 1H, NH), 8.71 (d, 1H, H2_Pyridine_), 8.22 (dd, 1H, H6_Pyridine_), 8.01 (d, 1H, H4_Pyridine_), 7.31 (dd, 1H, H5_Pyridine_), 2.58 (m, 2H, CH_2_CH_2_CO_2_H), 2.53 (m, 2H, CH_2_CH_2_CO_2_H); **^13^C NMR** (25 °C, DMSO-*d*_6_): 174.22 (CO_2_H), 171.18 (NHC(=O)), 144.35 (C6_Pyridine_), 140.98 (C2_Pyridine_), 136.35 (C3_Pyridine_), 126.23 (C4_Pyridine_), 124.06 (C5_Pyridine_), 31.37 (CH_2_CH_2_CO_2_H), 29.12 (CH_2_CH_2_CO_2_H); **HRMS**: calcd for C_9_H_11_N_2_O_3_: 195.0770 found: 195.0772 [M + H]^+^.

4-[(2-Chloro-3-pyridinyl)amino]-4-oxobutanoic Acid **5d**

This compound was synthesized using 3-amino-2-chloropyridine **4d** (2.57 g, 20 mmol, 1.0 eq.) in 25 mL of THF and 2.20 g (22 mmol, 1.1 eq.) of succinic anhydride in 20 mL of THF under reflux for 5 h. After workup and purification as described for **5a** 2.41 g (11 mmol) of the pure product was obtained. Yield: 53%. **MP**: 142 °C; **^1^H NMR** (25 °C, DMSO-*d*_6_): 12.14 (s, 1H, CO_2_H), 9.69 (s, 1H, NH), 8.16 (m, 1H, H4_Pyridine_), 8.16 (m, 1H, H6_Pyridine_), 7.41 (dd, 1H, H5_Pyridine_), 2.66 (m, 2H, CH_2_CH_2_CO_2_H), 2.51 (m, 2H, CH_2_CH_2_CO_2_H); **^13^C NMR** (25 °C, DMSO-*d*_6_): 173.68 (CO_2_H), 171.07 (NHC(=O)), 145.06 (C6_Pyridine_), 142.77 (C2_Pyridine_), 133.73 (C4_Pyridine_), 132.02 (C3_Pyridine_), 123.31 (C5_Pyridine_), 30.64 (CH_2_CH_2_CO_2_H), 28.76 (CH_2_CH_2_CO_2_H); **HRMS**: calcd for C_9_H_10_ClN_2_O_3_: 229.0380 found: 229.0382 [M + H]^+^.

t-butyl 4-[4-(4-pyridinylamino)-1,4-dioxobutyl]-1-piperazine carboxylate **6a**

EDCI × HCl (0.96 g, 5 mmol, 1.0 eq.) was suspended in 30 mL of DMF. To this suspension 1.0 mL (5 mmol, 1.0 eq.) of the DIPEA was added resulting in the slow dissolution of the initial suspension and formation of a clear solution. To this mixture were added: 0.97 g (5 mmol, 1.0 eq.) of the 4-oxo-4-(4-pyridinylamino)butanoic acid **5a** and after complete dissolving of **5a**, 0.88 g (4.7 mmol, 0.94 eq.) of the *N*-Boc-piperazine. The resulting mixture was stirred at room temperature overnight and then the solvent was removed under vacuum. The residue was treated with 25 mL of water and extracted with DCM (3 × 20 mL). Collected extracts were dried over magnesium sulphate and condensed to give a crude product, which was purified on silica gel (chloroform/methanol 98:2 → 95:5), resulting in 0.84 g (2.3 mmol) of the **6a**. Yield: 46%. **^1^H NMR** (25 °C, DMSO-*d*_6_): 9.38 (s, 1H, NH), 8.39 (d, 2H, H2_Pyridine_ and H6_Pyridine_), 7.42 (d, 2H, H3_Pyridine_ and H5_Pyridine_), 3.60 (m, 2H, H6_Pipreazine_ (or H2_Piperazine_), 3.48 (m, 4H, H2_Piperazine_ and H3_Piperazine_ (or H6_Piperazine_ and H5_Piperazine_)), 3.42 (m, 2H H5_Piperazine_ (or H3_Piperazine_)), 2.75 (s, 4H, both CH_2_), 1.46 (s, 9H, CH_3_); **^13^C NMR** (25 °C, DMSO-*d*_6_): 171.58 (NHC(=O)), 170.67 (C(=O)Pip), 154.42 (C(=O)O*t*-Bu), 150.26 (C2_Pyridine_ and C6_Pyridine_), 145.37 (C4_Pyridine_), 113.42 (C3_Pyridine_ and C5_Pyridine_), 80.48 (C(CH_3_)_3_), 45.17 (C2_Piperazine_ and C3_Piperazine_ (or C6_Piperazine_ and C5_Piperazine_)), 42.83 (C5_Piperazine_ (or C3_Piperazine_)), 41.72 (C6_Piperazine_ (or C2_Piperazine_)), 32.42 NH(C(=O)CH_2_CH_2_), 28.48 (NH(C(=O)CH_2_CH_2_), 28.30 (CH_3_); **HRMS**: calcd for C_18_H_27_N_4_O_4_: 363.2032 found [M + H]^+^.

The following compounds were obtained in the same manner.

t-Butyl 4-[4-(5-chloro-2-pyridinylamino)-1,4-dioxobutyl]-1-piperazine carboxylate **6b**

The compound **6b** was synthesized using **5b** (1.14 g, 5 mmol, 1.0 eq.), 0.96 g (5 mmol, 1.0 eq.) of EDCI × HCl, 1.0 mL (5 mmol, 1.0 eq.) of DIPEA and 0.86 g (4.7 mmol, 0.94 eq.) of *N*-Boc-piperazine in 30 mL of DMF. After workup and purification as described for **6a** 1.41 g (3.6 mmol) of the product was obtained. Yield: 72%. **MP**: 196 °C; **^1^H NMR** (25 °C, CDCl_3_): 9.07 (s, 1H, NH), 8.19 (d, 1H, H6_Pyridine_), 8.16 (d, 1H, H3_Pyridine_), 7.62 (dd, 1H, H4_Pyridine_), 3.62 (m, 2H, H2_Piperazine_ (or H6_Piperazine_)), 3.47 (bs, 4H, H5_Piperazine_ and H6_Piperazine_ (or H3_Piperazine_ and H2_Piperazine_)), 3.40 (m, 2H, H3_Piperazine_ (or H5_Piperazine_)), 2.79 (m, 2H, NH(C(=O)CH_2_CH_2_), 2.76 (m, 2H, NH(C(=O)CH_2_CH_2_)), 1.46 (s, 9H, CH_3_); **^13^C NMR** (25 °C, CDCl_3_): 171.18 (NHC(=O)), 170.28 (C(=O)Pip), 154.51 (C(=O)O*t*-Bu), 149.77 (C2_Pyridine_), 145.95 (C6_Pyridine_), 138.11 (C4_Pyridine_), 126.39 (C5_Pyridine_), 114.78 (C3_Pyridine_), 80.36 (C(CH_3_)_3_), 45.18 (C2_Piperazine_ and C3_Piperazine_ (or C6_Piperazine_ and C5_Piperazine_)), 43.59 (C3_Piperazine_ (or C5_Piperazine_)), 41.72 (C2_Piperazine_ (or C6_Piperazine_)), 32.34 (NH(C(=O)CH_2_CH_2_), 28.35 (CH_3_), 28.27 (NH(C(=O)CH_2_CH_2_); **HRMS**: calcd for C_18_H_26_ClN_4_O_4_: 397.1643 found: 397.1651 [M + H]^+^.

t-butyl 4-[4-[(3-pyridinyl)amino]-1,4-dioxobutyl]-1-piperazine carboxylate **6c**

The compound **6c** was synthesized using **5c** (0.97 g, 5 mmol, 1.0 eq.), 0.96 g (5 mmol, 1.0 eq.) of EDCI × HCl, 1.0 mL (5 mmol, 1.0 eq.) of DIPEA and 0.88 g (4.8 mmol, 0.94 eq.) of *N*-Boc-piperazine in 25 mL of DMF. After workup and purification as described for **6a** 1.25 g (3.5 mmol) of the product was obtained. Yield: 70%. **MP**: 138 °C (dec); **^1^H NMR** (25 °C, CDCl_3_): 8.96 (s, 1H, NH), 8.56 (d, 1H, H2_Pyridine_), 8.28 (dd, 1H, H6_Pyridine_), 8.08 (d, 1H, H4_Pyridine_), 7.19 (dd, 1H, H5_Pyridine_), 3.60 (m, 2H, H2_Piperazine_ (or H6_Piperazine_), 3.48 (s, 4H, H6_Piperazine_ and H5_Piperazine_ (or H2_Piperazine_ and H3_Piperazine_)), 3.41 (m, 2H, H3_Piperazine_ (or H5_Piperazine_)), 2.77 (s, 4H, both CH_2_), 1.46 (s, 9H, CH_3_); **^13^C NMR** (25 °C, CDCl_3_): 171.19 (NHC(=O)), 170.68 (C(=O)Pip), 154.45 (C(=O)O*t*-Bu), 144.78 (C6_Pyridine_), 141.04 (C2_Pyridine_), 135.14 (C3_Pyridine_), 126.67 (C4_Pyridine_), 123.48 (C5_Pyridine_), 80.43 (C(CH_3_)_3_), 45.18 (C2_Piperazine_ and C3_Piperazine_ (or C6_Piperazine_ and C5_Piperazine_)), 41.72 (C6_Piperazine_ and C5_Piperazine_ (or C2_Piperazine_ and C3_Piperazine_)), 32.31 (NHC(=O)CH_2_CH_2_), 28.69 (NHC(=O)CH_2_CH_2_), 28.32 (CH_3_); **HRMS**: calcd for C_18_H_27_N_4_O_4_: 363.2032 found: 363.2032 [M + H]^+^.

t-Butyl 4-[4-(2-chloro-3-pyridinylamino)-1,4-dioxobutyl]-1-piperazine carboxylate **6d**

The compound **6d** was synthesized using **5d** (0.38 g, 1.7 mmol, 1.0 eq.), 0.32 g (1.7 mmol, 1.0 eq.) of EDCI × HCl, 0.35 mL (1.7 mmol, 1.0 eq.) of DIPEA and 0.29 g (1.6 mmol, 0.94 eq.) of *N*-Boc-piperazine in 15 mL of DMF. After workup and purification as described for (**6a**) 0.15 g (0.4 mmol) of the product was obtained. Yield: 24%. **MP**: 172 °C; **^1^H NMR** (25 °C, CDCl_3_): 8.70 (d, 1H, H6_Pyridine_), 8.48 (bs, 1H, NH), 8.09 (dd, 1H, H4_Pyridine_), 7.22 (dd, 1H, H5_Pyridine_), 3.62 (m, 2H, H5_Piperazine_ (or H3_Piperazine_)), 3.48 (m, 4H, H3_Piperazine_ and H2_Piperazine_ (or H5_Piperazine_ and H6_Piperazine_), 3.42 (m, 2H, H6_Piperazine_ (or H2_Piperazine_), 2.82 (m, 2H, NHC(=O)CH_2_CH_2_), 2.77 (m, 2H, NH(C(=O)CH_2_CH_2_), 1.47 (s, 9H, CH_3_); **^13^C NMR** (25 °C, CDCl_3_): 171.40 (NHC(=O)), 170.07 (C(=O)Pip), 154.52 (C(=O)O*t*-Bu), 143.64 (C6_Pyridine_), 139.97 (C2_Pyridine_), 132.17 (C4_Pyridine_), 129.02 (C3_Pyridine_), 123.12 (C5_Pyridine_), 80.42 (C(CH_3_)_3_), 45.12 (C2_Piperazine_ and C3_Piperazine_ (or C6_Piperazine_ and C5_Piperazine_)), 41.73 (C6_Piperazine_ and C5_Piperazine_ (or C2_Piperazine_ and C3_Piperazine_)), 32.79 (NHC(=O)CH_2_CH_2_), 28.79 (NHC(=O)CH_2_CH_2_), 28.34 (CH_3_); **HRMS**: calcd for C_18_H_24_ClN_4_O_4_: 395.1486 found: 395.1489 [M + H]^–^.

4-Oxo-*N*-4-pyridinyl-1-piperazinebutanamide dihydrochloride **YAN-156**

The compound **6a** (0.11 g, 0.30 mmol) was dissolved in 1 mL of chloroform. To this solution 2 mL of 6M HCl_(g)_ in AcOEt/EtOH (1:1 *v*/*v*) was added resulting in precipitation of a solid. The product was filtered off, washed with cold MTBE and dried under vacuum obtaining 0.10 g (0.29 mmol). Yield: 97%; **Purity**: 99.70% (HPLC); **MP**: 215 °C (dec); **^1^H NMR** (25 °C, D_2_O): 8.47 (d, 2H, H2_Pyridine_ and H6_Pyridine_), 7.99 (d, 2H, H3_Pyridine_ and H5_Pyridine_), 3.84 (t, 2H, H2_Piperazine_ (or H6_Piperazine_), 3.75 (t, 2H, H6_Piperazine_ (or H2_Piperazine_), 3.21 (t, 2H, H5_Piperazine_ (or H3_Piperazine_), 3.30 (t, 2H, H3_Piperazine_ (or H5_Piperazine_), 2.80 (s, 4H, both CH_2_); **^13^C NMR** (25 °C, D_2_O): 175.09 (NHC(=O)), 172.73 (C(=O)Pip), 153.08 (C4_Pyridine_), 141.59 (C2_Pyridine_ and C6_Pyridine_), 115.03 (C3_Pyridine_ and C5_Pyridine_), 42.97 (C3_Piperazine_ or C5_Piperazine_), 42.92 (C5_Piperazine_ or C3_Piperazine_), 42.17 (C2_Piperazine_ or C6_Piperazine_), 38.58 (C6_Piperazine_ or C2_Piperazine_), 31.60 (NHC(=O)CH_2_), 27.01 (NHC(=O)CH_2_CH_2_); **HRMS**: calcd for C_13_H_19_N_4_O_2_: 263.1508 found: 263.1512 [M + H]^+^, calcd for C_13_H_18_N_4_O_2_Na: 285.1327 found: 285.1334 [M + Na]^+^.

4-Oxo-*N*-(5-chloro-2-pyridinyl)-1-piperazinebutanamide dihydrochloride **YAN-157**

The compound **YAN-157** was obtained using **6b** (0.23 g, 0.58 mmol) as described for **YAN-156** and resulting in 0.17 g (0.46 mmol) of the product. Yield: 79%; **Purity**: 96.88% (HPLC); **MP**: 180 °C (dec); **^1^H NMR** (25 °C, DMSO-*d*_6_): 10.69 (s, 1H, NH), 9.94, 9.59 (2 × s, 1H, NH_Piperazine_), 8.33 (d, 1H, H6_Pyridine_), 8.07 (d, 1H, H3_Pyridine_), 7.86 (dd, 1H, H4_Pyridine_), 3.72 (m, 2H, H2 (or H6)_Piperazine_), 3.65 (m, 2H, H6 (or H2)_Piperazine_), 3.08 (m, 2H, H3 (or H5)_Piperazine_), 2.99 (m, 2H, H5 (or H3)_Piperazine_), 2.64 (s, 4H, both CH_2_); **^13^C NMR** (25 °C, DMSO-*d*_6_): 171.66 (NHC(=O)), 170.15 ((O=)CPip), 150.74 (C2_Pyridine_), 146.17 C6_Pyridine_), 137.87 C4_Pyridine_), 124.69 C5_Pyridine_), 114.41 C3_Pyridine_), 42.59 (C3_Piperazine_ or C5_Piperazine_), 42.43 (C5_Piperazine_ or C3_Piperazine_), 41.62 (C2_Piperazine_ or C6_Piperazine_), 37.86 (C6_Piperazine_ or C2_Piperazine_), 31.08 (NHC(=O)CH_2_CH_2_), 27.06 (NHC(=O)CH_2_); **HRMS**: calcd for C_13_H_18_ClN_4_O_2_: 297.1118 found: 297.1124 [M + H]^+^, calcd for C_13_H_17_ClN_4_O_2_Na: 319.0945 found: 319.0945 [M + Na]^+^.

4-Oxo-*N*-3-pyridinyl-1-piperazinebutanamide **YAN-158**

The compound **YAN-158** was obtained using **6c** (0.23 g, 0.64 mmol) as described for **YAN-156** and resulting in 0.11 g (0.34 mmol) of the product. Yield: 53%; **Purity**: 97.00% (HPLC); **MP**: 175 °C; **^1^H NMR** (25 °C, DMSO-*d*_6_): 11.41 (s, 1H, NH), 9.56 (s, 2H, NH_Piperazine_), 9.21 (d, 1H, 8.56 (s, 1H, H2_Pyridine_), 8.56 (s, 1H, H4_Pyridine_), 8.56 (s, 1H, H6_Pyridine_), 7.95 (dd, 1H, H5_Pyridine_), 3.74 (m, 2H, H6 (or H2)_Piperazine_), 3.66 (m, 2H, H2 (or H6)_Piperazine_), 3.10 (m, 2H, H5 (or H3)_Piperazine_), 3.00 (m, 2H, H3 (or H5)_Piperazine_), 2.70 (m, 4H, both CH_2_); **^13^C NMR** (25 °C, DMSO-*d*_6_): 172.15 (NHC(=O)), 169.94 ((O=)CPip), 138.62 (C3_Pyridine_), 136.31 (C6_Pyridine_), 133.32 (C4_Pyridine_), 131.67 (C2_Pyridine_), 127.26 (C5_Pyridine_), 42.57 (C5_Piperazine_ or C3_Piperazine_), 42.40 (C3_Piperazine_ or C5_Piperazine_), 41.58 (C6_Piperazine_ or C2_Piperazine_), 37.86 (C2_Piperazine_ or C6_Piperazine_), 31.19 (NHC(=O)CH_2_CH_2_), 27.03 (NHC(=O)CH_2_CH_2_); **HRMS**: calcd for C_13_H_19_N_4_O_2_: 263.1510 found: 263.1510 [M + H]^+^, calcd for C_13_H_18_N_4_O_2_Na: 285.132 found: 285.1330 [M + Na]^+^.

4-Oxo-*N*-(2-chloro-3-pyridinyl)-1-piperazinebutanamide **YAN-159**

The compound **YAN-159** was obtained using **6d** (0.14 g, 0.35 mmol) as described for **YAN-156** and resulting in 0.05 g (0.15 mmol) of the product. Yield: 43%; **Purity**: 99.56% (HPLC); **MP**: 186 °C (dec); **^1^H NMR** (25 °C, DMSO-*d*_6_): 9.71 (s, 1H, NH), 9.41 (s, 2H, NH_Piperazine_), 8.16 (2 × d, 2H, H4_Pyridine_ and H6_Pyridine_), 7.41 (dd, 1H, H5_Pyridine_), 3.72 (m, 2H, H2 (or H6)_Piperidine_), 3.67 (m, 2H, H6 (or H2)_Piperidine_), 3.10 (m, 2H, H5 (or H3)_Piperidine_), 3.00 (m, 2H, H3 (or H5)_Piperidine_), 2.68 (s, 4H, both CH_2_); **^13^C NMR** (25 °C, DMSO-*d*_6_): 171.43 (NHC(=O)), 170.13 ((O=)CPip), 144.97 (C6_Pyridine_), 142.71 (C2_Pyridine_), 133.56 (C4_Pyridine_), 132.08 (C3_Pyridine_), 123.30 (C5_Pyridine_), 42.60 (C5_Piperidine_ or C3_Piperidine_), 42.46 (C3_Piperidine_ or C5_Piperidine_), 41.64 (C6_Piperidine_ or C2_Piperidine_), 37.90 (C2_Piperidine_ or C6_Piperidine_), 30.84 (NHC(=O)CH_2_CH_2_), 27.22 (NHC(=O)CH_2_CH_2_); **HRMS**: calcd for C_13_H_18_ClN_4_O_2_: 297.1122 found: 297.1122 [M + H]^+^, calcd for C_13_H_17_ClN_4_O_2_Na: 319.0938 found: 319.0941 [M + Na]^+^.

### 3.3. In Vitro Biological Studies

#### 3.3.1. Histamine H_4_ Receptor Affinity

The radioligand competition binding experiments were performed in membrane fractions of Sf9 cells co-infected with baculoviruses encoding hH_4_R, Gα_i2_ and Gβ_1_γ_1_ subunit. Cell infection, cultivation and membrane preparation was conducted according to Kottke et al. [48]. Prior to the experiments, cell membranes were thawed at 4 °C, homogenized by sonication on ice and maintained in ice cold binding buffer (12.5 mM MgCl_2_, 1 mM EDTA and 75 mM Tris/HCl, pH 7.4). For the radioligand displacement assay, membrane fraction (40 µg/well) was incubated with [^3^H]-Histamine (10 nM, 16.40 Ci/mmol) and test compound (1 µM) in a total volume of 200 µL for 60 min at room temperature. To determine non-specific binding, **JNJ-7777120** was used at 100 µM. Radioactivity was determined by liquid scintillation counting. Each compound was screened in triplicate one-point measurements at a test concentration of 1 µM in 2–3 separate experiments, and percent inhibition was calculated relative to total radioligand binding. Non-specific binding was subtracted from the raw data. Global mean values ± SEM of all experiments were calculated with GraphPad Prism v. 9.3.1 (San Diego, CA, USA).

#### 3.3.2. Cell Viability and Cell Proliferation Evaluation

The effect of the tested compounds on cell viability was assessed in CHO-K1 cells stably expressing hH_4_R (CHO-H_4_R) by measuring metabolic activity using the PrestoBlue^TM^ (Thermo Fisher Scientific/Invitrogen, Waltham, MA, USA) resazurin-based assay according to the manufacturer’s instructions. Cell proliferation was evaluated by measuring DNA synthesis in replicating cells with the BrdU Cell Proliferation Assay (Calbiochem, Merck KGaA, Darmstadt, Germany), following the manufacturer’s protocol. Compounds were dissolved in DMSO (stock concentrations: 20 mM for **YAN-151**–**YAN-155** and **YAN-157**; 50 mM for **YAN-156**, **YAN-158** and **YAN-159**) and applied at final concentrations of 10, 100 and 1000 µM. The corresponding DMSO content was ≤0.05% at 10 µM and ≤0.5% at 100 µM, and 5% (**YAN-151**–**YAN-155** and **YAN-157**) or 2% (**YAN-158**, **YAN-159**) at 1000 µM. Vehicle controls containing 2% and 5% DMSO were included in every experiment. Readouts were performed after 48 h incubation of cells with the tested compounds. Each experiment was carried out in three independent repetitions, each performed in triplicate.

#### 3.3.3. cAMP Accumulation Assay

Intrinsic activity of the tested compounds in the *G* protein-dependent pathway was analyzed in a cAMP accumulation assay. CHO cells stably expressing human H_4_R (PerkinElmer, Waltham, MA, USA) were incubated for 30 min at room temperature with histamine (140 nM), forskolin (10 µM) and the evaluated compounds (10 concentrations spanning 5 logarithmic units), in the presence of phosphodiesterase inhibitor RO-201724 (100 µM).

Intracellular cAMP levels were then measured using a homogenous TR-FRET immunoassay (LANCE^®^ Ultra cAMP kit, PerkinElmer, Waltham, MA, USA), according to the manufacturer’s instructions. The TR-FRET signal, which is inversely proportional to the cAMP concentration in the sample, was recorded with an EnVision microplate reader (PerkinElmer, Waltham, MA, USA) and converted to absolute cAMP concentrations using a standard curve.

Sigmoidal dose–response curves were fitted with GraphPad Prism software (version 8.4.3, San Diego, CA, USA), using cAMP concentration as a function of ligand concentration. The graphs shown represent the mean of three independent experiments, each performed in triplicate.

#### 3.3.4. β-Arrestin Recruitment Assay

The cell-based LiveBLAzer^®^ assay (Thermo Fisher Scientific, Waltham, MA, USA) was used to determine the activity of H_4_R ligands in the β-arrestin pathway. In this model, recruitment of β-arrestin-2 to human H_4_R (hH_4_R) leads to the release of a β-lactamase transcription factor and activation of a β-lactamase reporter. β-lactamase activity is then detected using a FRET-based substrate, which under intact conditions emits a green fluorescent signal (detected at 530 nm). Cleavage of the β-lactam ring by β-lactamase disrupts FRET, resulting in a decrease in the 530 nm signal and a relative increase at 460 nm, which indirectly reflects β-arrestin recruitment upon receptor activation.

For the experiments, Tango-H4-bla U2OS cells were cultured in McCoy’s 5A medium supplemented according to the vendor’s recommendation (10% dialyzed FBS, 0.1 mM NEAA, 25 mM HEPES, 1 mM sodium pyruvate, 100 U/mL penicillin/streptomycin, 200 µg/mL zeocin, 100 µg/mL geneticin and 50 µg/mL hygromycin; all from Thermo Fisher Scientific, Waltham, MA, USA). One day prior to the experiment, cells were detached from culture flasks using trypsin, counted and seeded into 384-well black-wall, clear-bottom plates at 10,000 cells/well in FreeStyle^™^ medium (Thermo Fisher Scientific, Waltham, MA, USA). After 24 h incubation, cell growth and confluence were verified microscopically, and serial dilutions of the test compounds in FreeStyle medium were prepared and added to the appropriate wells.

In agonist mode, compounds were added to the wells and plates were incubated for 16 h. In antagonist mode, test compounds were first added and plates were incubated for 30 min; subsequently, histamine was added at 500 nM (corresponding to the EC_80_ concentration), and plates were further incubated for 16 h.

β-Arrestin recruitment was detected by the addition of the β-lactamase substrate mixture followed by fluorescence measurement at 460 nm and 530 nm after 2 h incubation, using a Spark multimode microplate reader (Tecan, Männedorf, Switzerland). All calculations were based on background-corrected fluorescence ratios (460 nm/530 nm). The resulting ratios were plotted against compound concentration, and sigmoidal dose–response curves were fitted to derive EC_50_/IC_50_ values. The data shown represent the mean of three independent experiments, each performed in triplicate.

#### 3.3.5. Cytosolic Ca^2+^ Measurements

CHO-K1 cells stably expressing hH_4_R were cultured on glass coverslips in 35 mm dishes for 48 h. Confluent cells were loaded with 4 μM Fura-2 AM (Invitrogen/Thermo Fisher Scientific, Waltham, MA, USA) in culture medium for 30 min at 37 °C. The cells were then washed twice with the solution containing 5 mM KCl, 1 mM MgCl_2_, 0.5 mM Na_2_HPO_4_, 25 mM HEPES, 130 mM NaCl, 0.5 mM sodium pyruvate, 17.5 mM *d*-glucose, and 0.1 mM CaCl_2_ (pH 7.4). Coverslips were mounted in a cuvette containing 3 mL of assay solution (same composition but with 2 mM CaCl_2_) and placed at room temperature in a spectrofluorometer (Hitachi F-7000, Hitachi, Ltd., Tokyo, Japan). Cells were preincubated for 15 min with the tested compounds (**YAN-151**, **YAN-152**, **YAN-153**, **YAN-155**, **YAN-156**, each at 10 µM), after which 10 µM **4-MH** was added. In parallel, each compound was also applied alone, without subsequent **4-MH**, to test for intrinsic agonist activity. Fluorescence was recorded at 510 nm with excitation at 340 and 380 nm. At the end of each experiment, Fura-2 fluorescence was calibrated by the addition of 10 μM ionomycin to determine maximal fluorescence, followed by the addition of EGTA to chelate Ca^2+^.

#### 3.3.6. Data Analysis

All data analyses were performed using GraphPad Prism (version 9.3.1). Concentration–response curves for cAMP accumulation and β-arrestin recruitment were fitted by nonlinear regression using a four-parameter logistic model to obtain EC_50_/IC_50_ values. Data are presented as mean ± SD or SEM, as indicated, from three independent experiments, unless stated otherwise. No formal statistical hypothesis testing was applied, given the exploratory nature of the study.

## 4. Conclusions

In summary, we designed and synthesized nine new pyrido[2,3-*d*]pyrimidine and pyrimidine analogs that did not exhibit any relevant cytotoxic or antiproliferative effects at concentrations up to 1 mM. In the single-concentration [^3^H]histamine binding assay at human H_4_R, the pyrimidine series showed generally lower binding, whereas several pyrido[2,3-*d*]pyrimidine derivatives (**YAN-151**, **YAN-153**, and **YAN-155**) and one pyrimidine analog (**YAN-156**) produced >60% inhibition at 1 µM. Among them, **YAN-153** showed the highest inhibition (69.9%). To further explore structure–activity relationship (SAR), a matched pair from the pyrido[2,3-*d*]pyrimidine series differing only at the R2 position, **YAN-153** (higher binding) and **YAN-152** (moderate binding), was selected for detailed functional studies in cAMP and β-arrestin assays. Across the active binders, the Ca^2+^ assay revealed divergent functional profiles (inhibition, no effect, and enhancement of the **4-MH** response), underscoring that binding affinity and functional efficacy are independent properties at H_4_R.

In the *G*i–cAMP pathway, both compounds acted as moderate H_4_R antagonists, reversing histamine-induced inhibition of cAMP accumulation with similar potencies (IC_50_ ≈ 9–10 µM), clearly weaker than the reference antagonist **JNJ-7777120**. In contrast, their profiles diverged in the β-arrestin pathway as follows: **YAN-152** displayed a predominantly antagonistic behavior with minimal agonist activity (E_max_ = 3 ± 5%), whereas **YAN-153** showed weak partial agonism (E_max_ = 29 ± 6%) and limited antagonistic efficacy (IC_50_ > 10 µM). These findings suggest ligand-dependent differences in signaling across *G*-protein and β-arrestin pathways at H_4_R.

In the Ca^2+^ mobilization assay, both compounds reduced the **4-MH**-evoked Ca^2+^ response, with **YAN-153** producing the strongest inhibition and **YAN-152** exerting a more moderate effect. Overall, **YAN-153** emerges as a promising lead structure for further optimization and for more detailed in vitro (e.g., metabolic stability, selectivity) and in vivo studies, while the contrasting signaling profiles of **YAN-152** and **YAN-153** warrant additional investigation of potential functional selectivity at the H_4_ receptor.

## Figures and Tables

**Table 1:** Inhibition of [^3^H]histamine binding to human H_4_R by test compounds **YAN-151**–**YAN-159** and the reference antagonist **JNJ-7777120**. Values greater than 60% are highlighted in bold.

Compound’s Symbol	Inhibition at 1 μM [%] ^1^	SEM	*n*
**YAN-151**	**66.1**	4.2	3
**YAN-152**	45.7	6.5	3
**YAN-153**	**69.9**	5.2	3
**YAN-154**	39.2	10.4	2
**YAN-155**	**60.8**	1.9	3
**YAN-156**	**64.1**	1.6	3
**YAN-157**	42.8	16.0	3
**YAN-158**	53.4	6.1	3
**YAN-159**	46.3	4.2	3
**JNJ-7777120** (100 μM, non-specific binding control)	100	0.4	3

^1^ Values are expressed as mean ± SEM (*n* = 2–3). Specific radioligand binding in the vehicle control was set to 100%. Percent inhibition was calculated as 100 minus the remaining specific [^3^H]histamine (10 nM) binding measured in the presence of the test compound at 1 µM. **JNJ-7777120** was used at 100 µM to define non-specific binding, which was subtracted from all raw data. At the saturating concentration used, **JNJ-7777120** was assumed to produce complete displacement of specific binding and thereby define non-specific binding. The value reported for **JNJ-7777120** therefore represents an operational reference point rather than a potency measurement comparable with those obtained for the test compounds at 1 µM.

**Table 2:** Intrinsic activity of tested compounds at histamine H_4_ receptor in *G* protein-dependent and independent pathways.

Compound	cAMP Accumulation (Antagonist Mode) IC_50_ ± SD [µM]	β-Arrestin Recruitment (Antagonist Mode) IC_50_ ± SD [µM]	β-Arrestin Recruitment (Agonist Mode) % Emax ± SD [%] ^1^
**4-MH**	N/A	N/A	EC_50_: 59.2 ± 10.1 nM
**JNJ-7777120**	0.0833 ± 0.030	0.0520 ± 0.0118	n.d.
**YAN-152**	9.91 ± 3.83	2.37 ± 0.56	3 ± 5
**YAN-153**	8.95 ± 3.03	>10	29 ± 6 ^1^

^1^ Compared to 4-methylhistamine (**4-MH**) at 10 µM; n.d., not determined; N/A, not applicable. Values are expressed as mean ± SD from three independent experiments. Due to limited DMSO tolerability in the β-arrestin recruitment assay, the maximal test compounds concentration tested was 10 µM.

## Data Availability

All data supporting the findings of this study are available within the article and its Supplementary Materials.

## Supplementary Materials

The following supporting information can be downloaded at: https://www.mdpi.com/article/10.3390/ijms27156892/s1.

## Abbreviations

The following abbreviations are used in this manuscript:

Glide XP	Glide extra precision
GPCRs	G protein-coupled receptors
H_1_R–H_4_R	Histamine receptors H_1_–H_4_
HRMS	High resolution mass spectrometry
IDEC	Inflammatory epidermal dendritic cells
IFD-MD	Induced-fit docking molecular dynamics
**4-MH**	4-methylhistamine
MM-GBSA	Molecular mechanics/generalized born surface area
NMR	Nuclear magnetic resonance
POPC	1-palmitoyl-2-oleoyl-*sn*-glycero-3-phosphocholine
QSAR	Quantitative structure–activity relationship
RA	Rheumatoid arthritis
SD	Standard deviation
SEM	Standard error of the mean
TR-FRET	Time-resolved fluorescence resonance energy transfer
VSGB	Variable solvent generalized born
