# Supplementary material for: Virtual Screening, Synthesis and In Vitro Characterization of Histamine H4 Receptor Ligands Based on Pyrimidine Scaffolds
Source: Int J Mol Sci. 2026 Aug 1;27(15):6892. doi: 10.3390/ijms27156892 (PMC13466428; doi:10.3390/ijms27156892)
Supplement: Supplementary file 1 [file ijms-27-06892-s001.zip › ijms-4367012-supplementary.pdf]

Supplementary Materials

Virtual Screening, Synthesis and In Vitro Characterization of Histamine H_4 Receptor Ligands Based on Pyrimidine Scaffolds

Olga Michalak [1,*], Marcin Cybulski [1], Piotr Krzeczyński [1], Oliwia Zegrocka-Stendel [2], Małgorzata Dutkiewicz [2], Dorota Dymkowska [3], Agnieszka Olejarz-Maciej [4], Tadeusz Karcz [4], Mariam Dubiel [5], Pakhuri Mehta [6,†], Marek Kubiszewski [7], Marcin Lorkowski [6], Jakub Jakowiecki [6], Paweł Pasznik [6], Przemysław Miszta [6], Holger Stark [5], Katarzyna Koziak [2] and Sławomir Filipek [6,*]

[1] Pharmacy, Cosmetic Chemistry and Biotechnology Research Group, Łukasiewicz Research Network-Industrial Chemistry Institute, 8 Rydygiera Str., 01-793 Warsaw, Poland; marcin.cybulski@ichp.lukasiewicz.gov.pl (M.C.); piotr.krzeczynski@ichp.lukasiewicz.gov.pl (P.K.)
[2] Department of Biochemistry and Nutrition, Centre for Preclinical Research and Technologies, Medical University of Warsaw, S. Banacha Str. 1b, 02-097 Warsaw, Poland; oliwia.stendel@wum.edu.pl (O.Z.-S.); malgorzata.dutkiewicz@wum.edu.pl (M.D.); katarzyna.koziak@wum.edu.pl (K.K.)
[3] Laboratory of Cellular Metabolism, Nencki Institute of Experimental Biology, Polish Academy of Sciences, Ludwika Pasteura Str. 3, 02-093 Warsaw, Poland; d.dymkowska@nencki.edu.pl (D.D.)
[4] Department of Chemical Technology and Biotechnology of Drugs, Jagiellonian University Medical College, Medyczna Str. 9, 30-688 Kraków, Poland; agnieszka.olejarz@uj.edu.pl (A.O.-M.); t.karcz@uj.edu.pl (T.K.)
[5] Institute for Pharmaceutical and Medicinal Chemistry, Heinrich Heine University Düsseldorf, Universitaetsstrasse 1, 40225 Duesseldorf, Germany; mariam.dubiel@hhu.de (M.D.), stark@hhu.de (H.S.)
[6] Faculty of Chemistry, Biological and Chemical Research Centre, University of Warsaw, Pasteura Str. 1, 02-093 Warsaw, Poland; pakhurimehta@gmail.com (P.M.); marcin.lorkowski@gmail.com (M.L.); jjakowiecki@chem.uw.edu.pl (J.J.); p.pasznik@gmail.com (P.P.); pmfenix@wp.pl (P.M.)
[7] Analytical Research Section, Pharmaceutical Analysis Laboratory, Łukasiewicz Research Network–Industrial Chemistry Institute, 8 Rydygiera Str., 01-793 Warsaw, Poland; marek.kubiszewski@ichp.lukasiewicz.gov.pl (M.K.)

* Correspondence: olga.michalak@ichp.lukasiewicz.gov.pl (O.M.); sh.filipek@uw.edu.pl (S.F.)
† Current address: Venkateshwar Institute of Pharmacy, Sai Tirupati University, Ambua Road, Umarda, Girwa, Udaipur 313015, India

Supplementary Materials

The compounds listed below appear in the order in which they are described in the main body of the article.

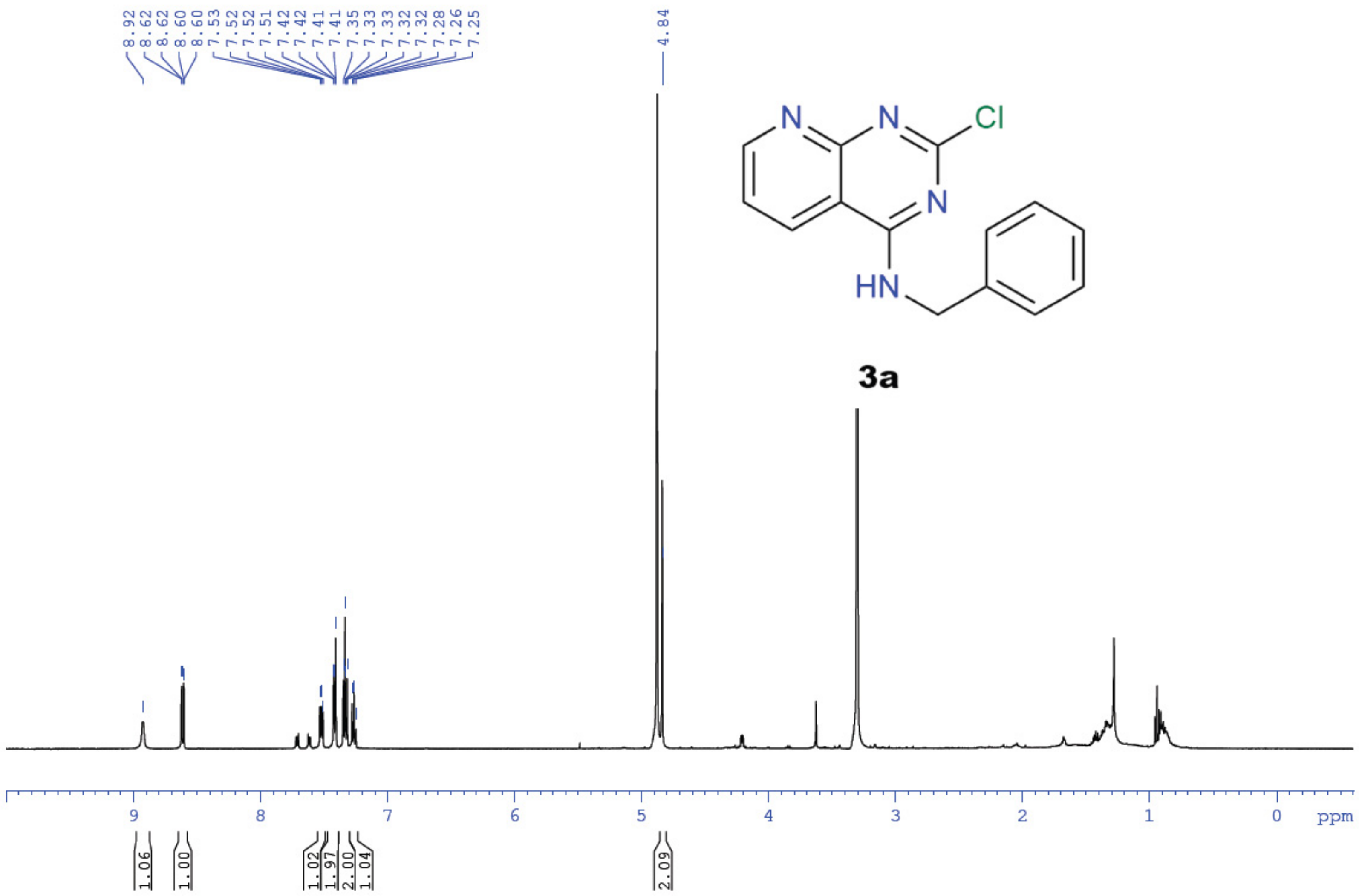

Figure S1. 3a — ¹H NMR spectrum

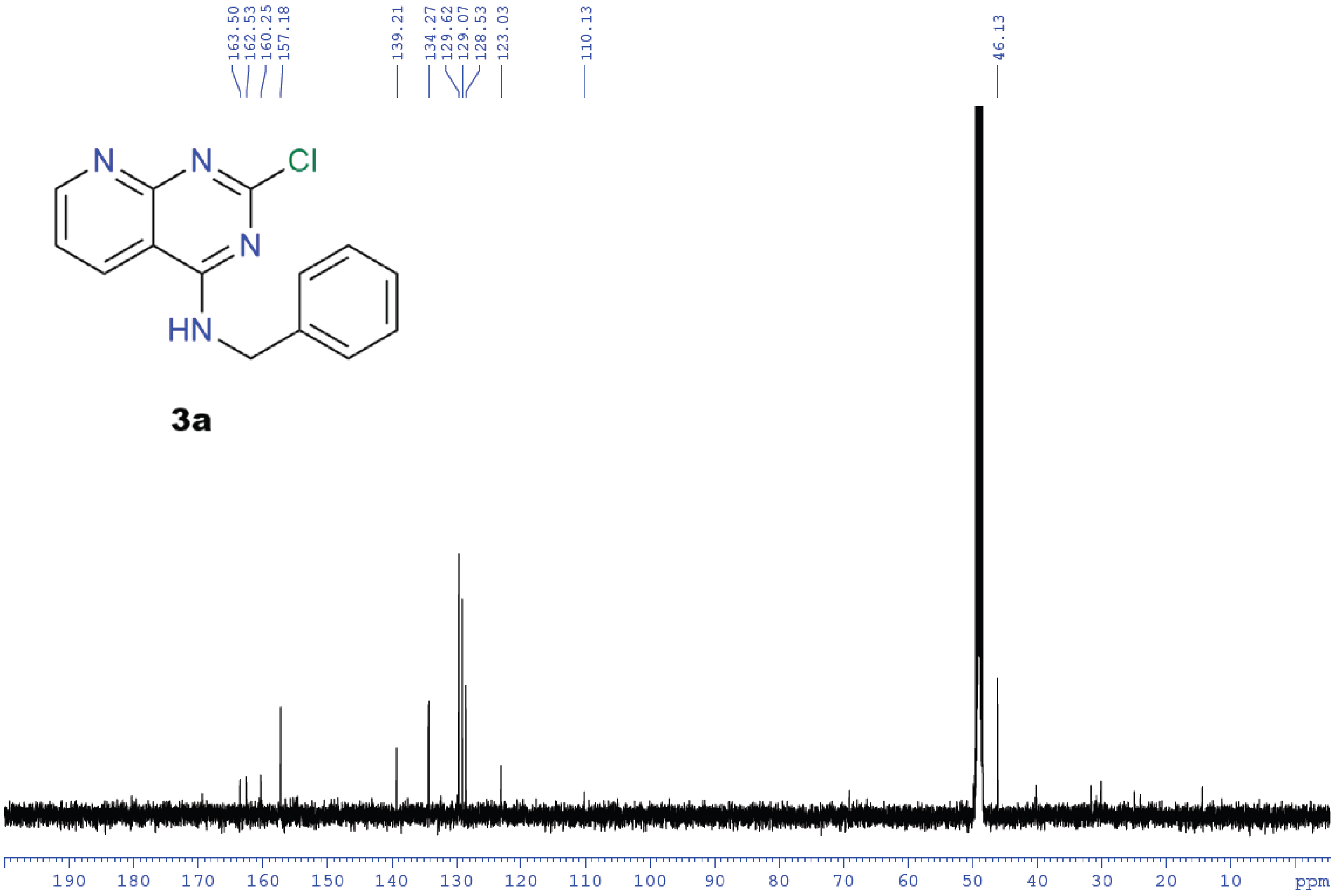

Figure S2. 3a — ^{13}C NMR spectrum

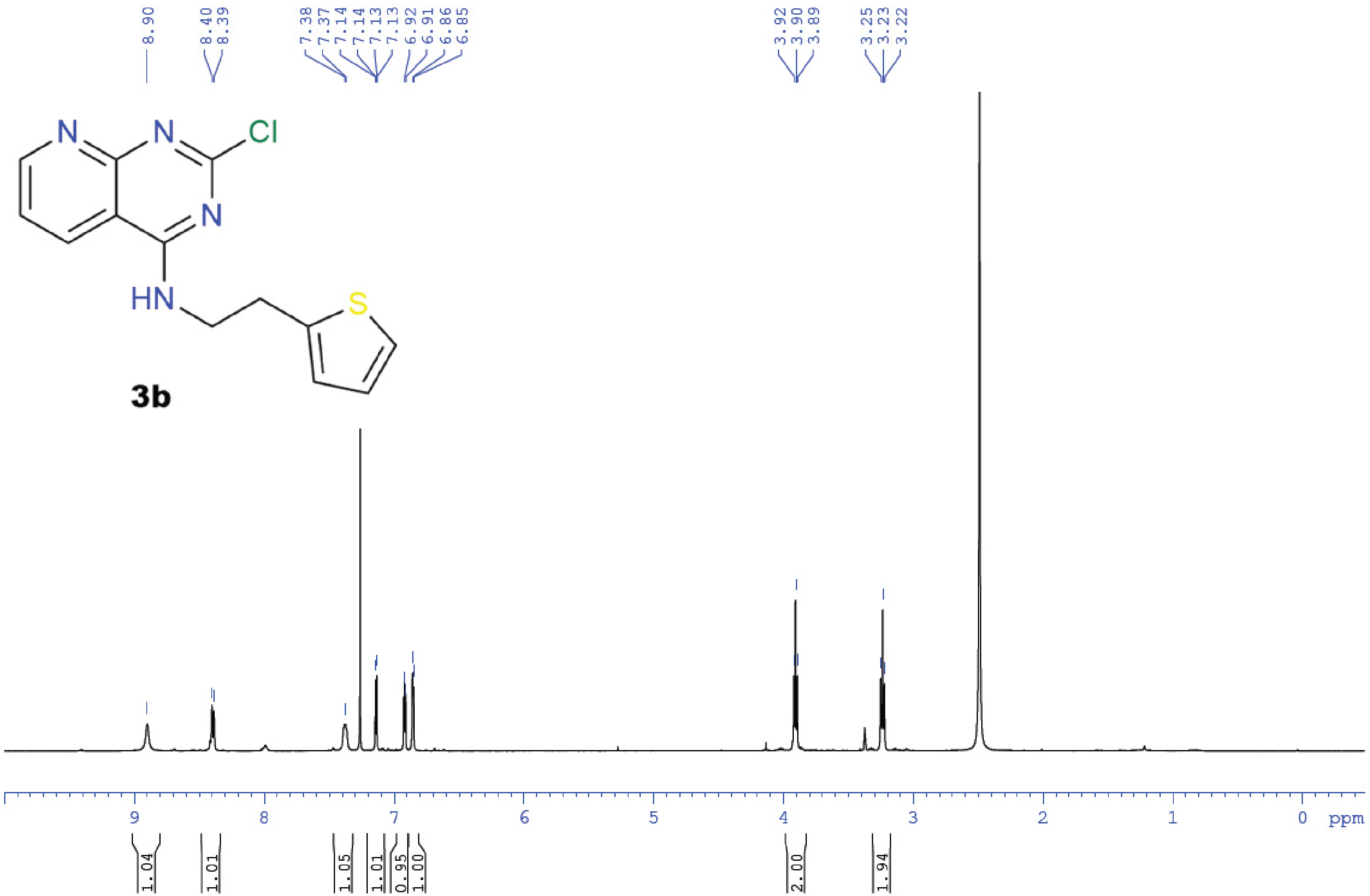

Figure S3. 3b — ^{1}H NMR spectrum

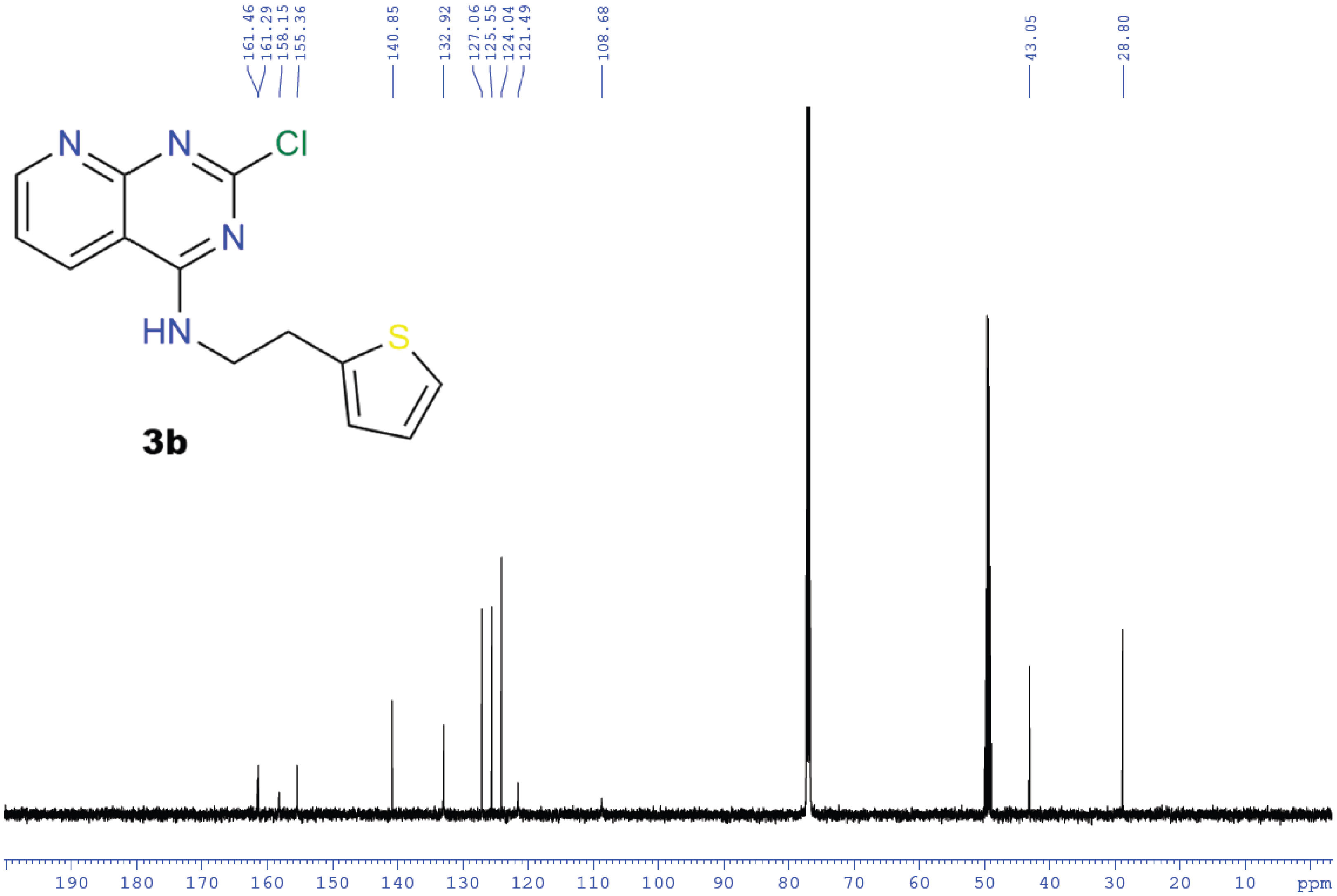

Figure S4. 3b — ^{13}C NMR spectrum

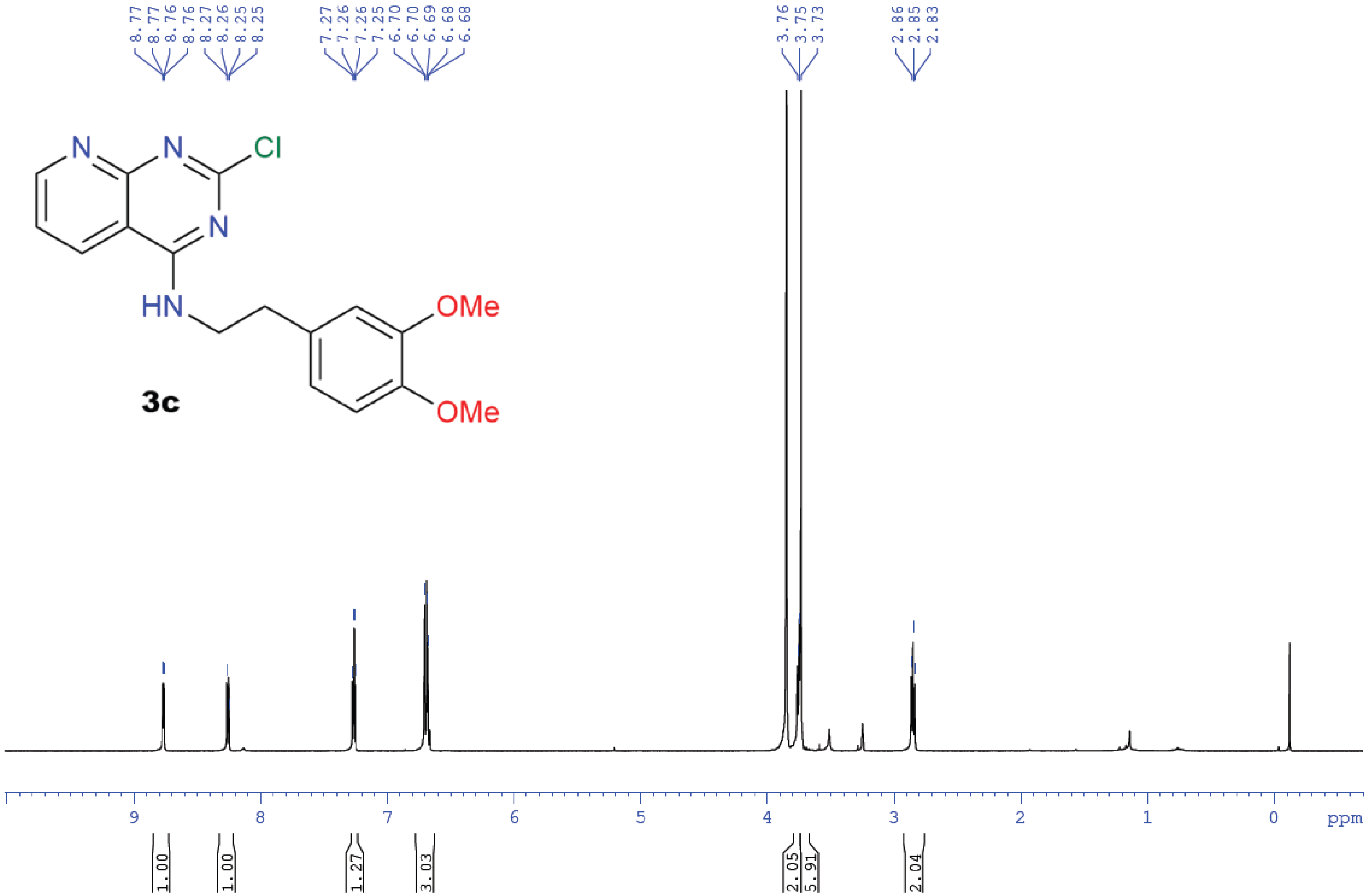

Figure S5. 3c — ^{1}H NMR spectrum

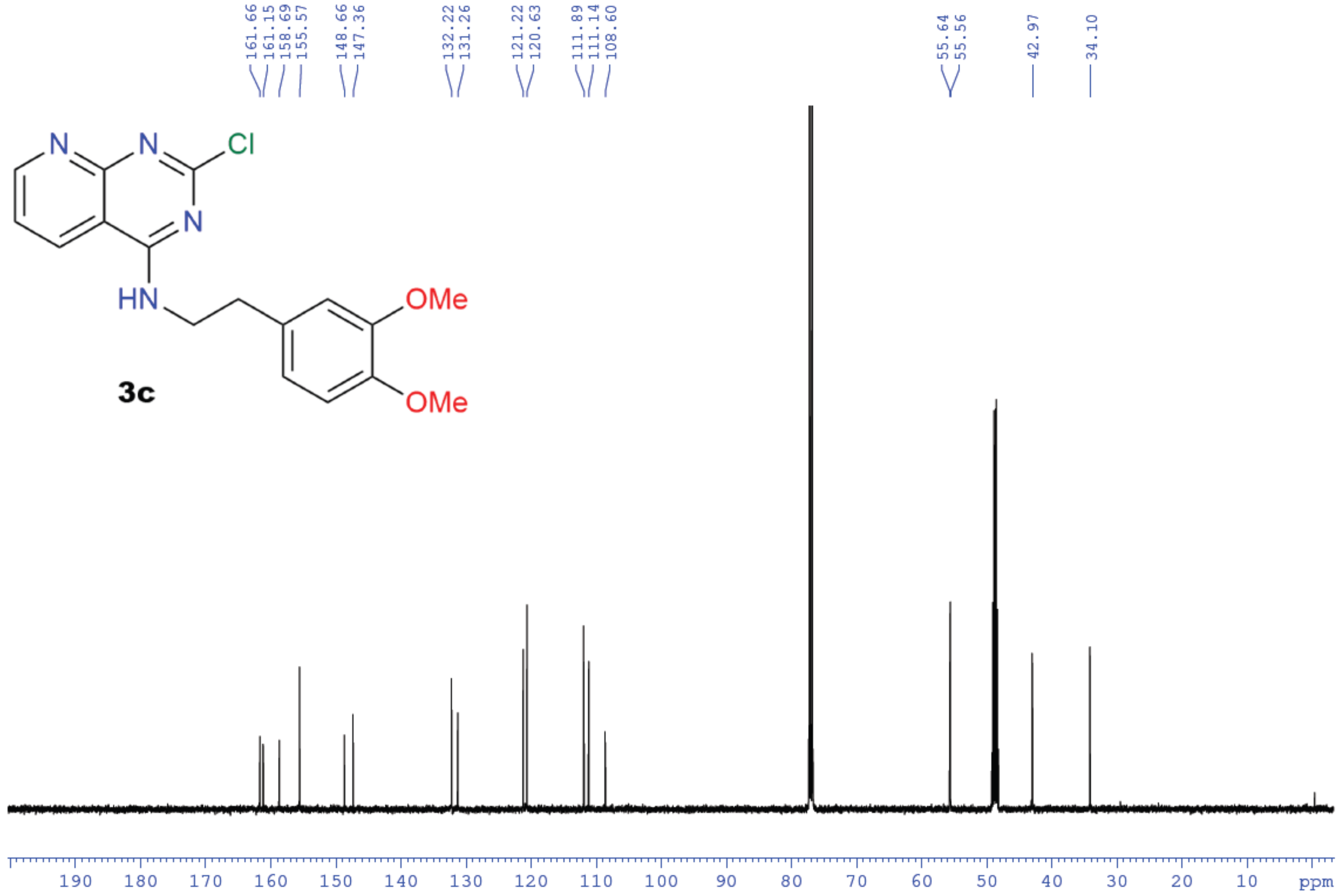

Figure S6. 3c — ^{13}C NMR spectrum

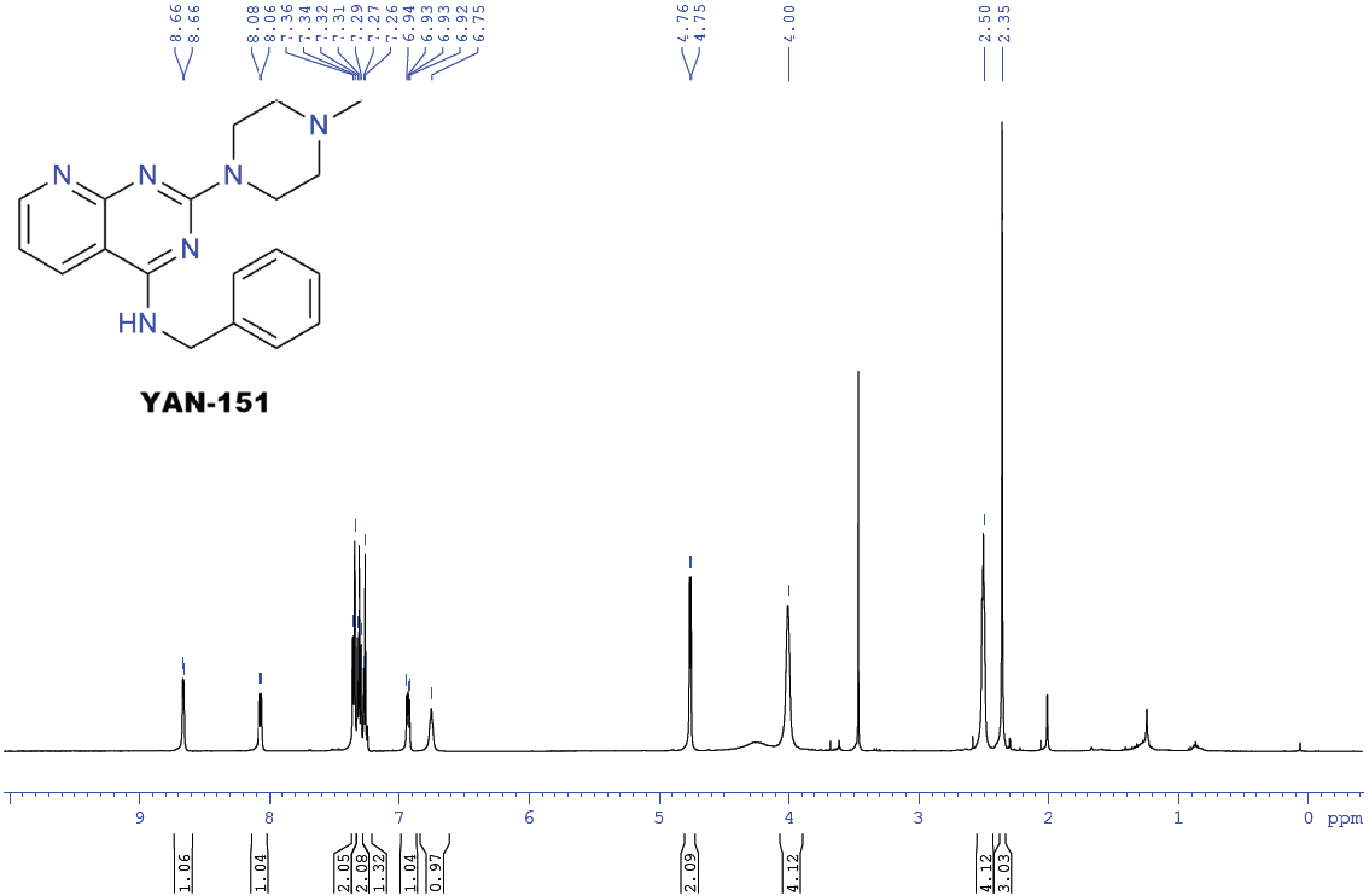

Figure S7. YAN-151 — ^{1}H NMR spectrum

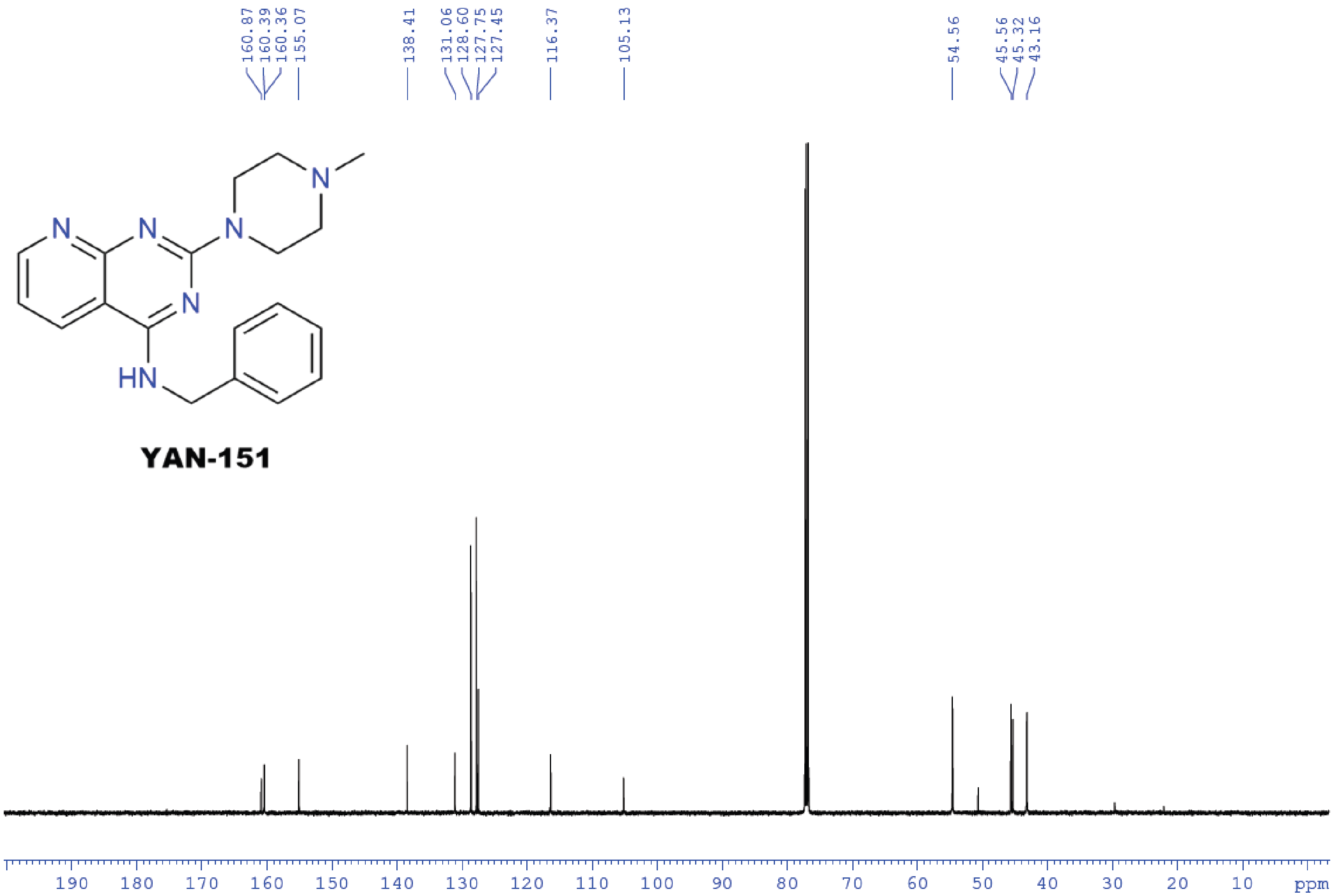

Figure S8. YAN-151 — ^{13}C NMR spectrum

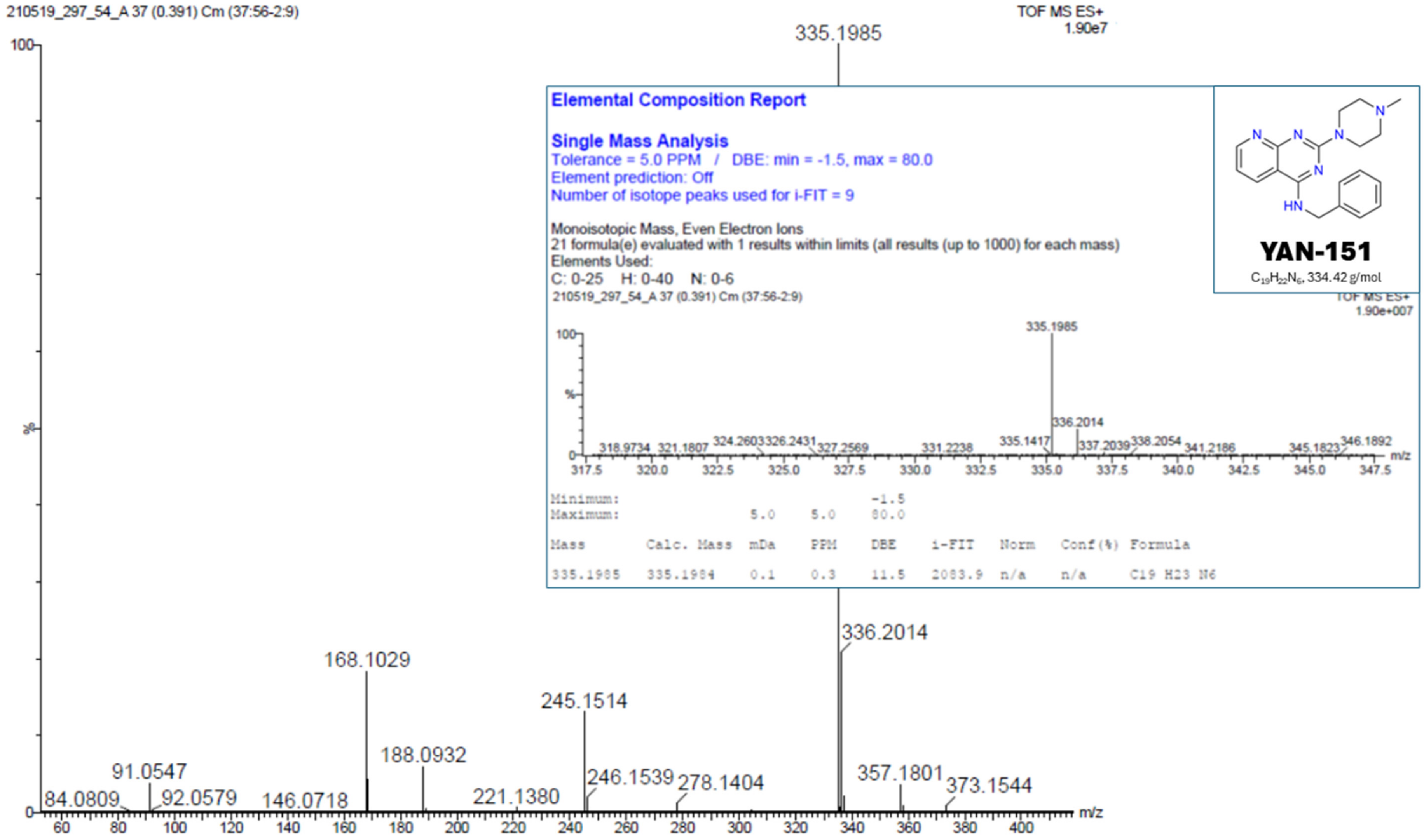

Figure S9. YAN-151 — HRMS (+) spectrum

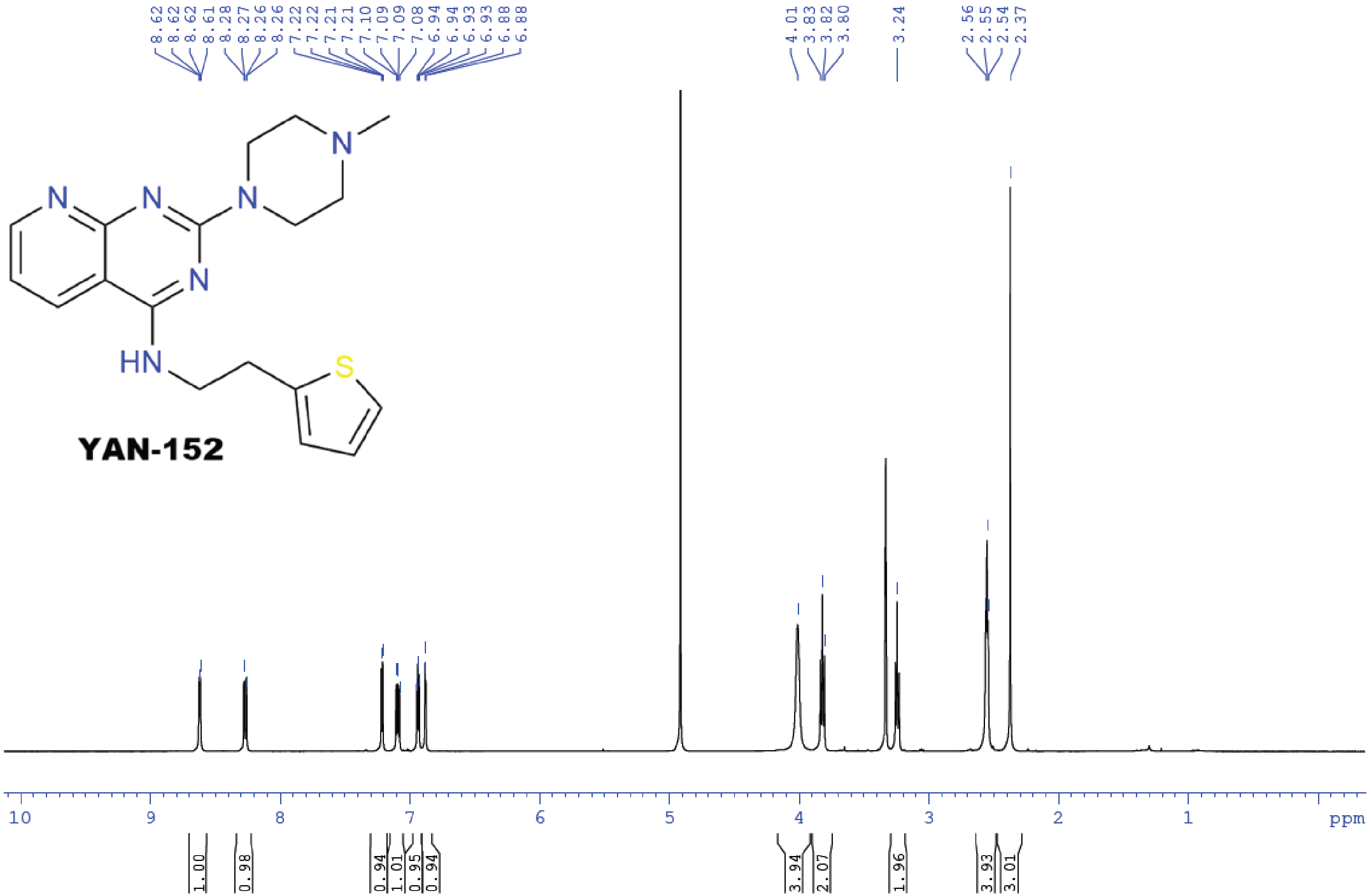

Figure S10. YAN-152 — ^{1}H NMR spectrum

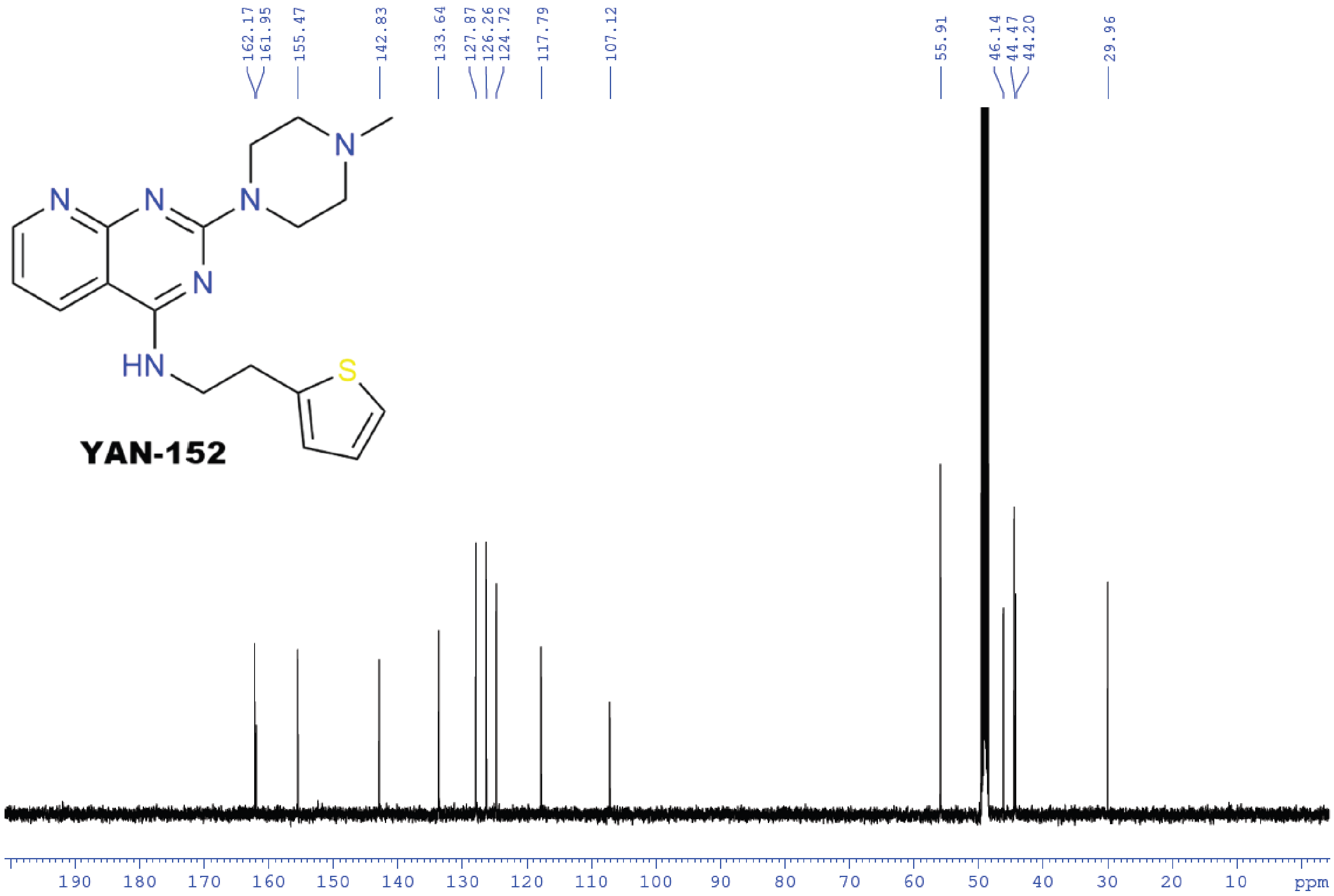

Figure S11. YAN-152 — ^{13}C NMR spectrum

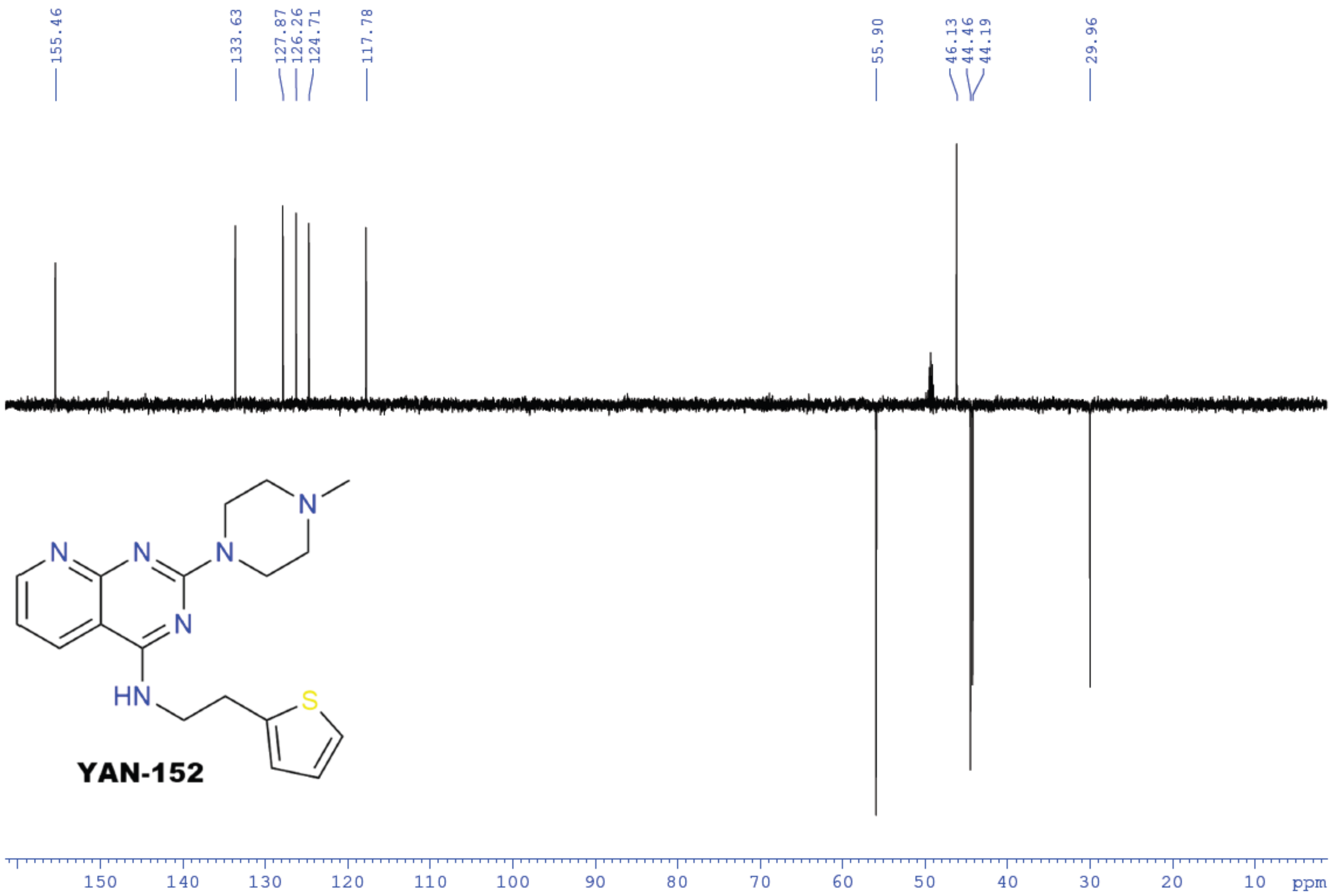

Figure S12. YAN-152 — DEPT NMR spectrum

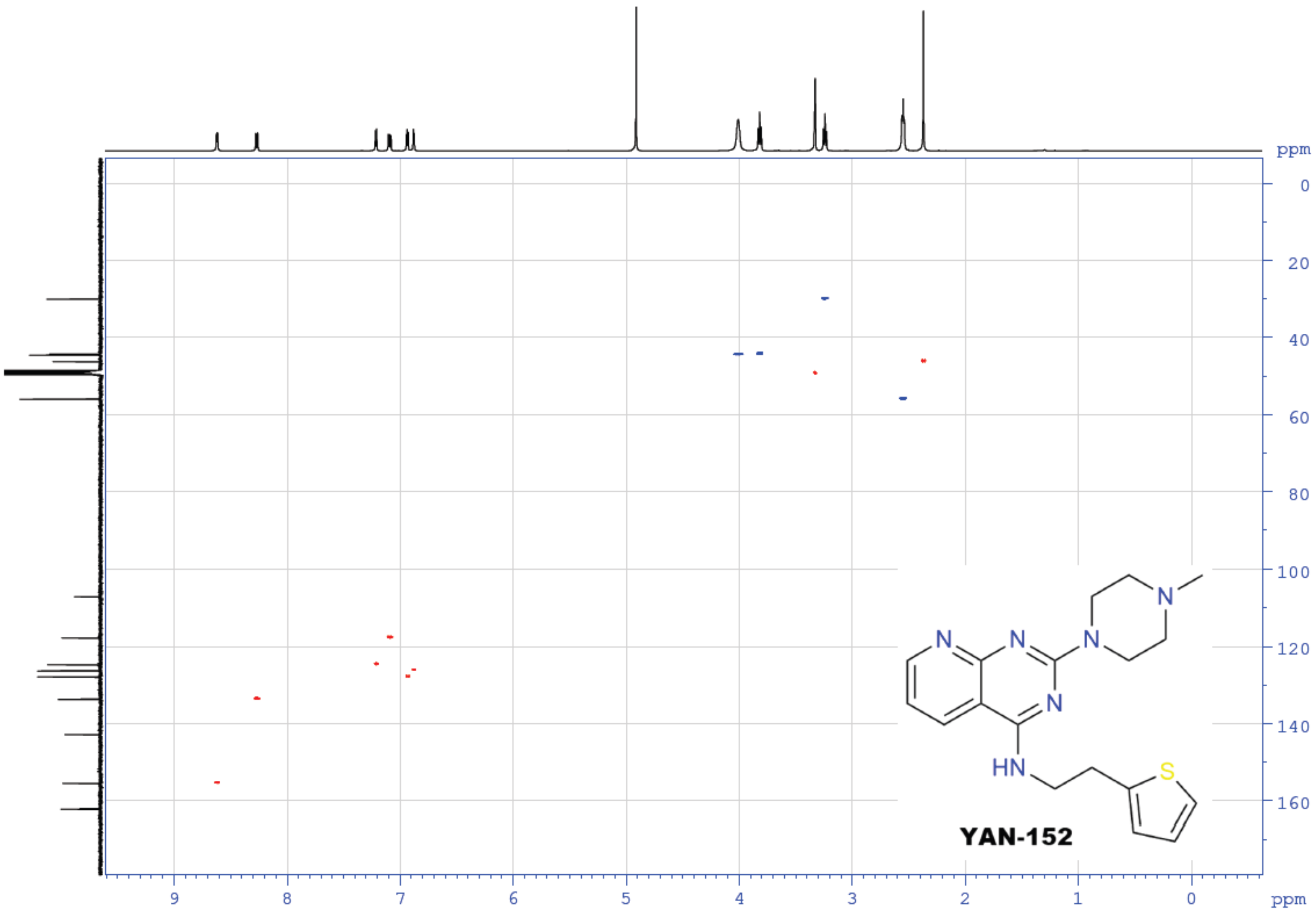

Figure S13. YAN-152 — ^{1}H-^{13}C HSQC NMR spectrum

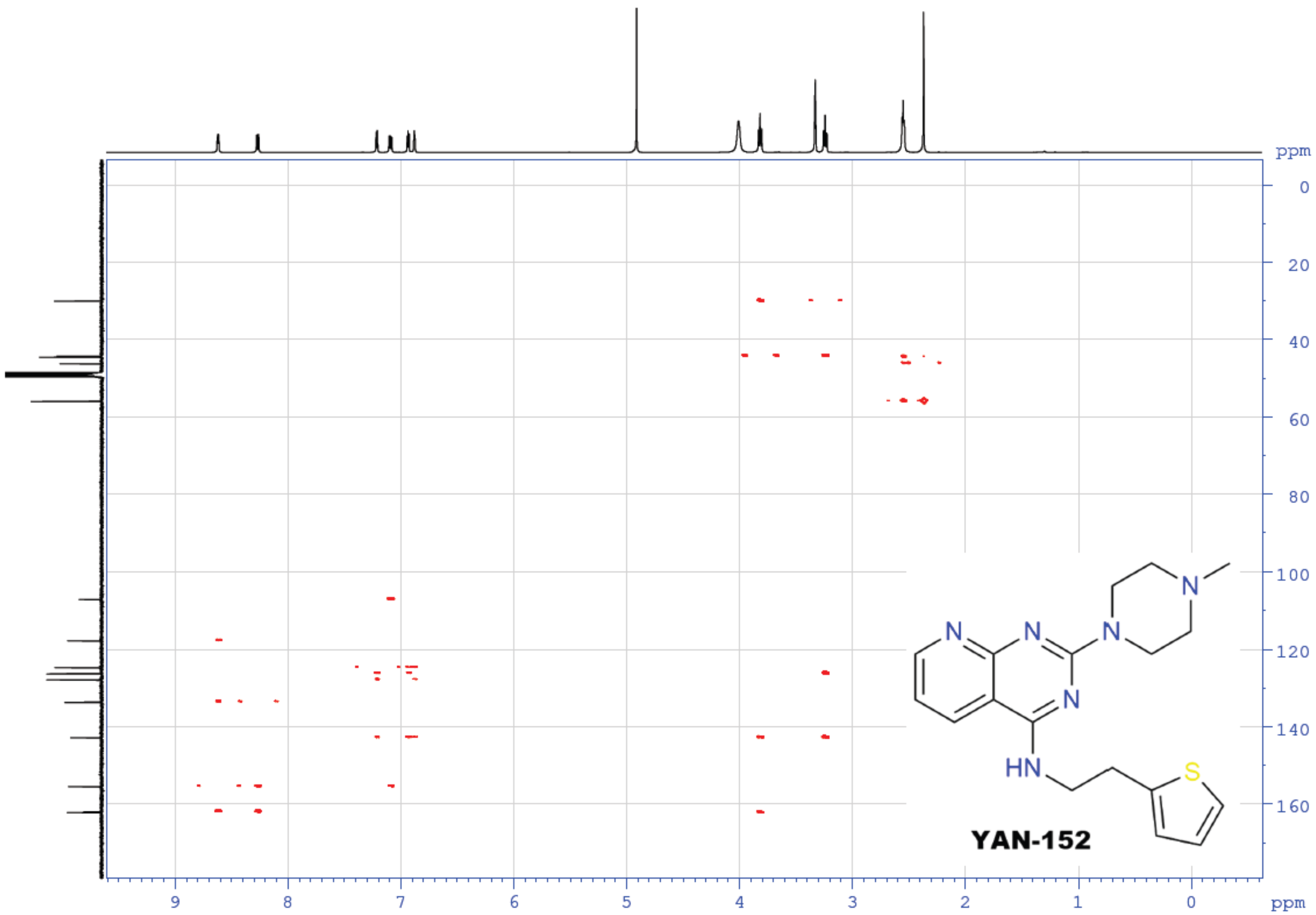

Figure S14. YAN-152 — ^{1}H→^{13}C HMBC NMR spectrum

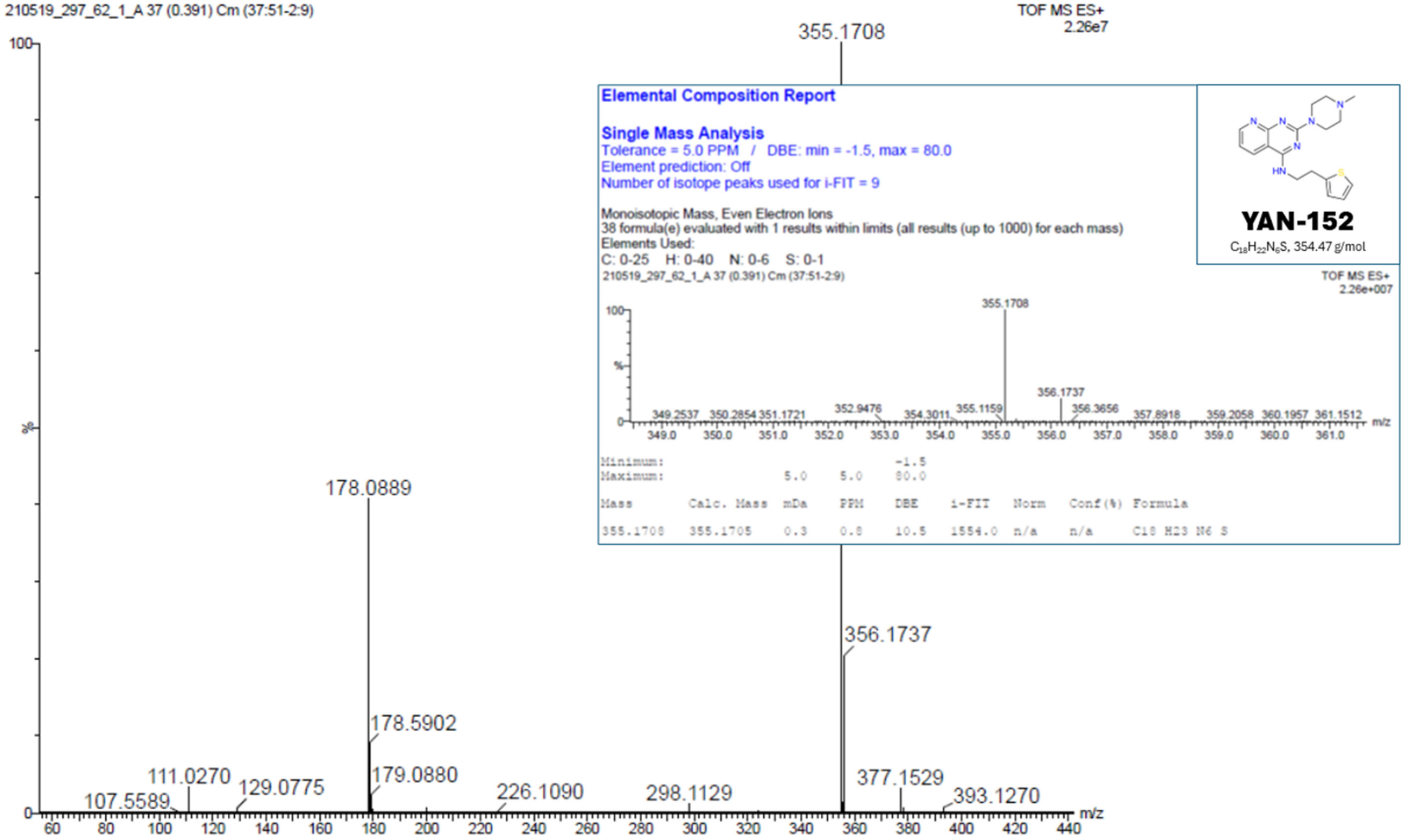

Figure S15. YAN-152 — HRMS (+) spectrum

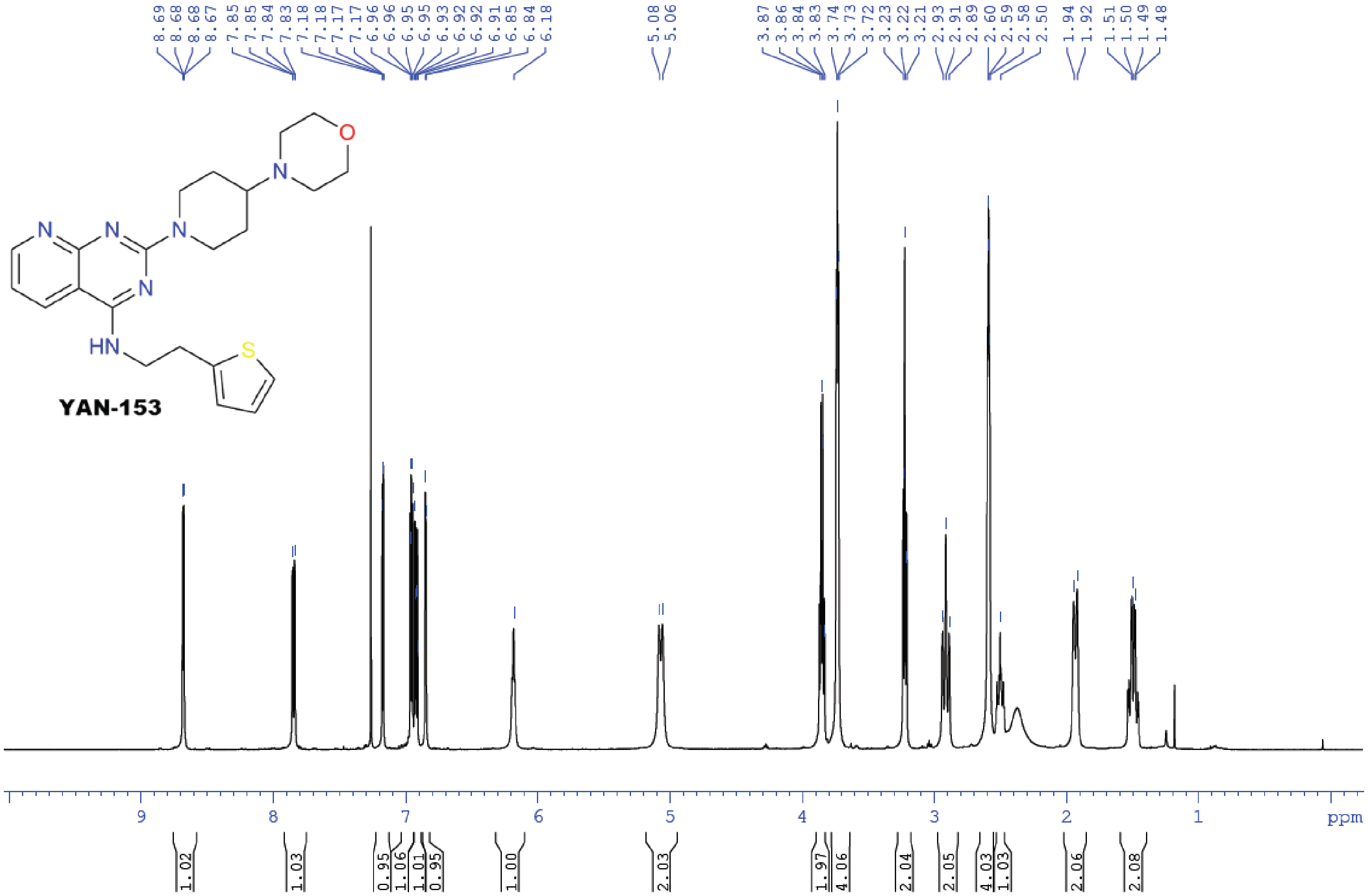

Figure S16. YAN-153 — ^{1}H NMR spectrum

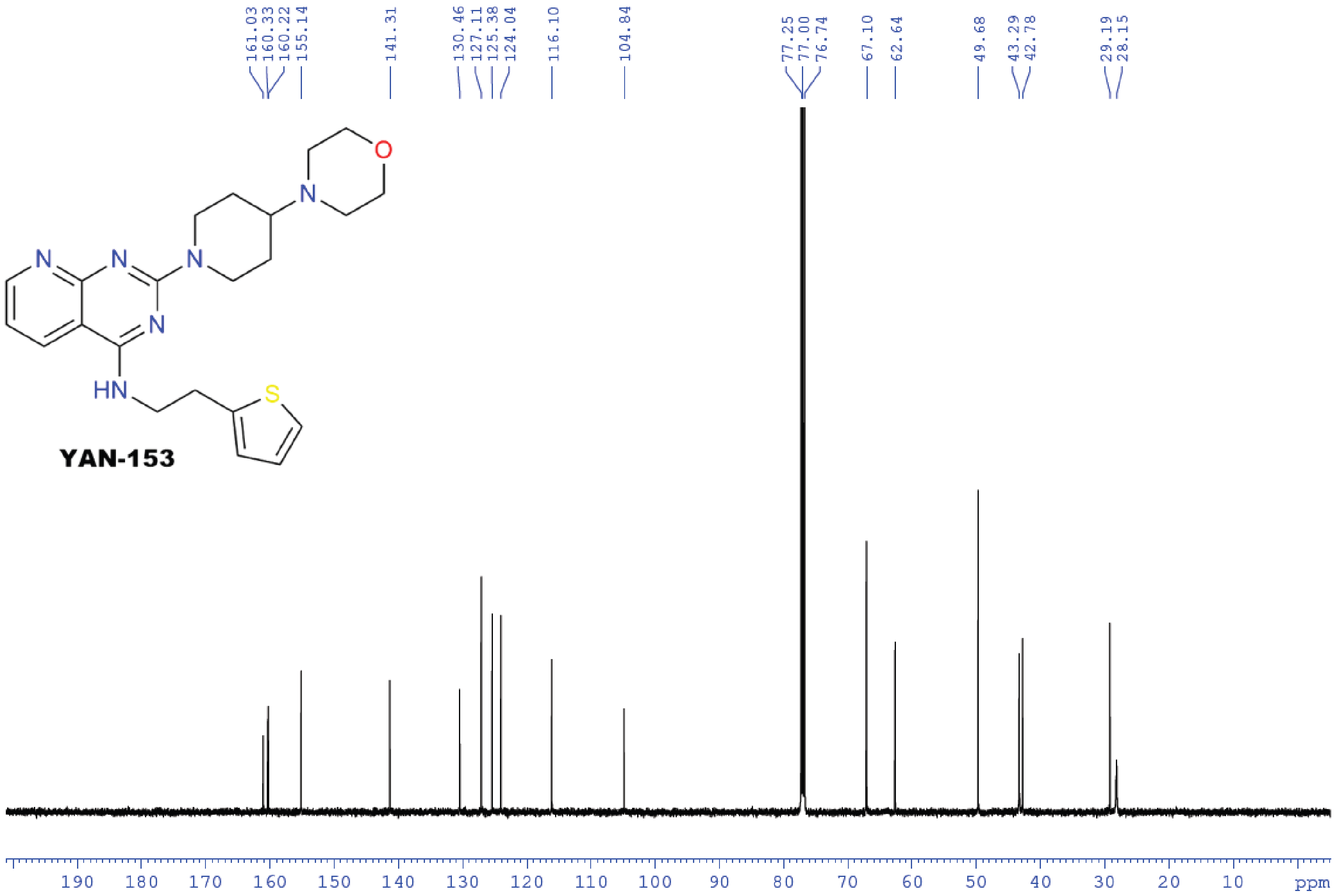

Figure S17. YAN-153 — ^{13}C NMR spectrum

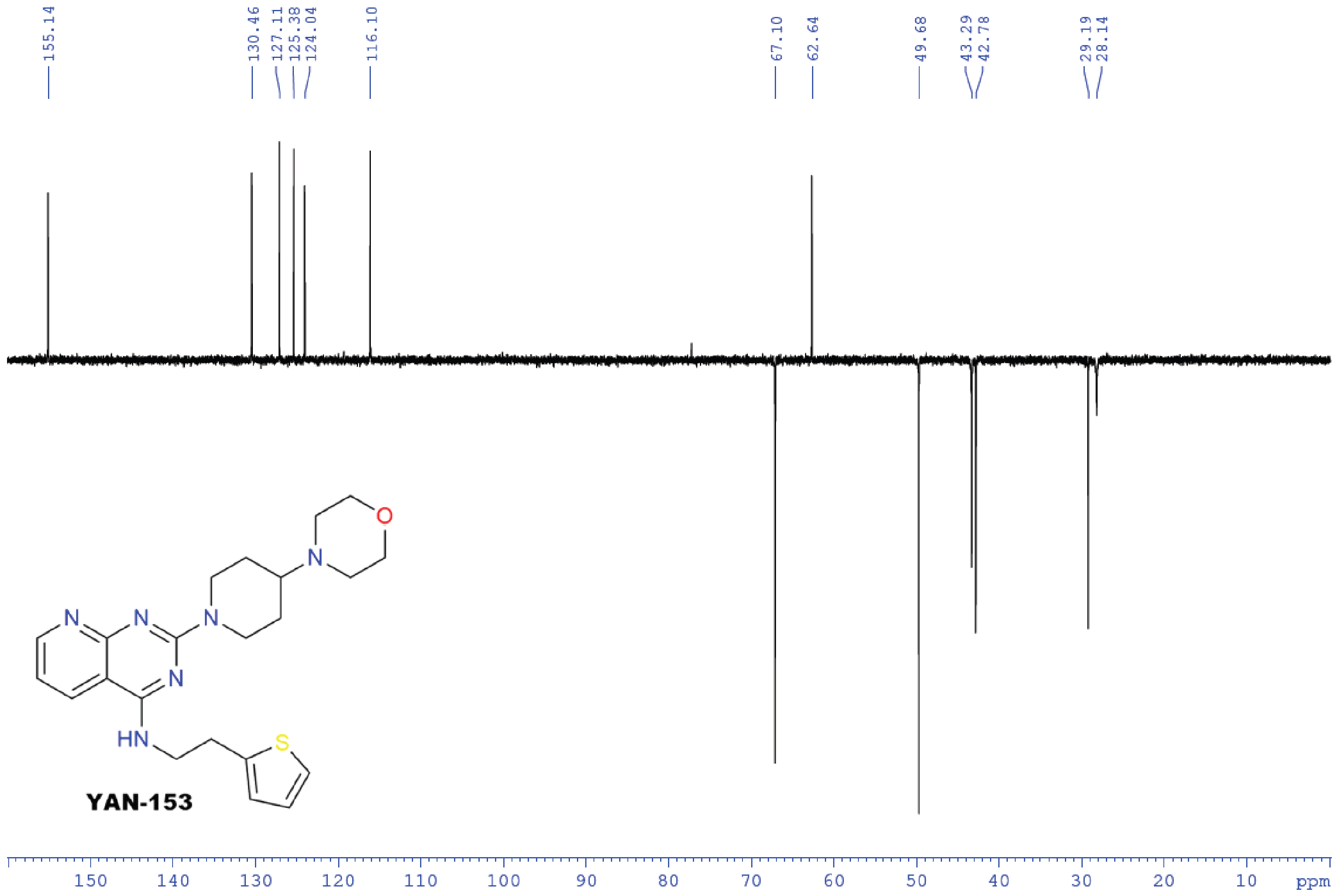

Figure S18. YAN-153 — DEPT NMR spectrum

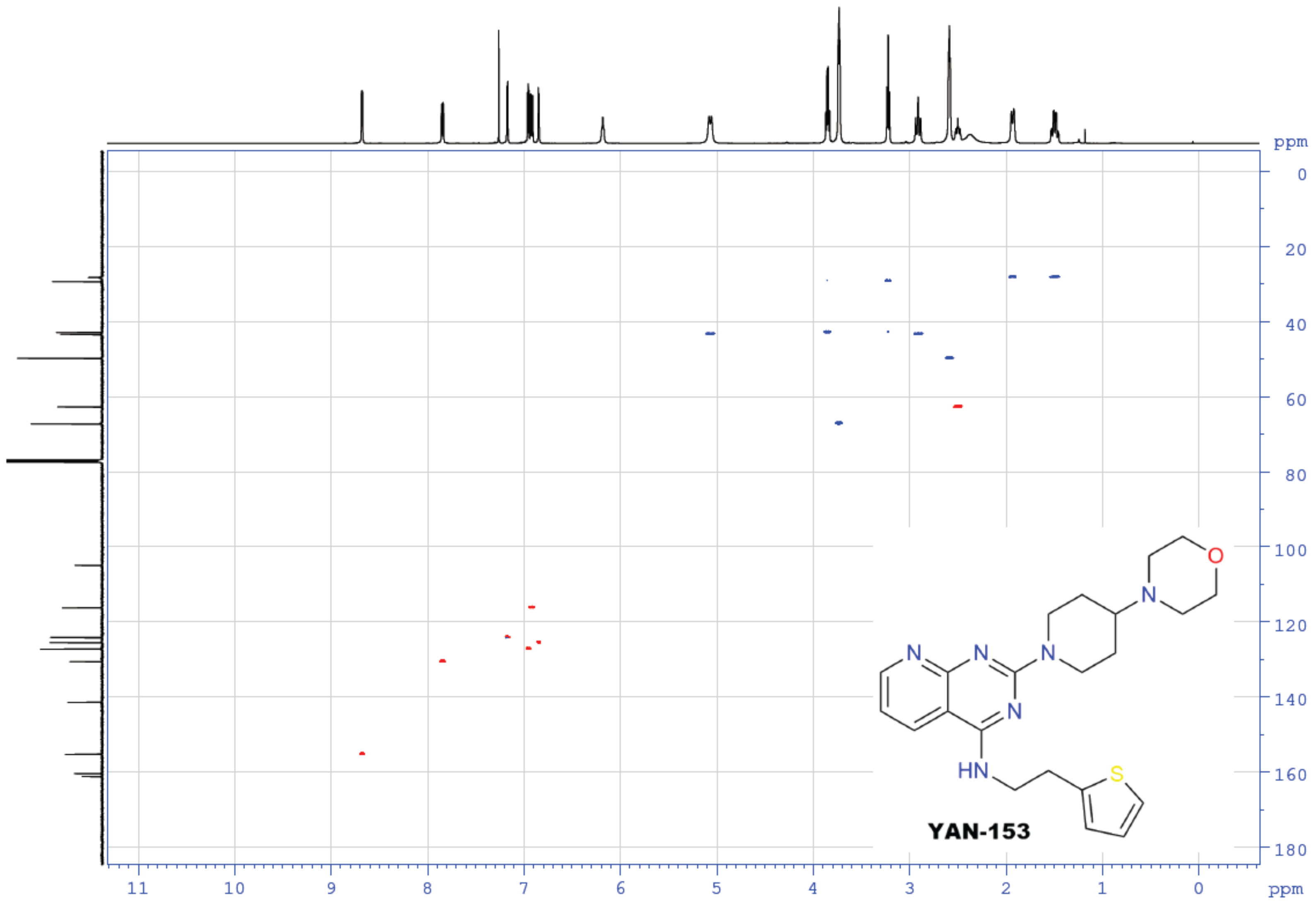

Figure S19. YAN-153 — ^{1}H-^{13}C HSQC spectrum

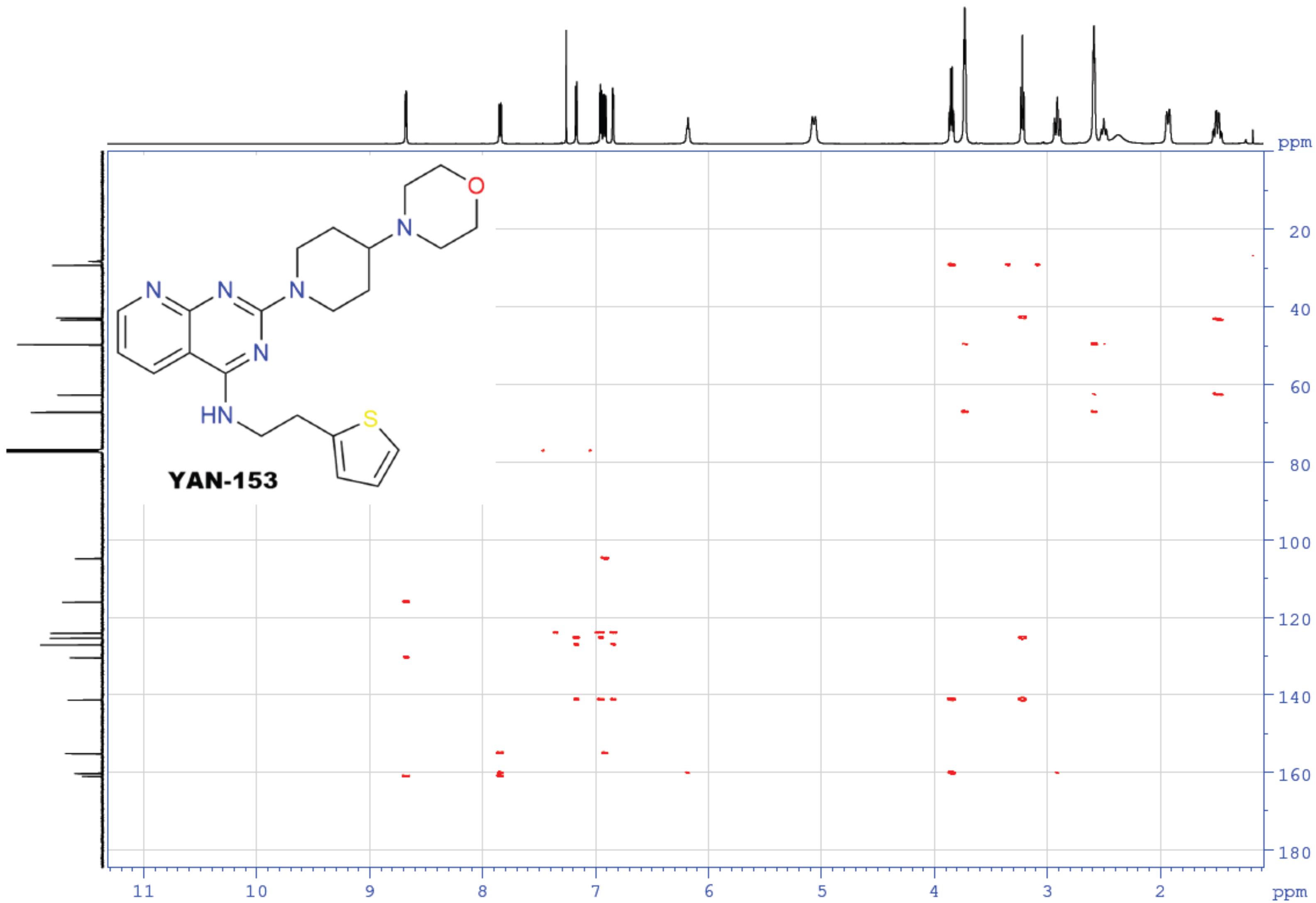

Figure S20. YAN-153 — $^{1}H \rightarrow ^{13}C$ HMBC NMR spectrum

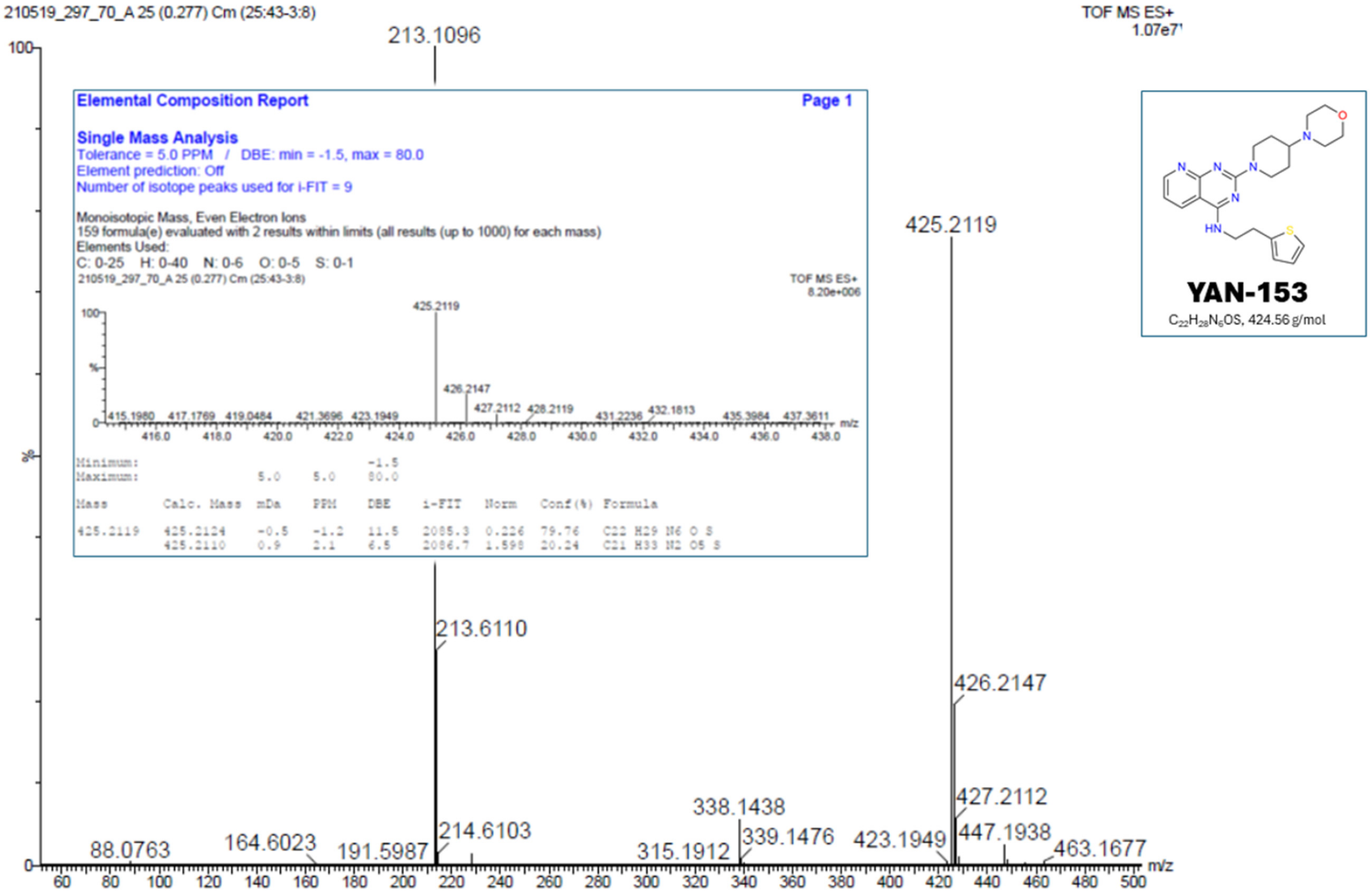

Figure S21. YAN-153 — HRMS (+) spectrum

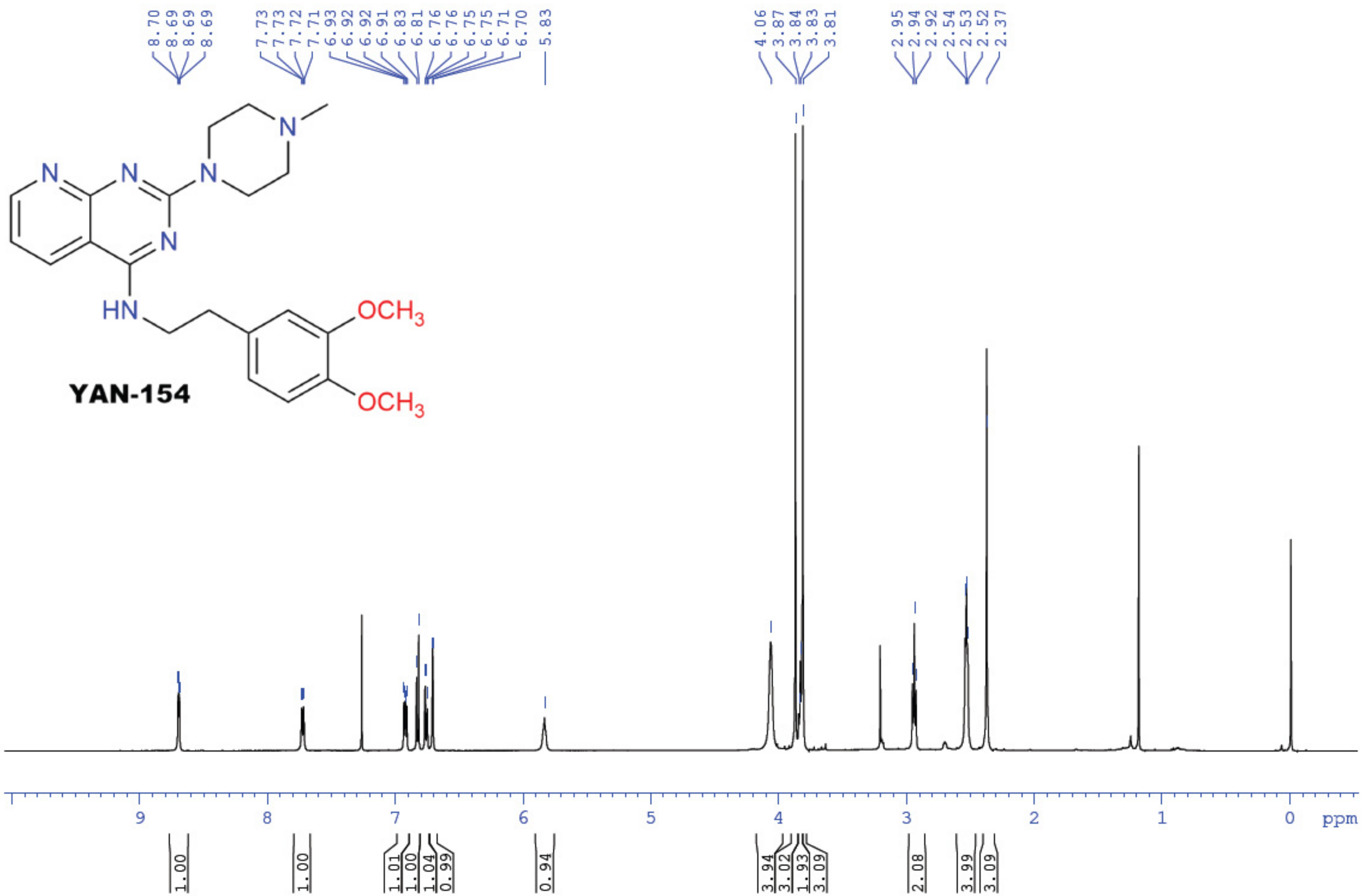

Figure S22. YAN-154 — ¹H NMR spectrum

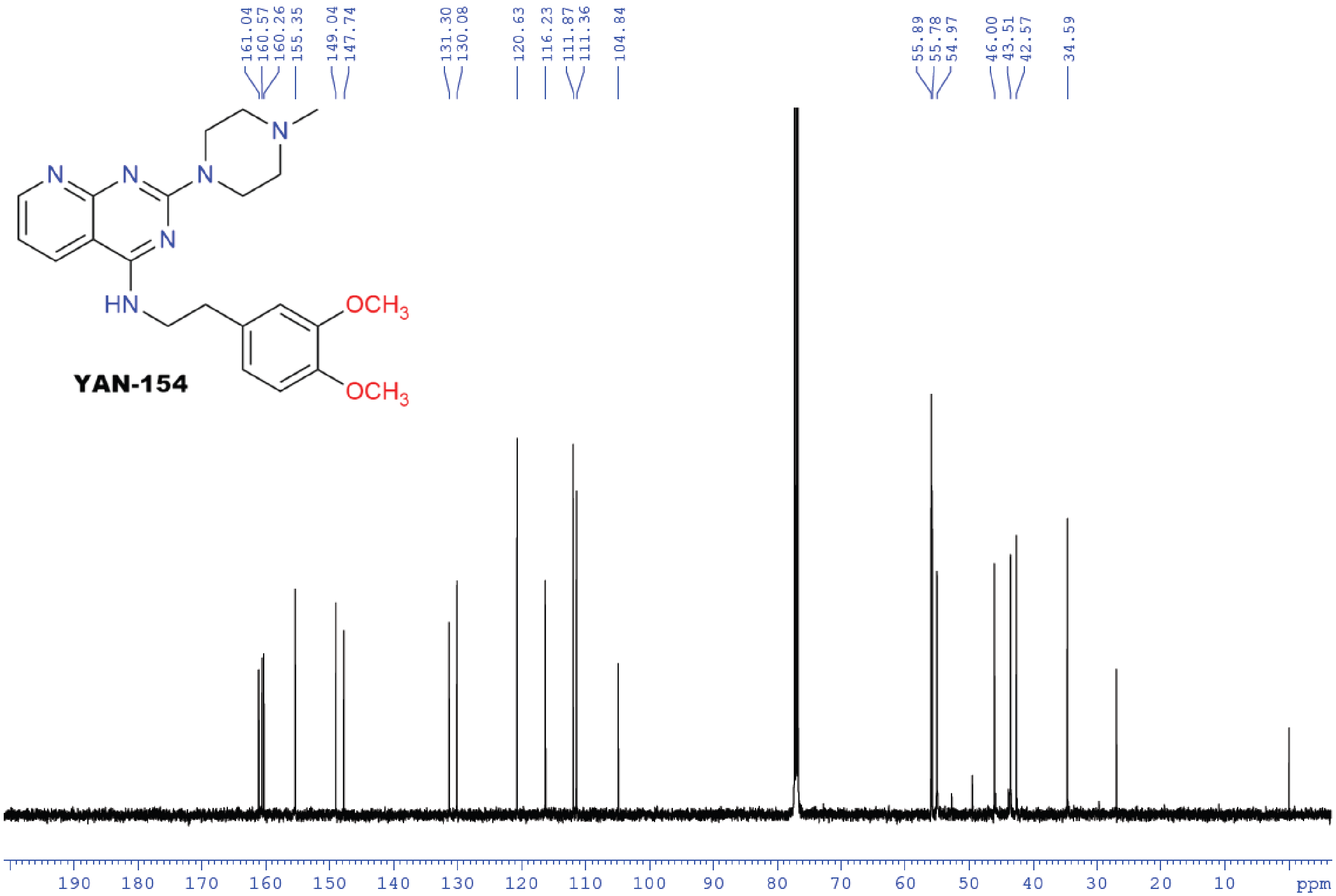

Figure S23. YAN-154 — ^{13}C NMR spectrum

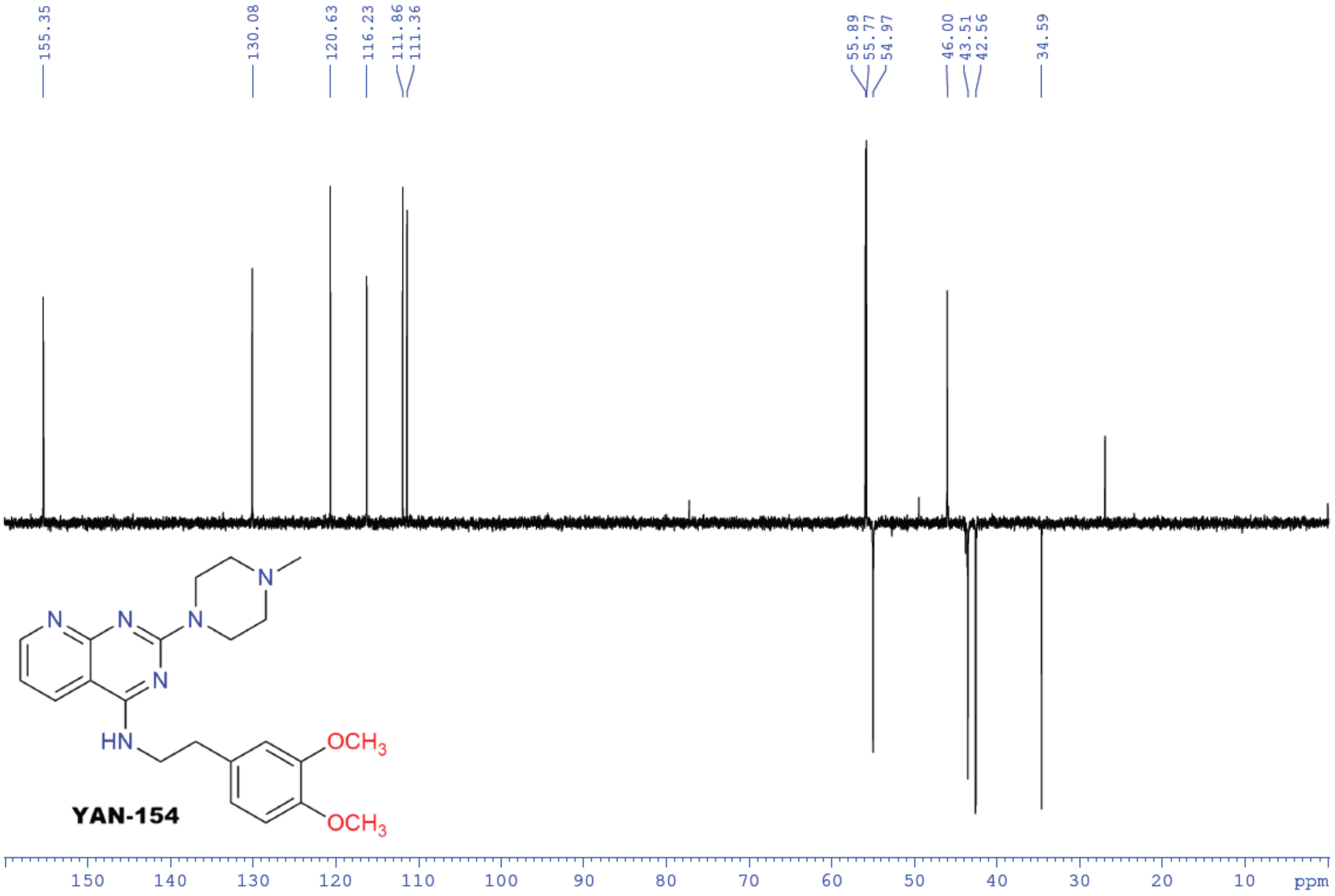

Figure S24. YAN-154 — DEPT NMR spectrum

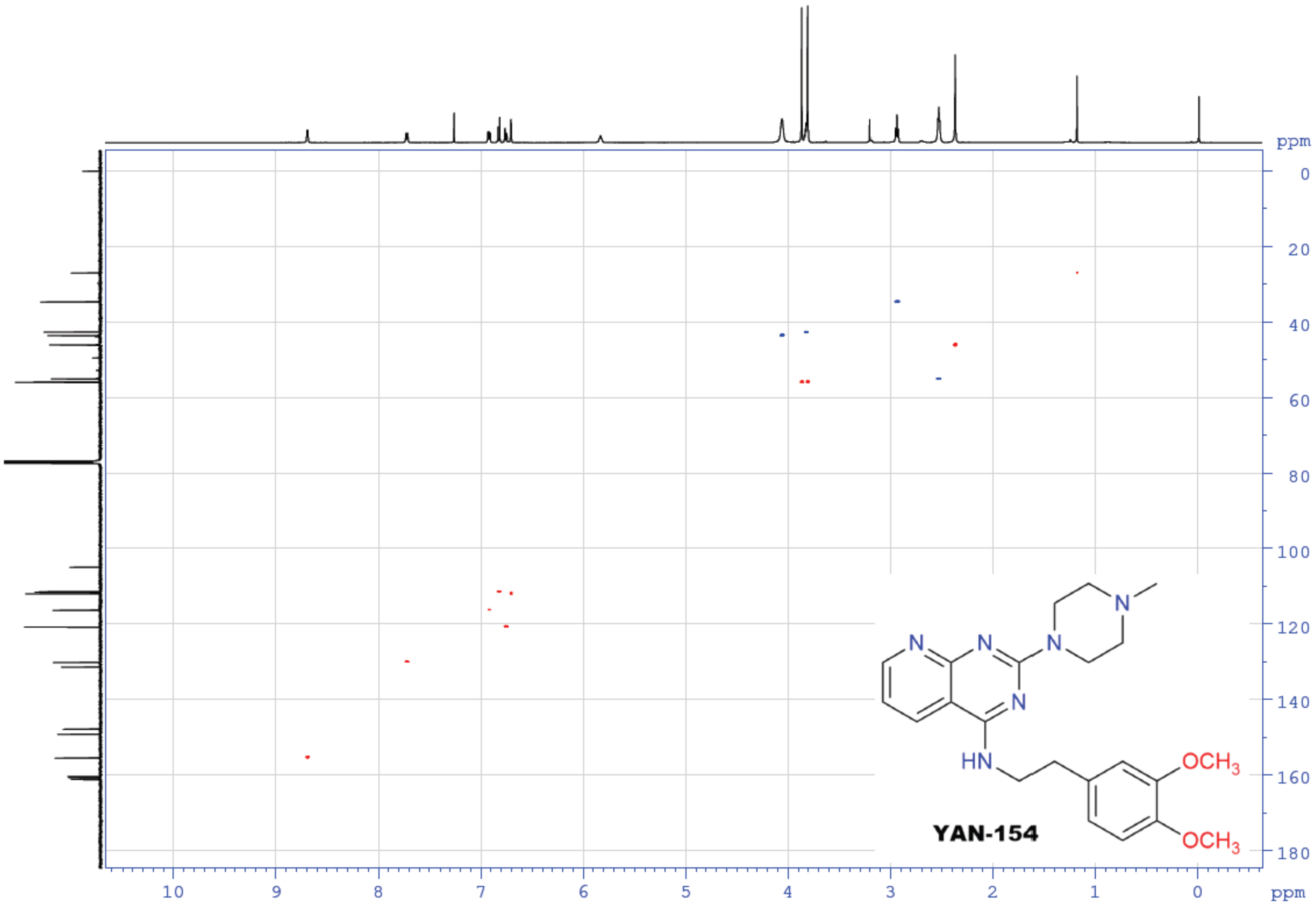

Figure S25. YAN-154 — ^{1}H-^{13}C HSQC NMR spectrum

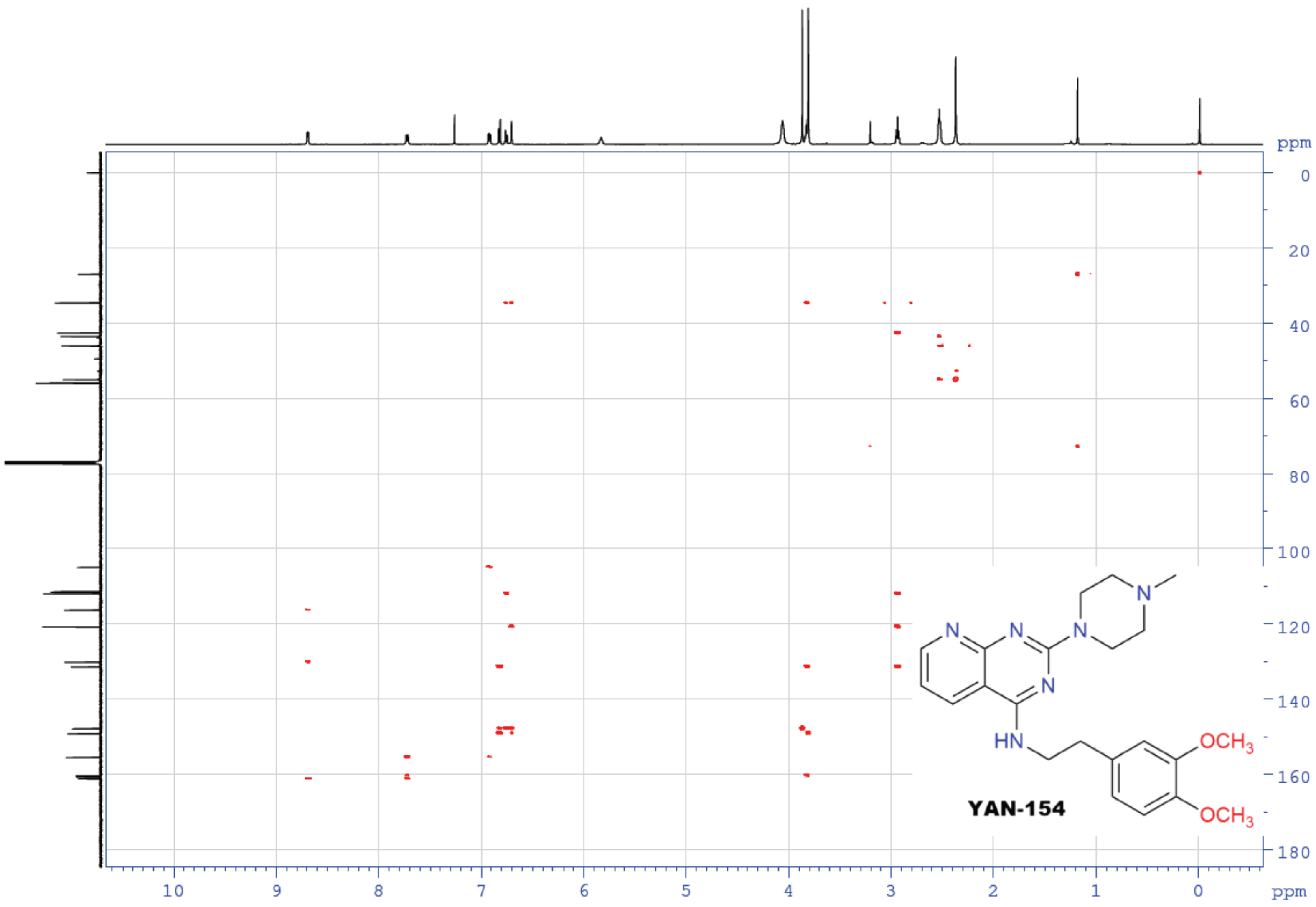

Figure S26. YAN-154 — $^{1}H \rightarrow ^{13}C$ HMBC NMR spectrum

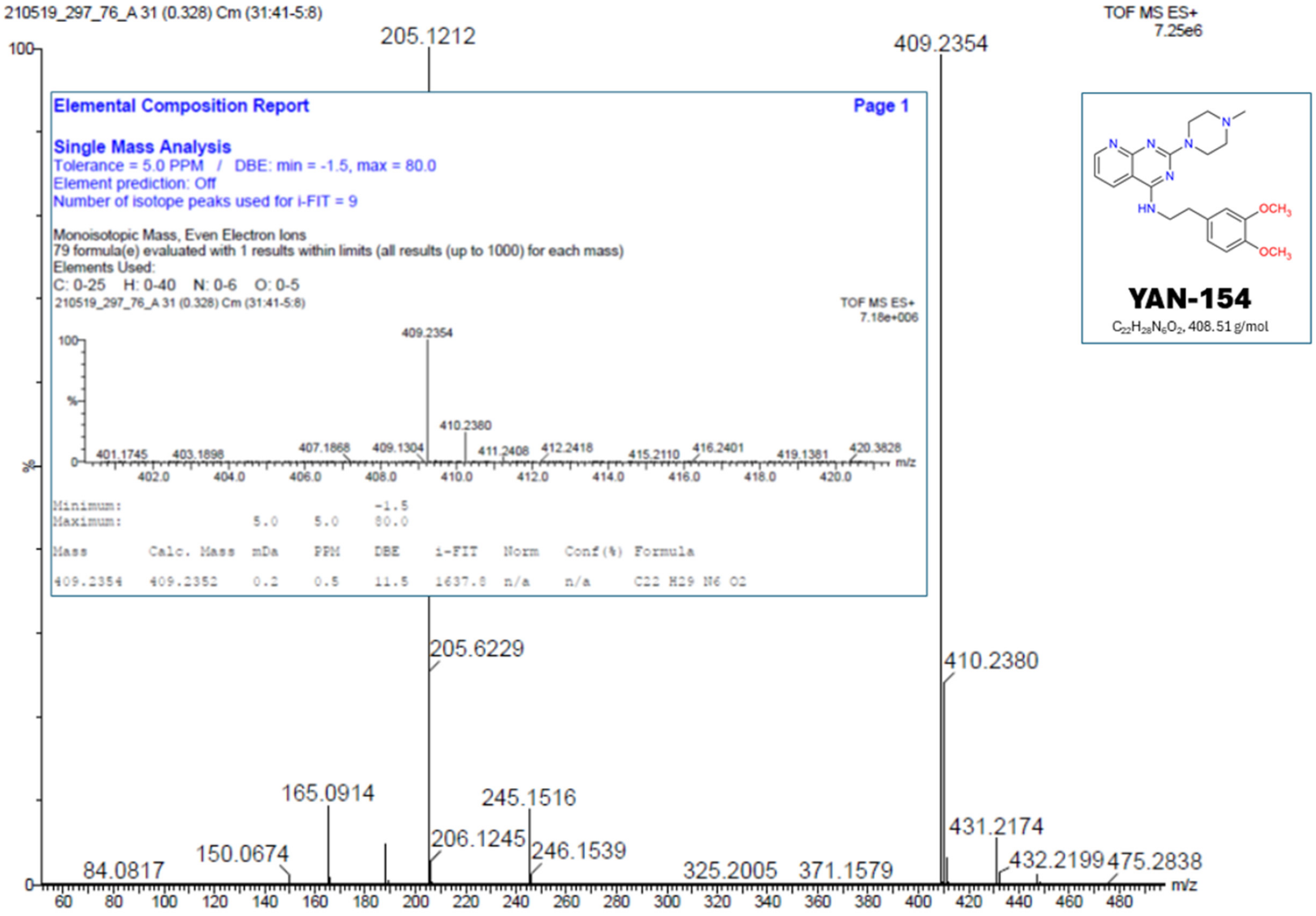

Figure S27. YAN-154 — HRMS (+) spectrum

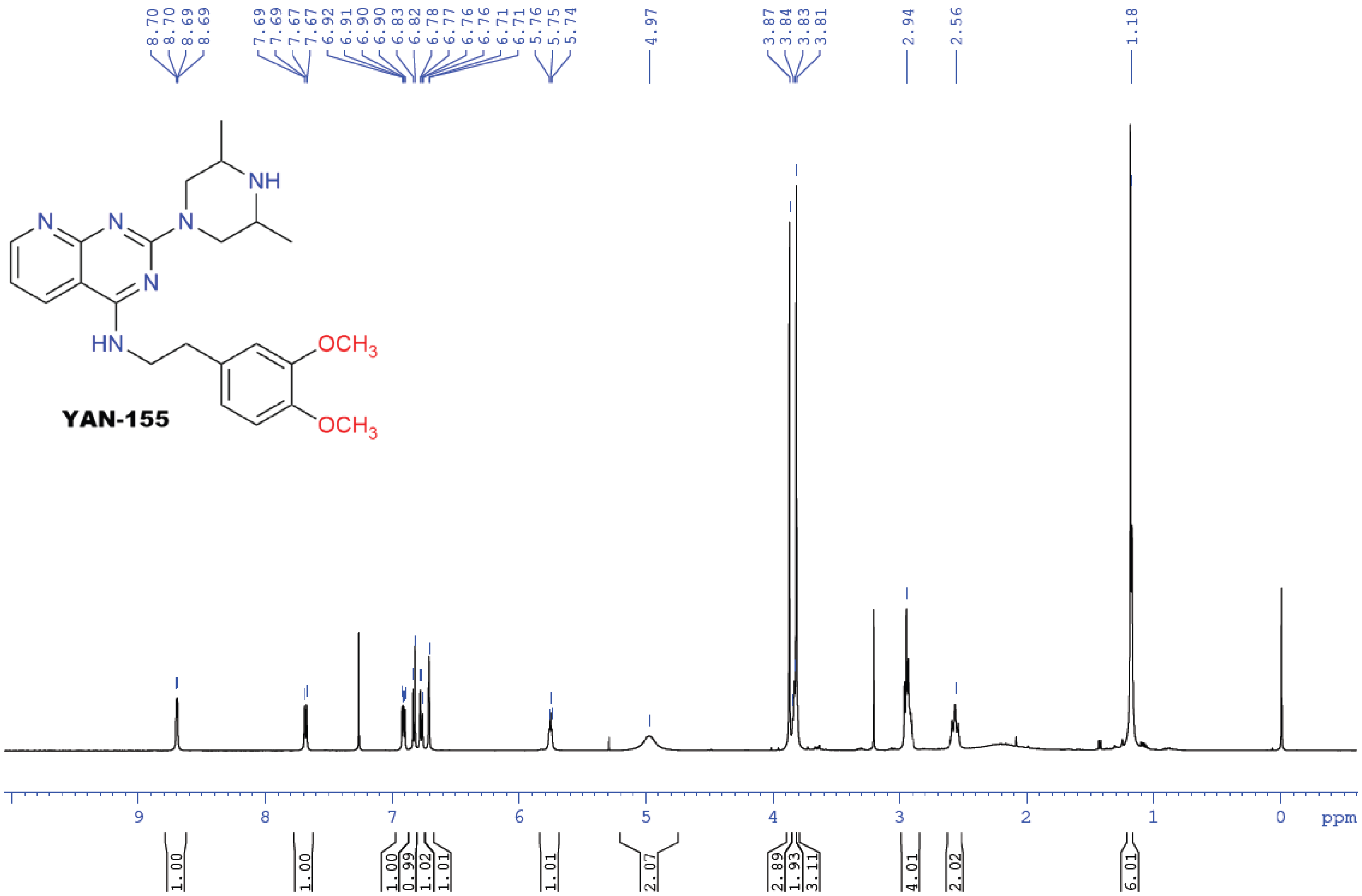

Figure S28. YAN-155 — ¹H NMR spectrum

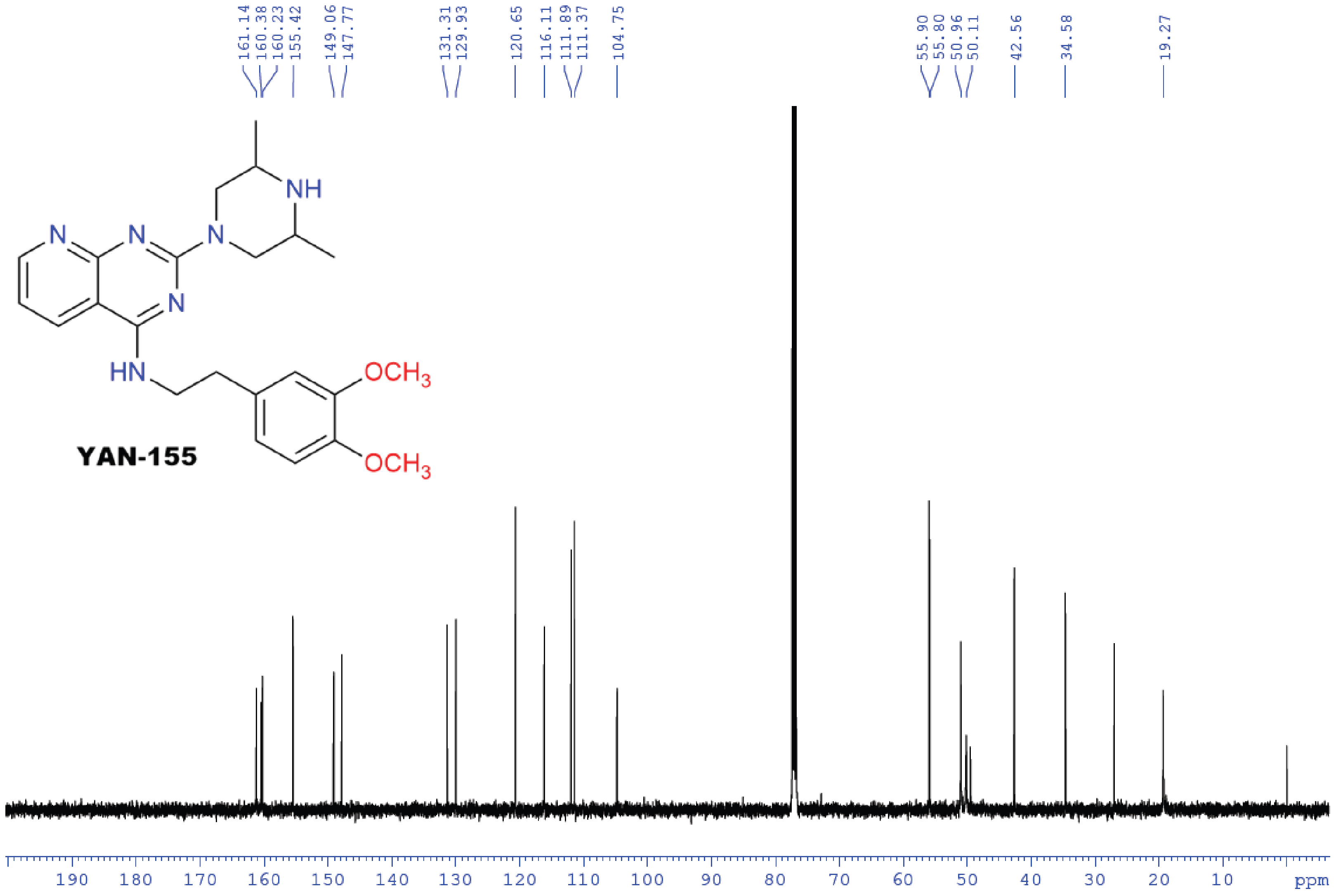

Figure S29. YAN-155 — ^{13}C NMR spectrum

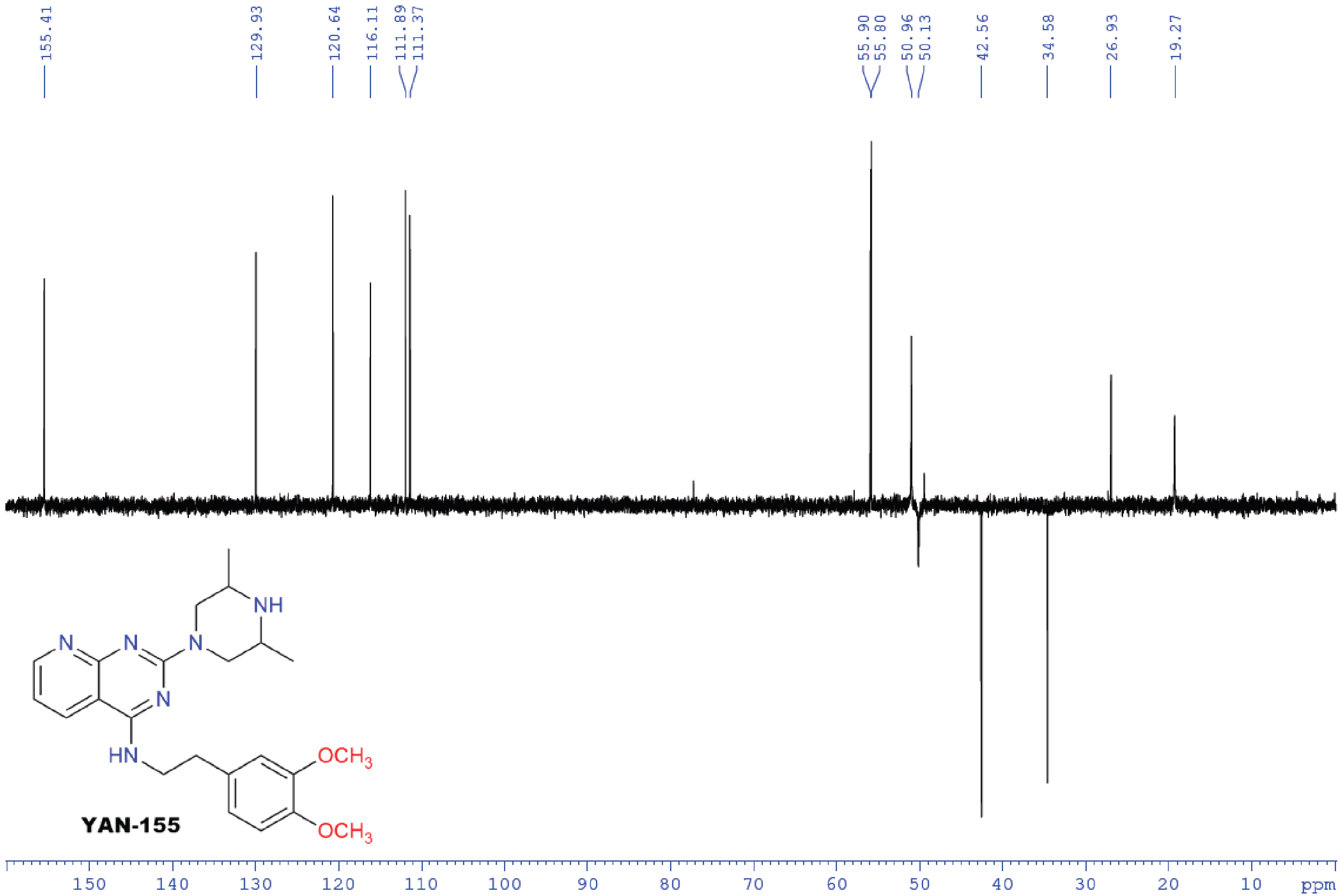

Figure S30. YAN-155 — DEPT NMR spectrum

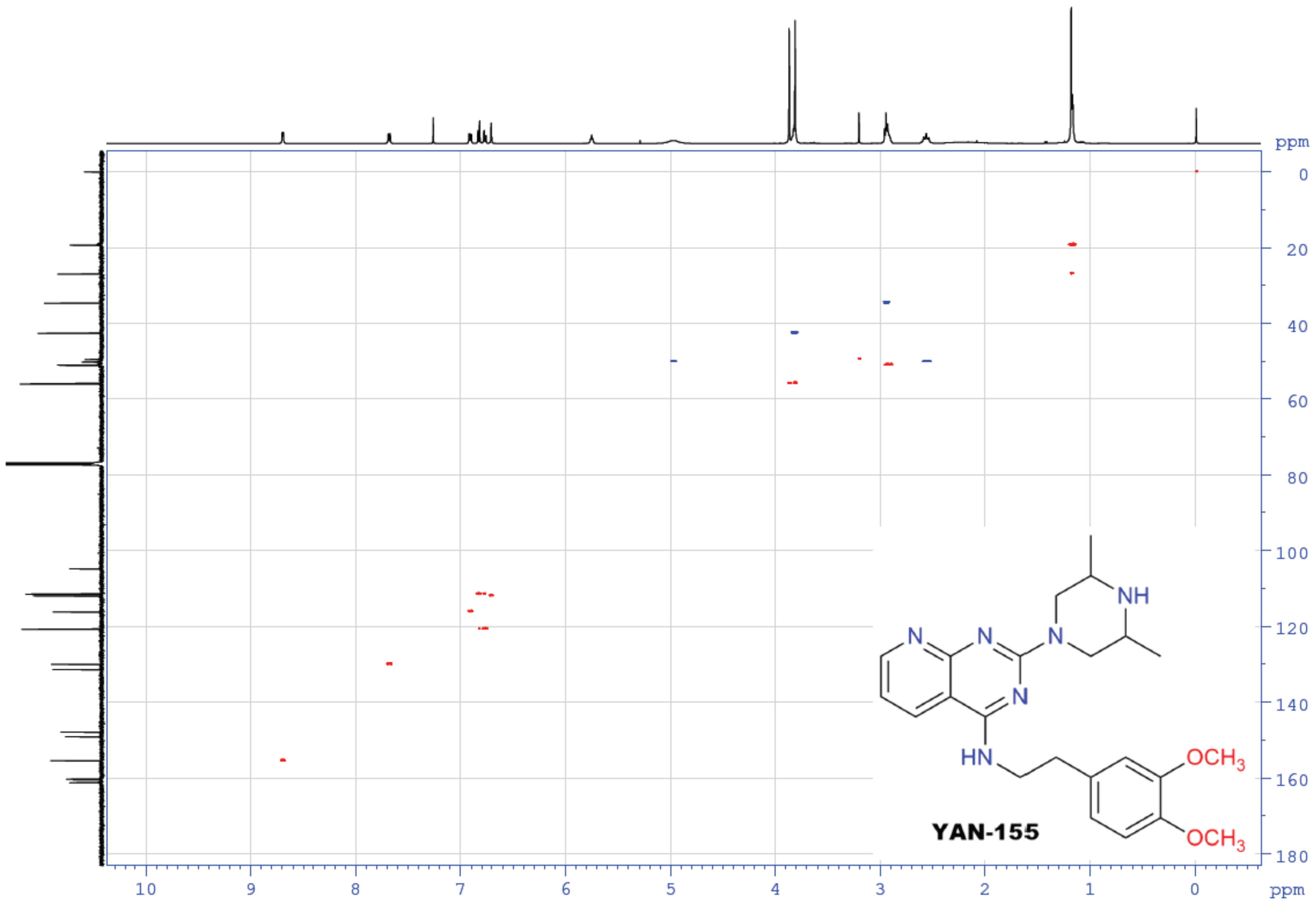

Figure S31. YAN-155 — ^{1}H-^{13}C HSQC NMR spectrum

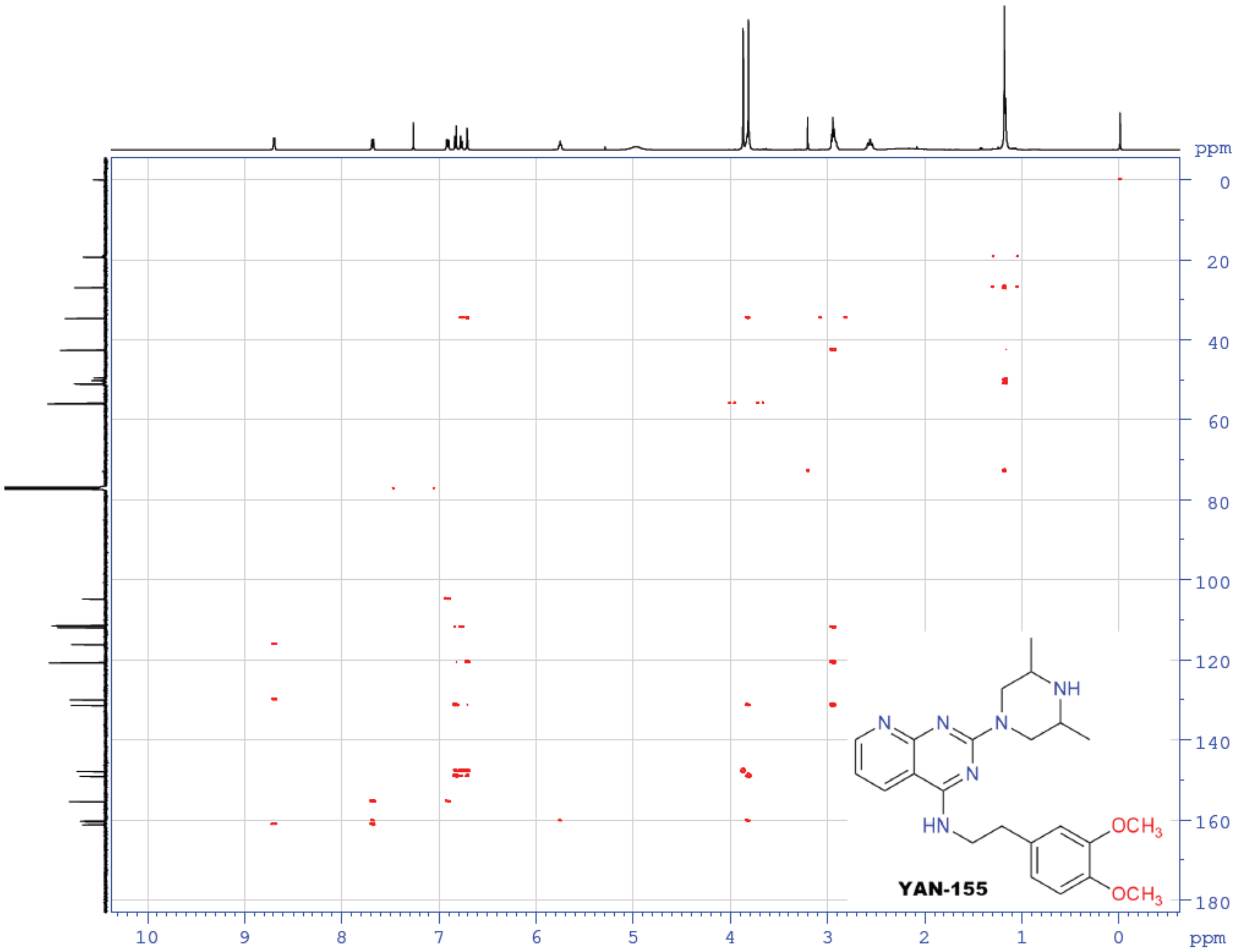

Figure S32. YAN-155 — ^{1}H→^{13}C HMBC NMR spectrum

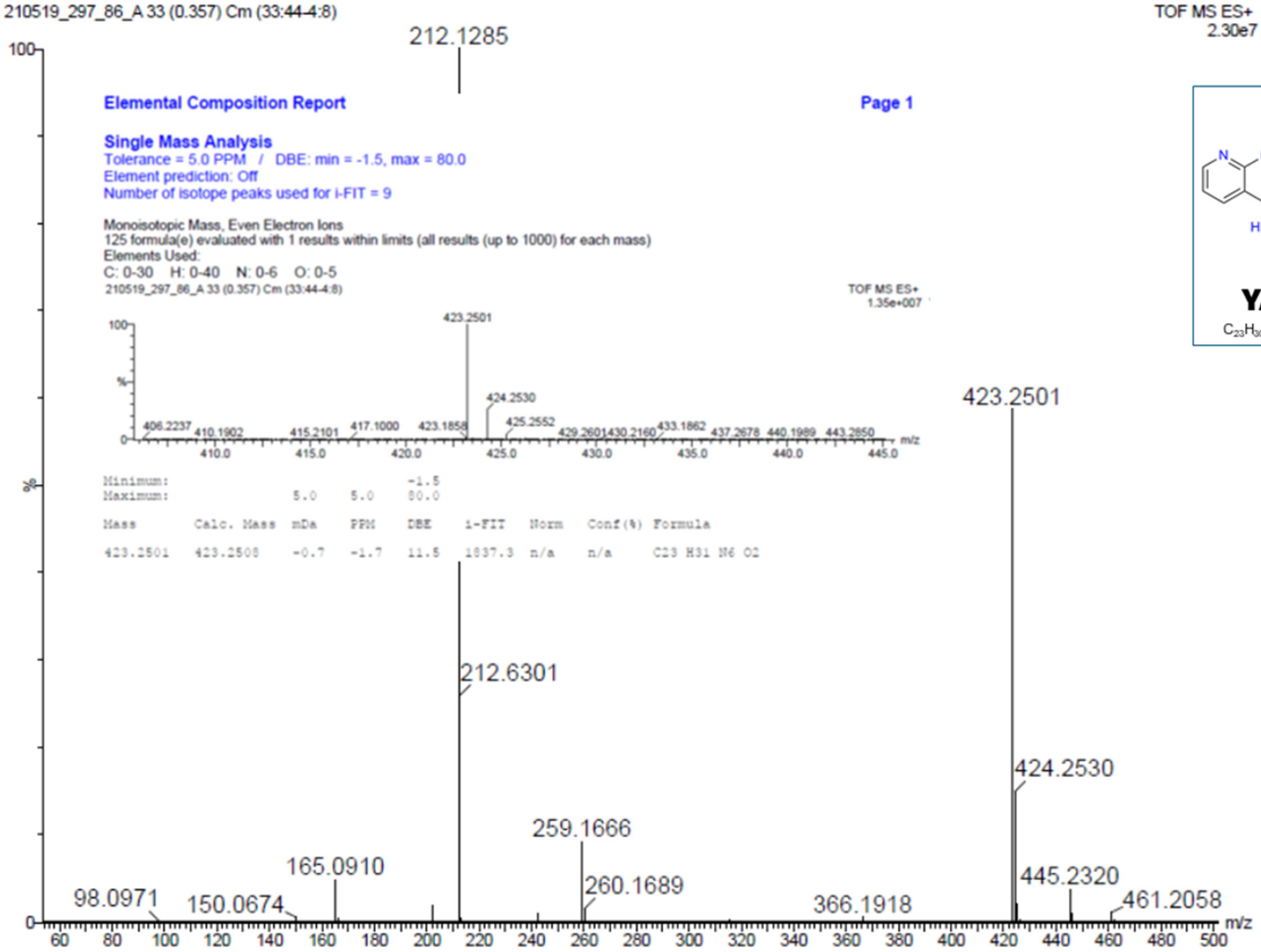

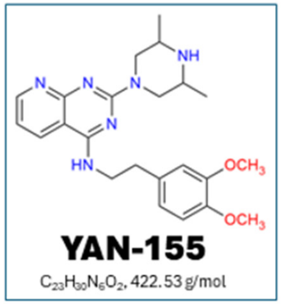

Figure S33. YAN-155 — HRMS (+) spectrum

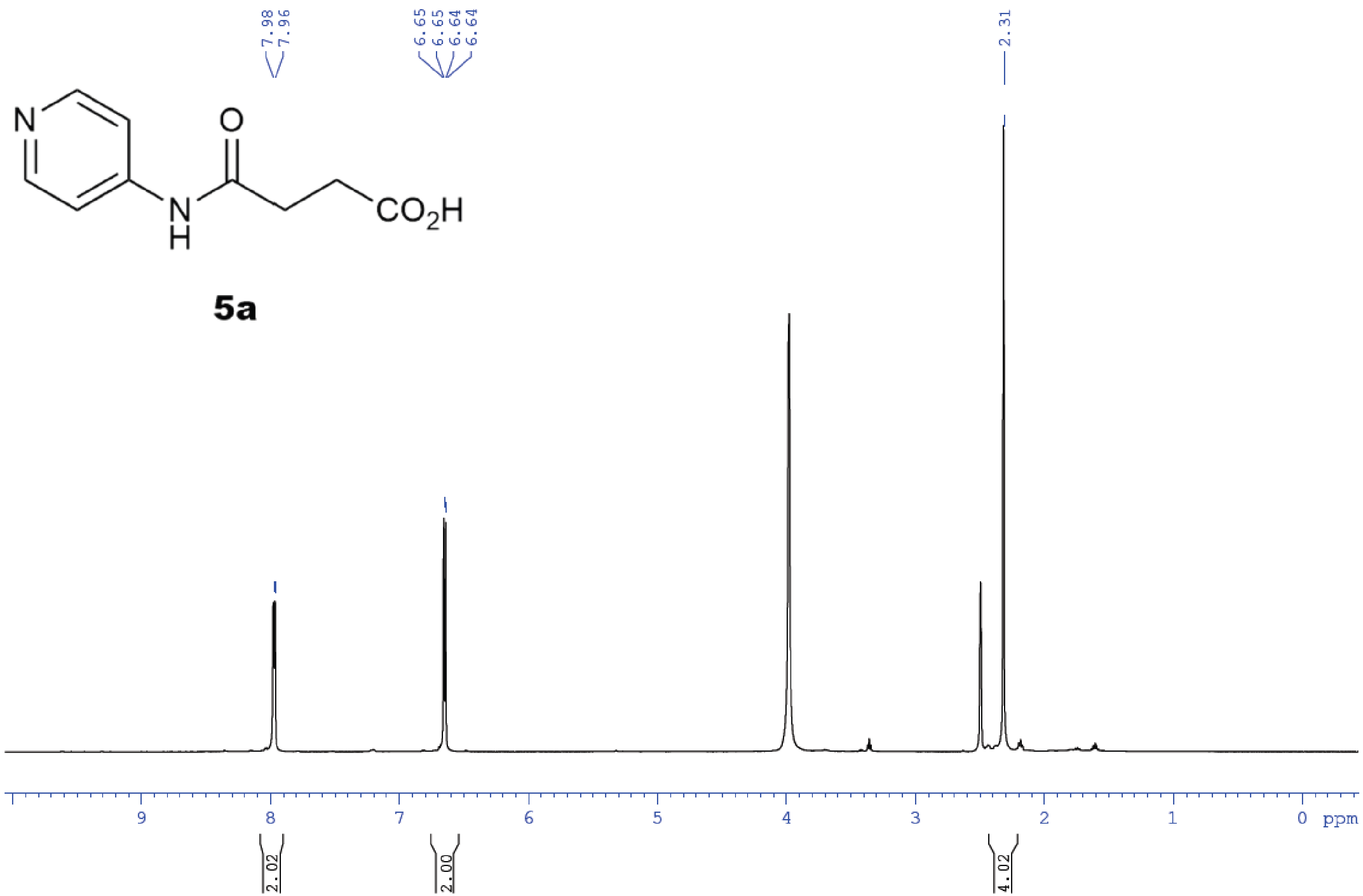

Figure S34. 5a — ^{1}H NMR spectrum

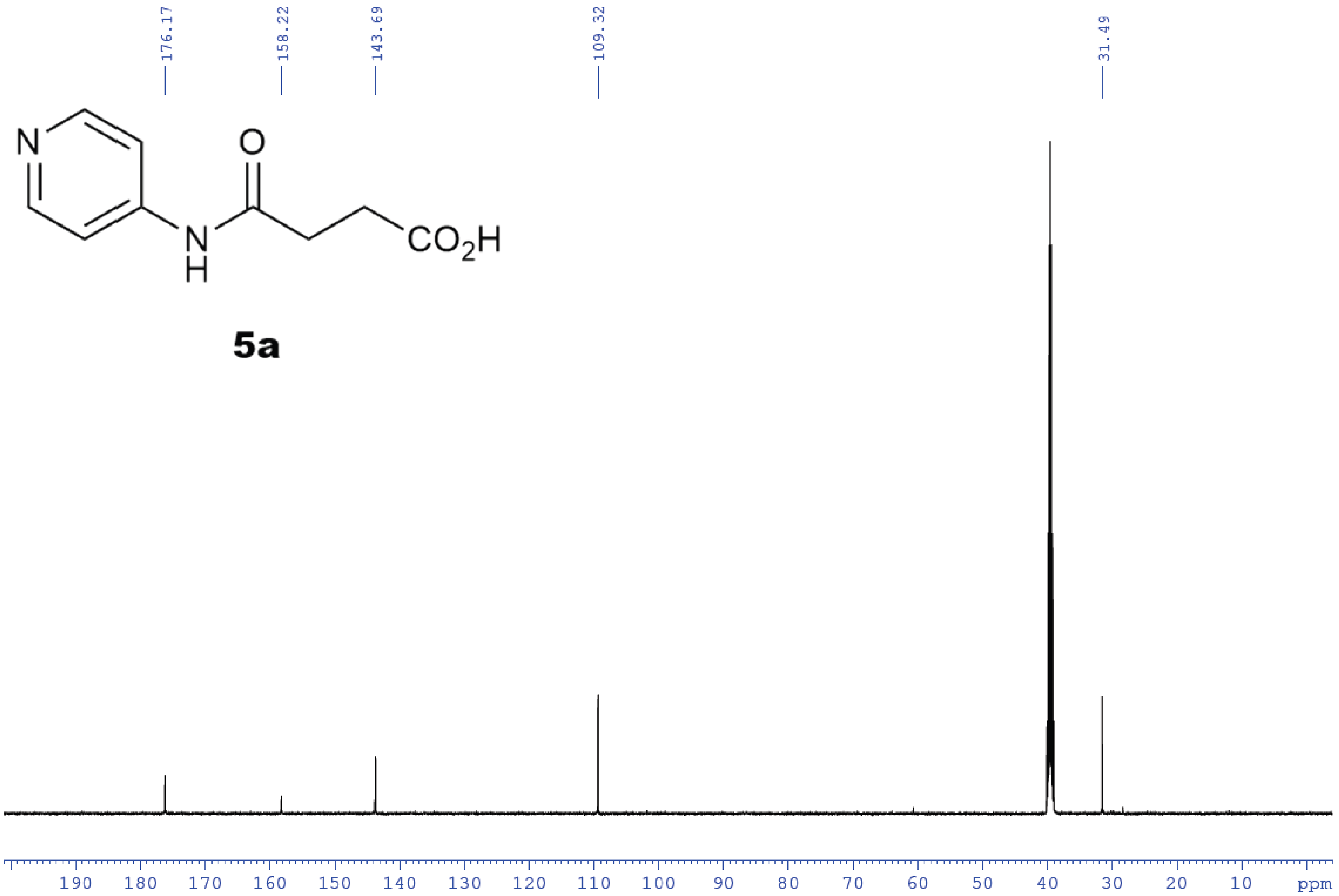

Figure S35. 5a — ^{13}C NMR spectrum

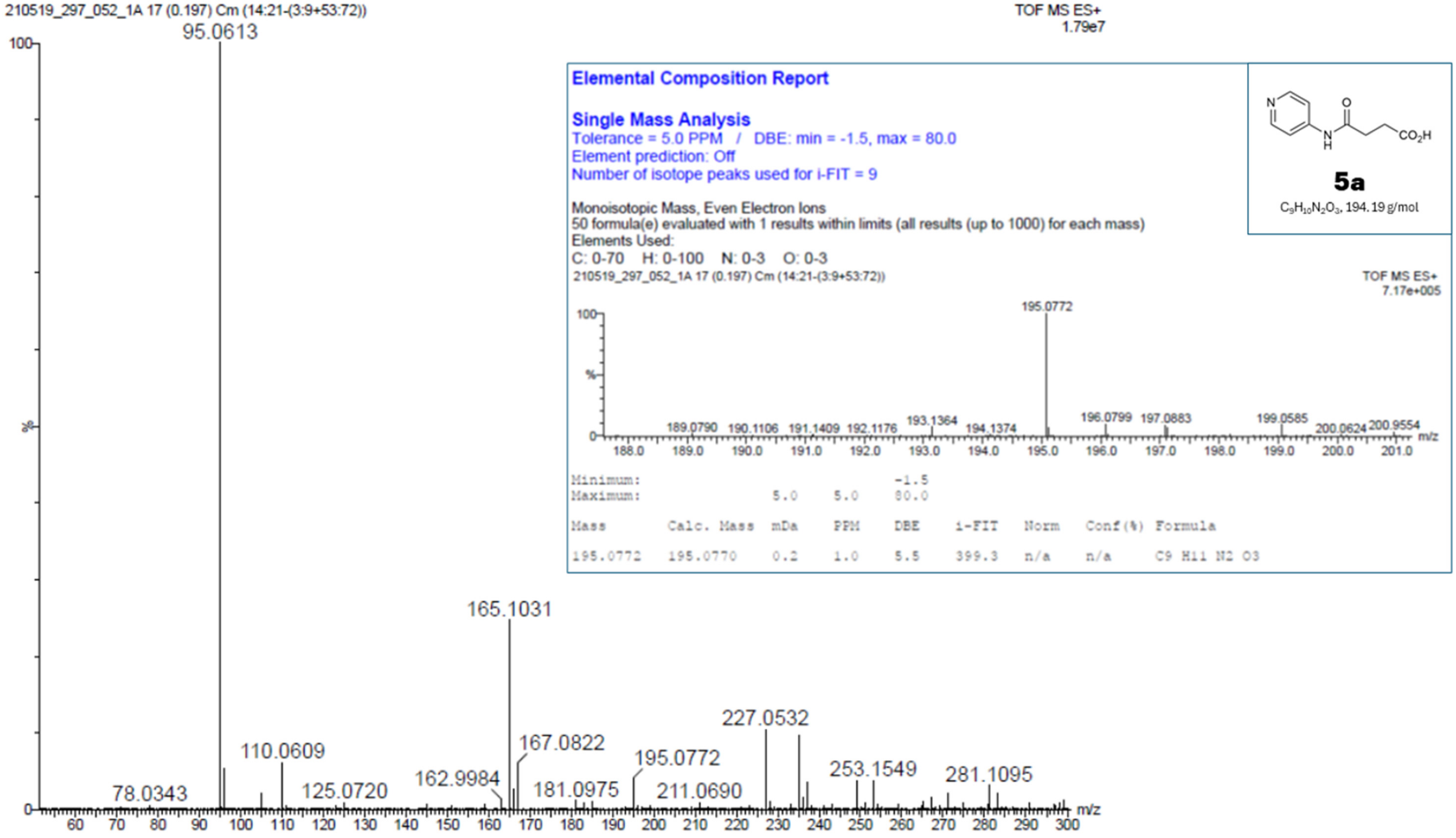

Figure S36. 5a — HRMS (+) spectrum

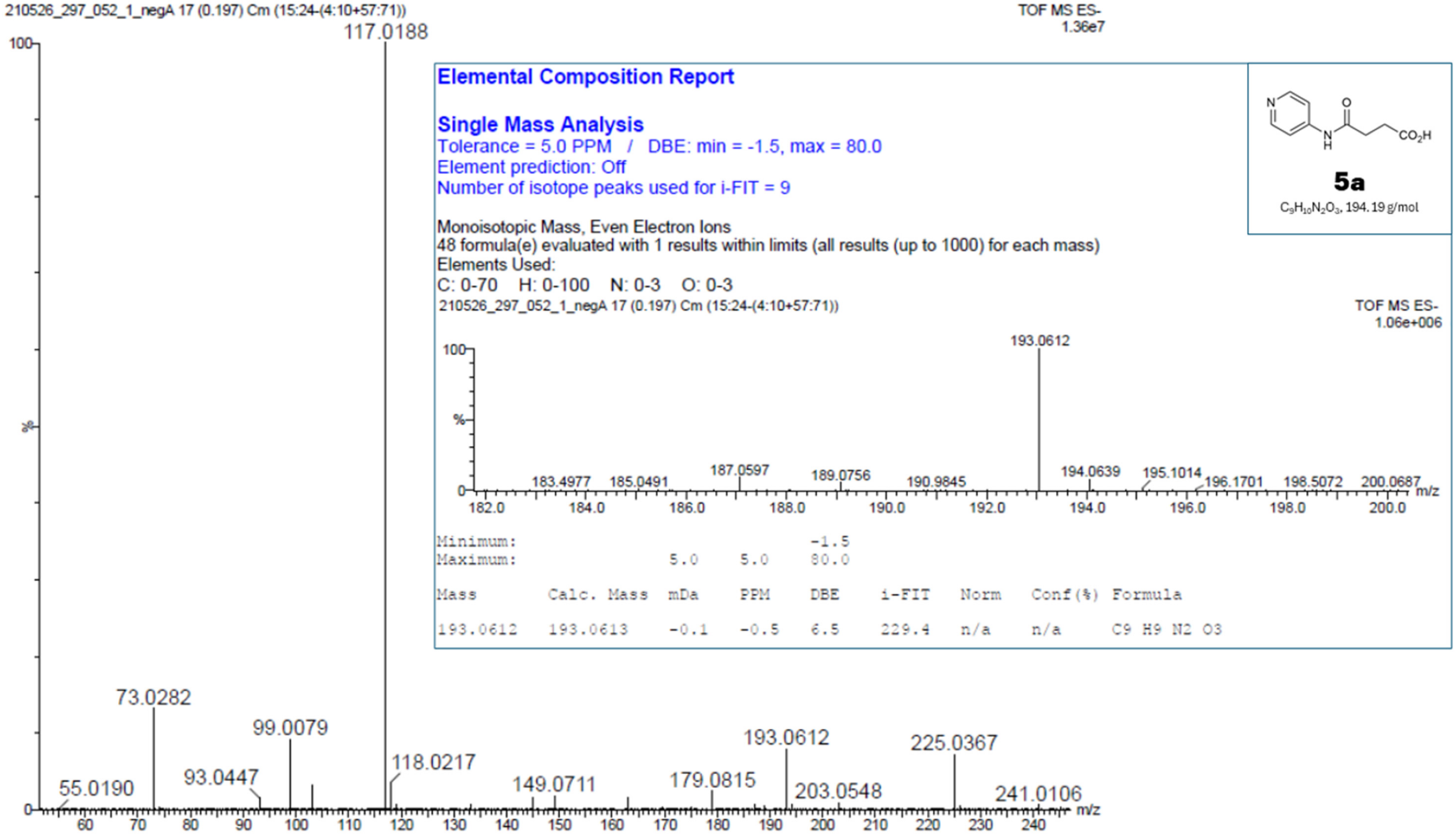

Figure S37. 5a — HRMS (–) spectrum

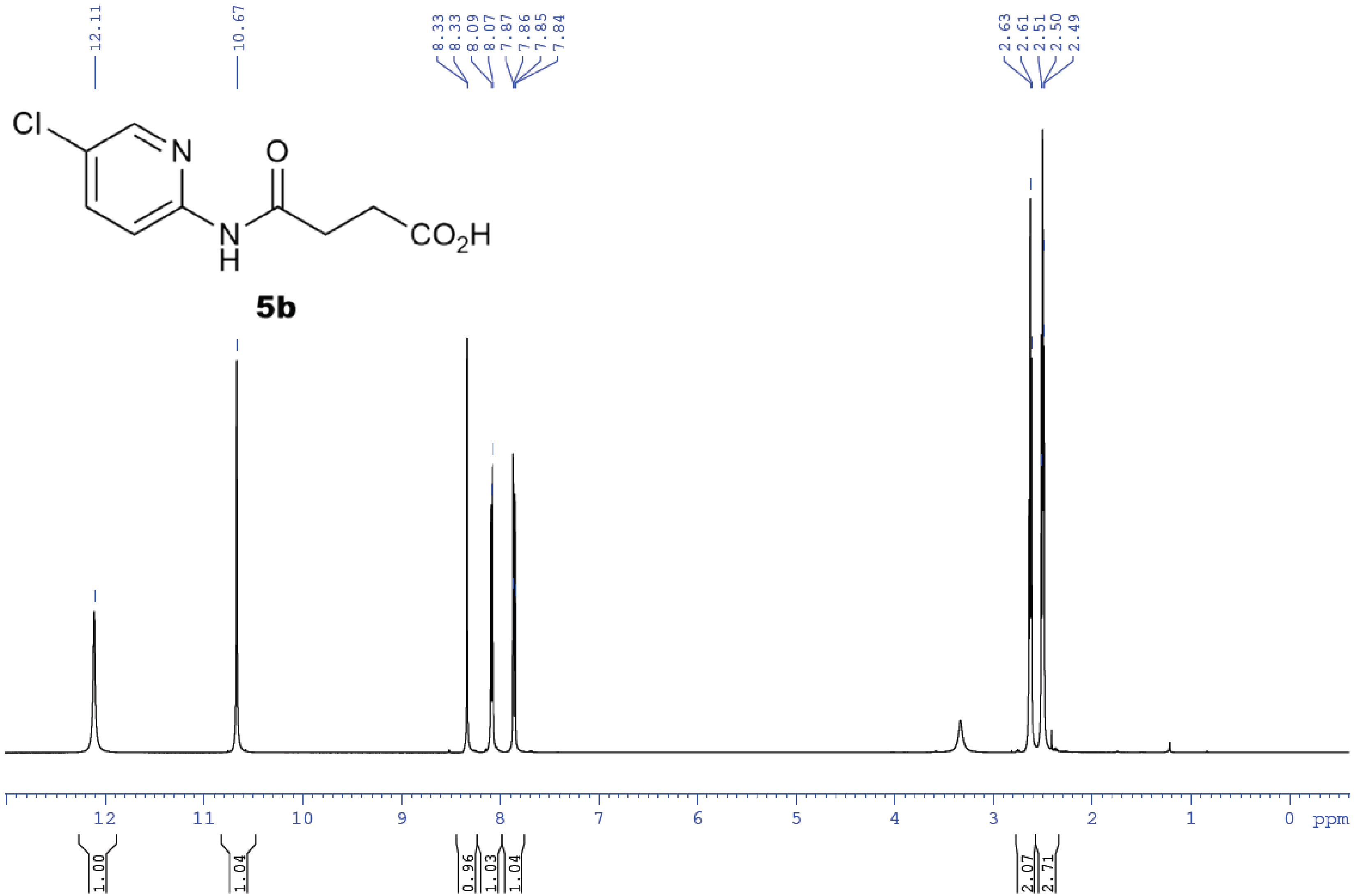

Figure S38. 5b — ^{1}H NMR spectrum

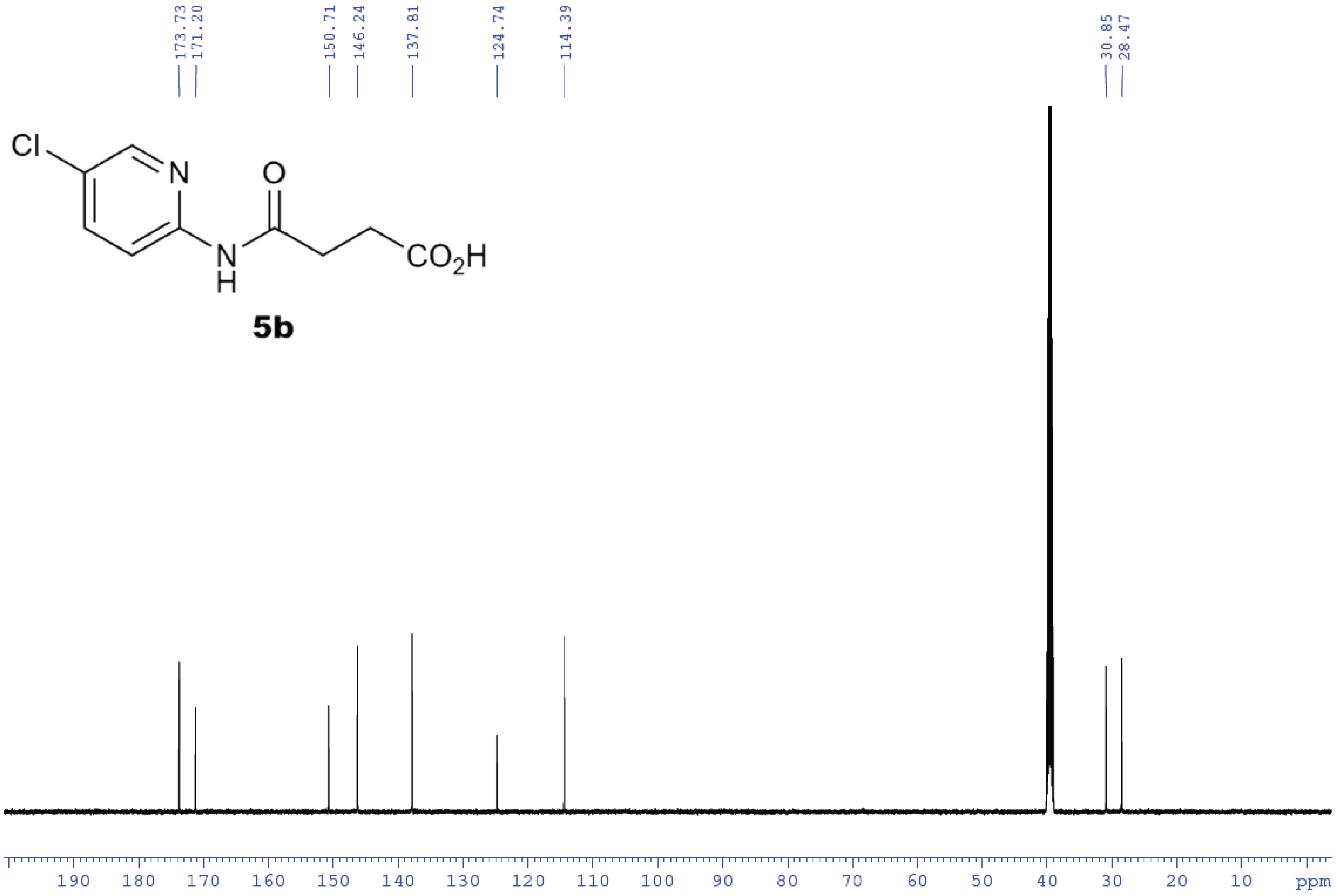

Figure S39. 5b — ^{13}C NMR spectrum

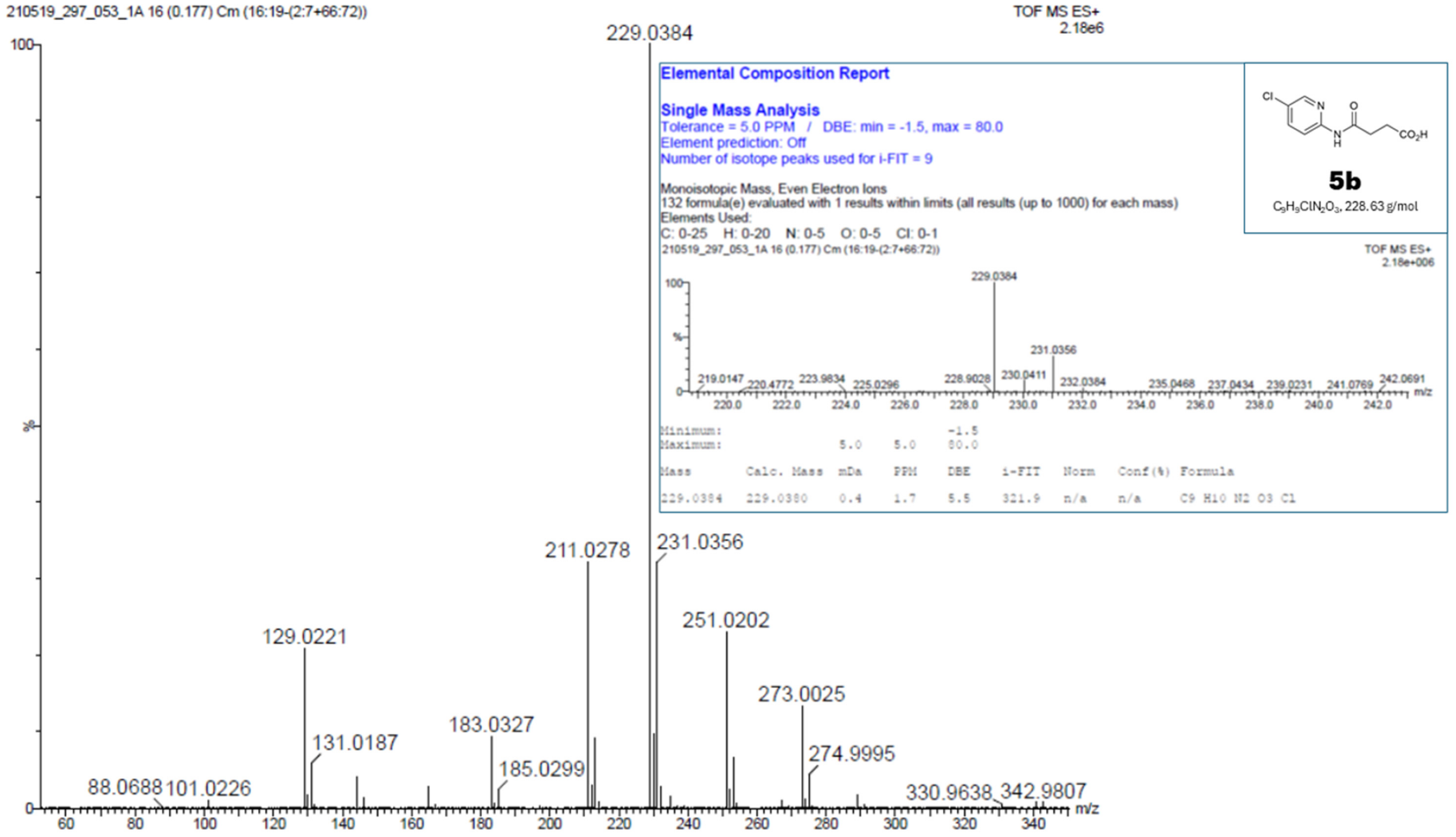

Figure S40. 5b — HRMS (+) spectrum

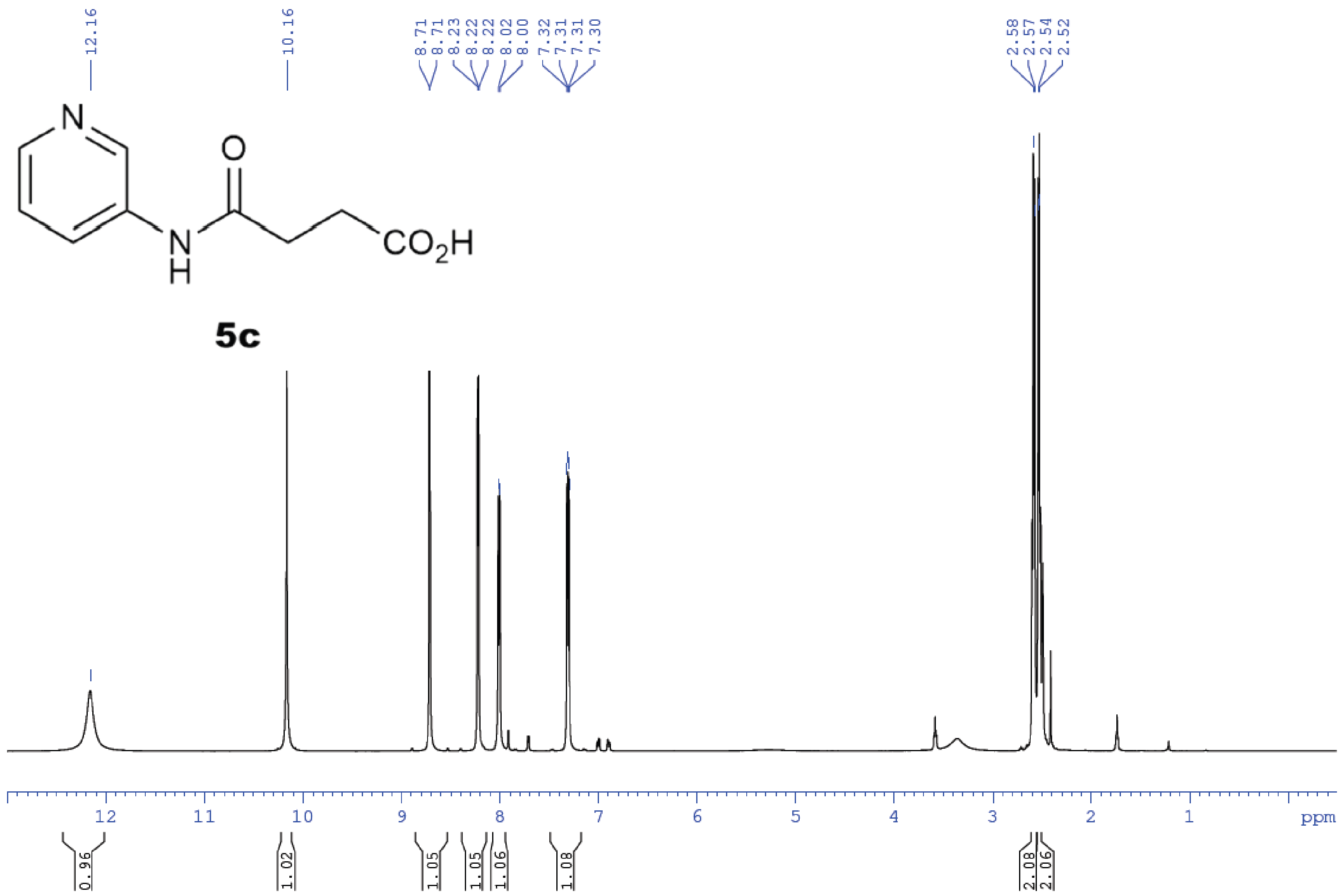

Figure S41. 5c — ^{1}H NMR spectrum

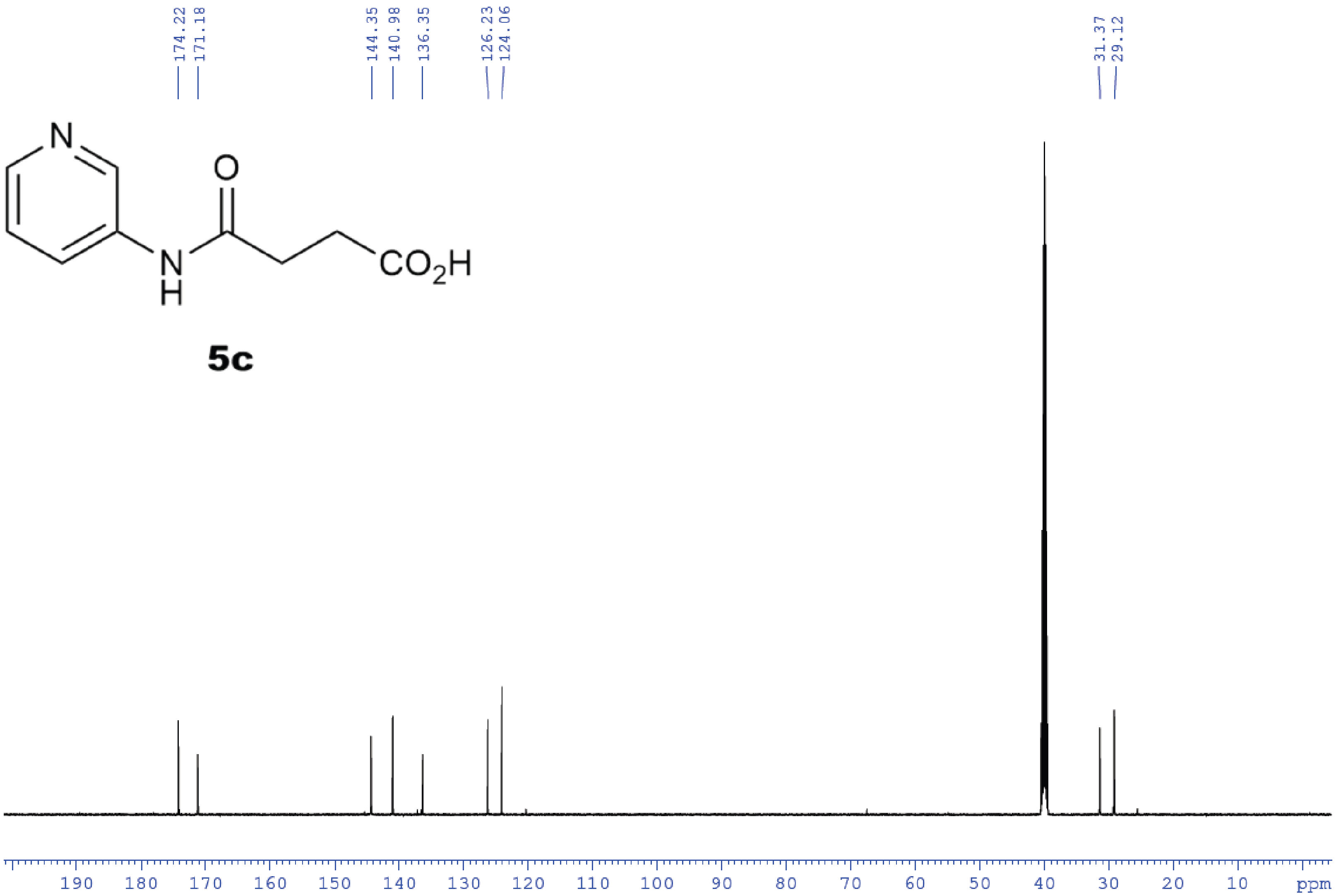

Figure S42. 5c — ^{13}C NMR spectrum

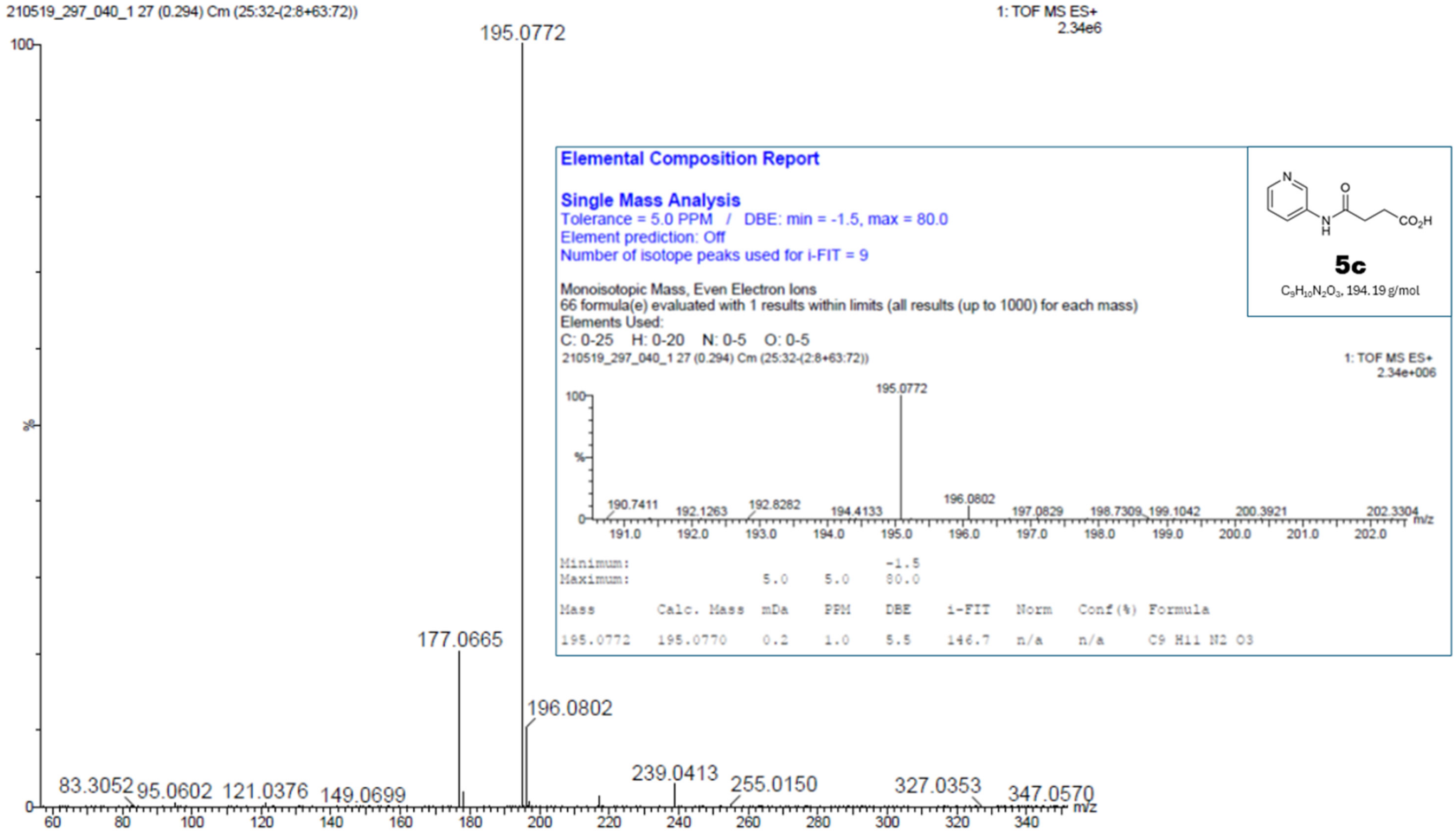

Figure S43. 5c — HRMS (+) spectrum

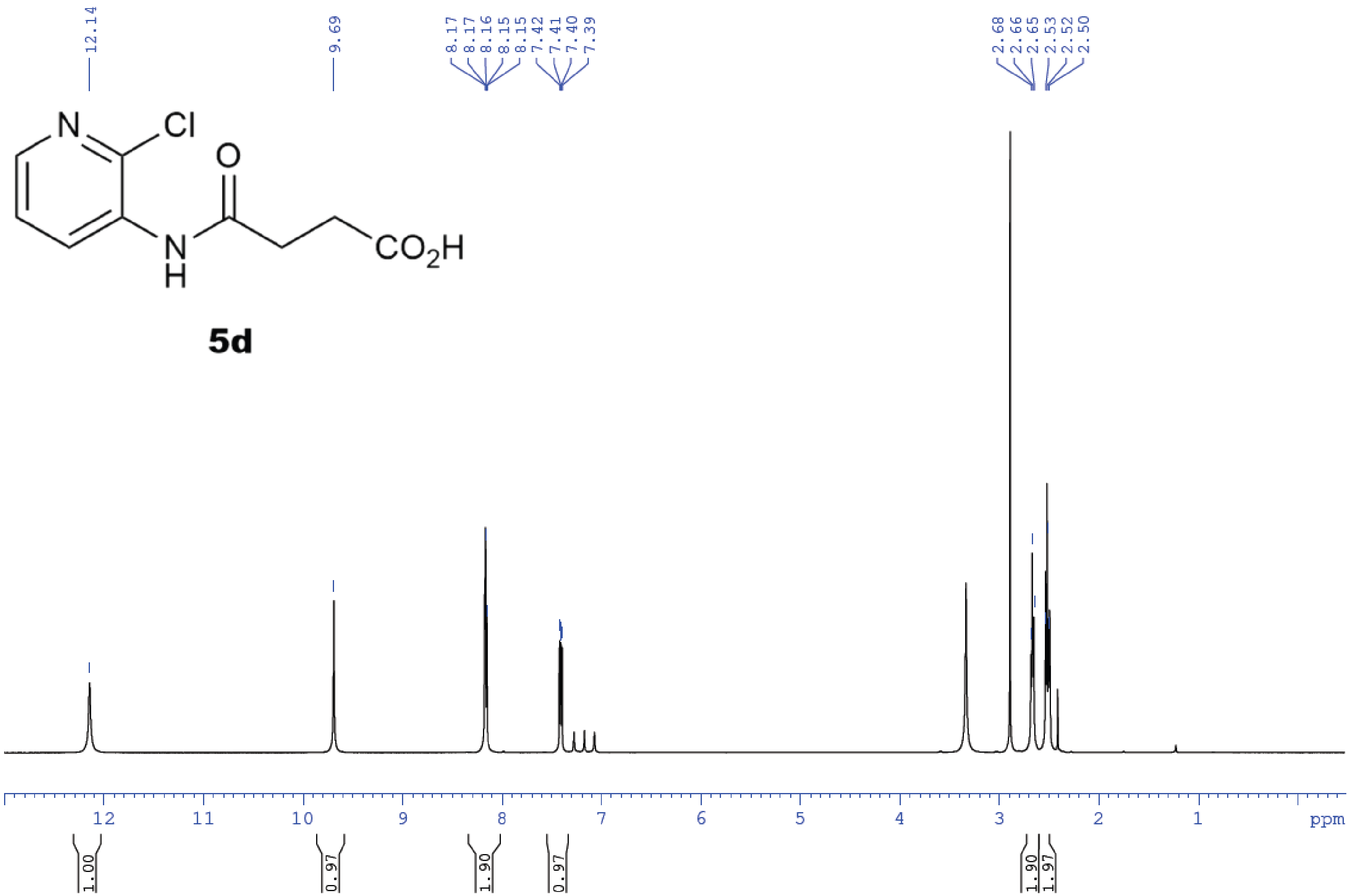

Figure S44. 5d — ^{1}H NMR spectrum

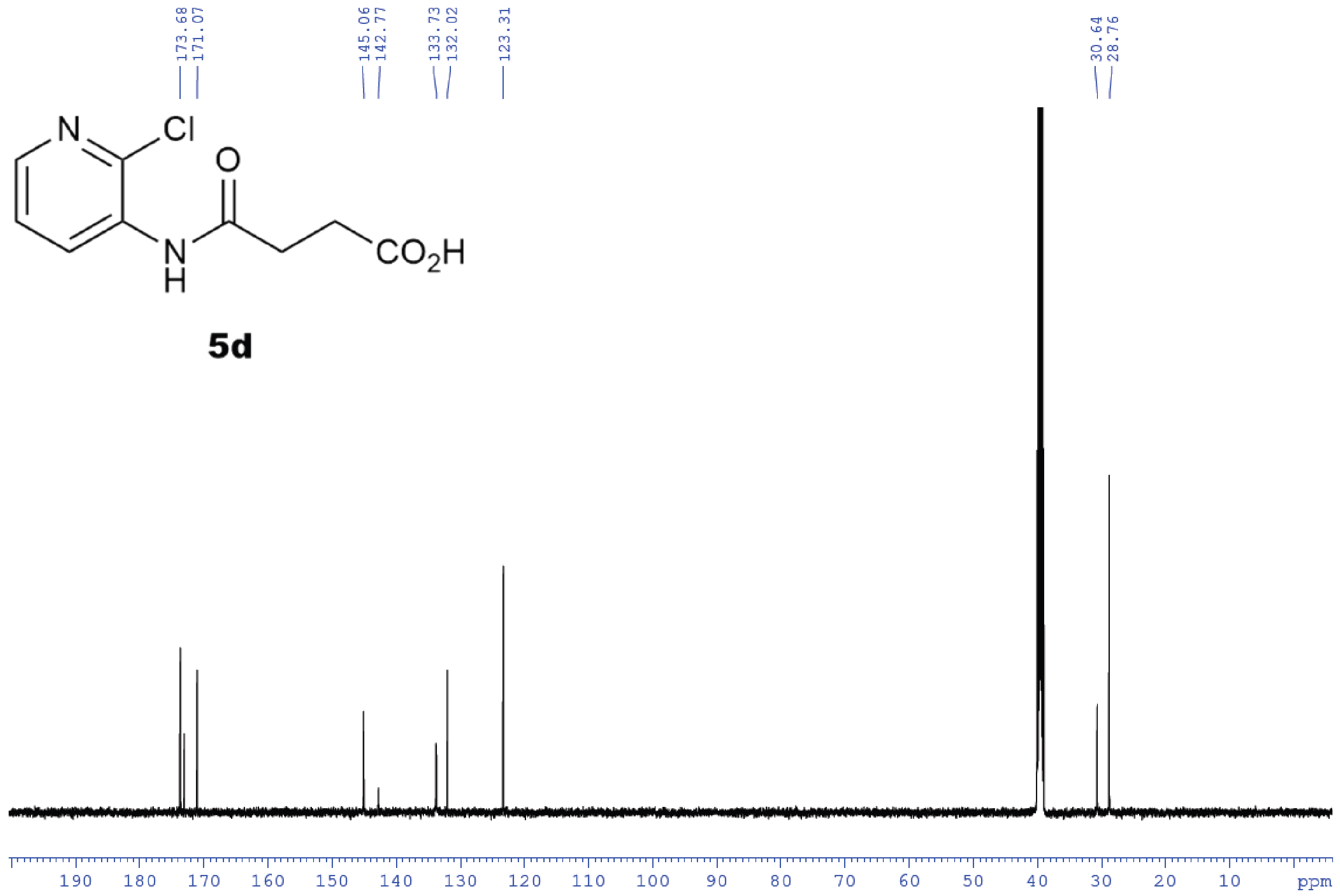

Figure S45. 5d — ^{13}C NMR spectrum

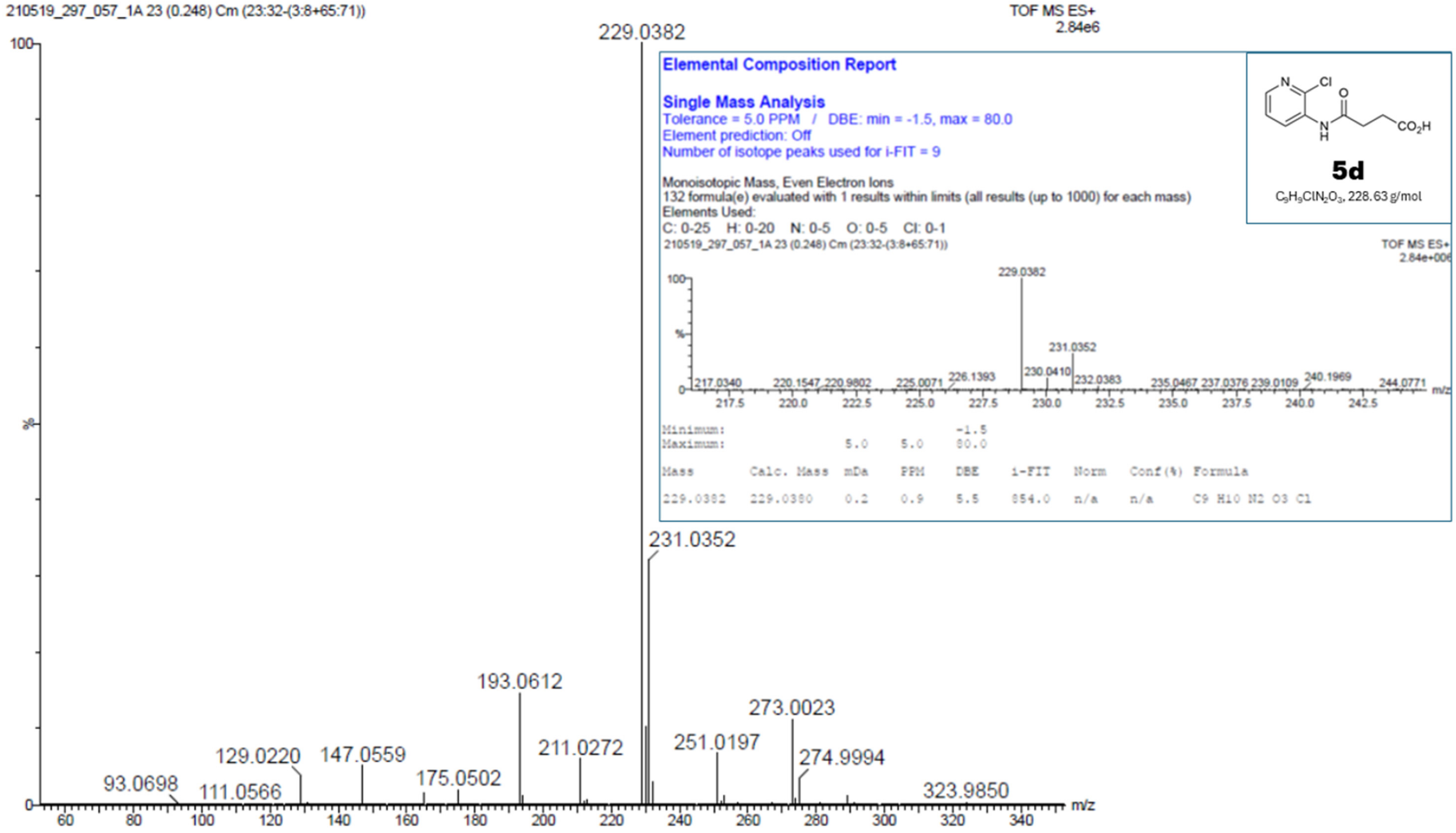

Figure S46. 5d — HRMS (+) spectrum

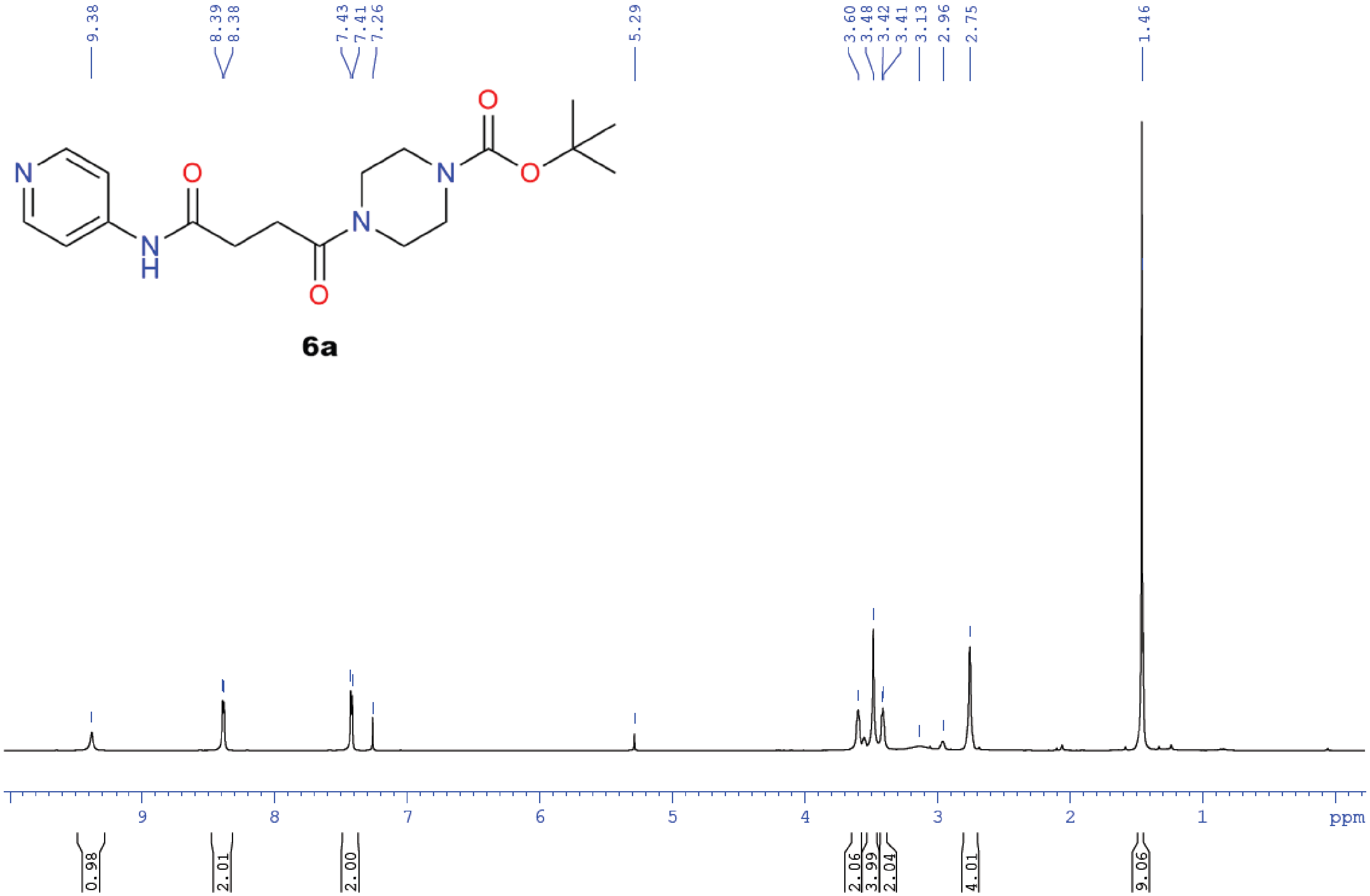

Figure S47. 6a — ^{1}H NMR spectrum

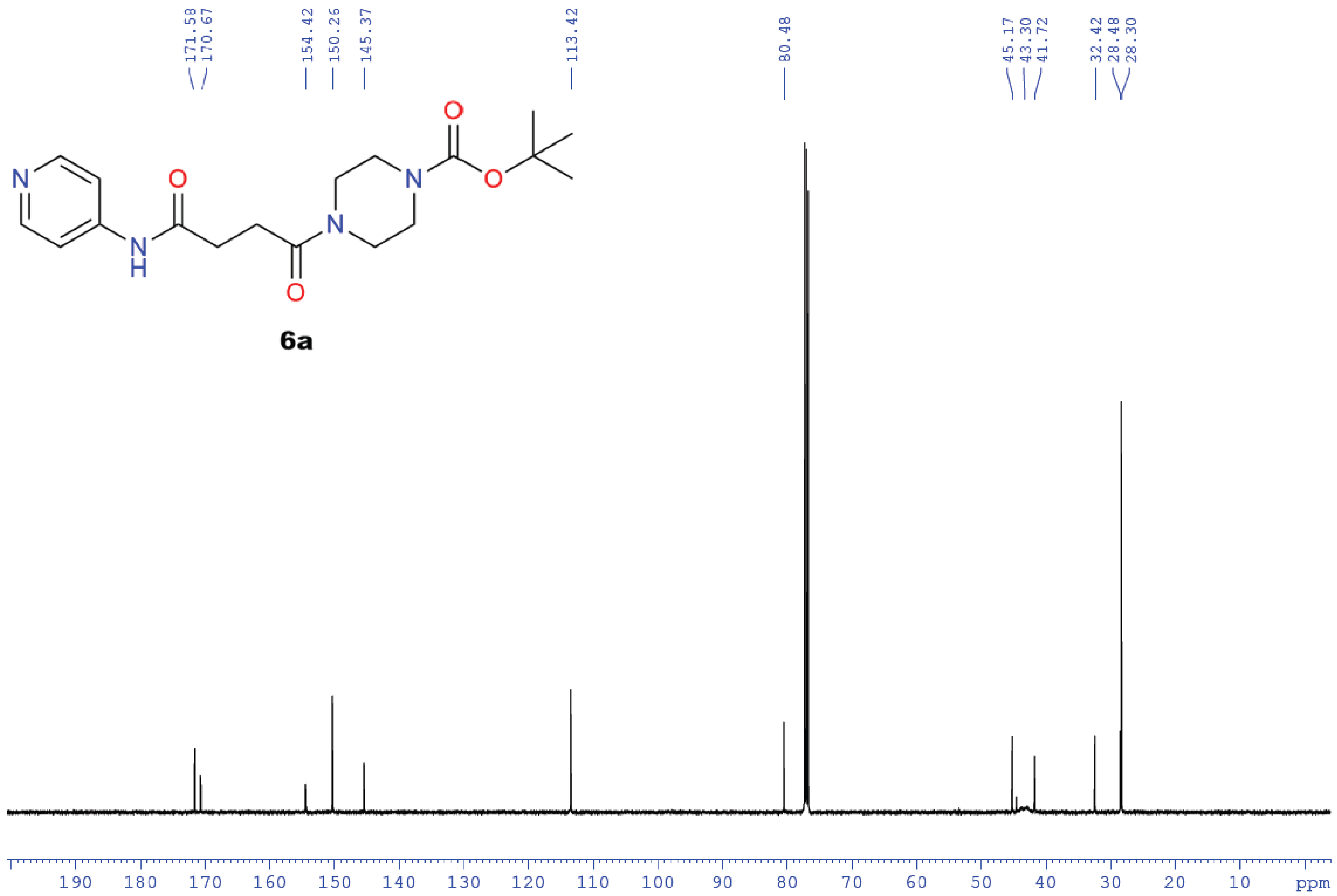

Figure S48. 6a — ^{13}C NMR spectrum

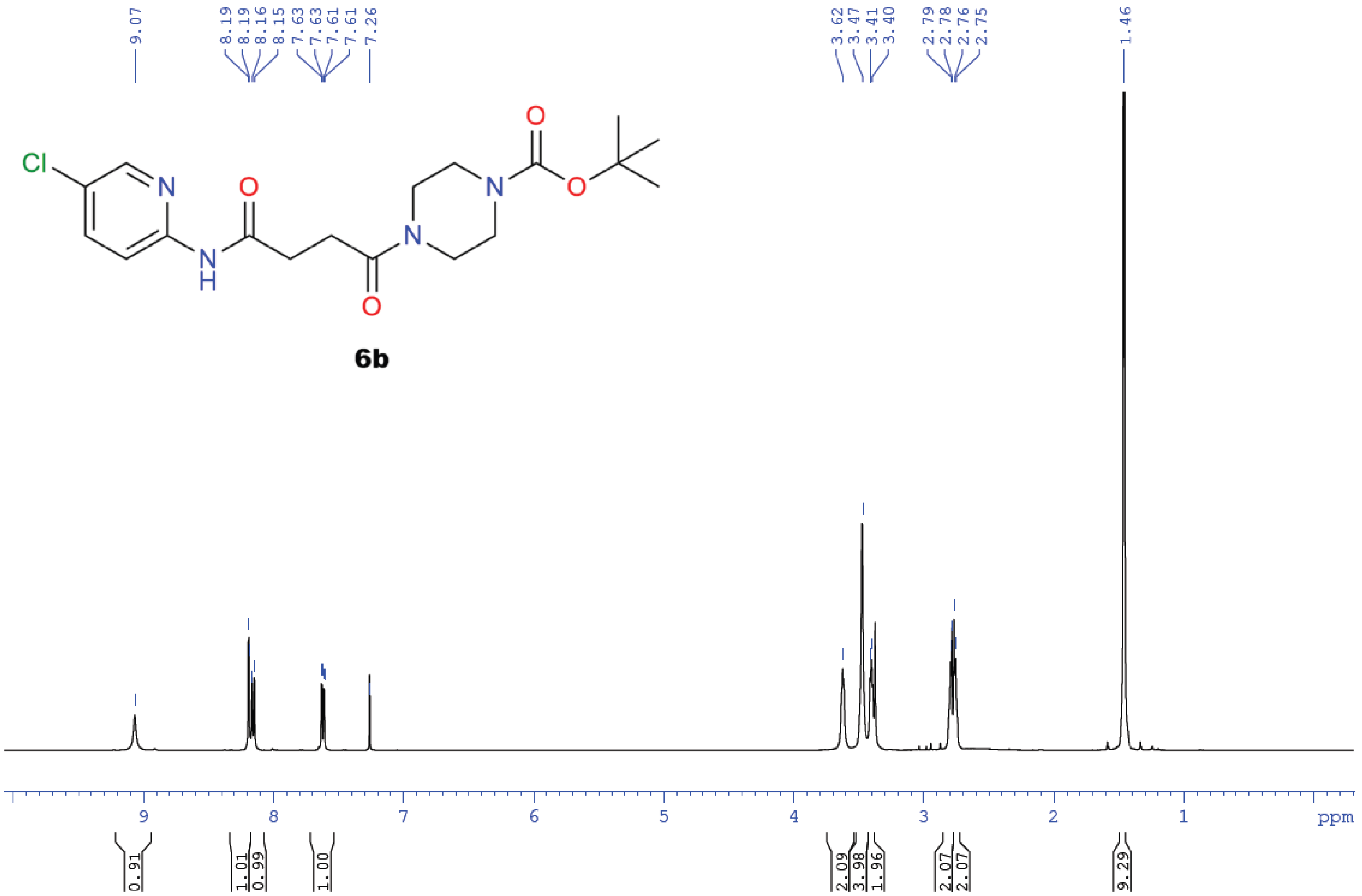

Figure S49. 6b — ^{1}H NMR spectrum

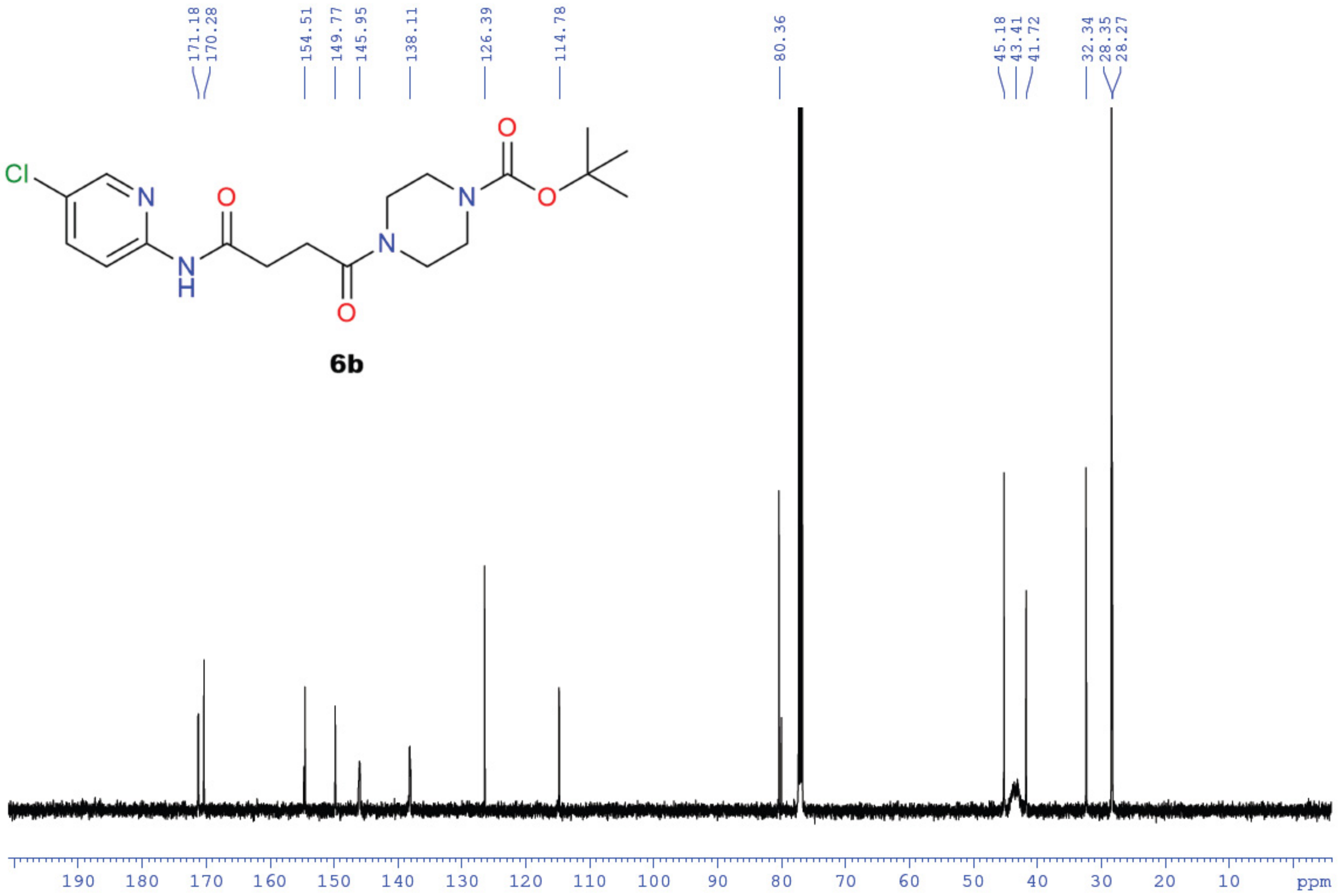

Figure S50. 6b — ^{13}C NMR spectrum

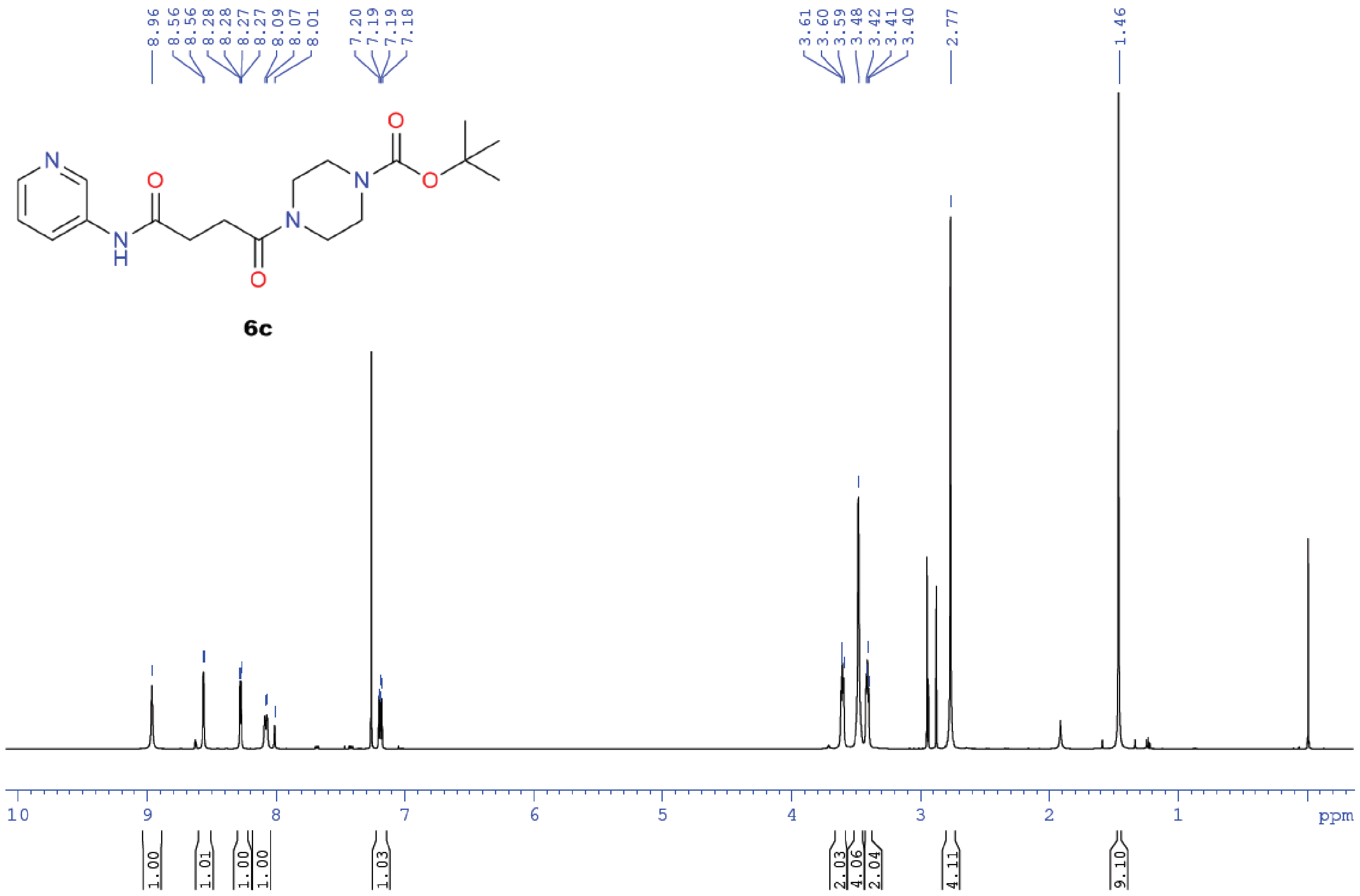

Figure S51. 6c — ^{1}H NMR spectrum

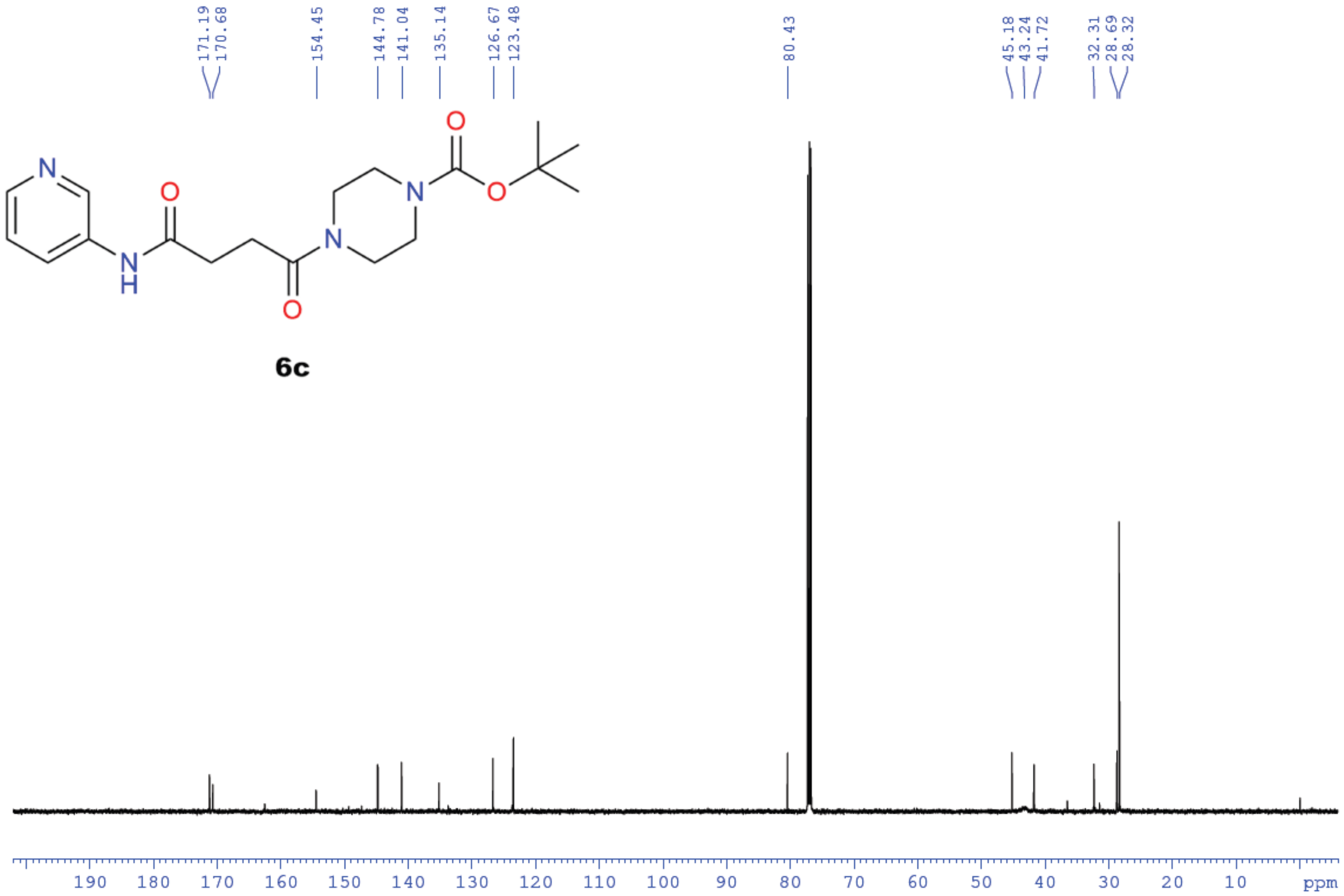

Figure S52. 6c — ^{13}C NMR spectrum

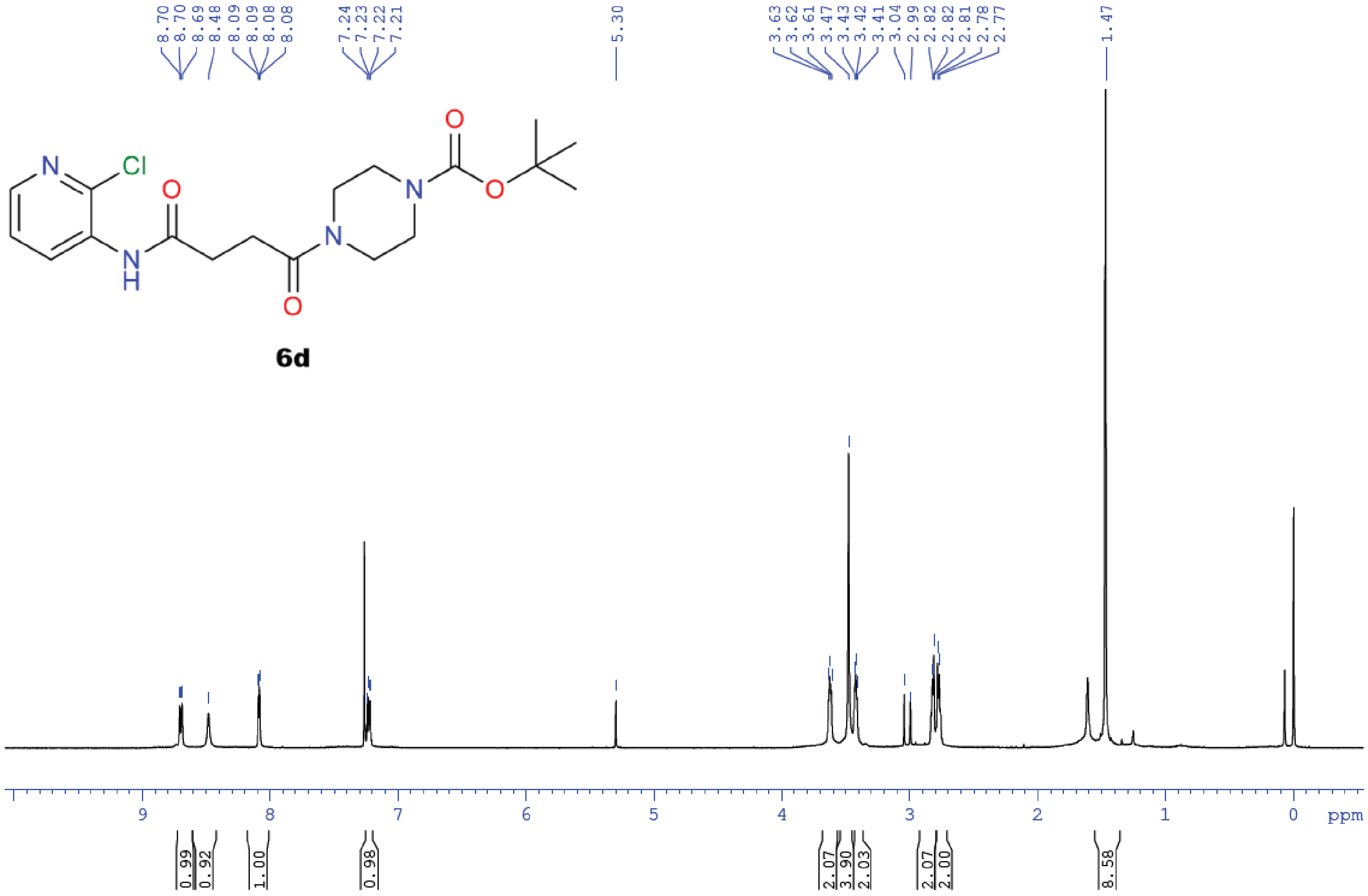

Figure S53. 6d — ^{1}H NMR spectrum

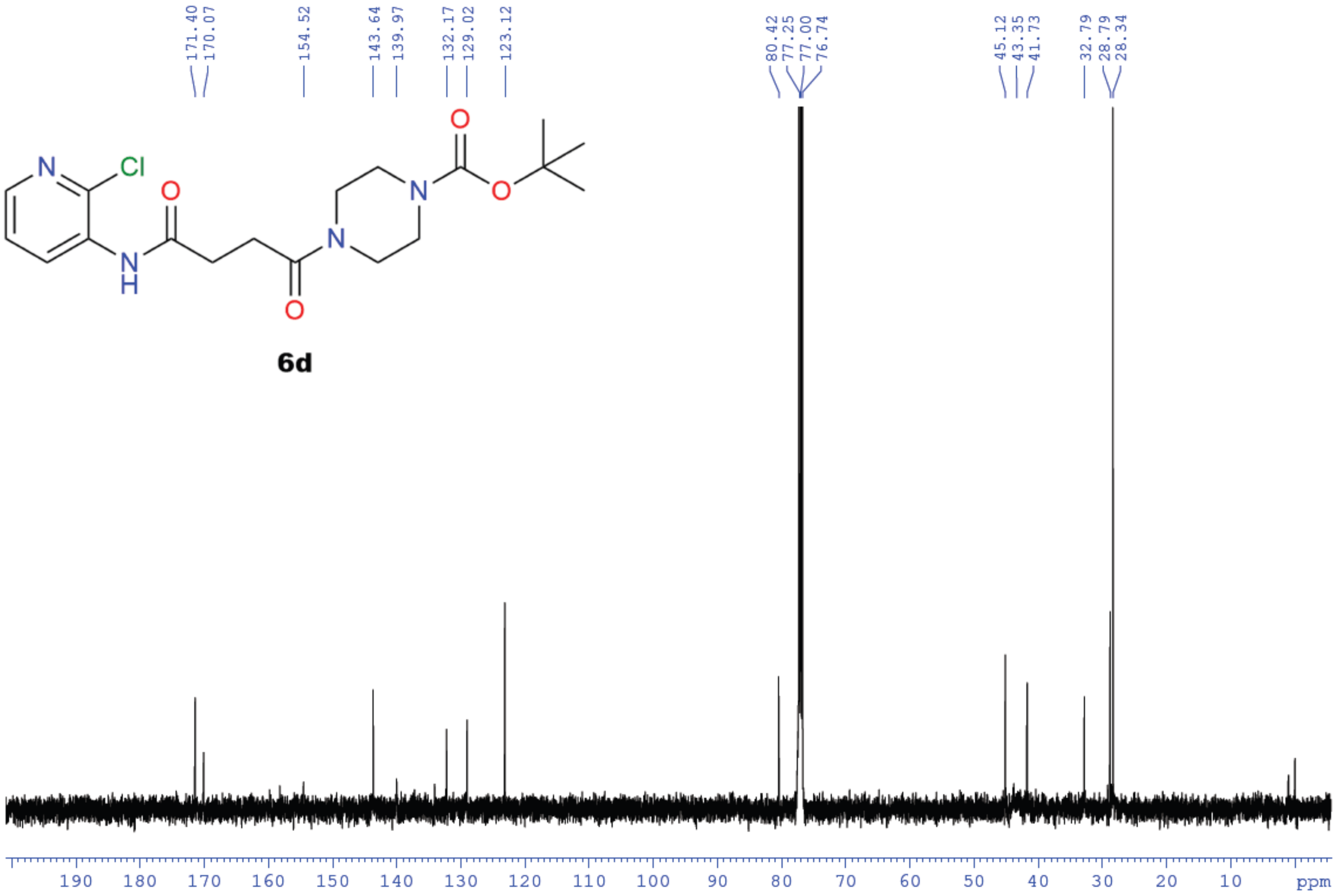

Figure S54. 6d — ^{13}C NMR spectrum

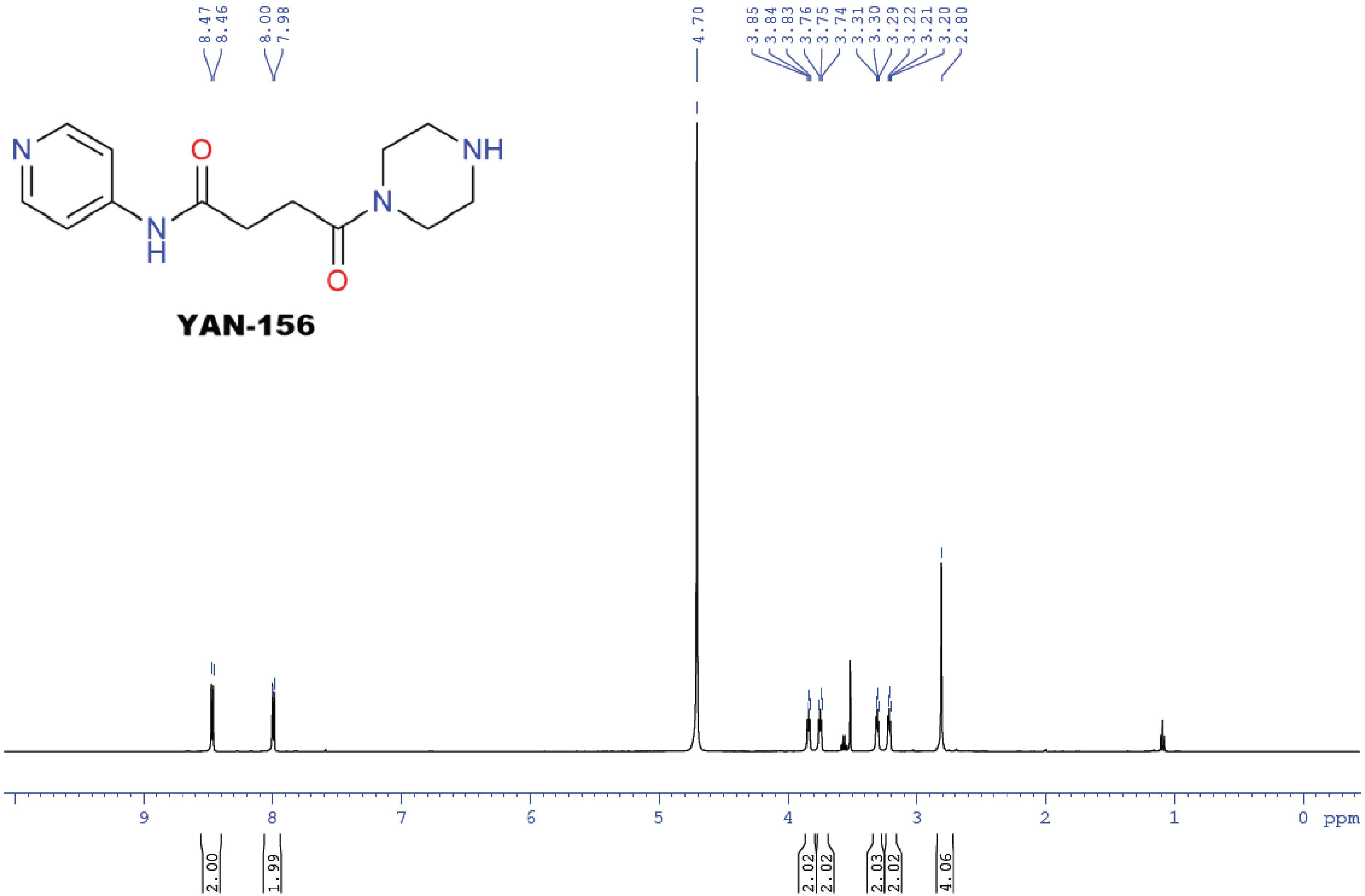

Figure S55. YAN-156 — ^{1}H NMR spectrum

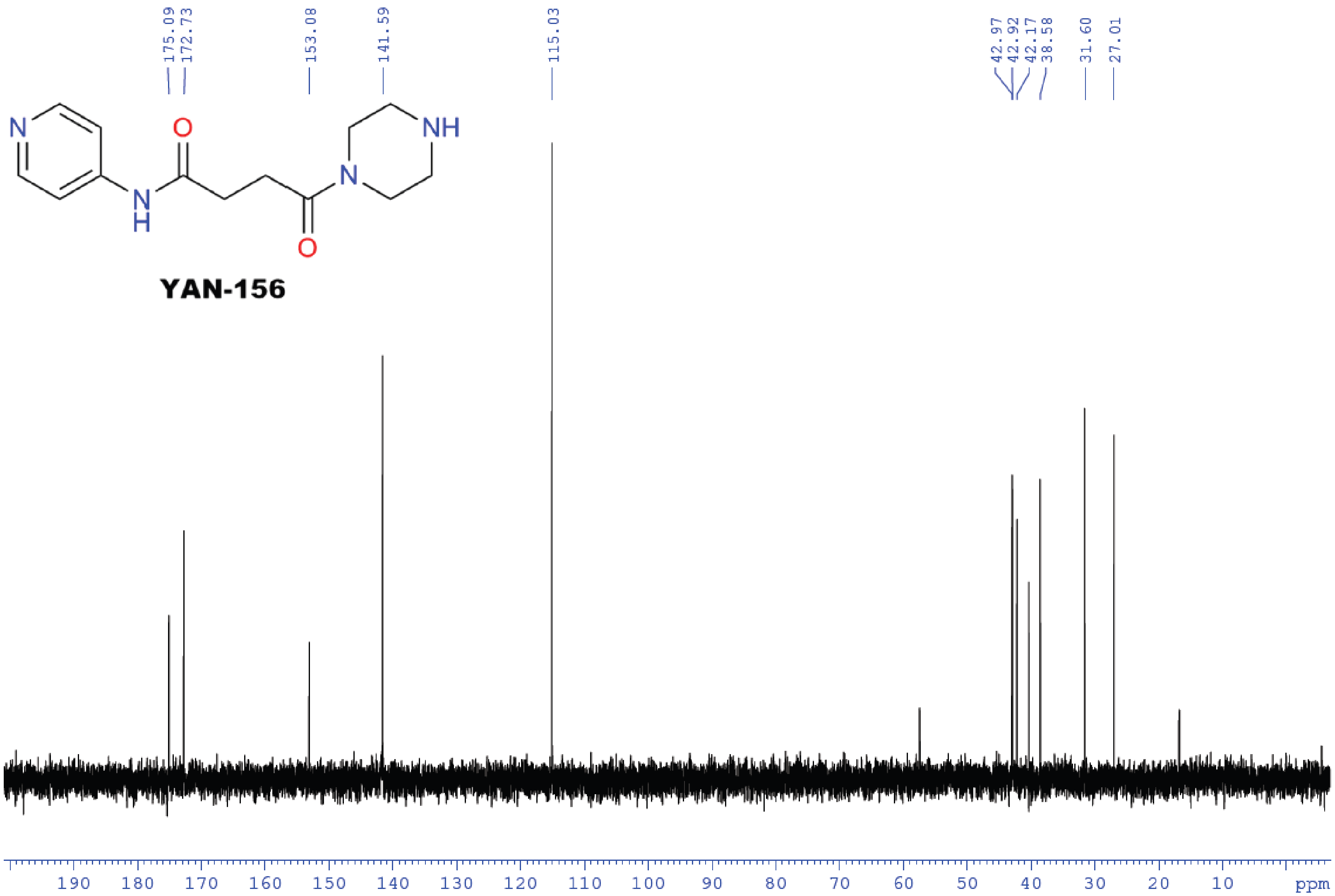

Figure S56. YAN-156 — ^{13}C NMR spectrum

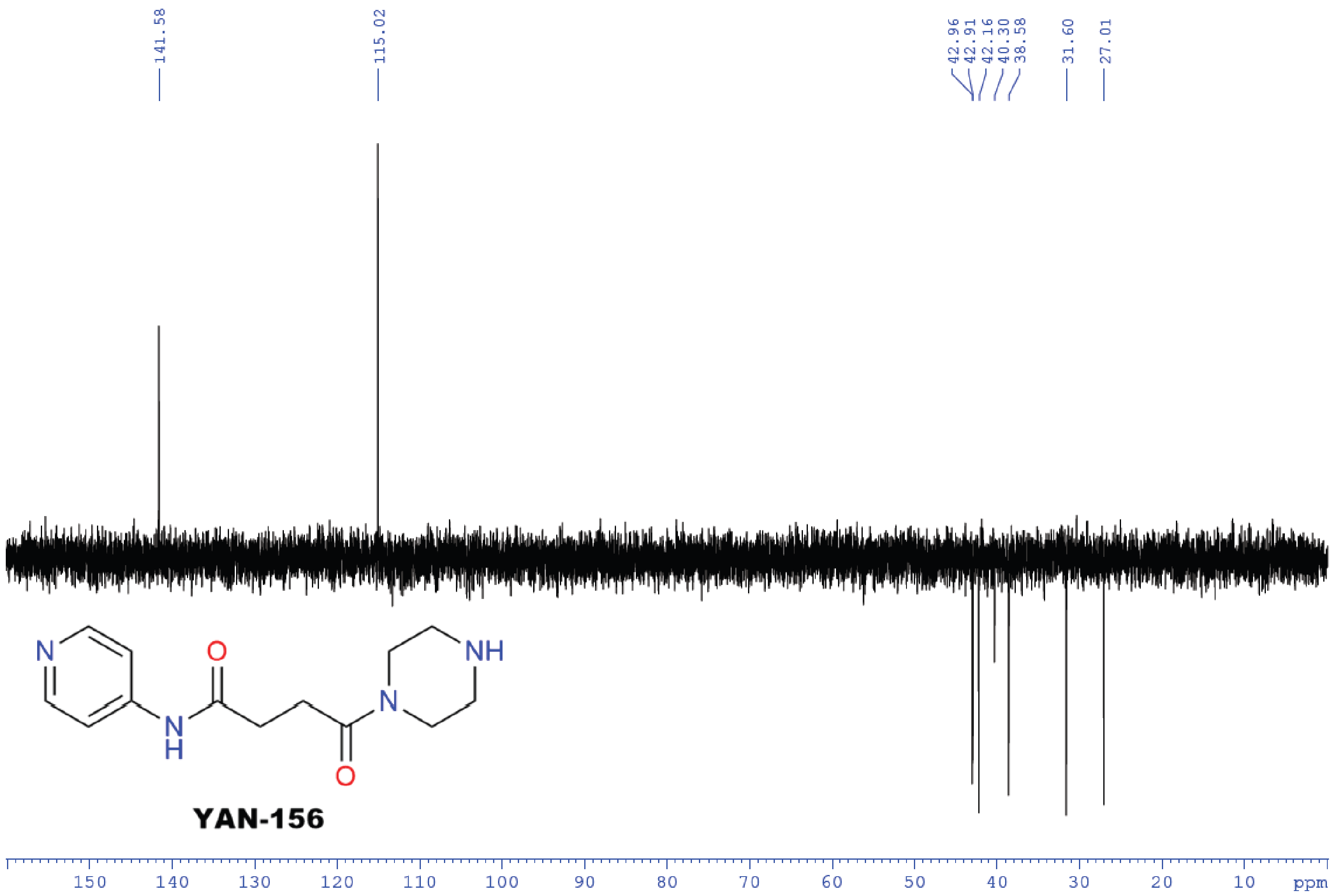

Figure S57. YAN-156 — DEPT NMR spectrum

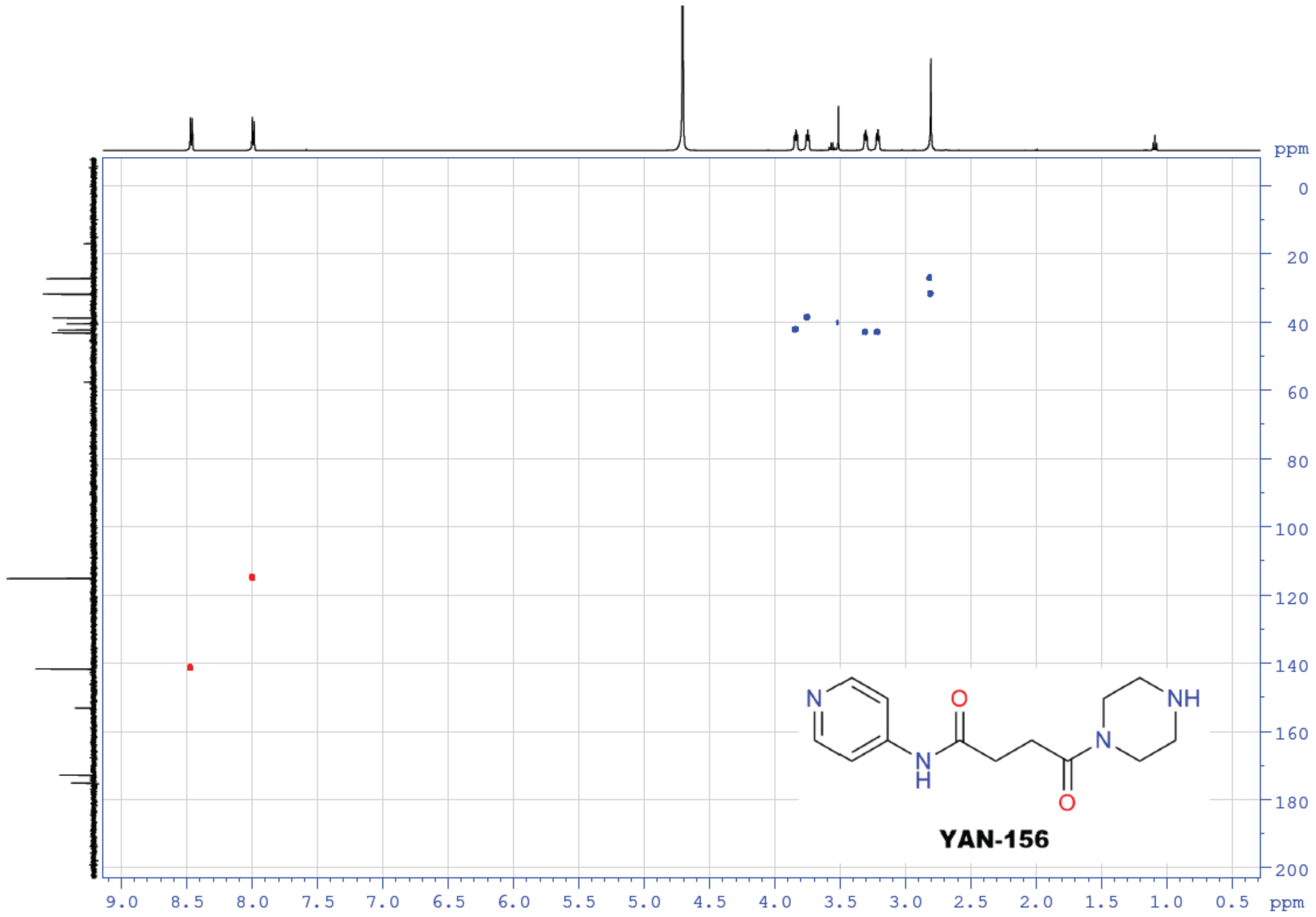

Figure S58. YAN-156 — ^{1}H-^{13}C HSQC NMR spectrum

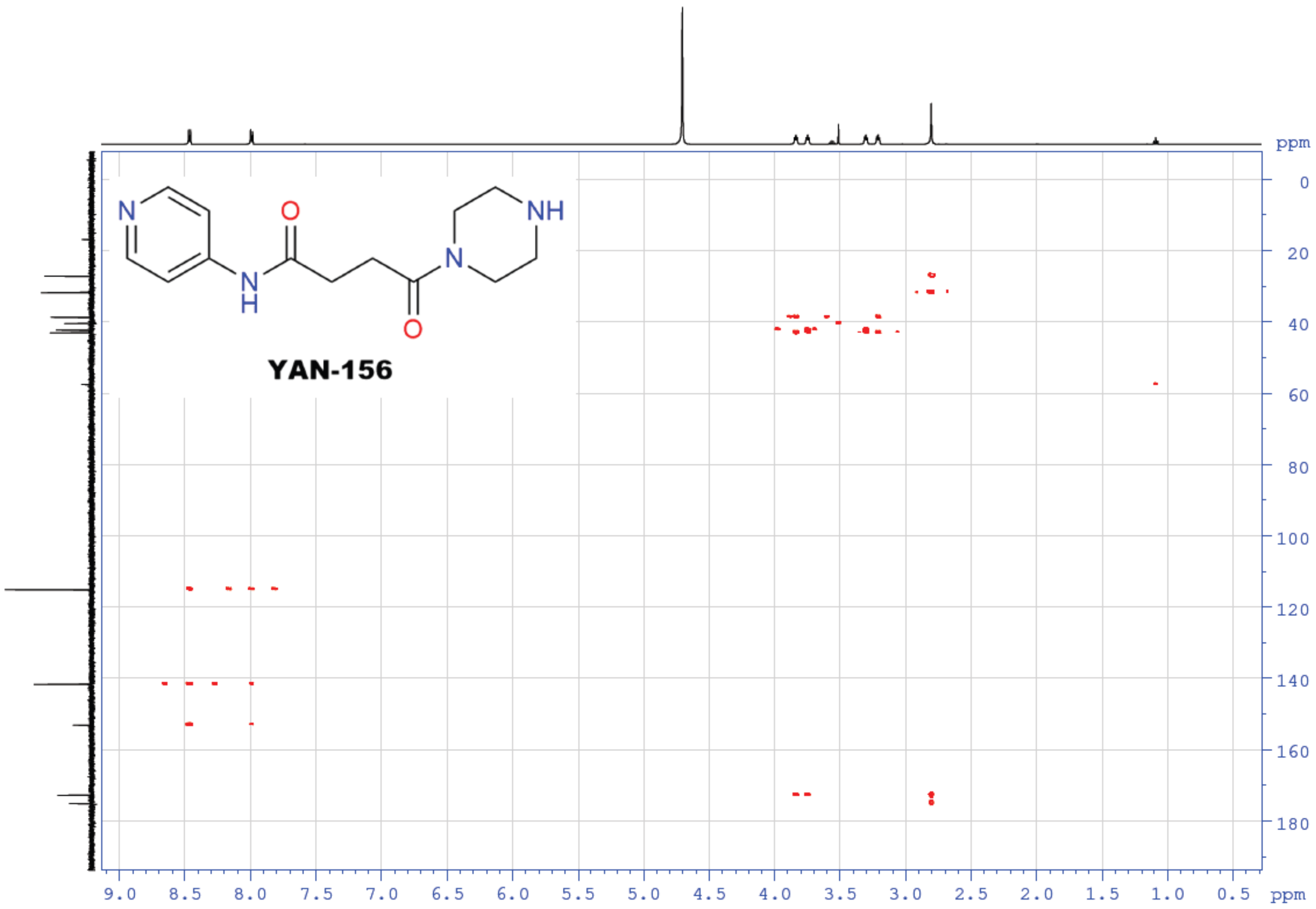

Figure S59. YAN-156 — $^{1}H \rightarrow ^{13}C$ HMBC NMR spectrum

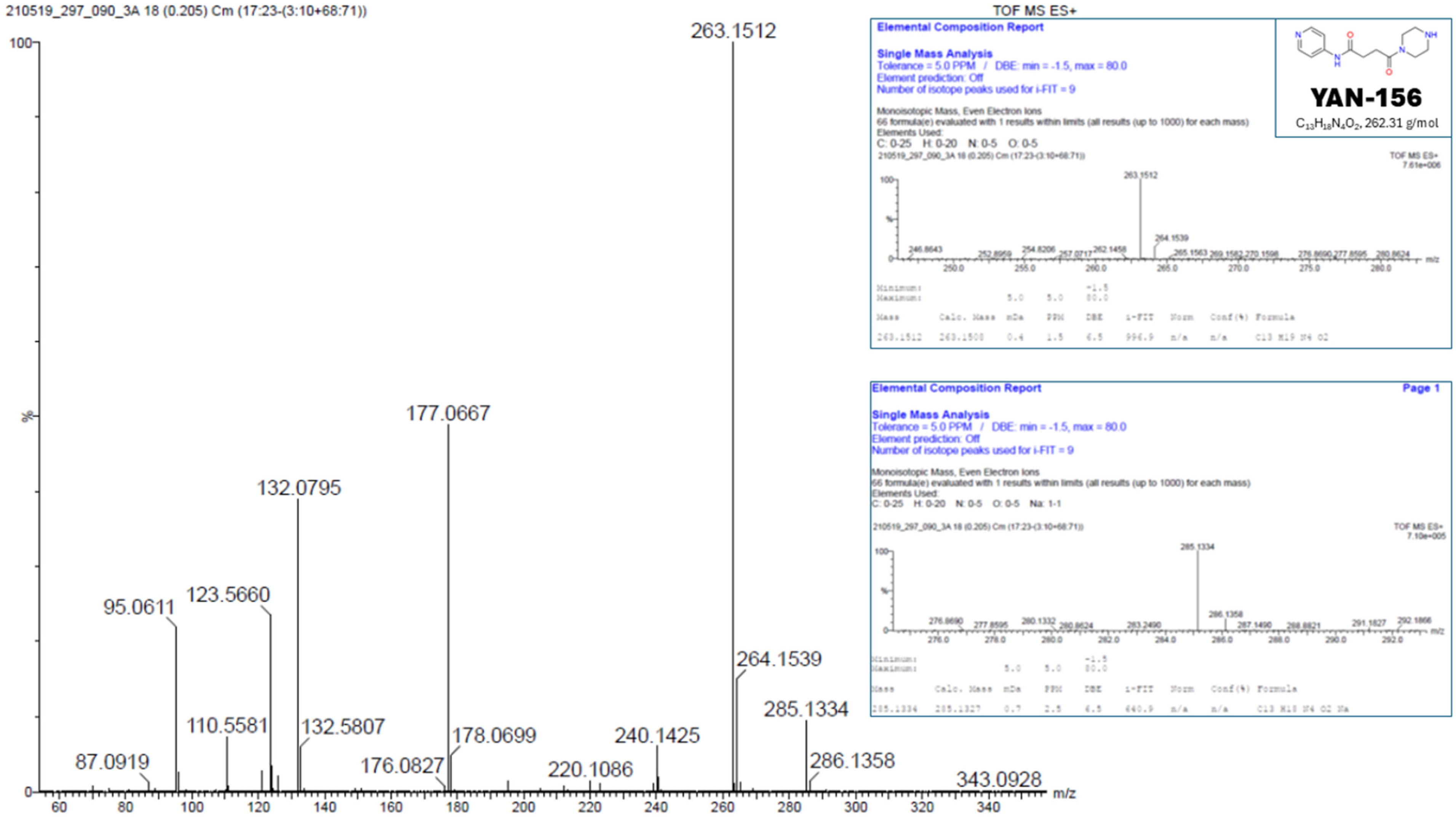

Figure S60. YAN-156 — HRMS (+) spectrum

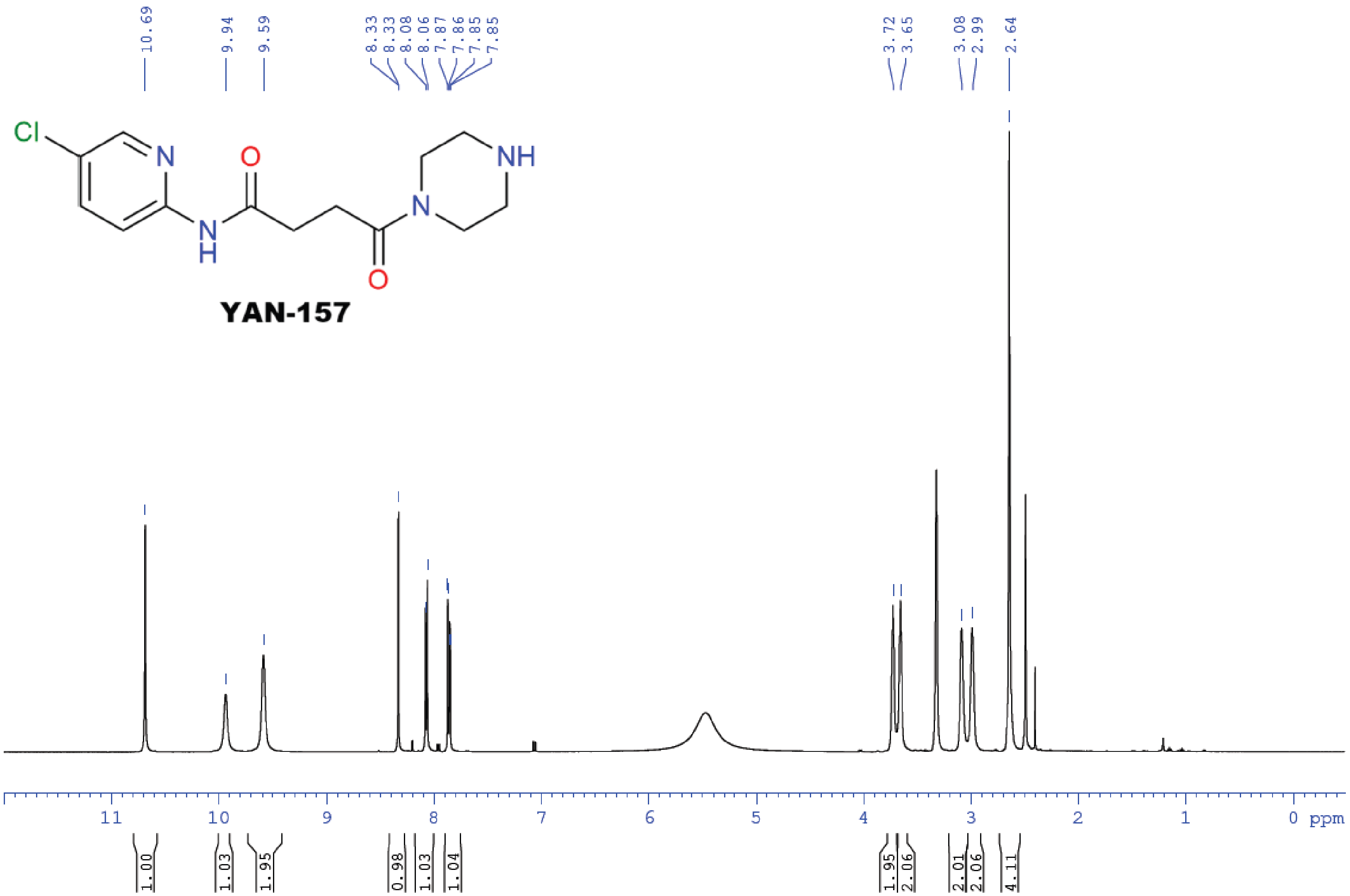

Figure S61. YAN-157 — ^{1}H NMR spectrum

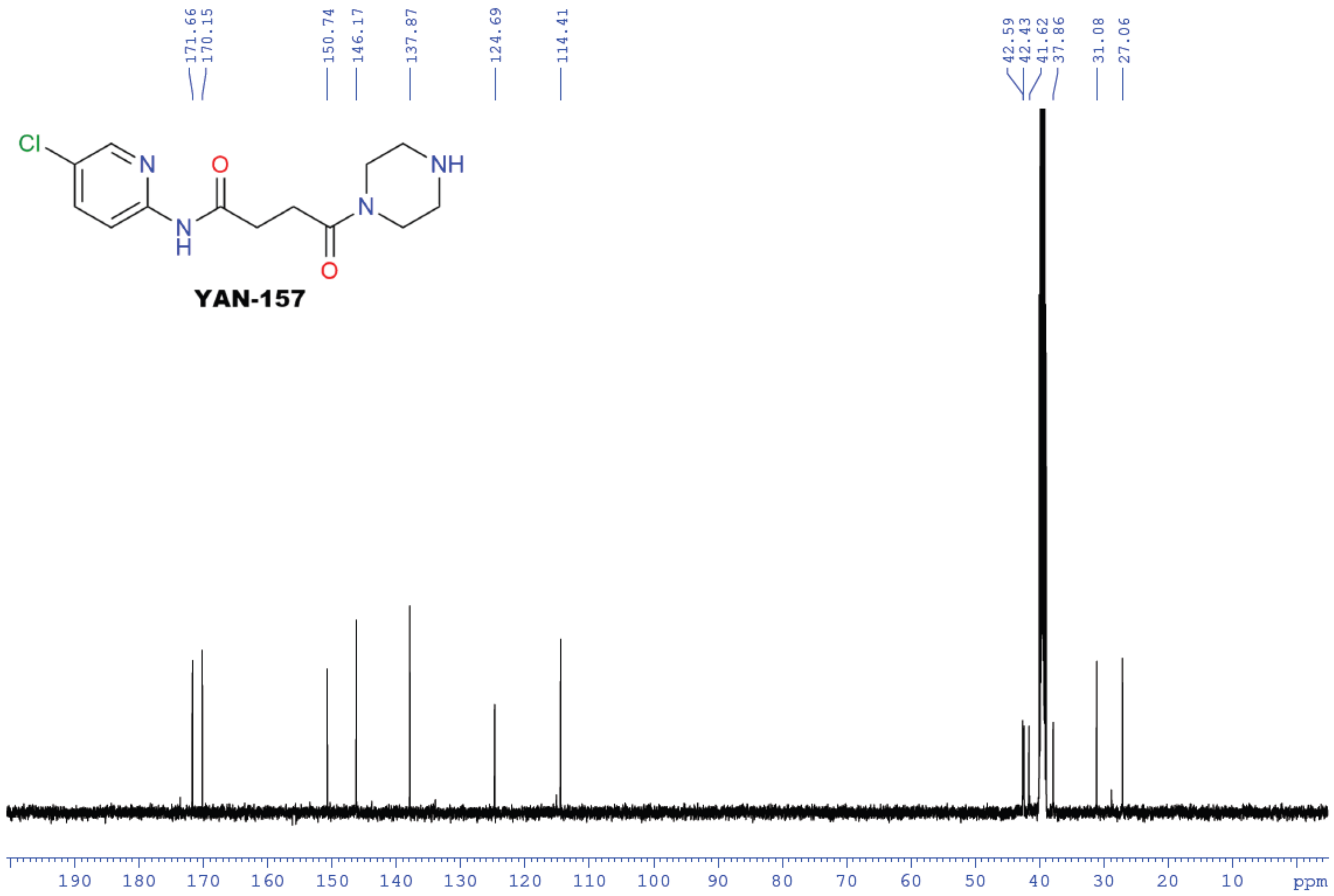

Figure S62. YAN-157 — ^{13}C NMR spectrum

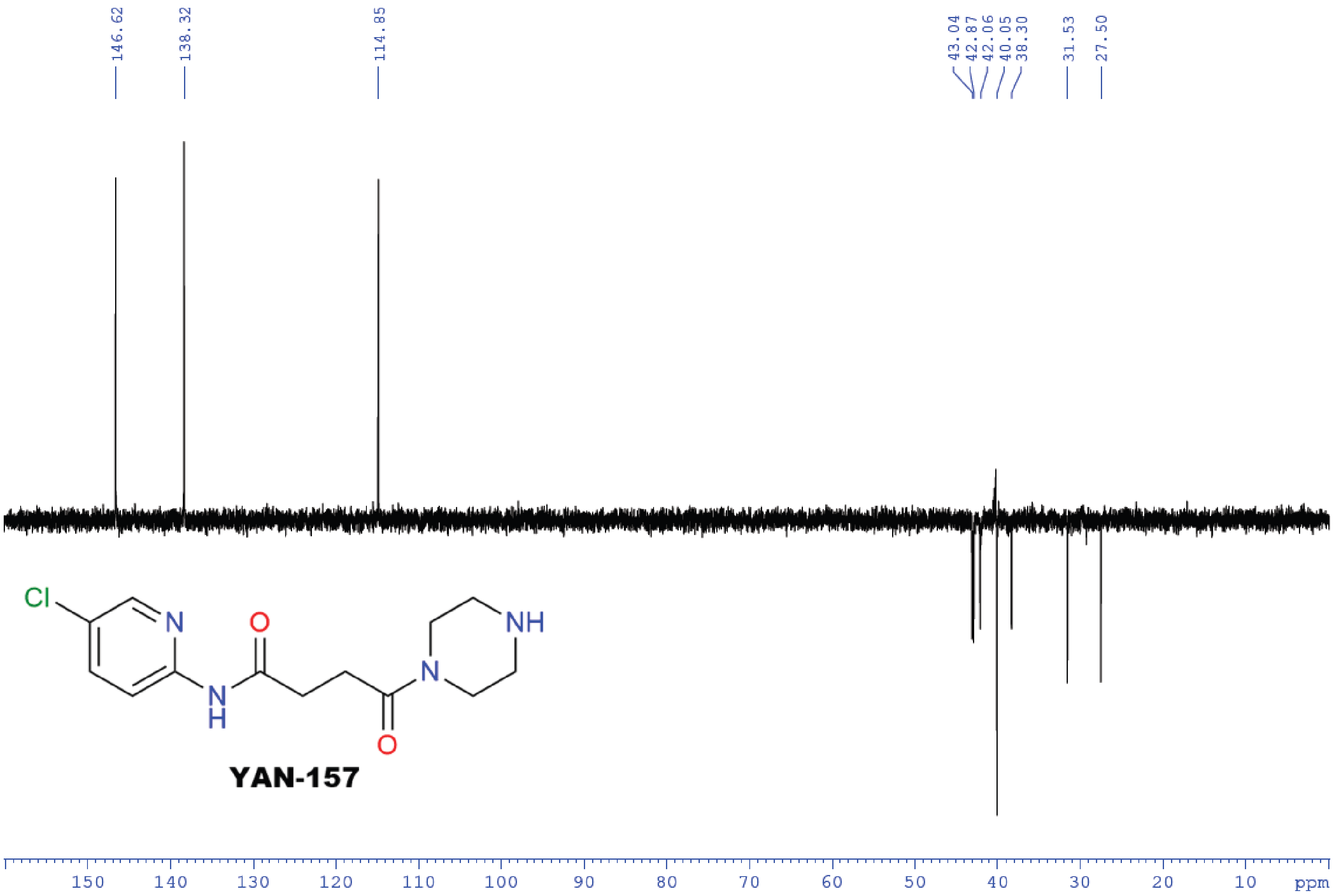

Figure S63. YAN-157 — DEPT NMR spectrum

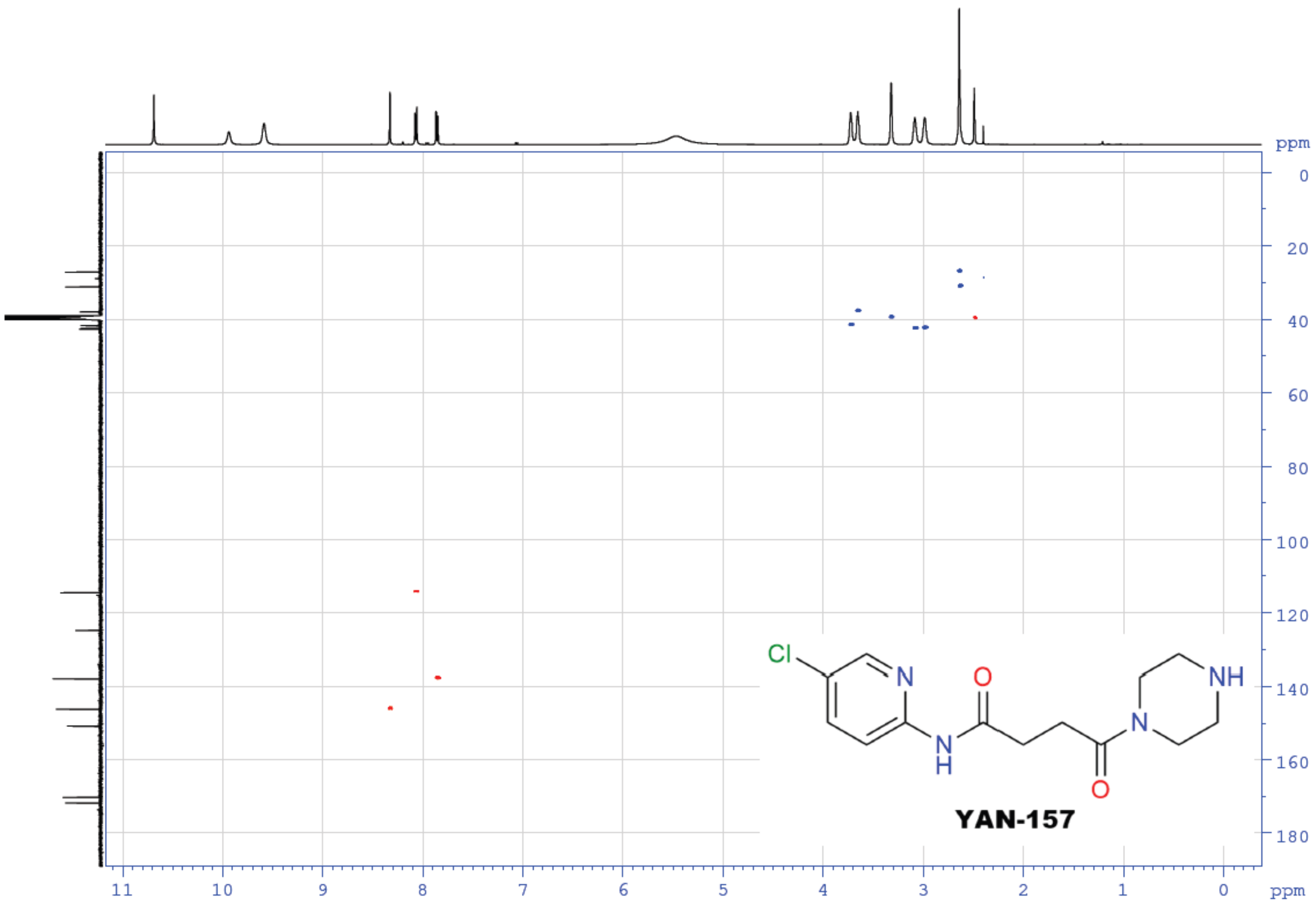

Figure S64. YAN-157 — ^{1}H-^{13}C HSQC NMR spectrum

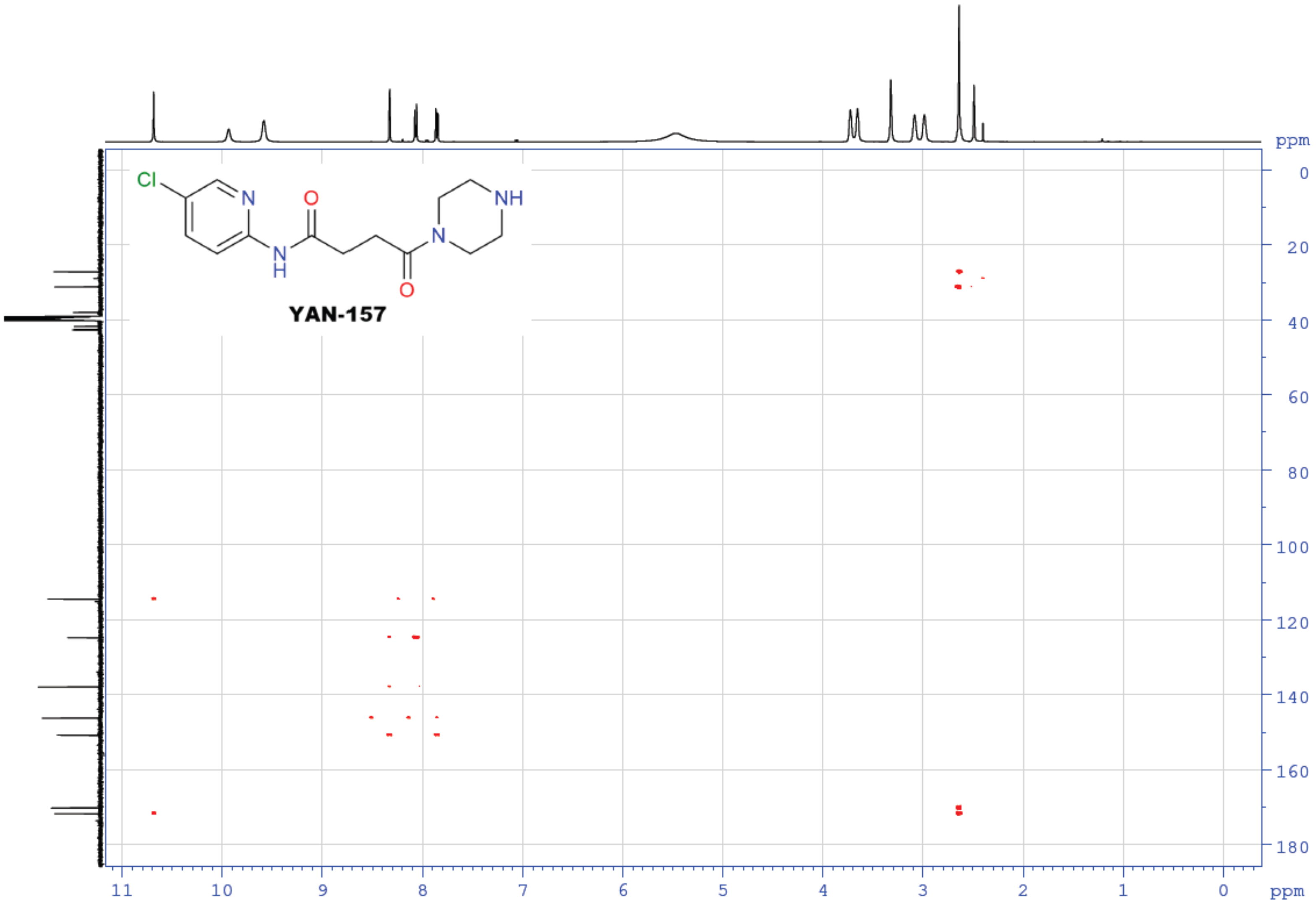

Figure S65. YAN-157 — $^{1}H \rightarrow ^{13}C$ HMBC NMR spectrum

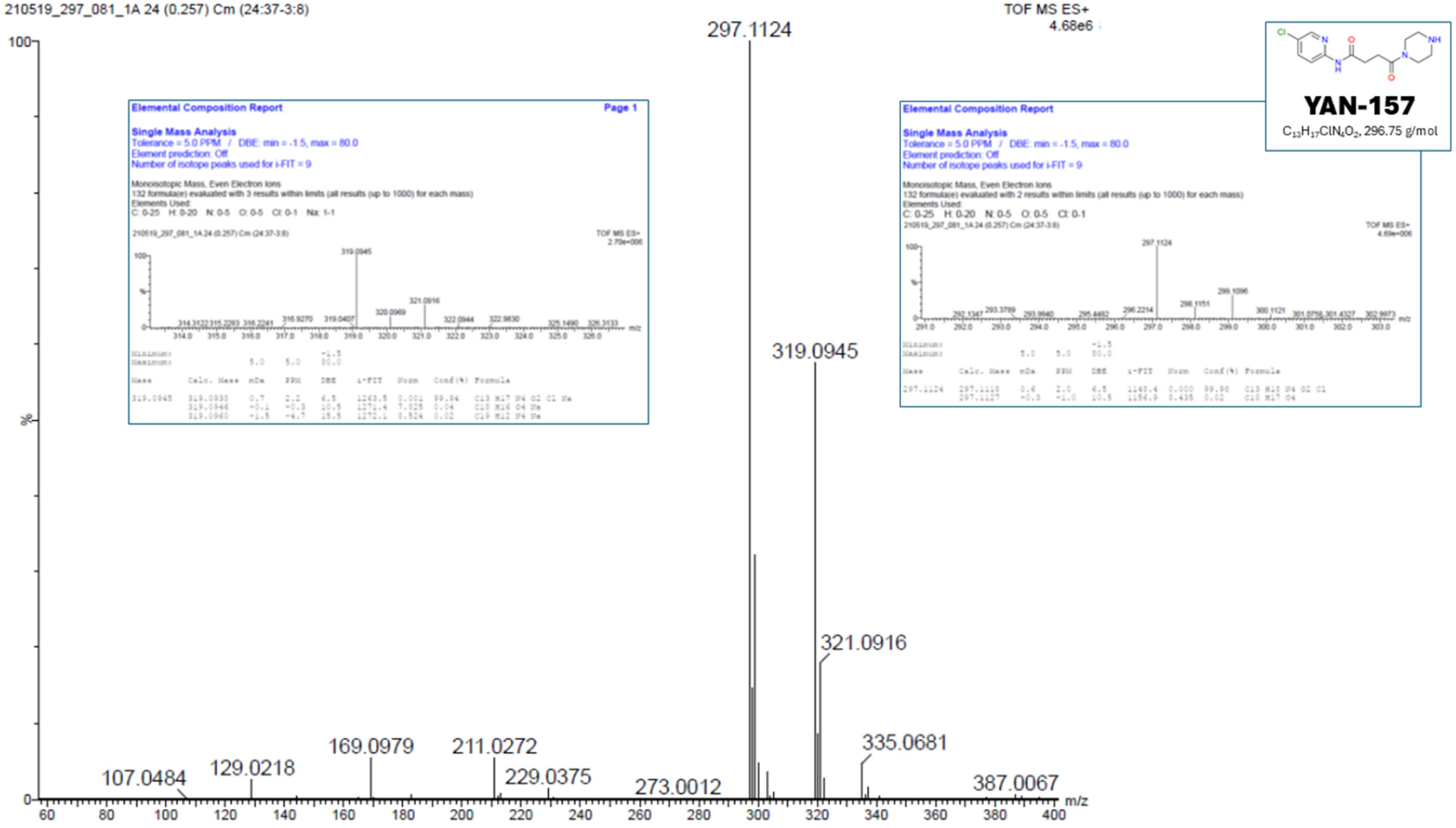

Figure S66. YAN-157 — HRMS (+) spectrum

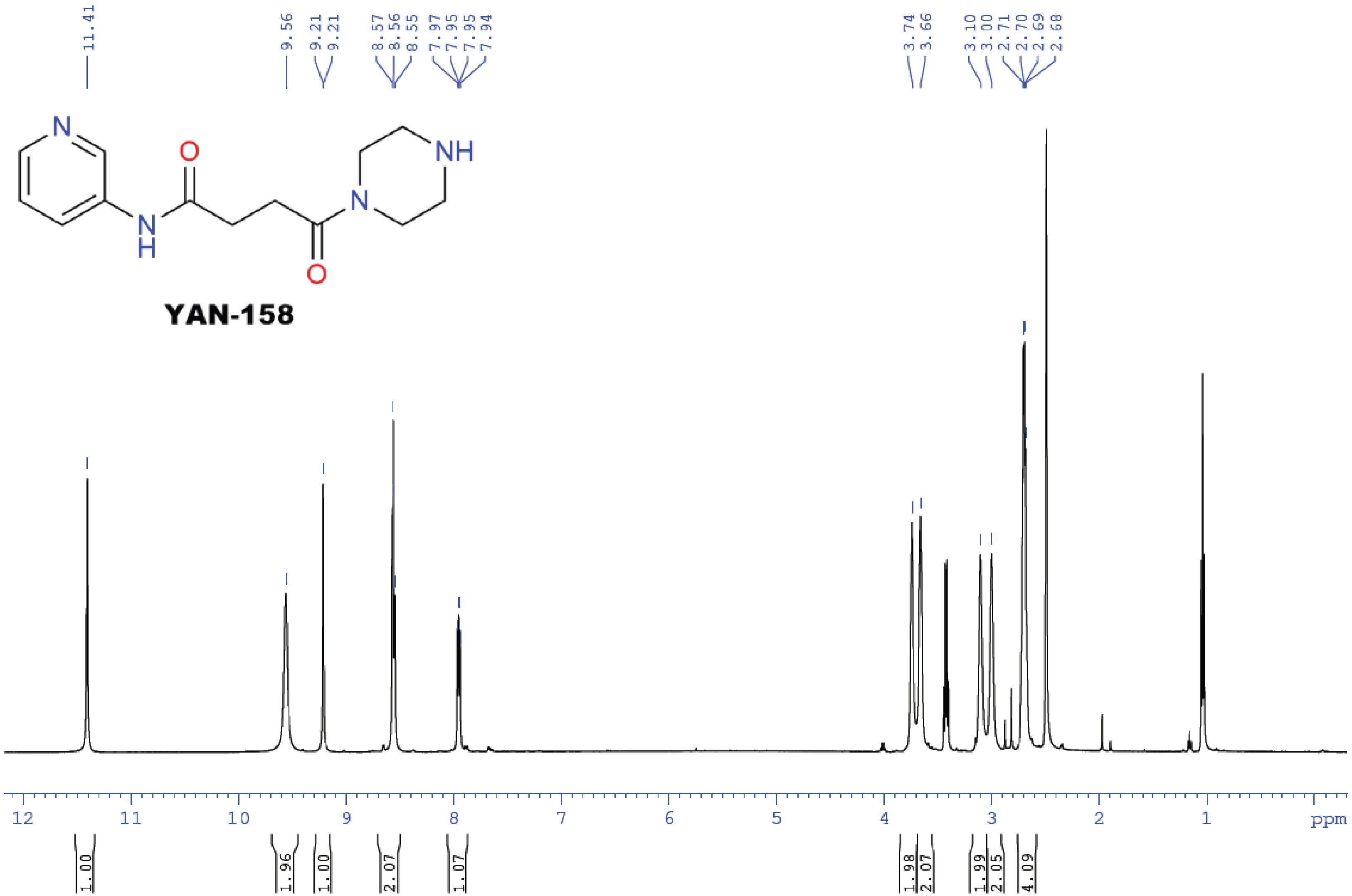

Figure S67. YAN-158 — ^{1}H NMR spectrum

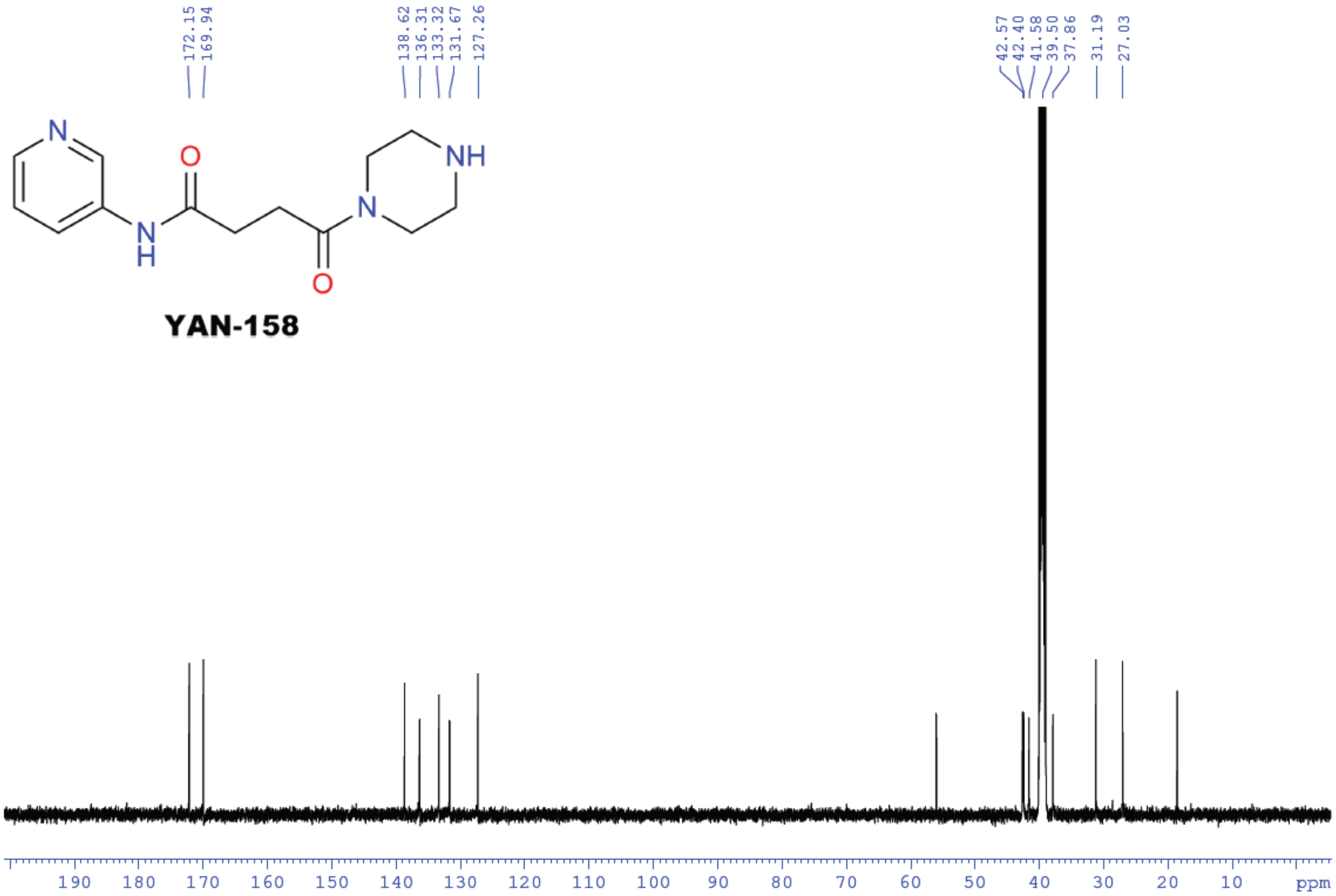

Figure S68. YAN-158 — ^{13}C NMR spectrum

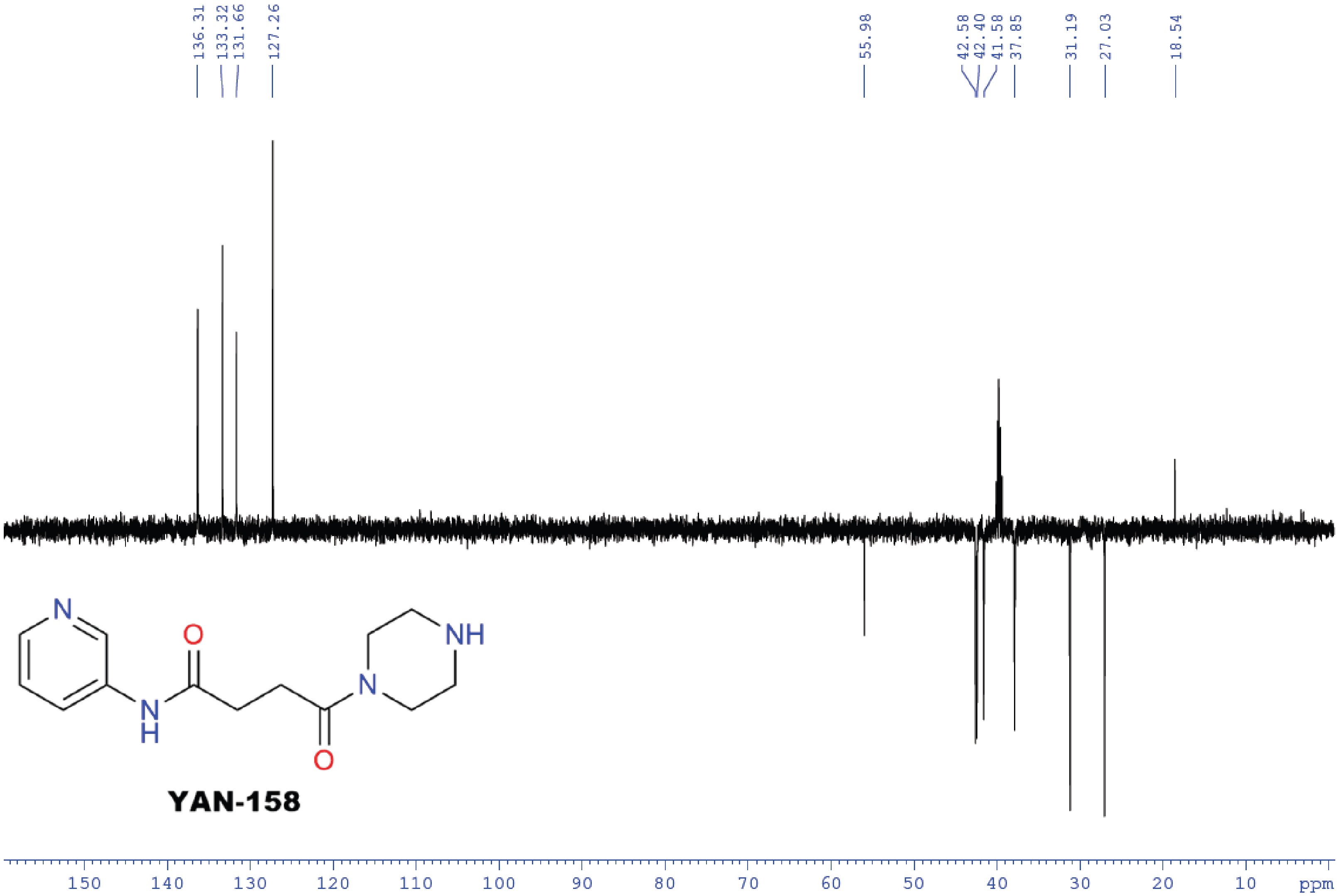

Figure S69. YAN-158 — DEPT NMR spectrum

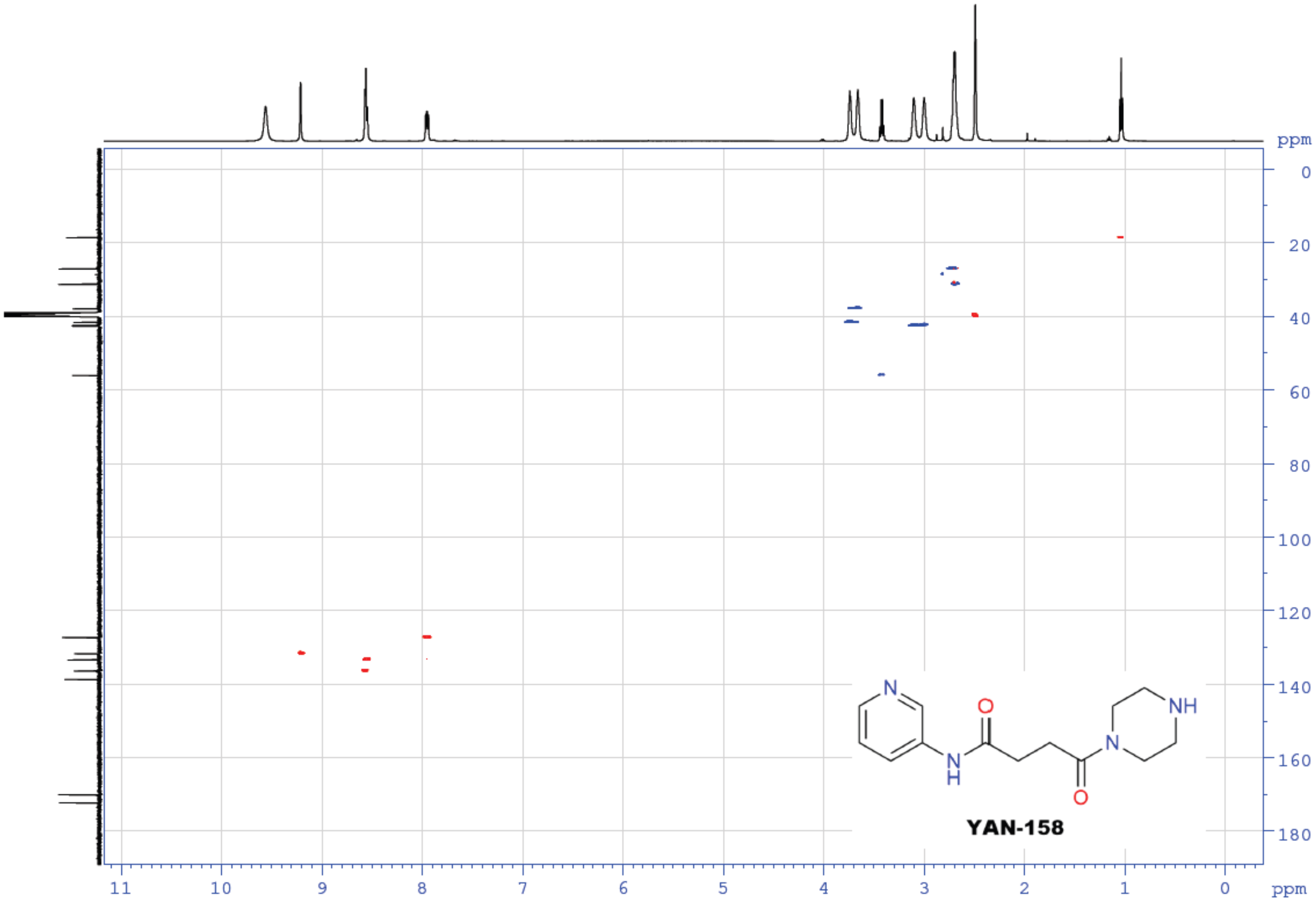

Figure S70. YAN-158 — ^{1}H-^{13}C HSQC NMR spectrum

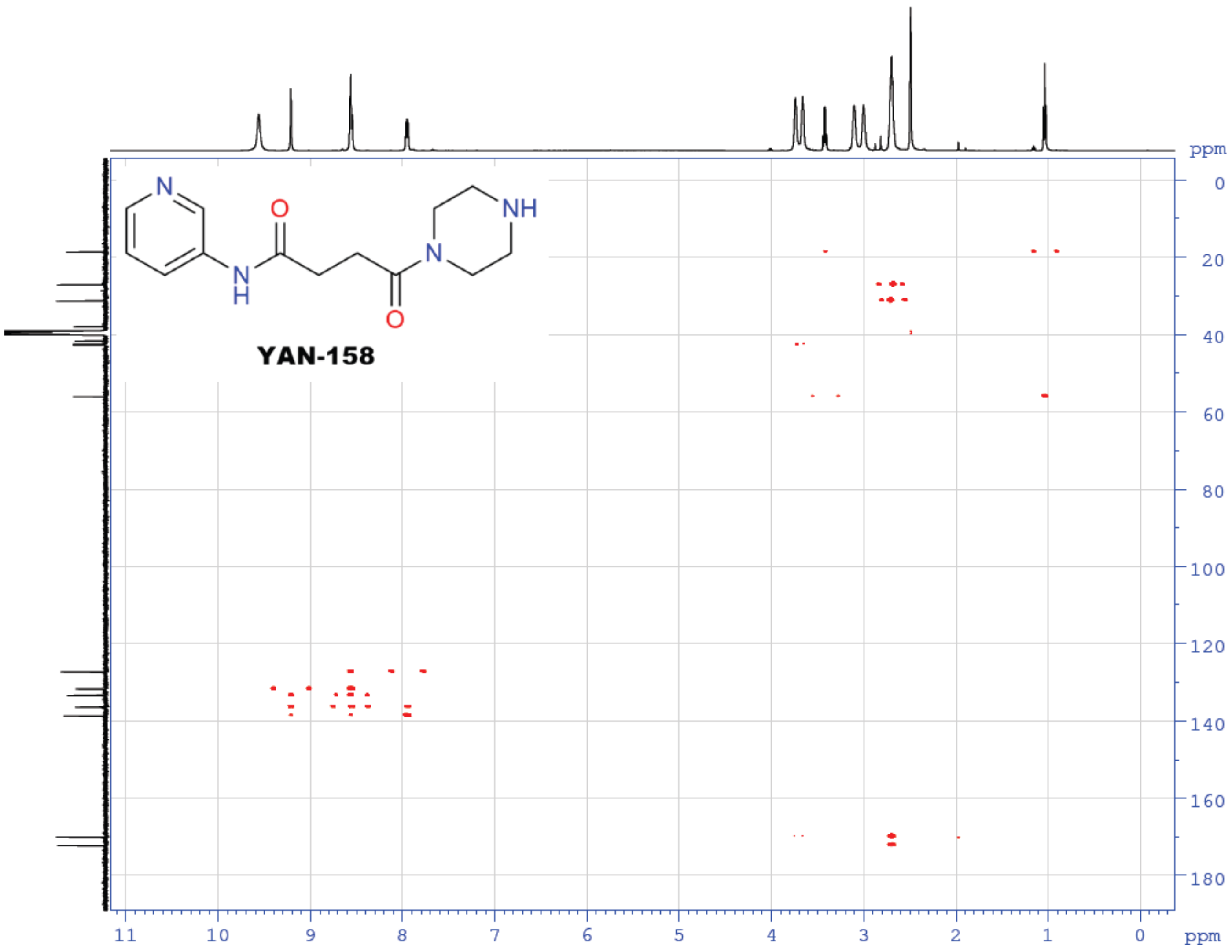

Figure S71. YAN-158 — $^{1}H \rightarrow ^{13}C$ HMBC NMR spectrum

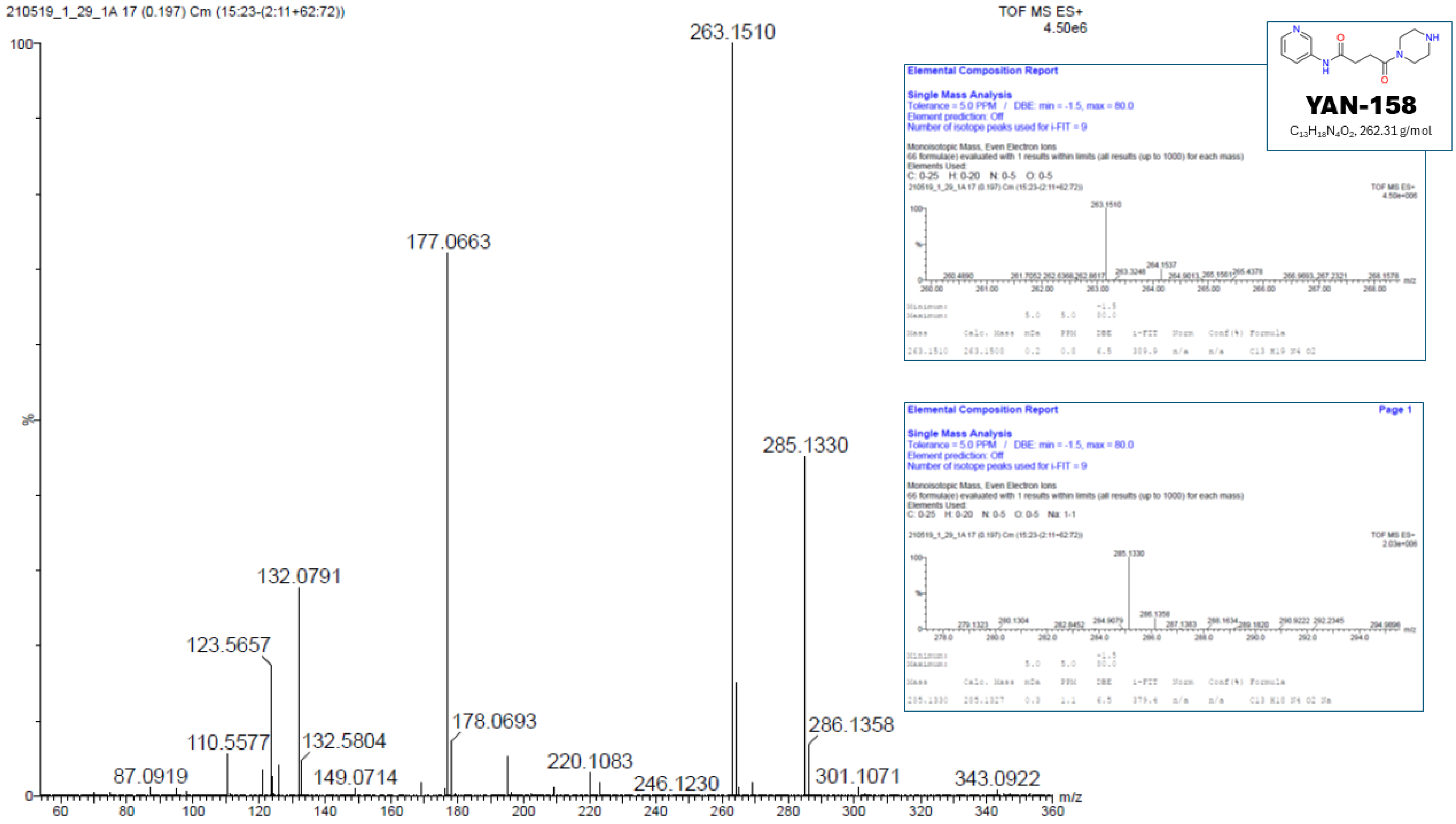

Figure S72. YAN-158 — HRMS (+) spectrum

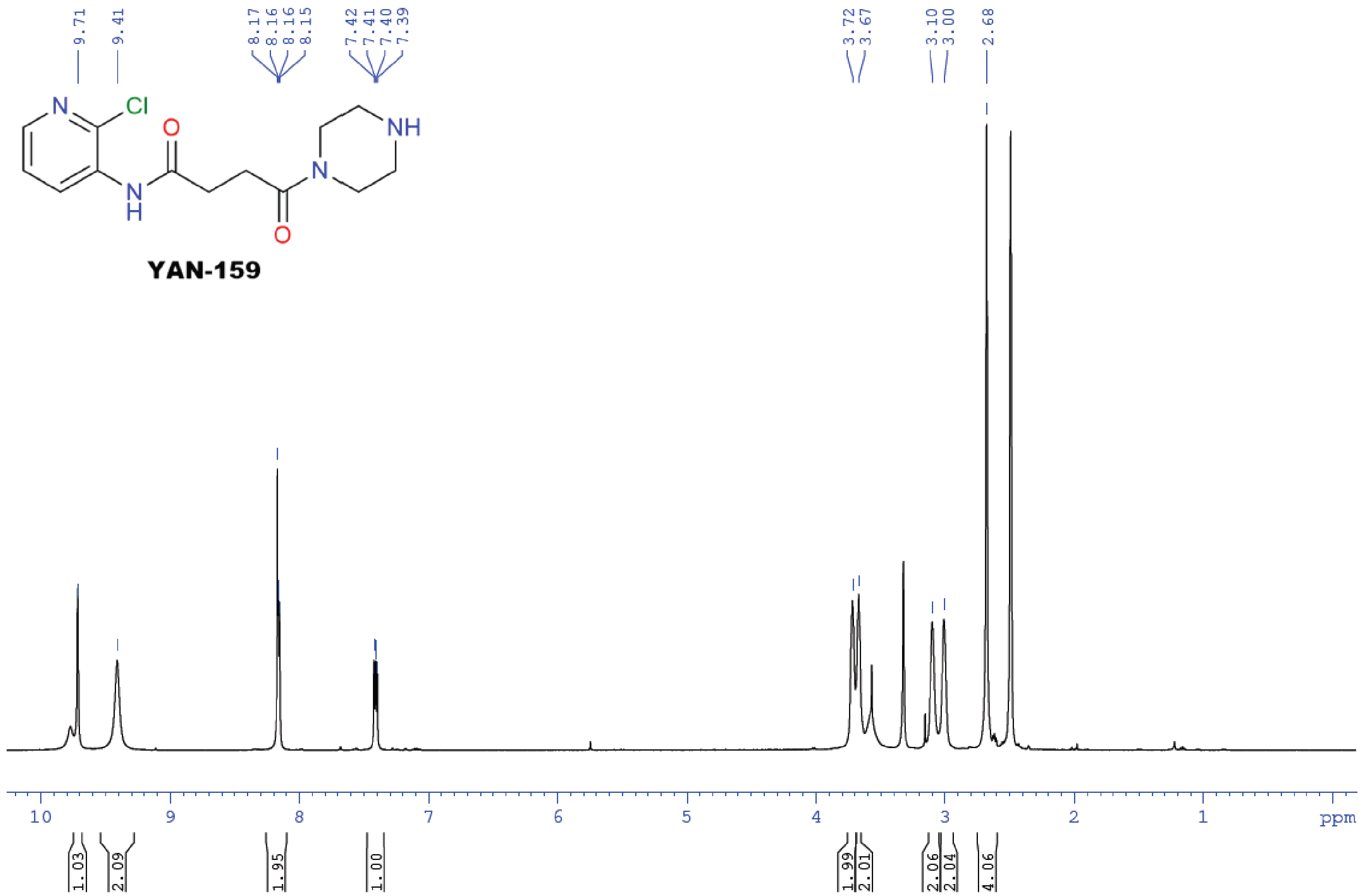

Figure S73. YAN-159 — ^{1}H NMR spectrum

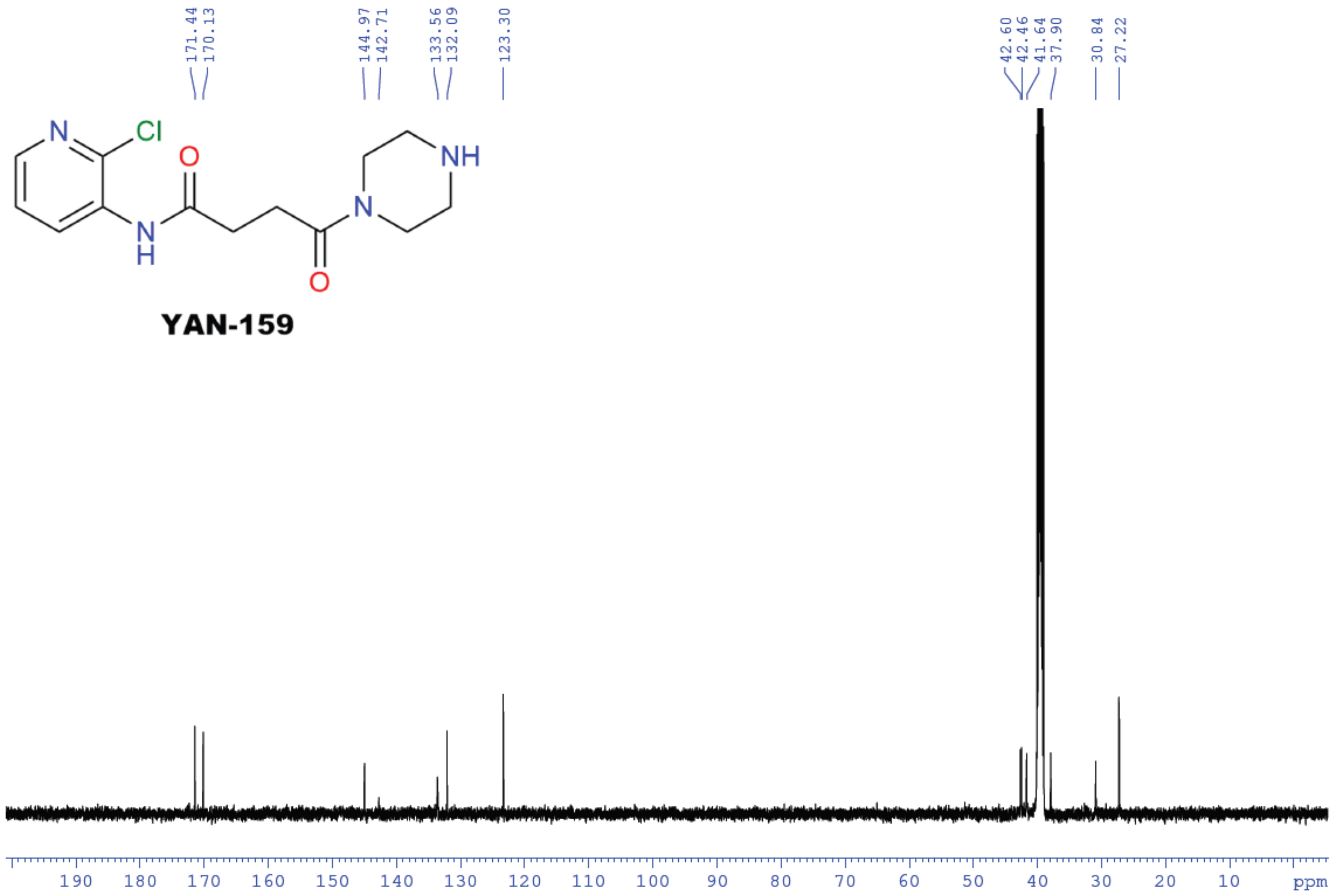

Figure S74. YAN-159 — ^{13}C NMR spectrum

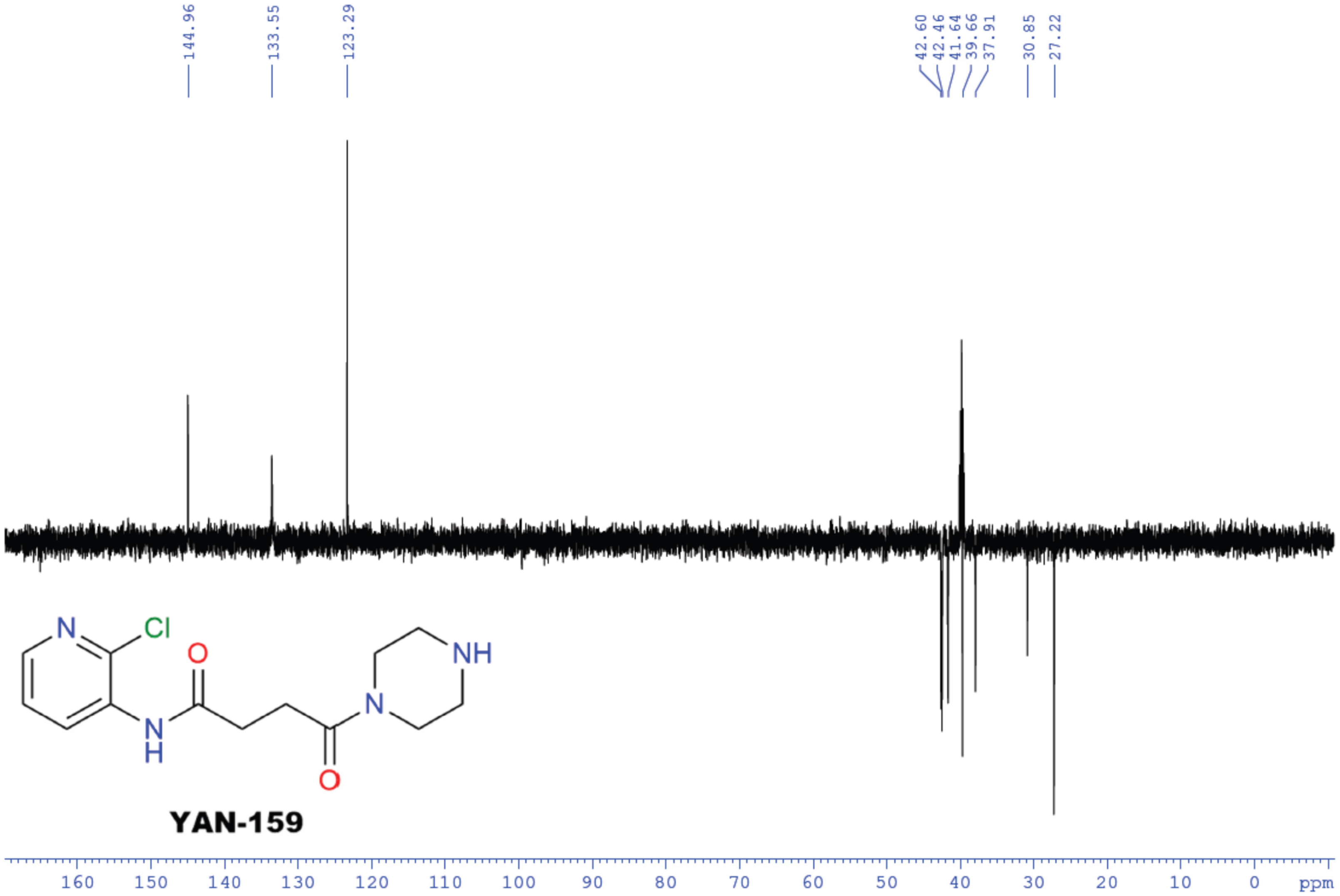

Figure S75. YAN-159 — DEPT NMR spectrum

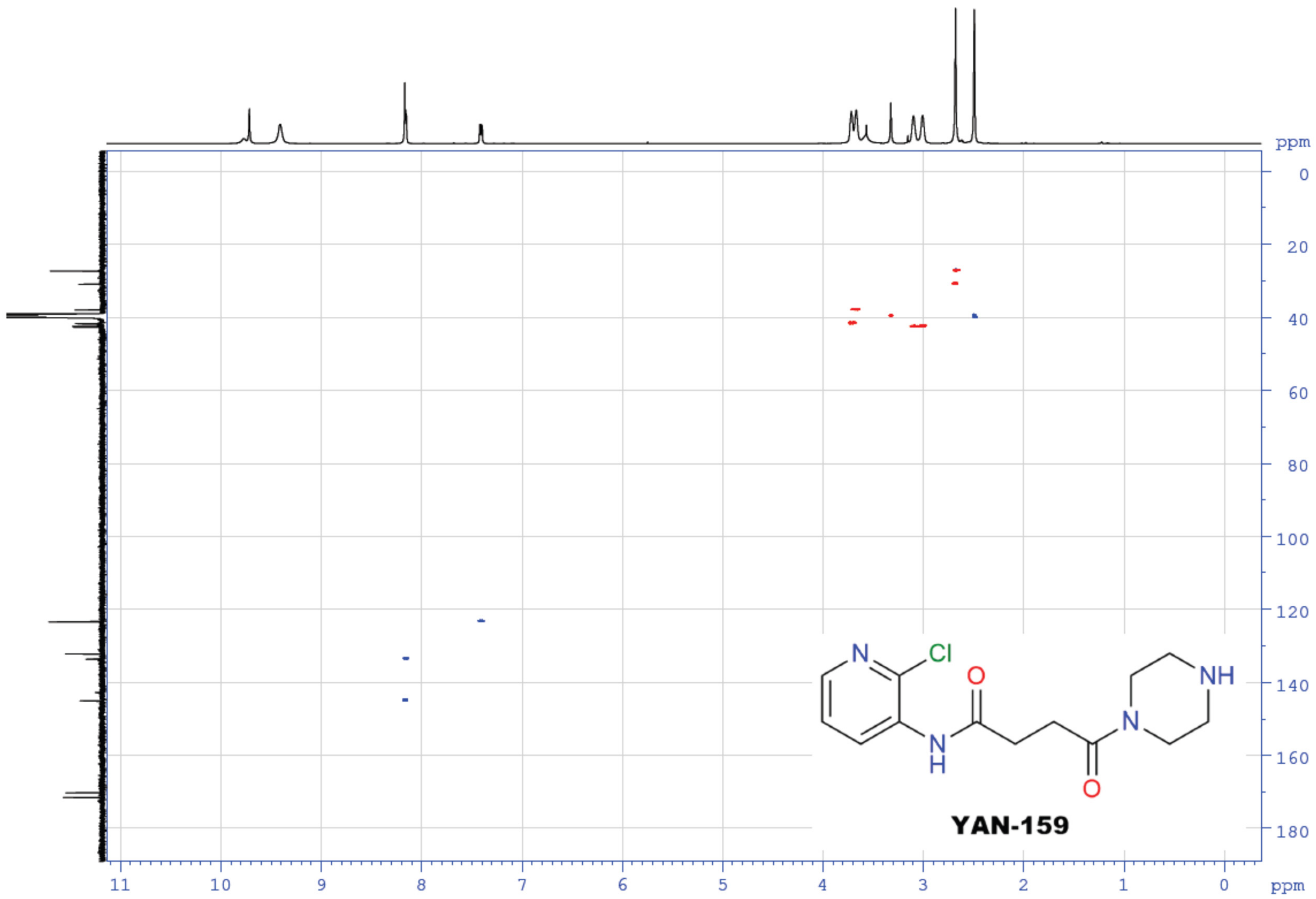

Figure S76. YAN-159 — ^{1}H-^{13}C HSQC NMR spectrum

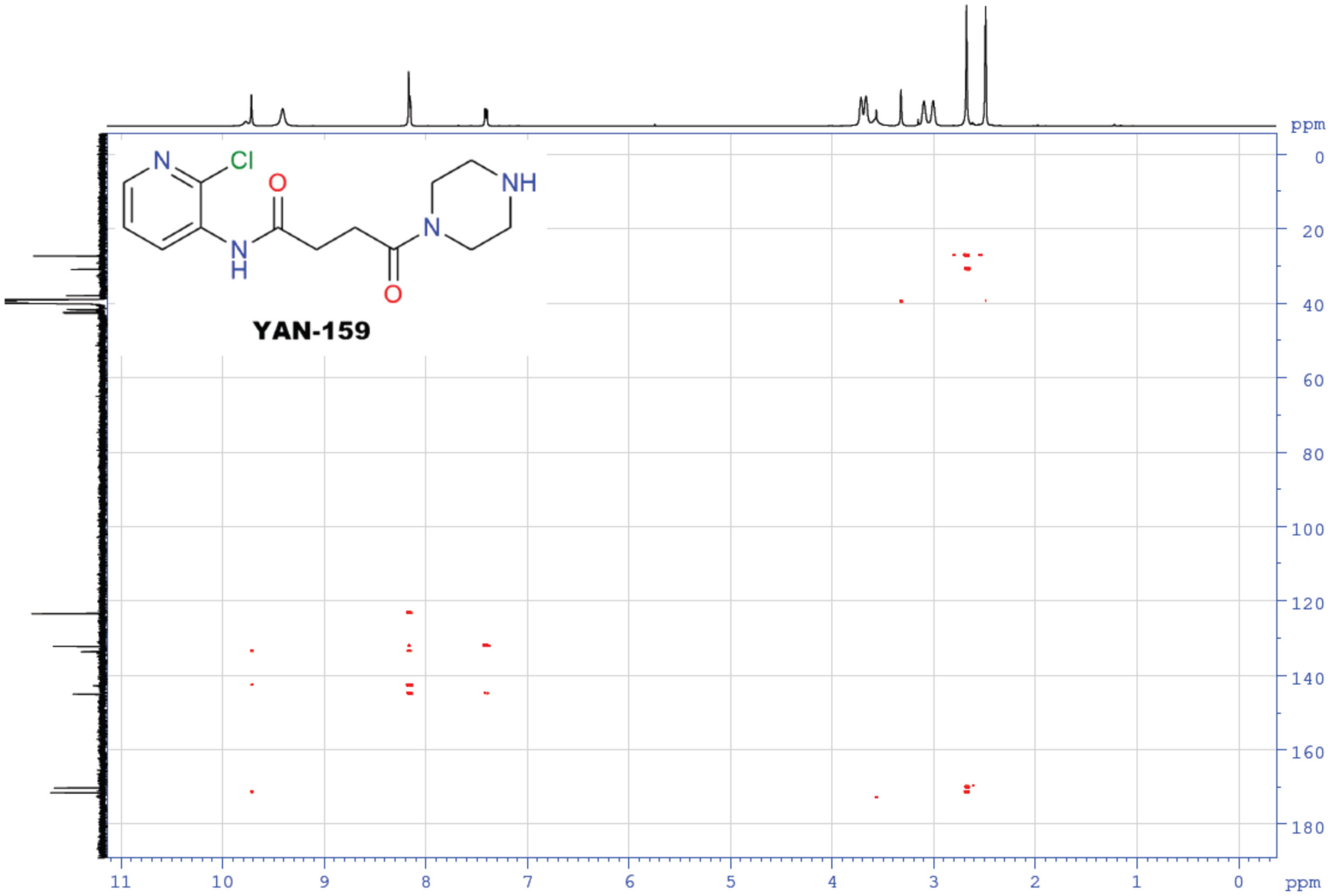

Figure S77. YAN-159 — $^{1}H \rightarrow ^{13}C$ HMBC NMR spectrum

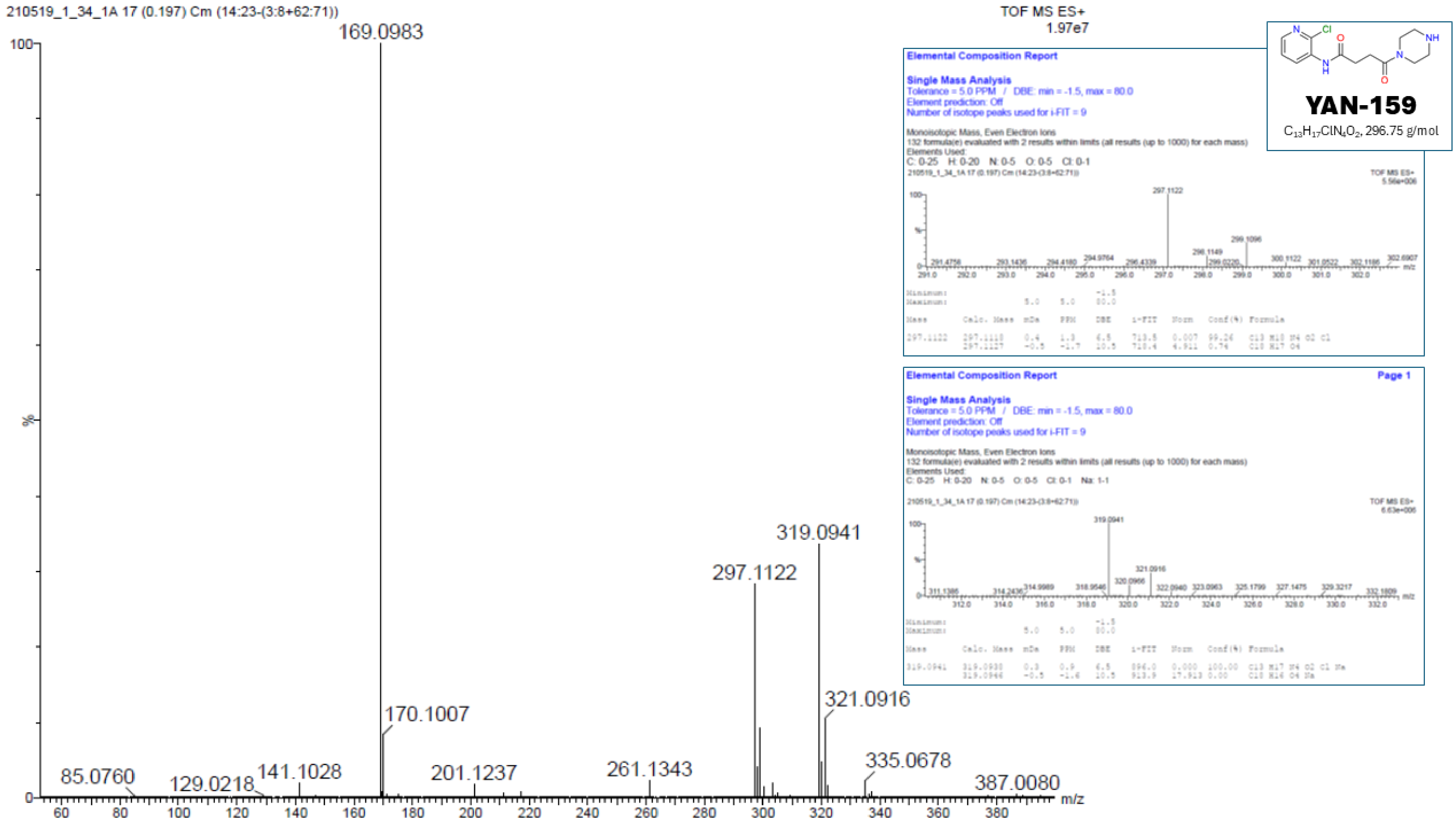

Figure S78. YAN-159 — HRMS (+) spectrum

SAMPLE INFORMATION

Sample Name:	YAN 151 297/54
Sample Type:	Unknown
Vial:	5
Injection Volume:	3,00 ul
Run Time:	15,0 Minutes
Channel Name:	Extract 278,0
Proc. Chnl. Descr.:	PDA 278,0 nm (PDA 190.0 to 400.0 nm at 1.2 nm)

Auto-Scaled Chromatogram

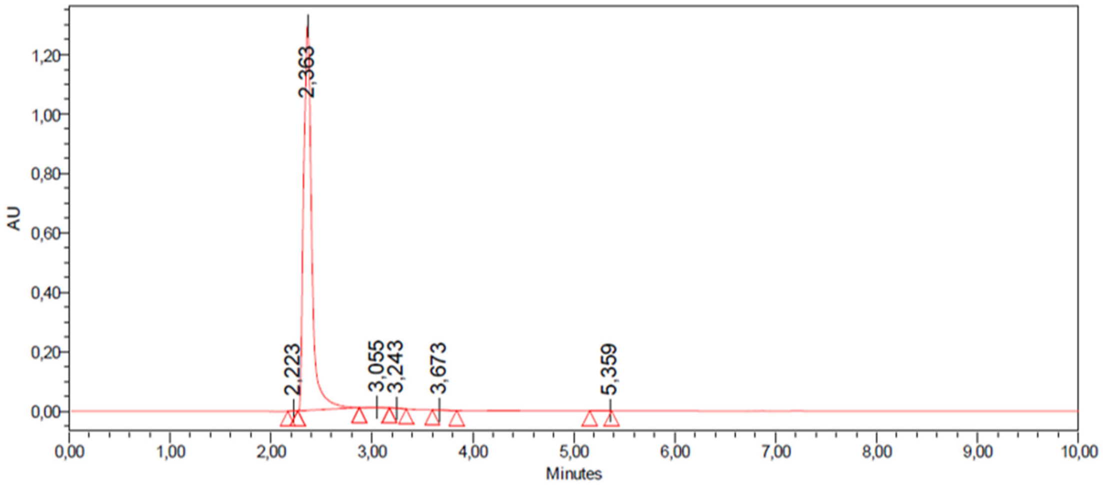

Peak Results

	RT	Area	% Area
1	2,223	2530	0,04
2	2,363	7202033	99,67
3	3,055	13242	0,18
4	3,243	4022	0,06
5	3,673	2660	0,04
6	5,359	1324	0,02

Figure S79. YAN-151 — HPLC

SAMPLE INFORMATION

Sample Name:	YAN 152 297/62/1
Sample Type:	Unknown
Vial:	11
Injection Volume:	3,00 ul
Run Time:	15,0 Minutes
Channel Name:	Extract 278,0
Proc. Chnl. Descr.:	PDA 278,0 nm (PDA 190.0 to 400.0 nm at 1.2 nm)

Auto-Scaled Chromatogram

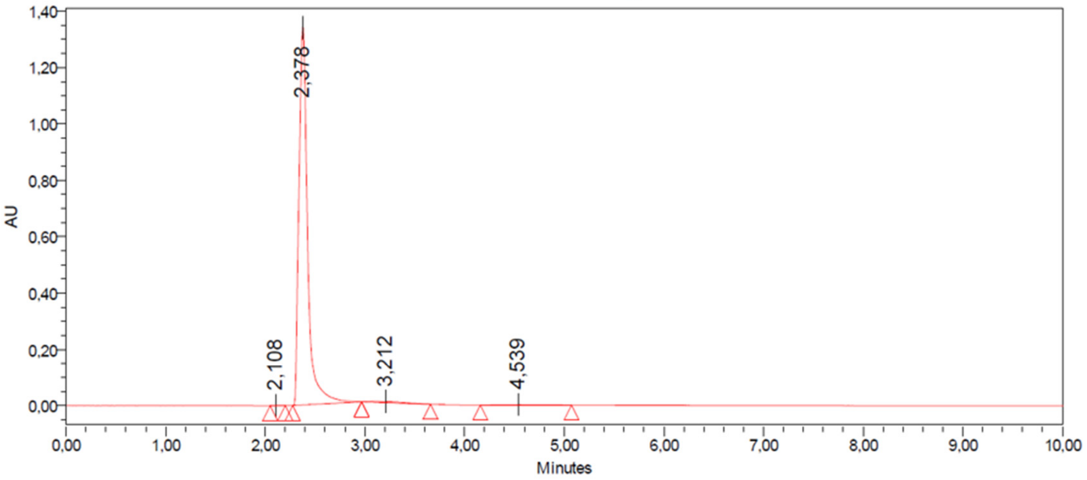

Peak Results

	RT	Area	% Area
1	2,108	3235	0,04
2	2,378	8084696	98,35
3	3,212	86187	1,05
4	4,539	46106	0,56

Figure S80. YAN-152 — HPLC

SAMPLE INFORMATION

Sample Name:	YAN 153 297/70
Sample Type:	Unknown
Vial:	2
Injection Volume:	3,00 ul
Run Time:	15,0 Minutes
Channel Name:	Extract 278,0
Proc. Chnl. Descr.:	PDA 278,0 nm (PDA 190.0 to 400.0 nm at 1.2 nm)

Auto-Scaled Chromatogram

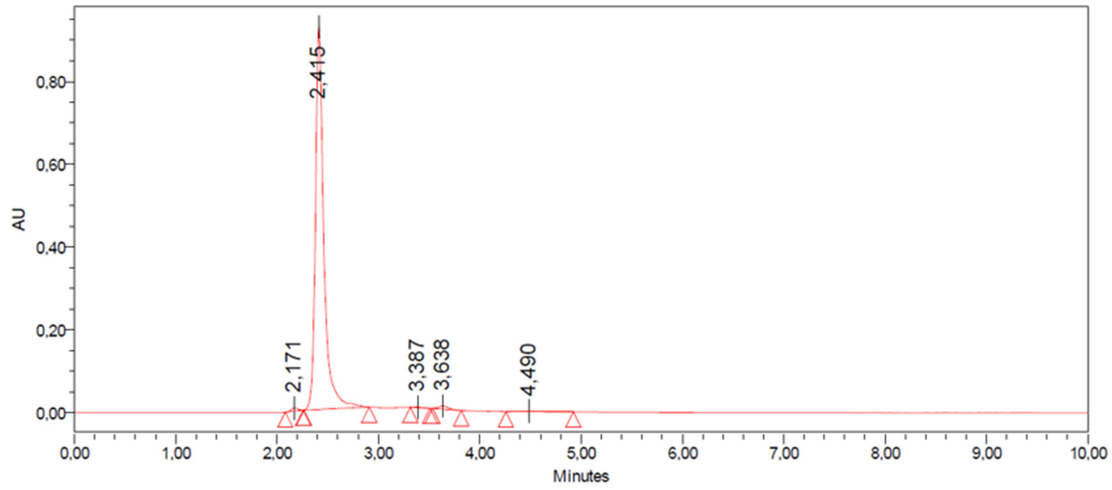

Peak Results

	RT	Area	% Area
1	2,171	37571	0,69
2	2,415	5307552	97,87
3	3,387	10949	0,20
4	3,638	52242	0,96
5	4,490	14651	0,27

Figure S81. YAN-153 — HPLC

SAMPLE INFORMATION

Sample Name:	YAN 154 297/76/1
Sample Type:	Unknown
Vial:	6
Injection Volume:	3,00 ul
Run Time:	15,0 Minutes
Channel Name:	Extract 278,0
Proc. Chnl. Descr.:	PDA 278,0 nm (PDA 190.0 to 400.0 nm at 1.2 nm)

Auto-Scaled Chromatogram

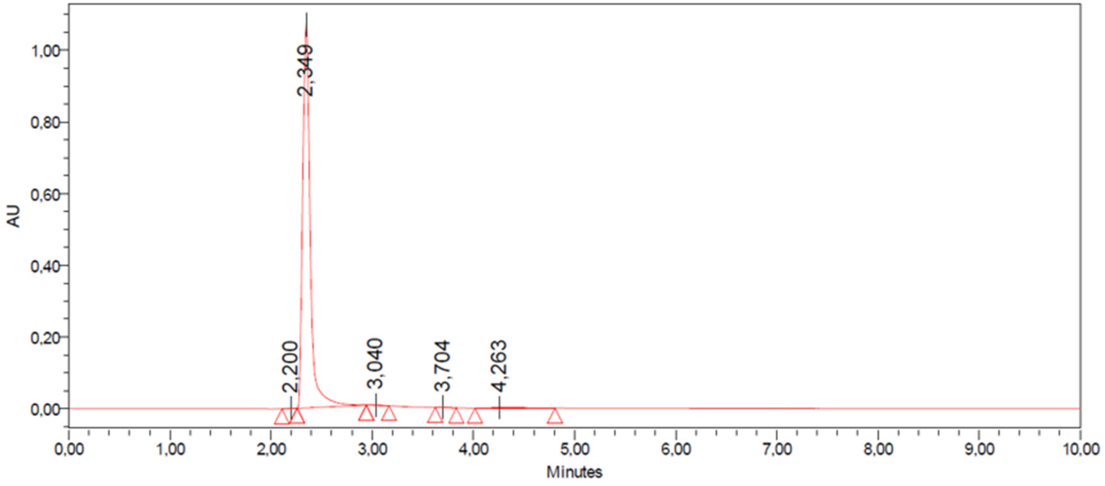

Peak Results

	RT	Area	% Area
1	2,200	3259	0,06
2	2,349	5578797	99,22
3	3,040	6558	0,12
4	3,704	1564	0,03
5	4,263	32615	0,58

Figure S82. YAN-154 — HPLC

SAMPLE INFORMATION

Sample Name:	YAN 155 297/86
Sample Type:	Unknown
Vial:	10
Injection Volume:	3,00 ul
Run Time:	15,0 Minutes
Channel Name:	Extract 278,0
Proc. Chnl. Descr.:	PDA 278,0 nm (PDA 190.0 to 400.0 nm at 1.2 nm)

Auto-Scaled Chromatogram

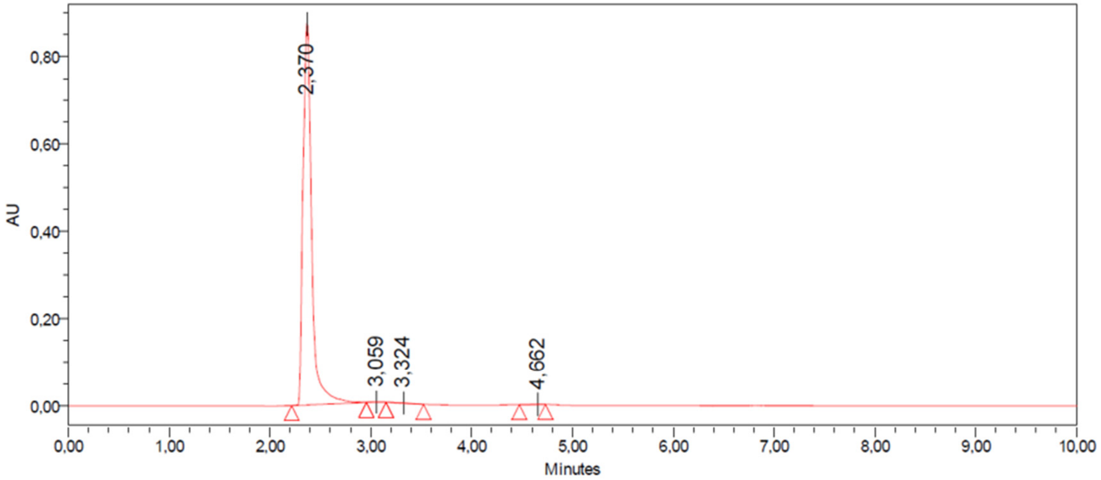

Peak Results

	RT	Area	% Area
1	2,370	5207002	99,74
2	3,059	2978	0,06
3	3,324	6937	0,13
4	4,662	3625	0,07

Figure S83. YAN-155 — HPLC

SAMPLE INFORMATION

Sample Name:	YAN 156 297/090/3
Sample Type:	Unknown
Vial:	9
Injection Volume:	3,00 ul
Run Time:	15,0 Minutes
Channel Name:	Extract 278,0
Proc. Chnl. Descr.:	PDA 278,0 nm (PDA 190.0 to 400.0 nm at 1.2 nm)

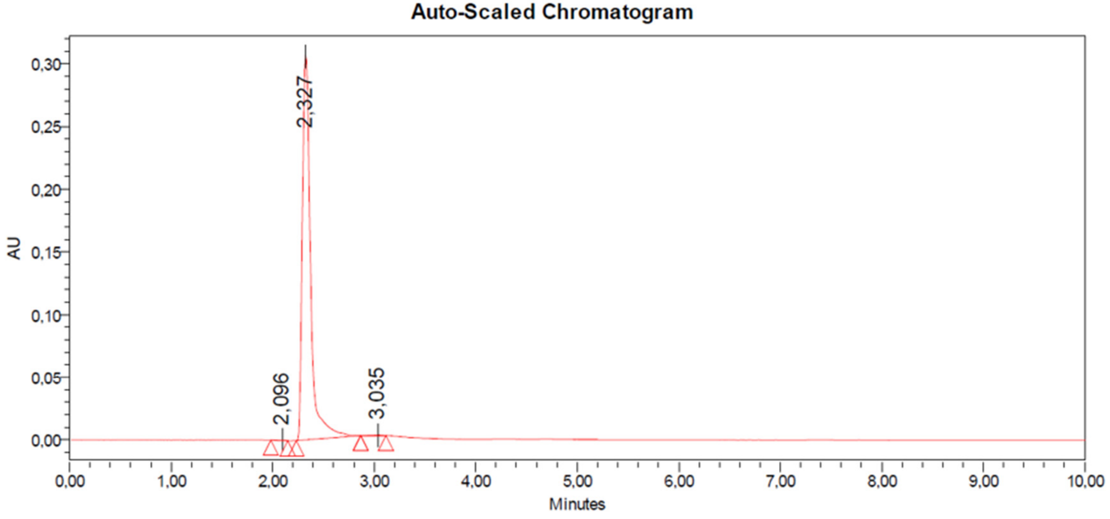

Peak Results

	RT	Area	% Area
1	2,096	2257	0,13
2	2,327	1737576	99,70
3	3,035	3047	0,17

Figure S84. YAN-156 — HPLC

SAMPLE INFORMATION

Sample Name:	YAN 157 297/081/2
Sample Type:	Unknown
Vial:	8
Injection Volume:	3,00 ul
Run Time:	15,0 Minutes
Channel Name:	Extract 278,0
Proc. Chnl. Descr.:	PDA 278,0 nm (PDA 190.0 to 400.0 nm at 1.2 nm)

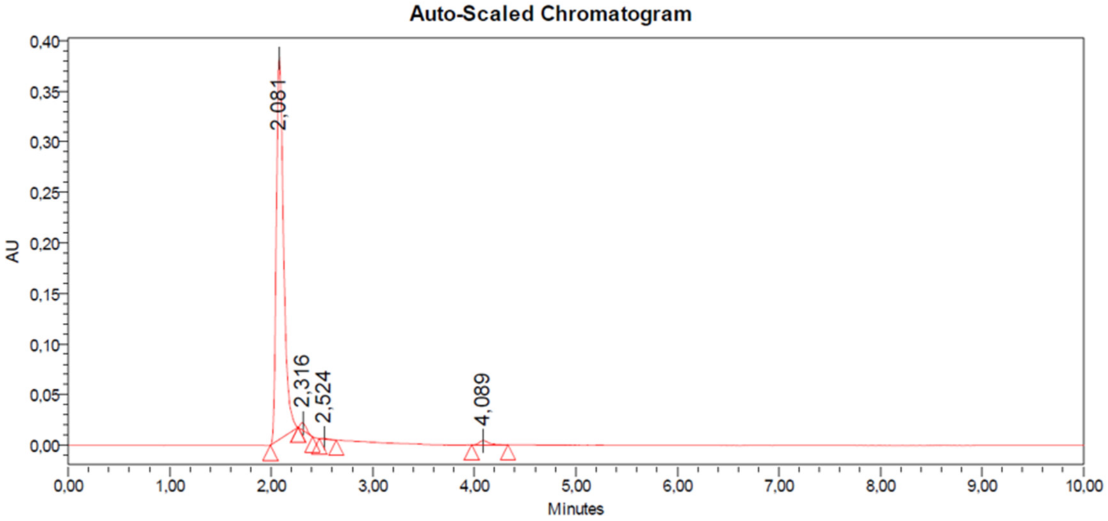

Peak Results

	RT	Area	% Area
1	2,081	1879949	96,88
2	2,316	30241	1,56
3	2,524	3267	0,17
4	4,089	26977	1,39

Figure S85. YAN-157 — HPLC

SAMPLE INFORMATION

Sample Name:	YAN 158 1/29/1
Sample Type:	Unknown
Vial:	4
Injection Volume:	3,00 ul
Run Time:	15,0 Minutes
Channel Name:	Extract 278,0
Proc. Chnl. Descr.:	PDA 278,0 nm (PDA 190.0 to 400.0 nm at 1.2 nm)

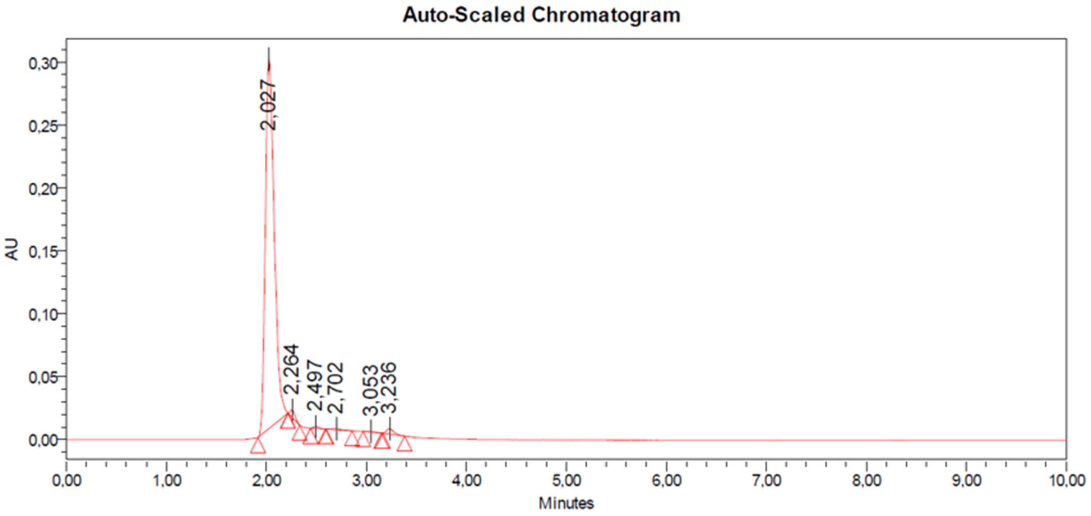

Peak Results

	RT	Area	% Area
1	2,027	1798773	97,00
2	2,264	20381	1,10
3	2,497	5960	0,32
4	2,702	6283	0,34
5	3,053	2302	0,12
6	3,236	20751	1,12

Figure S86. YAN-158 — HPLC

SAMPLE INFORMATION

Sample Name:	YAN 159 297/76/1
Sample Type:	Unknown
Vial:	7
Injection Volume:	3,00 ul
Run Time:	30,0 Minutes
Channel Name:	Extract 278,0
Proc. Chnl. Descr.:	PDA 278,0 nm (PDA 190.0 to 400.0 nm at 1.2 nm)

Auto-Scaled Chromatogram

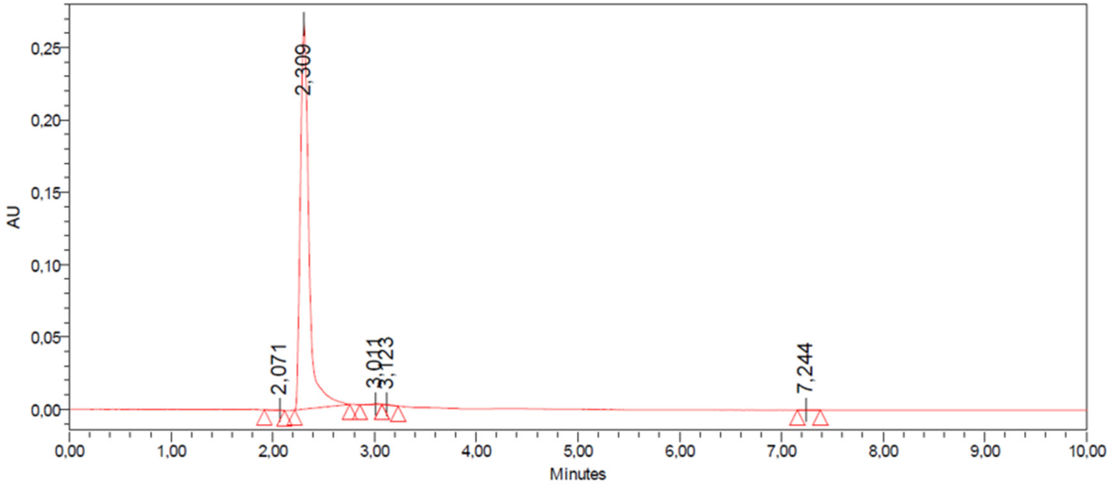

Peak Results

	RT	Area	% Area
1	2,071	1842	0,12
2	2,309	1563244	99,56
3	3,011	2184	0,14
4	3,123	1545	0,10
5	7,244	1288	0,08

Figure S87. YAN-159 — HPLC

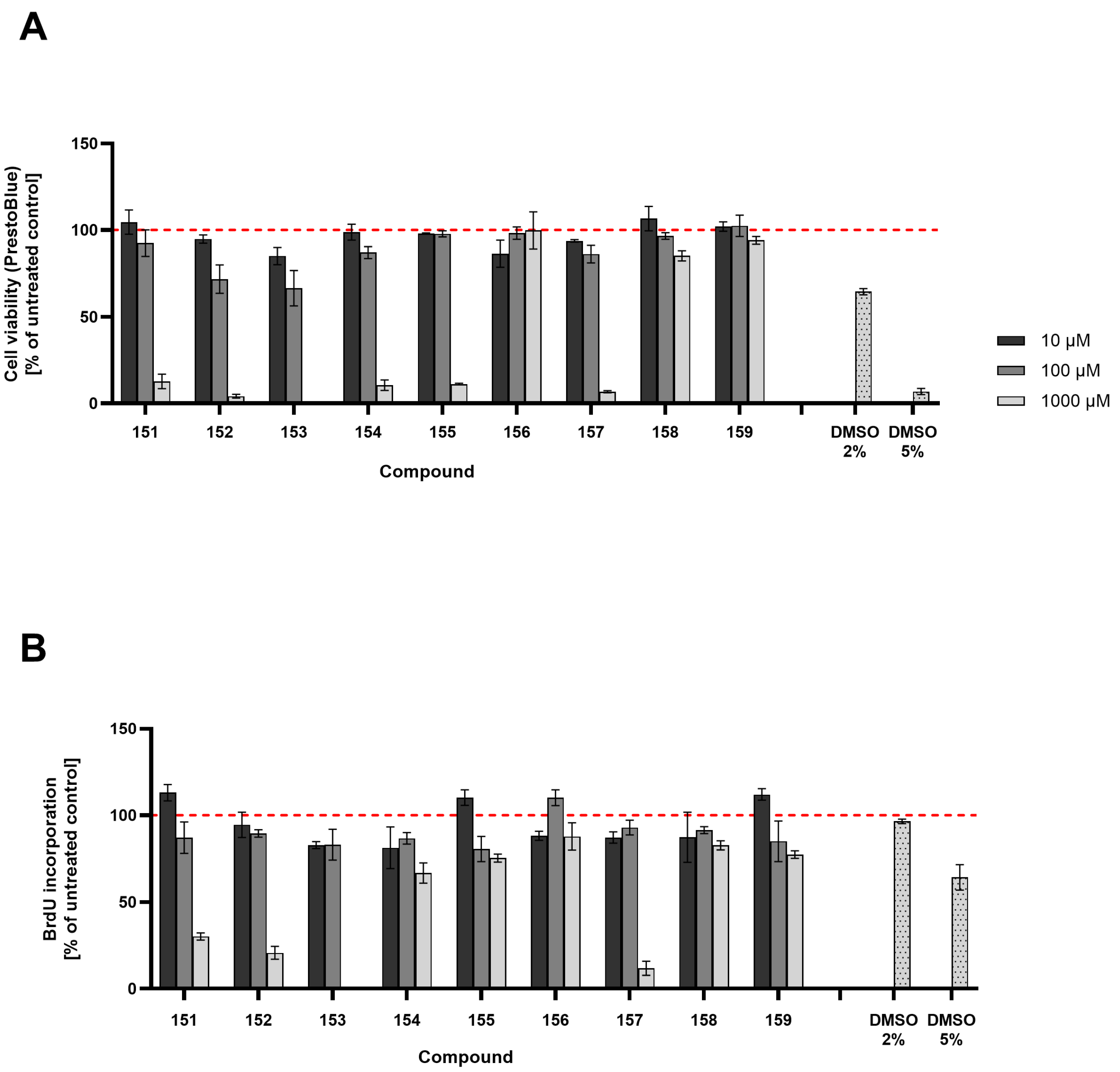

Figure S88. Effect of compounds **151–159** on cell viability and proliferation in CHO-H_4R cells. (**A**) Cell viability assessed by PrestoBlue assay and (**B**) cell proliferation assessed by BrdU incorporation assay, following incubation with 10, 100 and 1000 µM of each compound. Data are expressed as percentage of the untreated control (dashed line, 100%). Vehicle controls (dotted bars) correspond to the DMSO concentrations present at the highest compound concentration: 5% v/v for compounds **151–157** (20 mM stock) and 2% v/v for compound **158** (50 mM stock); compound **159** (100 mM stock) contained ~1% v/v DMSO. DMSO alone markedly reduced both parameters (5% DMSO: 6.9 ± 3.0% viability and 64.3 ± 12.7% BrdU incorporation; 2% DMSO: 64.5 ± 3.0% and 96.5 ± 2.4%, respectively), indicating that the decrease observed at 1 mM for most compounds is largely attributable to solvent toxicity rather than to the compounds themselves. For compound **153** at 1 mM, the signal in both assays was at or below the blank (viability −0.1%, BrdU incorporation −6.8% of control); the corresponding bars are therefore not visible. At the working concentration of 10 µM, viability ranged from 85 to 107% and BrdU incorporation from 81 to 113% of control for all compounds. Bars represent mean ± SEM of three replicates.
